# Postpartum Primary Care Engagement Using Default Scheduling and Tailored Messaging: A Randomized Clinical Trial

**DOI:** 10.1001/jamanetworkopen.2024.22500

**Published:** 2024-07-16

**Authors:** Mark A. Clapp, Alaka Ray, Pichliya Liang, Kaitlyn E. James, Ishani Ganguli, Jessica L. Cohen

**Affiliations:** 1 Department of Obstetrics and Gynecology, Massachusetts General Hospital, Boston; 2 Harvard Medical School, Boston, Massachusetts; 3 Department of Medicine, Massachusetts General Hospital, Boston; 4 Department of Medicine, Brigham & Women’s Hospital, Boston, Massachusetts; 5 Department of Global Health and Population, Harvard T. H. Chan School of Public Health, Boston, Massachusetts

## Abstract

**Importance:**

More than 30% of pregnant people have at least 1 chronic medical condition, and nearly 20% develop gestational diabetes or pregnancy-related hypertension, increasing the risk of future chronic disease. While these individuals are often monitored closely during pregnancy, they face major barriers when transitioning to primary care following delivery, due in part to a lack of health care support for this transition.

**Objective:**

To evaluate the impact of an intervention designed to improve postpartum primary care engagement by reducing patient administrative burden and information gaps.

**Design, Setting, and Participants:**

An individual-level randomized clinical trial was conducted from November 3, 2022, to October 11, 2023, at 1 hospital-based and 5 community-based outpatient obstetric clinics affiliated with a large academic medical center. Participants included English- and Spanish-speaking pregnant or recently postpartum adults with obesity, anxiety, depression, diabetes, chronic hypertension, gestational diabetes, or pregnancy-related hypertension and a primary care practitioner (PCP) listed in their electronic health record.

**Intervention:**

A behavioral economics–informed intervention bundle, including default scheduling of postpartum PCP appointments and tailored messages.

**Main Outcome and Measures:**

Completion of a PCP visit for routine or chronic condition care within 4 months of delivery was the primary outcome, ascertained directly by reviewing the patient’s electronic health record approximately 5 months after their estimated due date. Intention-to-treat analysis was conducted.

**Results:**

A total of 360 patients were randomized (control, 176; intervention, 184). Individuals had a mean (SD) age of 34.1 (4.9) years and median gestational age of 36.3 (IQR, 34.0-38.6) weeks at enrollment. The distribution of self-reported race and ethnicity was 6.8% Asian, 7.4% Black, 68.6% White, and 15.0% multiple races or other. Most participants (75.4%) had anxiety or depression, 16.1% had a chronic or pregnancy-related hypertensive disorder, 19.5% had preexisting or gestational diabetes, and 40.8% had a prepregnancy body mass index of 30 or greater. Medicaid was the primary payer for 21.2% of patients. Primary care practitioner visit completion within 4 months occurred in 22.0% (95% CI, 6.4%-28.8%) of individuals in the control group and 40.0% (95% CI, 33.1%-47.4%) in the intervention group. In regression models accounting for randomization strata, the intervention increased PCP visit completion by 18.7 percentage points (95% CI, 9.1-28.2 percentage points). Intervention participants also had fewer postpartum readmissions (1.7% vs 5.8%) and increased receipt of the following services by a PCP: blood pressure screening (42.8% vs 28.3%), weight assessment (42.8% vs 27.7%), and depression screening (32.8% vs 16.8%).

**Conclusions and Relevance:**

The findings of this randomized clinical trial suggest that the current lack of support for postpartum transitions to primary care is a missed opportunity to improve recently pregnant individual’s short- and long-term health. Reducing patient administrative burdens may represent relatively low-resource, high-impact approaches to improving postpartum health and well-being.

**Trial Registration:**

ClinicalTrials.gov Identifier: NCT05543265

## Key Points

**Question:**

What is the impact of a behavioral economic intervention designed to reduce patient administrative burden and information gaps on primary care practitioner (PCP) visit completion in the postpartum period?

**Findings:**

In this randomized clinical trial of 360 patients with or at risk for a chronic condition, default PCP scheduling, tailored messages, and reminders increased postpartum PCP visit rates by 19 percentage points. The intervention also resulted in more individuals receiving important screening tests and services provided by their PCP.

**Meaning:**

The findings of this trial suggest that a multifaceted and relatively low-resource behavioral economic intervention may improve postpartum health and well-being.

## Teaser

This randomized clinical trial examines receipt of postpartum primary care through default primary care practitioner scheduling and tailored messages.

## Introduction

Although the chronic disease burden in pregnancy is high and increasing in the US, most people with chronic conditions do not successfully transition to primary care management following delivery.^1,2,3,4,5,6,7,8,9^ More than 30% of pregnant people have diabetes, hypertension, or obesity, and 11% to 22% have anxiety or depression.^10,11,12^ Furthermore, common pregnancy-related conditions (eg, gestational diabetes and pregnancy-related hypertension), which combined affect nearly 20% of pregnancies, confer an increased risk of developing chronic disease.^13,14,15,16,17,18^ Strong evidence underpins the benefits of managing chronic conditions through primary care and of managing these conditions earlier in life.^19,20,21,22^ However, while pregnant people with these conditions are often carefully monitored during pregnancy, many receive no routine care after their pregnancy, and nearly half of those with chronic conditions do not see their primary care practitioner (PCP) at all in the postpartum year.^23^ The abrupt drop off from high health system engagement and motivation during pregnancy to limited or no health care encounters post partum has been termed a postpartum cliff.^24^ Low rates of postpartum primary care engagement reflect a missed opportunity to improve the prevention and management of chronic disease.

Postpartum transitions from obstetric to primary care are encouraged by guidelines yet stymied by numerous barriers. Specifically, the American College of Obstetrics and Gynecology^25^ recommends that all individuals have a comprehensive postpartum visit within 12 weeks of their delivery; at that time, obstetric care clinicians typically counsel patients on the importance of ongoing primary care follow-up. Yet, a range of systemic, financial, and behavioral barriers often prevents postpartum people from successfully transitioning to primary care.^26,27,28,29,30^ Patient administrative burden (eg, appointment scheduling, information seeking, and insurance/billing issues) is increasingly recognized as a barrier to accessing care.^31^ In a 2021 survey, 33% of patients reported that they delayed or did not seek health care because of the administrative burden.^31^ The results of this burden may be amplified in the postpartum period when new parents are sleep deprived and face many competing demands, including caring for their newborn and family. This study aimed to increase patient engagement in primary care after the immediate postpartum period for pregnant individuals with conditions that convey a long-term health risk by reducing administrative burden and motivating continued health activation through an intervention based on insights from behavioral economics.

## Methods

### Study Design

This study was an individual-level, 2-group, 1:1 stratified randomized clinical trial of the effectiveness of a behavioral economics–informed intervention to increase the rate of postpartum primary care visit completion. The study was conducted at 1-hospital based obstetric clinic and 5 community-based obstetric clinics from November 3, 2022, to October 11, 2023 (trial protocol and analysis plan included in Supplement 1). The Mass General Brigham Human Subjects Committee approved this study, and analyzed data were deidentified. Individuals provided verbal consent to participate and received financial compensation. The Consolidated Standards of Reporting Trials (CONSORT) reporting guideline was followed in reporting the study and its results.

### Patient Eligibility

Patients who had obesity (prepregnancy body mass index ≥30; calculated as weight in kilograms divided by height in meters squared), anxiety or depressive mood disorder, type 1 or 2 diabetes, chronic hypertension, gestational diabetes, or pregnancy-related hypertension listed in their electronic health record (EHR) were eligible to participate. Patients at high risk for hypertensive disorders of pregnancy, defined as those who would be recommended for low-dose aspirin by US Preventative Services Task Force guidelines, were eligible. Patients with these conditions were prioritized for inclusion in the study as they were more likely to have ongoing care needs after pregnancy. Also, this study was limited to patients who had a PCP listed or identified in their EHR, as the barriers and solutions to postdelivery primary care reengagement are different than establishing care with a new PCP; a preliminary analysis of patients receiving obstetric care at the study institution revealed that 90% had a PCP listed in the EHR. Other eligibility criteria included (1) pregnant or recently post partum (defined as up to 2 weeks after their estimated due date [EDD]), (2) receipt of prenatal care at the study institution or its affiliated clinics, (3) enrolled and elected to receive messages in the study institution’s EHR patient portal, (4) primary language of English or Spanish, (5) age 18 years or older at the time of enrollment, and (6) not actively undergoing a workup for or known to have fetal demise at the time of enrollment.

### Enrollment and Randomization

Eligible patients were approached in person and via telephone during the eligibility window (up to 2 weeks after their EDD). Those who consented to participate in the study were also asked to consent to receive text (SMS) messages separately. Individuals were randomized using a randomization table created by the statistician (K.E.J.) and uploaded directly into the REDCap randomization module, which was blinded to the primary investigators and study staff. The assignment sequence was stratified by 2 variables that were determined a priori to be important to ensure balance: visit with a PCP within 3 years before the EDD and site of prenatal care (hospital campus vs community-based obstetric clinic). Patients were randomized after they consented and completed a baseline survey.

### Study Intervention

The intervention was designed to increase the rate of postpartum primary care visit completion within 4 months after the patient’s EDD. The bundle included a targeted introduction message about the importance of seeing their PCP after delivery and informed them that, to support them in this, a study staff member would be making an appointment on their behalf; they were allowed to opt out or communicate about scheduling preferences. For those who did not opt out, the study staff called the PCP office and requested that a health care maintenance or annual visit be scheduled within the target 4-month window. If a patient had already seen their PCP for an annual visit within the year, they were scheduled for this visit when they were next eligible (ie, 1 year after their last annual examination), even if outside the 4-month study follow-up period. For those who had appointments scheduled, study-specific appointment reminders were sent approximately 1 month after the EDD and 1 week before the scheduled appointment via the EHR patient portal and SMS, and both used salient labeling to describe the visit; examples are shown in the eFigure in Supplement 2. If the PCP worked in the same health system and an appointment was scheduled, an electronic message was sent to the PCP from the study staff about the appointment scheduled by the study staff. For those for whom an appointment could not be scheduled, similar reminders were sent on the importance of PCP follow-up and encouraged the patient to contact their PCP office directly to schedule. Reminders included best practice wording from behavioral economic nudge mega-studies, including that the appointment had been reserved for them.^32^ Using salient labeling, the appointment was described as the postpartum-to–primary care transition appointment. Patients in the control group received 1 message within 2 weeks of the EDD with a generic recommendation for PCP follow-up after delivery.

### Study Measures

The primary outcome was completing a primary care visit for routine or chronic condition care within 4 months of the patient’s EDD. Specifically, we considered the outcome to have occurred if the patient attended a health care maintenance (ie, annual examination) visit or a problem-based visit in which obesity, anxiety and/or depression, diabetes, or hypertension were addressed with a primary care clinician within 4 months after their EDD. This definition was chosen to include visits most likely to reflect primary care reengagement after delivery instead of a visit for an acute illness or issue. This time frame was selected for 2 reasons: to capitalize on the increased health activation and motivation that have been noted during pregnancy and because these patients were more likely to have conditions that required ongoing and active management outside of the traditional postpartum period (up to 12 weeks after delivery). We considered practitioners affiliated with internal medicine, family medicine, pediatric and adolescent medicine, and gynecology practices to provide primary care; however, we did not count designated postpartum visits to be primary care visits.

Alternative specifications for the primary outcome were compared in sensitivity analyses: (1) self-reported PCP visits within 4 months after the EDD, obtained from a survey sent approximately 5 months after the EDD; (2) primary outcome restricted to visits with the patient’s designated PCP; (3) primary outcome restricted to patients whose PCP was affiliated with the study institution’s health system; (4) primary outcome expanded to include any PCP visit (not only routine or chronic condition care) within 4 months after a patient’s EDD; and (5) primary outcome expanded to include any completed or scheduled PCP visit within 1 year of a patient’s EDD.

We examined secondary outcomes measuring unscheduled care: obstetric triage visit, emergency department or urgent care use, and readmission within 4 months after the delivery. We also measured the likelihood of a patient having a PCP visit that included specific primary care services within 4 months: weight screening, blood pressure screening, mood screening, plan for diabetes screening, plan for mental health care, and contraception planning. Content of care outcomes were also compared within population subgroups related to the eligibility health condition. All outcomes are defined in detail in eTable 1 in Supplement 2.

The primary and most secondary outcomes were ascertained directly by reviewing the patient’s EHR approximately 5 months after their EDD. Study staff that performed the review were blinded to the group assignment. Secondary self-reported outcomes were obtained by an electronic survey sent to patients approximately 5 months after their EDD.

### Sample Size Calculation

Based on a historical cohort, we estimated that 33% of the targeted study population would have a PCP visit within 4 months of delivery. We estimated the intervention would increase the rate of PCP visit attendance by at least 15 percentage points (pp), a conservative estimate based on a prior study that examined default scheduling of postpartum obstetric care appointments (24-pp increase).^33^ Assuming an α level of .05 and power of 80% and using a 2-sided *z* test, 334 patients were needed to detect a 15-pp difference. To account for individuals who may be lost to follow-up or withdraw, we planned to randomize 360 patients.

### Statistical Analysis

Patients were examined by intention-to-treat analysis. Patients who were lost to follow-up (ie, transferred obstetric care before delivery) or withdrew before the outcome assessment were excluded. Baseline patient characteristics and the percentage of patients who accessed the study messages in the EHR patient portal are reported. Primary and secondary outcomes were compared using χ^2^, *t* tests, and Fisher exact test, where appropriate. The pp difference in outcomes between the 2 groups was estimated using a linear probability regression model that included 2 indicator terms for the randomization strata, which were defined a priori.

A heterogeneity analysis was performed to understand the potential impact of the intervention among patient factors known or hypothesized to be disproportionately affected by administrative burdens. The primary outcome was compared among subgroups based on site of prenatal care (hospital- vs community-based clinic), chronic conditions (anxiety and/or depression, diabetes, hypertension, obesity, and multimorbidity, defined as >1 of the listed conditions), race (self-described Asian, Black, White, other [including American Indian or Alaska Native and Native Hawaiian or Other Pacific Islander], or multiple) and ethnicity (self-described Hispanic or non-Hispanic), individual earnings/income (≤$30 000, $30 001-$75 000, or >$75 000), primary payer for delivery hospitalization (Medicaid or private/other), and self-reported physical and mental health status at the time of enrollment.^31,34^ As known racial disparities exist in maternal health outcomes, including maternal morbidity and mortality, we examined the outcome by race to understand whether there was a differential impact among subgroups.

Stata, version 16.1 (StataCorp LLC) was used for the analysis. *P* values are reported for the primary outcome; *P <* .05 was considered statistically significant. As this project was not designed to have statistical power to detect the intervention’s impact on secondary outcomes or differences across subgroups, multiple hypothesis testing was not planned or prespecified. Results from secondary analyses are presented with 95% CIs that were not adjusted for multiple hypothesis testing. These secondary analyses should be considered exploratory, and results may not be reproducible.

## Results

**Figure 1.**
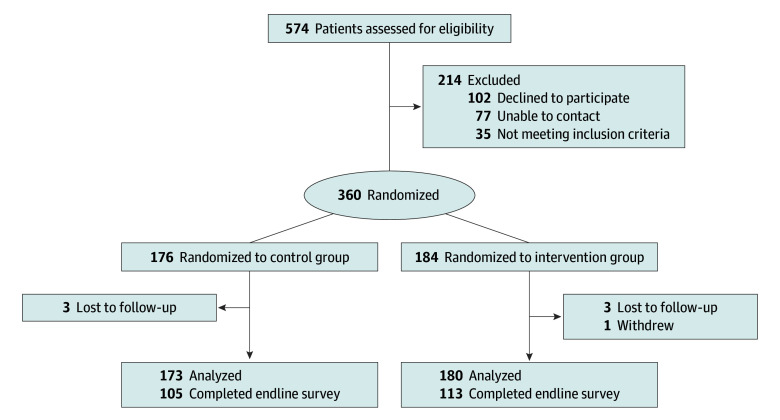
Patient Flowchart

Initially, 574 patients were identified as likely to be eligible based on predefined eligibility filters within the EHR (Figure 1). Upon EHR review, 35 individuals were determined ineligible. Of those confirmed eligible, 77 could not be contacted and 102 declined. Thus, 360 patients were randomized: 176 to the control group and 184 to the intervention group. Six patients were excluded from the final analysis because they transferred their care to another institution before delivery (3 in each group). One patient in the intervention group withdrew from the study before the end of the follow-up period. The final number of patients analyzed in each group was 173 in the control group and 180 in the intervention group. Among study participants, 345 of 353 (97.7%) accessed study-related messages in the online patient portal. The proportion of patients in the intervention group who received each component of the intervention bundle is included in eTable 2 in Supplement 2; the study staff scheduled appointments for 137 participants (76.1%), of whom only 6 (4.4%) did not present and did not cancel their appointment. The most common reason the study staff did not schedule an appointment was that a PCP appointment was already scheduled (21 of 43 [48.8%]) (eTable 3 in Supplement 2 provides the full list). Of all participants, 61.8% completed the online electronic survey 5 months after the EDD.

The intervention and control groups were balanced in all baseline patient characteristics (Table 1). Individuals included in the trial had a mean (SD) age of 34.1 (4.9) years and median gestational age of 36.3 (IQR, 34.0-38.6) weeks at enrollment. The distribution of self-reported race and ethnicity was 6.8% Asian, 7.4% Black, 68.6% White, and 15.0% multiple races or other; 2.3% declined to report their race. The distribution of self-reported ethnicities was 22.1% Hispanic and 75.4% non-Hispanic; 2.5% declined to report their ethnicity. Of the eligibility conditions, which were not mutually exclusive, 75.4% of all participants had anxiety or depression, 16.1% had a chronic or pregnancy-related hypertensive disorder, 19.5% had preexisting or gestational diabetes, and 40.8% had a prepregnancy body mass index of 30 or greater. Medicaid was the primary payer for the delivery encounter for 21.2% of patients. When surveyed, 11.6% reported their physical health and 19.6% reported their mental health as fair or poor. At enrollment, 34.3% of the participants had not seen any PCP within the previous 3 years and 29.2% were receiving obstetric care at one of the hospital’s satellite or affiliated health center clinics.

**Table 1.** Baseline Characteristics of the Analytical Sample

Characteristic	Patients, No. (%)	
	Control group (n = 173)	Intervention group (n = 180)
Patient age at estimated due date, mean (SD), y	34.0 (5.0)	34.2 (4.8)
Enrollment		
During pregnancy	170 (98.3)	173 (96.1)
Post partum	3 (1.7)	7 (3.9)
Gestational age at enrollment, mean (SD), d	254 (20)	255 (20)
Primary site of prenatal care		
Hospital-based clinic	121 (69.9)	129 (71.7)
Community-based clinic	52 (30.1)	51 (28.3)
PCP visit within 3 y prior to enrollment	121 (69.9)	111 (61.7)
Health condition		
Anxiety or depression	128 (74.0)	138 (76.7)
Chronic or gestational hypertensive disorder	26 (15.0)	31 (17.2)
Chronic or gestational diabetes	38 (22.0)	31 (17.2)
Obesity^a^	75 (43.4)	69 (38.3)
Race^b^		
Asian	13 (7.5)	11 (6.1)
Black	12 (6.9)	14 (7.8)
Multiple races or other^c^	28 (16.2)	25 (13.9)
White	115 (66.5)	127 (70.6)
Declined/not reported	5 (2.9)	3 (1.7)
Ethnicity^b^		
Hispanic	41 (23.7)	37 (20.6)
Non-Hispanic	127 (73.4)	139 (77.2)
Not reported	5 (2.9)	4 (2.2)
Preferred language^b^		
English	161 (93.1)	167 (92.8)
Spanish	12 (6.9)	13 (7.2)
Marital status^b^		
Married	125 (72.3)	137 (76.1)
Not married	48 (27.7)	43 (23.8)
Educational level^b^		
High school graduate or some high school	30 (17.3)	22 (12.2)
Some college	11 (6.4)	22 (12.2)
Bachelor’s or associate degree	69 (39.9)	71 (39.4)
Graduate school degree	63 (36.4)	65 (36.1)
Individual annual earnings, $^b^		
≤30 000	32 (18.5)	36 (20.0)
30 001-75 000	37 (21.4)	55 (30.6)
>75 000	82 (47.4)	76 (42.2)
Not reported	22 (12.7)	13 (7.2)
Primary payer for delivery hospitalization		
Medicaid	40 (23.1)	35 (19.4)
Private/other	130 (75.1)	138 (76.7)
Unknown	3 (1.7)	7 (3.9)
Physical health status at time of enrollment^b^		
Good, very good, or excellent	151 (87.3)	161 (89.4)
Fair or poor	22 (12.7)	19 (10.6)
Mental health status at time of enrollment^b^		
Good, very good, or excellent	136 (78.6)	148 (82.2)
Fair or poor	37 (21.4)	32 (17.8)

Abbreviation: PCP, primary care practitioner.

^a^ Prepregnancy body mass index greater than or equal to 30 (calculated as weight in kilograms divided by height in meters squared).

^b^ Self-reported.

^c^ Patients could select other as a race option if they did not self-identify with Asian, Black, or White race. Other included American Indian or Alaska Native and Native Hawaiian or Other Pacific Islander.

Table 2 reports the effects of the intervention on completion of a primary care visit for routine or chronic condition care within 4 months of the patient’s EDD. This primary outcome occurred in 40.0% (95% CI, 33.1%-47.4%) of the intervention group and 22.0% (95% CI, 6.4%-28.8%) of the control group (*P* < .001). When adjusted using linear probability regression models for prespecified randomization strata, the intervention increased the primary outcome by 18.7 (95% CI, 9.1-28.2) pp. The effects on the primary outcome were similar in the sensitivity analyses (Table 3). There were no significant effects on obstetric triage visits or emergency department or urgent care use. However, the intervention group had fewer postpartum readmissions: 1.7% (95% CI, 0.5%-5.1%) vs 5.8% (95% CI, 3.1%-10.4%) (Table 2).

**Table 2.** Effects on Care Use^a^

Outcome	Control group, No. (%)	Intervention group, No. (%)	Adjusted between-group difference, percentage point (95% CI)^b^
Primary outcome			
Completion of a primary care visit for routine or chronic condition care within 4 mo of EDD^c^	38 of 173 (22.0)	72 of 180 (40.0)	18.7 (9.1 to 28.2)
Secondary outcomes of unscheduled care use			
Obstetric triage visit	28 (16.2)	28 (15.6)	−0.1 (−8.2 to 7.2)
Emergency department or urgent care visit	23 (13.3)	20 (11.1)	−1.8 (−8.6 to 5.0)
Postpartum readmission	10 (5.8)	3 (1.7)	−3.9 (−7.8 to −0.1)

Abbreviations: EDD, estimated due date; PCP, primary care practitioner.

^a^ More descriptive definitions for all outcomes are included in eTable 2 in Supplement 2.

^b^ To account for randomization strata, regressions include indicator variables for whether or not the participant had any PCP visit in the 3 years before randomization and whether the participant received prenatal care from a hospital or health center.

^c^ *P* < .001.

**Table 3.** Sensitivity Analyses for the Primary Outcome

Sensitivity analysis	Control group, No. (%)	Intervention group, No. (%)	Adjusted between-group difference, percentage point (95% CI)^a^
Self-reported PCP visit completion within 4 mo of delivery	46 of 113 (40.7)	65 of 105 (61.9)	20.1 (7.0-33.3)
Primary outcome restricted to visits with the patient’s designated PCP	23 of 173 (13.3)	50 of 180 (27.8)	15.1 (6.7-23.5)
Primary outcome restricted to patients with PCP in the same health system	30 of 123 (24.4)	58 of 118 (49.2)	24.6 (12.5-36.6)
Any primary care visit within 4 mo of EDD	54 of 173 (31.2)	82 of 180 (45.6)	15.1 (5.0-25.3)
Any primary care visit or scheduled visit within 1 y of EDD	75 of 173 (43.4)	117 of 180 (65.0)	24.0 (14.2-33.8)

Abbreviations: EDD, estimated due date; PCP, primary care practitioner.

^a^ To account for randomization strata, regressions include indicator variables for whether or not the participant had any PCP visit in the 3 years before randomization and whether the participant received prenatal care from a hospital or health center.

**Figure 2.**
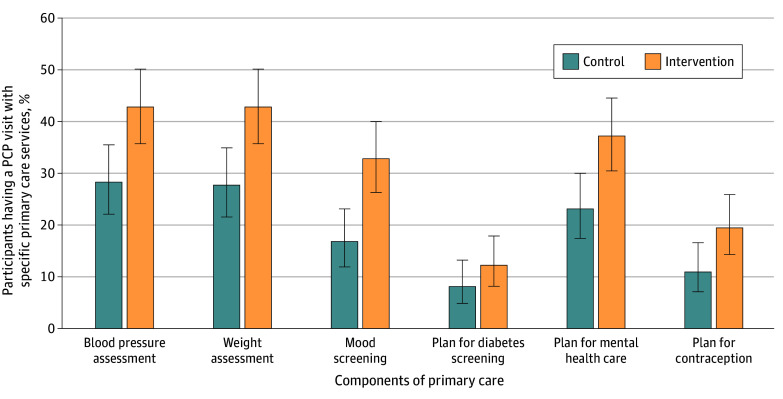
Effects on Content of Care Received by a Primary Care Practitioner (PCP) Outcomes are not contingent on having a primary care visit. Error bars indicate 95% CI.

Figure 2 compares the secondary outcomes related to the content or provision of care between the 2 groups. Intervention group participants had a higher likelihood of having a PCP visit with a weight screening (42.8%; 95% CI, 35.7%-50.1% vs 27.7%; 95% CI, 21.6%-34.9%), blood pressure screening (42.8%; 95% CI, 35.7%-50.1% vs 28.3%; 95% CI, 22.1%-35.1%), and mood screening (32.8%; 95% CI, 26.3%-40.0% vs 16.8%; 95% CI, 11.9%-23.1%). Intervention group participants were also more likely to have a PCP visit with a plan documented about their mental health (37.2%; 95% CI, 30.5%-44.5%) vs 23.1%; 95% CI, 17.4%-30.0%) and with a documented contraception plan (19.4%; 95% CI, 14.3%-25.9% vs 11.0%; 95% CI, 7.1%-16.6%). There was no significant difference in a documented plan for diabetes screening between the 2 groups. Comparisons of the secondary outcomes related to the content of care among subgroups of health conditions are reported in eTable 4 in Supplement 2; many comparisons were limited by small sample sizes.

There was treatment effect heterogeneity across health conditions, demographic characteristics, and baseline self-reported physical and mental health status (eTable 5 in Supplement 2). While the study was not powered to detect outcomes within subgroups, the intervention was associated with increases in PCP visits among nearly all subgroups examined.

## Discussion

Among pregnant people with common comorbidities, a behavioral economics–informed intervention bundle, including default appointment scheduling, tailored messaging, and nudge reminders, increased PCP visit completion within 4 months post partum by 18.7 pp, a nearly 2-fold increase. The primary finding was robust to multiple definitions or variations of the primary outcome, including self-reported PCP visit attendance. The effects on the primary outcome appeared largely consistent among population subgroups, although small sample sizes limited power in these comparisons. Not only did the intervention increase PCP visit completion, it also resulted in more individuals receiving important screening tests and services. There were no observed changes in emergent or urgent care visits between the 2 groups. However, any potential effects of facilitated primary care engagement on emergent care use are more likely to occur later in the postpartum year or beyond, and we intend to measure longer-term care use and outcomes in future studies.

Our results suggest that behavioral economic–informed interventions that reduce patient administrative burden have the potential to be relatively low-resource, high-impact approaches to increasing primary care use, a critical priority in the context of decreasing and inequitable primary care engagement in the US.^35,36^ Behavioral economics research examines how people make predictable decision errors and tests interventions that leverage these insights to remove behavioral barriers (nudges).^37,38,39,40,41,42,43,44,45,46,47^ These interventions often try to make it easier for people to make choices they already want to undertake but do not. In kind, the underlying hypothesis of the present study was that many postpartum individuals with or at high risk for chronic conditions who have a PCP assigned want to receive care by their PCP but face multiple barriers to primary care engagement in the postpartum period, including identifying who their PCP is and scheduling with them. Our study design was built to address 2 common behavioral barriers, namely, inattention and status quo bias, and demonstrated how default primary care appointment scheduling—a salient label for the appointment—and tailored SMS messages and appointment reminders can increase postpartum primary care engagement. Similar approaches have motivated other health behaviors, including in obstetric and postpartum care.^47,48,49,50,51^

This study builds on prior efforts to improve postpartum health and well-being.^33,52,53,54,55,56,57,58^ Our study is most closely aligned with the intervention research on postpartum care navigation in which patient navigators identify and holistically address patient-level barriers to care and assist with care coordination.^52,59^ Although obstetric care navigators hold great promise for improving postpartum health care use, that level of intervention intensity and cost may not be necessary for most postpartum people needing primary care. Results from this study suggest that reducing some patient administrative barriers may be a relatively resource conscious but highly effective approach to encouraging postpartum primary care transitions. Specifically, we demonstrated this intervention could be delivered consistently, with the successful scheduling of an annual visit appointment for 76.1% of participants and a low no-show appointment rate of only 4.4%. Future work should focus on examining the health impacts and cost-effectiveness of the intervention.

### Limitations

The study had several limitations. First, the study tested a bundled intervention; we were unable to measure the effectiveness of the individual components for increasing PCP visits. Next, we observed health care encounters within a single health system, although the health system is large (>1300 PCPs). This study was conducted in Massachusetts, in which pregnancy-related Medicaid coverage extends for 12 months post partum and may impact the generalizability of our findings. We could not observe PCP visits for clinicians who do not use or are not affiliated with the health system’s common EHR. As an alternate measure, we examined self-reports of PCP visits, which was highly consistent with results using EHR data. However, the response rate of 61.8% (balanced across treatment and control groups) may also limit the generalizability of self-reported outcomes. This study focused on individuals who had an identified PCP at enrollment; given the limited availability of PCPs in certain areas, the effect of the intervention may be lessened for individuals seeking to establish care with a new PCP. In addition, the study was not powered to detect differences in many secondary outcomes related to the content of primary care within health conditions, and larger studies are needed to ascertain the impact of the intervention on the quality of primary care for specific conditions.

## Conclusions

In this randomized clinical trial, a behavioral economics–informed intervention to improve postpartum transitions to primary care substantially increased postpartum primary care visit completion for patients with or at risk for common comorbidities. Targeting this population at a time of high health activation and motivation, this intervention represents a potentially scalable approach to increasing primary care engagement and ongoing health condition management in the postpartum months and beyond. Ongoing follow-up related to this study seeks to analyze condition-specific management (ie, the content and quality of care provided in the postpartum period) and long-term health outcomes. Similarly, as many individuals still did not attend a PCP appointment within 4 months even with the assistance of this intervention, additional investigations should focus on identifying and addressing remaining barriers to transitioning to primary care after pregnancy.

## Notes

Supplement 1.
Study Protocol

Supplement 2. **eFigure.** Examples of Study Messages and Reminders **eTable 1.** Outcome Definition Table **eTable 2.** Comparison of the Intervention Components Between Groups **eTable 3.** Reasons the Primary Care Appointment Could Not Be Scheduled **eTable 4.** Secondary Outcomes by Health Condition Subgroups **eTable 5.** Primary Outcome by Population Subgroups (Heterogeneity Analysis)

Supplement 3.
Data Sharing Statement
