# Supplementary material for: Postpartum Primary Care Engagement Using Default Scheduling and Tailored Messaging: A Randomized Clinical Trial
Source: JAMA Netw Open. 2024 Jul 16;7(7):e2422500. doi: 10.1001/jamanetworkopen.2024.22500 (PMC11252898; doi:10.1001/jamanetworkopen.2024.22500)
Supplement: Supplement 1. — Study Protocol [file jamanetwopen-e2422500-s001.pdf]

Study Protocol and Amendments

Postpartum Primary Care Engagement using Default Scheduling and Tailored Messaging: *A Randomized Clinical Trial*
PIs: Mark Clapp, MD MPH; Jessica Cohen, PhD

Institutional Review Board: Mass General Brigham (MGB)
Original IRB protocol approved: 7/26/2022
Date of first patient enrollment: 11/3/2022
Date of last patient enrollment: 4/24/2023

The initial IRB protocol and approved updates to the original protocol are included below. Staff and funding IRB amendments are not included.

**Number**	**Submitted**	**Approved**	**Summary of Changes**
AME1	9/8/22	9/8/22	Staff amendment
**AME2**	9/13/22	9/14/22	The following updates were made:<br>1) minor adjustments to the enrollment criteria<br>2) minor updates to the language in the patient messages<br>3) added additional component to intervention arm (study team messages PCP that their patient is recently postpartum and an appointment has been scheduled for them)<br>4) added explicit language on the possibility of PCP visit cost to study messages and study fact sheet at request of project funder<br>5) minor sample size adjustments after more accurate estimates on potential eligible patients<br>6) study schema changed to reflect accurate outcome assessment time point
**AME3**	10/6/22	10/7/22	Updated baseline survey after initial piloting
**AME4**	10/12/22	10/14/22	Added Spanish versions of the IRB-approved documents (study invitation letter, patient messages, study fact sheet)<br>Added clinicaltrials.gov registration number
AME5	10/17/22	10/26/22	Single IRB (sIRB) set-up
**AME6**	10/26/22	10/28/22	Added Spanish translation of baseline survey<br>Updated patient messages
AME7	10/28/22	11/2/22	Added sIRB child site: Harvard School of Public Health
**AME8**	12/1/22	12/2/22	Amended the study protocol to add clarity and ensure consistency in the language of eligibility criteria related to due date (not delivery date)
**AME9**	1/26/23	2/2/23	Added the finalized version of the endline survey for participants enrolled in the study
AME10	2/9/23	2/22/23	Updated project sponsor fund number
AME11	3/12/23	3/12/23	Staff amendment
**AME12**	3/17/23	3/24/23	After piloting, the endline survey questions were modified.<br>Renumeration was decreased from $25 to $20 after finding the survey could be completed in 5-10 minutes rather than 10-20 minutes
AME13	4/1/23	4/2/23	Staff amendment
**AME14**	4/2/23	4/3/23	Added the endline survey materials translated into Spanish with certification of translation
**AME15**	4/25/23	5/4/23	Updated study status: study enrollment closed<br>Edited SMS endline survey messages to include the link to the REDCap survey link, which was sent via email
AME16	5/2/23	5/31/23	Staff amendment
AME17	9/12/23	9/12/23	Staff amendment
AME18	9/20/23	9/21/23	Staff amendment
AME19	9/25/23	10/11/23	Added additional funding source

Title: Bridging the Gap from Postpartum to Primary Care: A Behavioral Science Informed Intervention to Improve Chronic Disease Management among Postpartum Women (the Bridge Study)

Sponsor Name:

PI Name: Clapp, Mark A **Protocol #:** 2022P001723 **Type:** Initial Review (IR)

Date Received: July 09, 2022

Study Staff Added

Name	Role	Degree	Organization	Training
Clapp, Mark	Principal Investigator	MD, MPH	MGH > OB/GYN Service	08/24/22
Cohen, Jessica	Co-Investigator			
James, Kaitlyn	Data Coordinator/Manager	Ph.D, MPH	MGH > OB/GYN Service	09/13/22
Ray, Alaka	Co-Investigator	MD	MGH > Medical Services	02/06/24
Siegel, Molly	Co-Investigator	MD	MGH > OB/GYN Service	08/13/23
Warsame, Fowsia	Research Assistant		BWH > Medicine > Endocrine	11/10/21

Non Study Staff Added

Name	Degree	Organization
Murphy, Jamie	BS, MBA	MGH > OB/GYN Service

Funding source

Record #	Fund	Project Period	PI Name	Sponsor	Record Type	Process	Link Date	Link Status
2022A001786	241846	06/01/22-05/31/23	Clapp, Mark A	MASSACHUSETTS INSTITUTE OF TECHNOLOGY	RM – Funded Agreement	IR		Pending
2021A018645	241621	06/01/22-05/31/23	Clapp, Mark A	National Bureau of Economic Research (NBER)	RM – Funded Agreement	IR		Pending

Signatures

PI Name: Clapp, Mark A, MD, MPH

Authenticated: July 05, 2022

Department Chair: Ecker, Jeffrey L, MD

Authenticated: July 09, 2022

InitialReview

Title:

Bridging the Gap from Postpartum to Primary Care: A Behavioral Science Informed Intervention to Improve Chronic Disease Management among Postpartum Women (the Bridge Study)

The Mass General Brigham Institutional Review Board has created several forms for review of human subjects research. This questionnaire includes a series of questions to identify the form (s) you need to complete for your research project.

1. Intervention/Interaction
2. Health / Medical Information
3. Excess Human Material and Related Health / Medical Information
4. Secondary Use of Research Samples and/or Data (samples/data from another research study)
5. Research Data Repository (collecting and storing health/medical information for future research)
6. Tissue or Sample Repository
7. Coordinating Center / Core Labs
8. Emergency / Single Patient Use of Investigational Products

---

1. Intervention and/or Interaction

Does your research involve an **intervention** and/or **interaction** with subjects for the collection of specimens or biological material or data (including health or clinical data, surveys, focus groups or observation or behavior)?

NOTE: Do not answer YES if this protocol is to establish a Research Data Repository or Sample/Tissue Repository. There are separate forms for Data and Tissue Repositories.

◉ Yes
◯ No

Will the study population include **children**?

◯ Yes
◉ No

Will the study population include **adults with impaired decision-making capacity** for whom permission for participation will be obtained from their legally authorized representative (surrogate consent)?

◯ Yes
◉ No

Will the study population include **neonates of uncertain viability** and/or **nonviable neonates**?

◯ Yes
◉ No

Will the study population include **pregnant women** and/or **fetuses**?

- [x] Yes
- [ ] No

Will the study population include any of the following:

- Patients who are in the hospital at any time during the study **(inpatients)**
- Patients seen in the **Emergency Department**
- **Patient Care Services Staff,** e.g. nursing staff asked to complete surveys on patient care or nursing practice

- [ ] Yes
- [x] No

Will you be doing either of the following:

- **Testing a drug, biologic, dietary supplement or other agent** for safety and/or efficacy
- **Administering a drug, biologic, dietary supplement or other agent** to study human physiology

- [ ] Yes
- [x] No

Will you be doing any of the following:

- **Testing a medical device** for safety and/or efficacy
- **Using a marketed device** in a non-standard (off-label) way
- **Using a commercially available device** that is not approved for use in humans

- [ ] Yes
- [x] No

Will the study involve **ionizing radiation**, e.g. x-ray, radioactive drug, fluoroscopy?

- [ ] Yes
- [x] No

Will the study involve **non-ionizing radiation**, e.g. MRI, ultrasound, laser, ultraviolet light emitting device or microwave?

- [ ] Yes
- [x] No

COVID-19/SARS-CoV-2

Is this research related in any way to the novel coronavirus, COVID-19, SARS-CoV-2 or, any impact or

consequences related to the outbreak and pandemic?

○ Yes
◉ No

Sponsor Funding: MASSACHUSETTS INSTITUTE OF TECHNOLOGY [Non-Profit]

Select the source of funding that will be used to support the proposed research:

◉ Government / Foundation / Other Non-Profit
○ Corporate
○ Institutional Award
○ Department Funds
○ None

Indicate application type:

◉ Grant / Contract (direct award to an Institution)
○ Subcontract (from another Institution)

Indicate the applicant institution:

○ BWH
◉ MGH
○ SRH
○ McLean
○ Faulkner
○ Broad Institute
○ Other

Enter Principal Investigator name (if different):

Enter title of proposal (if different):

Bridging the Gap to Primary Care: A Behavioral Science Informed Intervention to Improve Chronic Disease Management among Postpartum Women

Enter grant number (if known):

Example of NIH grant number:

Insight Agreement Proposal Number (read-only field):

2022A001786

Has the project been awarded at the time of this submission?

○ Yes
◉ No

Explain:

Under review

For NIH-sponsored cooperative group multi-center trials: The IRB requires a copy of the cooperative group protocol and sample informed consent documents be submitted for review and comparison with the documents submitted for local IRB review.
For guidance, refer to, "IRB Review of Applications for HHS Support."

Medicare Coverage Analysis Requirement

Does the protocol for this study involve any items or services that will be billed to Medicare/private insurance, including study-specific procedures or those considered usual and customary care ("standard of care") outside the trial context?

- ○ Yes
- ◉ No

NOTE: If you are unsure how to answer this question, please contact Sarah Bednar at Mass General Brigham Clinical Trials Office at 617-954-9364, or for NWH investigators, please contact Jayita Sen at 617-243-6517 for more information.

Is this the primary source of funding?

- ○ Yes
- ◉ No
- ○ Not applicable

Sponsor Funding: National Bureau of Economic Research (NBER) [Non-Profit]

Select the source of funding that will be used to support the proposed research:

- ◉ Government / Foundation / Other Non-Profit
- ○ Corporate
- ○ Institutional Award
- ○ Department Funds
- ○ None

Indicate application type:

- ◉ Grant / Contract (direct award to an Institution)
- ○ Subcontract (from another Institution)

Indicate the applicant institution:

- ○ BWH
- ◉ MGH
- ○ SRH
- ○ McLean
- ○ Faulkner
- ○ Broad Institute
- ○ Other

Enter Principal Investigator name (if different):

Enter title of proposal (if different):

Bridging the Gap to Primary Care: A Behavioral Science Informed Intervention to Improve Chronic Disease Management among Postpartum Women

Enter grant number (if known):

Example of NIH grant number:

Insight Agreement Proposal Number (read-only field):

2021A018645

Has the project been awarded at the time of this submission?

- ◯ Yes
- ◉ No

Explain:

Under review

For NIH-sponsored cooperative group multi-center trials: The IRB requires a copy of the cooperative group protocol and sample informed consent documents be submitted for review and comparison with the documents submitted for local IRB review.
For guidance, refer to, "IRB Review of Applications for HHS Support."

Medicare Coverage Analysis Requirement

Does the protocol for this study involve any items or services that will be billed to Medicare/private insurance, including study-specific procedures or those considered usual and customary care ("standard of care") outside the trial context?

- ◯ Yes
- ◉ No

NOTE: If you are unsure how to answer this question, please contact Sarah Bednar at Mass General Brigham Clinical Trials Office at 617-954-9364, or for NWH investigators, please contact Jayita Sen at 617-243-6517 for more information.

Is this the primary source of funding?

- ◉ Yes
- ◯ No
- ◯ Not applicable

Will the funding cover all subject study-related drugs, devices, procedures, tests, and visits?

- ◯ Yes
- ◯ No
- ◉ Not applicable (no subject study-related costs)

Digital Health

*Please consider **ALL** applications, systems or technologies being used in your study when completing these questions. Applications, systems and technologies include:*

- Touchable or wearable devices collecting physiological and/or health data
- Apps on mobile phones, tablets or iPads
- Websites where people answer surveys/questions
- Externally hosted data collection tools, such as Survey Monkey or Qualtrics
- Interactive social media websites where people enter health data or connect with healthcare workers, researchers, or other research participants
- Communications/outreach to participants performed by external vendors

For guidance on technologies, please refer to:

- **Guidance on Research Using the Internet - Survey Research Using Web-Based Survey Tools**
- **Guidance on Research Using the Internet - Informed Consent in Online Research**
- **Research Computing Information Security (RISO) Website**
- **Research Information Security Review for IRB Applications**
- **Email: riso@partners.org**

NOTE: Not all IRB applications require an information security risk assessment. Also, the Research Information Security (RISO) does not get triggered to conduct a data security review/assessment at the same time the initial IRB application is submitted. The average timeline for review is 2-6 weeks from the time that RISO receives the request and the ServiceNow risk assessment form is submitted. To open up a ServiceNow ticket, click here. If there are any pressing timelines associated with starting the study, please contact riso@partners.org directly.

DATA COLLECTION, STORAGE AND SECURITY

Collection of Protected Health Information (PHI) or Sensitive Information

Will you be using applications, systems or technologies outside Mass General Brigham to collect, share or store protected health information (PHI) and/or sensitive information?

○ Yes
◉ No

Protected health information (PHI) includes the following:

- **Names, including initials**
- **Social security numbers**
- **Medical record numbers**
- **Addresses by street location**
- **Addresses by city, county, precinct, zip code**
- **All elements of dates (except year) related directly to individuals including, but not limited to, dates of birth, death, admission, discharge, or any service**
- **All ages over 89 and all elements of dates (including year) indicative of such age**
- **Telephone numbers**
- **FAX numbers**
- **Electronic email addresses**
- **Web URLs**

- **Internet protocol (IP) addresses**
- **Account numbers**
- **Certificate/license numbers**
- **Vehicle identification numbers and serial numbers including license plates**
- **Medical device identifiers and serial numbers**
- **Biometric identifiers, including finger and voice prints**
- **Full face photographs and any other comparable images**
- **Any other unique identifying numbers, characteristics or codes including, but not limited to, globally unique identifiers (GUID) and universally unique identifiers (UUID) or equivalent**

Digital Health Technology

Will you be using mobile and wireless devices, wearable devices, smartphone apps, digital health tools, health-related IT, new healthcare software and related new technologies to generate, use and/or disseminate health information or physiological data? Note: Wearable devices can include activity trackers (i.e., FitBit), free-standing monitors or sensors worn on body which connect through wireless, Bluetooth or other method to passively collect data.

- ○ Yes
- ◉ No

Are any of the technologies developed in-house by Mass General Brigham employees?

- ○ Yes
- ◉ No

Will any of the digital health technologies communicate with and/or be integrated with any clinical systems (e.g., EPIC, EMRs, etc.)?

- ○ Yes
- ◉ No

Social Media Data Collection Tools

Will you be using social media tools to **collect** data for this study?

- ○ Yes
- ◉ No

NOTE: This question does not include the use of social media to post advertisements and contact information for the study. This question is specifically asking about the collection of data using social media tools. Social media tools can include Facebook, Twitter, chat/text messages, message boards, interactive web pages and blogs.

Web-Based Data Collection Tools

Will you be using web-based survey or data collection tools to administer a survey or questionnaire?

- ◉ Yes
- ○ No

Indicate your collection methods (check all that apply):

- ☑ Survey / Data collection tool hosted inside Mass General Brigham firewall

  Indicate:
  - ☑ REDCap or StudyTRAX through Mass General Brigham
  - ☐ Other survey / data collection tool
- ☐ Survey / Data collection tool hosted outside Mass General Brigham firewall

---

Non-US Data Collection and/or Storage

Will you be collecting and/or storing data in countries outside of the United States?

- ○ Yes
- ◉ No

Data Storage

Describe data storage (physical location, security controls) and data transmission if data is sent/received to/from multiple sites/collaborators, etc.

The primary storage device for study information will be the system-issued, password-protected, encrypted desktop computer (Clapp) located behind the MGB firewall in his physical, locked office on a secure administrative floor (Austen 438). Online data collection tools will use REDCap software. Data shared with outside collaborators (Cohen) will be deidentified and not include any personal information or patient-level identifiers.

Will your research involve the use of any cloud-based technology (i.e., Amazon Web Services, Azure, Google Cloud, etc.)?

- ○ Yes
- ◉ No

Will your research involve the use of any removable media for data storage (i.e., external hard drive, USB flash drive, etc.)?

- ○ Yes
- ◉ No

Note: Mass General Brigham policy requires that all devices used to store data be encrypted.

Data Sharing

Will any data sharing take place with external non-Mass General Brigham collaborators?

- ◉ Yes
- ○ No

Check all that apply:

- ☑ MGB DropBox for Business

- ☐ MGB OneDrive for Business
- ☐ MGB Secure File Transfer
- ☐ MGB Syncplicity
- ☐ Other

STUDY COMMUNICATIONS: Email Distribution Lists / Text Messaging

Will you be using an external third-party vendor to send communications/perform outreach to participants?

- ○ Yes
- ◉ No

Will you be communicating with participants via text message?

- ◉ Yes
- ○ No

Check all that apply:

- ☐ Imprivata Cortext
- ☐ Twilio
- ☑ SMS
- ☐ Other

Conflicts of Interest

---

1. Is this study being funded or supported in whole or in part by an Outside Entity? **NOTE: Support includes providing funds, drug, device, or other resources. Outside Entity means any corporation, foundation or other entity or organization that is not a Mass General Brigham entity including any governmental entity or non-profit.**

- ◉ Yes
- ○ No

a. Name(s) of the Outside Entities

National Bureau Economic Research

Massachusetts Institute of Technology (MIT)

---

2. Are you studying or are you using in any significant way a drug or device or other technology that is owned, developed or licensed by an Outside Entity? **NOTE: Technology that is used in a significant way includes, for example, imaging equipment that, while not the focus of the study, is an important part of the study design and will need to be described and identified in the methods section of a publication. This includes licensed intellectual property such as patents, patent applications, and copyrighted materials, including technology licensed through Mass General Brigham.**

- ○ Yes
- ◉ No

Study Details

Mass General Brigham

Study Classification

The following information is being collected to allow the IRB to provide research administration with reports on the type of clinical research being conducted by Mass General Brigham investigators. This is self-reported information and will not affect what form questions you are presented with later in the application. Please select all that apply.

Type of Study: Select all that apply

Intervention

Content of Study: Select all that apply

Women's Health

Diabetes Mellitus

Prevention

Hypertension

Psychiatry

Will your research be **limited** to **any** of the following:

- Collection of one or more blood samples.
- Prospective collection of biological specimens for research purposes by noninvasive means.
- Collection of data through noninvasive procedures (not involving general anesthesia or sedation) routinely employed in clinical practice.
- Collection of data from voice, video, digital, or image recordings made for research purposes.
- Research on individual or group characteristics or behavior or research employing survey, interview, oral history, focus group, program evaluation, human factors evaluation, or quality assurance methodologies.

◉ No
◯ Yes

NOTE: You must attach a Detailed Protocol on the Attachments page of this submission using the Attachment Type 'Detailed Protocol.' If the study is industry sponsored, the sponsor protocol must be attached as the Detailed Protocol, and a Site Addendum must also be attached using the Attachment Type Site Addendum.

Is this a **randomized controlled clinical trial**?

◯ No
◉ Yes

NOTE:
1. You must attach a schema on the attachments page of this submission using the Attachment Type 'Schema;'
2. You must attach a Detailed Protocol on the Attachments page of this submission using the Attachment Type 'Detailed Protocol.' If the study is industry sponsored, the sponsor protocol must

be attached as the Detailed Protocol, and a Site Addendum must also be attached using the Attachment Type 'Site Addendum.'

Does the research involve the use of **human embryonic stem cells?**

- (●) No
- ( ) Yes

Does the research involve issues related to **women's health**?

- ( ) No
- (●) Yes

Is this a **cancer-related** trial?

- ( ) Yes
- (●) No

NOTE: If this is a cancer-related trial, this study must be withdrawn and submitted to the Dana-Farber/Harvard Cancer Center (DF/HCC) Office for Human Research Studies (OHRS) for review. The DF/HCC conducts centralized protocol review and monitoring for all cancer-related hypothesis-driven clinical research studies undertaken by investigators at DF/HCC institutions. Brigham and Women's Hospital (BWH) and Massachusetts General Hospital (MGH) are DF/HCC institutions. As such the following categories of research must be reviewed by the DFCI IRB, pursuant to the DF/HCC Cancer Center Support Grant:

1. **Cancer-relevant research**
2. **Research funded by NCI, even if not cancer-relevant; or**
3. **Research utilizing DF/HCC infrastructure or DFCI facilities**

The DF/HCC Office for Human Research Studies (OHRS) is the office at DFCI that manages DF/HCC Scientific Review Committee review and DFCI IRB review. For information on how to proceed with your submission, please contact: Dana-Farber Cancer Institute - Office for Human Research Studies (OHRS) at OHRS@dfci.harvard.edu [Attn: Lara Sloboda / Caroline Kokulis]. For detailed information about the above requirement, please review this guidance.

Study Design

Who designed the study?

- ( ) Other
- (●) Mass General Brigham Investigator
- ( ) Corporate Sponsor
- ( ) Cooperative Group / Consortium

NOTE: When the corporate sponsor designs the study, the corporate sponsor's protocol must be submitted to the IRB for review. Before the research can begin, the IRB must approve the sponsor's protocol and Mass General Brigham Clinical Research Office must execute the Agreement with the sponsor.

Will any data generated from this study be submitted to the FDA?

◉ No
◯ Yes

Clinical Trials Registration

Clinical Trials Registration and Results Reporting

Investigator–initiated clinical trials must be registered on ClinicalTrials.gov to comply with federal FDA requirements in FDA 42 CFR 11 (Final Rule) and/or NIH Policy. Studies that falls under both FDA and NIH requirements only need to be registered once. The information posted on ClinicalTrials.gov (CT.gov) must be updated and verified at least every 12 months.

The following information is used to identify studies that require clinical trials registration and results reporting, and to inform Principal Investigators (PI) of their responsibilities for registration and results reporting.

IMPORTANT NOTE: Even if your investigator-initiated clinical trial does not meet the NIH or FDA clinical trials registration requirements, you are strongly advised to read and consider registering your trial to comply with the following additional requirements:

- **A non-federal sponsor may require registration as part of the award's terms and conditions**
- **International Committee of Medical Journal Editors (ICMJE) for publication purposes**
- **Center for Medicare & Medicaid for research billing claims for qualifying clinical trials (Mass General Brigham Clinical Trials Office will notify you if applicable)**
- **Research funders now requiring registration and results reporting: May 18, 2017 Joint Statement**

For additional information, please see the Human Research Affairs Compliance and Education Office website for Clinical Trials Registration.

Is this a Mass General Brigham investigator – initiated research study?

◯ No
◉ Yes

Is this research funded in whole or in part by NIH **AND** does this research meet the NIH's definition of **clinical trial**?

NIH defines a clinical trial as any research study that meets all of the following criteria:

- The study involves human participants;
- The participants are prospectively assigned to an intervention;
- The study is designed to evaluate the effect of the intervention on participants; **AND**
- The effect being evaluated is a health-related, biomedical or behavioral outcome.

◯ No
◉ Yes

The responses to the questions above indicate this study meets the NIH Policy requirements for clinical trials registration and results reporting. Mass General Brigham Institutions have delegated responsibility for clinical trials registration, periodic updates, and results and adverse event reporting to the Principal Investigator ("Responsible Party"). This study must be registered on ClinicalTrials.gov prior to the first subject being enrolled into the study.

Additional information is available from the NIH:

- **NIH's Definition of a Clinical Trial | grants.nih.gov**
- **NIH Definition of Clinical Trial Case Studies | grants.nih.gov**

Responsible Party

Enter Name of MGB investigator who will be the Responsible Party, for example Jane Doe, MD:

Mark Clapp, MD

Enter the full name of the MGB Institution of the MGB investigator, for example, Massachusetts General Hospital:

Massachusetts General Hospital

Documentation of ClinicalTrials.gov Registration

Have you submitted registration information to ClinicalTrials.gov?

◉ No
◯ Yes

Results Reporting

ClinicalTrials.gov results reporting is required within 12 months of the primary endpoint completion date defined as the date that the final subject was examined or received the intervention for the purposes of collection of primary outcome. Early consultation is strongly advised. Please contact the HRA Compliance and Education Office for assistance:

Mass General Brigham Human Research Affairs Compliance and Education Office:
QIProgramCTgovTeam@partners.org

Additional information can be found at:
HRA Compliance and Education Office website for **Clinical Trials Registration**
www.ClinicalTrials.gov

Study Population

Mass General Brigham

How many subjects do you plan to enroll at Mass General Brigham' sites?

360

NOTE: Target enrollment at Mass General Brigham sites is the number of subjects you expect to provide written or verbal consent, or implied consent by voluntary completion of a survey or participation in a focus group.

How many subjects will be enrolled study-wide?

360

What is the age range of eligible subjects who will be enrolled at Mass General Brigham sites?

Enter 'None' if there is no maximum age.

Minimum age:

18

Maximum age:

None

Equitable Selection of Subjects

Will both males and females be enrolled?

○ Yes
◉ No

Which gender will be enrolled?

◉ Females only
○ Males only

Indicate below whether the study population that is being targeted for the research is any of the following groups that require additional protections:

- ☐ Children (less than 18 years of age)
- ☐ Economically or Educationally Disadvantaged
- ☐ Embryos
- ☐ Employees under the direct supervision of the investigators conducting the research
- ☐ Employees (physician, nurses, or other healthcare workers) in the course of, or related to, their employment related duties
- ☐ Individuals with Impaired Decision-Making Capacity
- ☐ Neonates -age up to 28 days
- ☐ Non-English Speakers
- ☐ Patients from the Medical Practice of the Investigator
- ☑ Pregnant Women / Fetuses
- ☐ Prisoners
- ☐ Students of Harvard Medical School
- ☐ U.S. Military Personnel
- ☐ None of the above

Performance Sites / Facilities

Where will investigators covered by this IRB consent/enroll subjects and perform study procedures?

- ☐ BWH
- ☐ Cooley Dickinson Hospital
- ☐ Faulkner
- ☐ MEE
- ☑ MGH

MGH facilities/resources. Check all that apply.

- ☐ Emergency Department
- ☐ Translational and Clinical Research Center
- ☑ Health Centers

Indicate MGH Health Centers:

- ☑ Charlestown Health Center
- ☑ Chelsea Health Center
- ☐ Everett Health Center
- ☑ Revere Health Center
- ☐ Other

- ☐ Inpatient
- ☐ Labor & Delivery / Post Partum Floors
- ☐ NICU
- ☐ Operating Room / Recovery Room
- ☑ Outpatient

Indicate MGH Outpatient Locations:

- ☑ Hospital Main Campus
- ☐ Building 149 Charlestown Navy Yard
- ☐ 1 Bowdoin Square
- ☐ 165 Cambridge Street – Charles River Plaza East
- ☐ 175 Cambridge Street – Charles River Plaza South
- ☐ 185 Cambridge Street - Simches Building
- ☐ 275 Cambridge Street – Professional Office Building
- ☐ Charles River Park (i.e., Emerson, Longfellow, Hawthorne, Whittier)
- ☐ 101 Merrimac Street
- ☐ 151 Merrimac Street
- ☐ 50 Staniford Street
- ☐ 60 Staniford Street
- ☐ Lurie Center - Lexington
- ☐ MGH Back Bay
- ☐ MGH Downtown
- ☑ MGH West - Waltham
- ☑ North Shore Center for Outpatient Care - Danvers
- ☐ Other

- ☐ NSMC
- ☐ NWH

- ☐ PCHI
- ☐ Shriners
- ☐ SRH
- ☐ IHP
- ☐ McLean
- ☐ Broad Institute
- ☐ Wentworth-Douglass Hospital
- ☐ Network Sites (e.g. NeuroNEXT and Stride)
- ☐ Off-site Research (off-site means sites other than those owned or controlled by Mass General Brigham entities)

NOTE: When more than one Mass General Brigham site is included in the submission, designate a Site-Responsible Investigator (not Residents, Fellows, or Trainees) for each site on the Staff & Access page.

Is this a Mass General Brigham investigator-initiated multi-site study?

- ○ Yes
- ◉ No

Recruitment

Source of Subjects

Indicate whether you will use any of the following resources to identify prospective subjects:

- ☐ Census / Public Records
- ☐ Commercial Mailing Lists
- ☐ Emergency Department
- ☐ Inpatient Units
- ☑ Medical Records
- ☑ Outpatient Clinics
- ☐ Primary Physician / Physician Specialist
- ☐ Registries / Patient Databases (e.g. cancer registry)
- ☑ Research Patient Data Registry (RPDR)
- ☐ Research Match (Vanderbilt Recruitment Tool)
- ☐ RSVP for Health
- ☐ Research Studies
- ☐ None of the above

Note: For more information about ResearchMatch, see https://www.researchmatch.org/about/

Methods / Materials

Mass General Brigham

Indicate whether you will use any of the following methods / materials to recruit subjects:

- ☐ Advertisements - E-Mail
- ☐ Advertisements - Internet
- ☐ Advertisements - Rally https://rally.partners.org (Contact rallyforresearch@partners.org for more information)
- ☐ Advertisements - Newspaper (e.g. Metro, Boston Globe)
- ☐ Advertisements - Radio
- ☐ Advertisements - Television
- ☐ Flyers / Postings (e.g. within BWH or MGH)
- ☑ Patient Gateway - Personalized Letters
- ☐ Patient Gateway - Targeted Research Announcements
- ☐ Mail
- ☐ ResearchMatch Volunteer Message (see https://www.researchmatch.org/researchers/)
- ☐ Telephone Calls to Prospective Subjects (who have previously agreed to be contacted, e.g. RSVP for Health)
- ☐ None of the above

NOTES:

1. **The text of all advertisements, letters, and telephone calls/scripts used to recruit subjects must be submitted for IRB approval. You may attach these documents to the application on the Attachments page. For guidance on recruitment and advertising, please see the following MGB IRB web pages Recruitment of Research Subjects and Guidelines for Advertisements for Recruiting Subjects.**
2. **When using Research Match, you must submit the contact message template for Research Match advertisement to the IRB for approval. The contact message template is available to registered researchers on the ResearchMatch website.**

Pre-Screen of Subjects During Recruitment

Will you ask prospective subjects pre-screening questions over the phone to determine eligibility?

- ○ Yes
- ◉ No

Will you ask prospective subjects to complete an online pre-screening tool to determine eligibility?

- ○ Yes
- ◉ No

Note: Refer to Mass General Brigham Guidance on Pre-Screening of Subjects During Recruitment.

Remuneration

Will subjects be paid or receive any type of remuneration / compensation for their time and expenses?

- ◉ Yes
- ○ No

Mass General Brigham

Payment for Participation in Research

Indicate the type and total amount of compensation for completion of the study.

- ☐ Cash
- ☐ Check
- ☑ Gift Certificate

Amount e.g., $50, enter 50.00

20

- ☐ Other

NOTE: Payments to subjects must be made by check if payment involves a one-time payment of greater than $50 OR multiple payments of any amount. See policies Payments to Human Subjects for Participation in Research and Cash Control and Accountability for Payments to Human Subjects for Participation in Research.

Reimbursement for Expenses Related to Participation in Research

If there is not a set amount for meals, parking, and transportation, then estimate or enter the maximum amount budgeted per subject.

- ☐ Meals
- ☐ Parking
- ☐ Transportation
- ☐ Other

Informed Consent

For guidance, refer to the MGB IRB web page Informed Consent of Research Subjects.

Will informed consent and authorization for participation in research be obtained **verbally (oral consent)**, or by use of a **written consent form** approved by the MGB IRB and signed by the subject or the subject's legally authorized representative?

- ◉ Yes
- ○ No

Indicate how informed consent and authorization will be obtained:

- ○ Written
- ◉ Verbal (oral consent)

Indicate who will obtain the informed consent of the subject or the subject's legally authorized representative.

- ☑ Licensed Physician Investigator
- ☑ Non-Physician Investigator
- ☐ Other

Mass General Brigham

Indicate from whom informed consent will be obtained. Check all that apply:

- ☑ Adult Subject
- ☐ Parent(s) / Guardian for Child
- ☐ Court-Appointed Guardian for Adult
- ☐ Surrogate for Adult, other than Guardian (e.g. health care proxy, person with durable power of attorney, spouse, adult child, or close family member).

NOTE: When surrogate consent for adults is obtained, the individuals with impaired decision-making capacity form must be completed. For guidance, refer to the following MGB IRB web pages, Surrogate Consent to Research for Individuals with Impaired Decision-making Capacity

Will the research target a non-English speaking group?

- ○ Yes
- ◉ No

NOTE: When investigators can reasonably expect that more than an incidental number of subjects speaking the same non-English language will be enrolled (for example, if the research is targeting a non-English speaking group), the use of a written translation of the entire English version of the consent form is required. The MGB IRB must approve all written translated versions of the consent from and recommends that the written translation be done by an in-house medical translator from Interpreter Services or other qualified person or service recommended by Interpreter Services. Refer to the MGB IRB guidance on Obtaining And Documenting Informed Consent Of Subjects Who Do Not Speak English.

Will a study subject advocate participate in the consent process?

- ○ Yes
- ◉ No

NOTE: A study subject advocate may be used, for example, because subjects have limited time to consider participation in a study involving significant risk, or may feel obligated to participate.

Will subjects have less than 12 hours to decide whether or not to participate?

- ○ Yes
- ◉ No

NOTE: The IRB may waive the requirement to obtain a signed written consent / authorization form if it finds either: (1) the only record linking the subject and the research is the consent form and the principal risk would be potential harm resulting from a breach in confidentiality; or (2) the research presents no more than minimal risk of harm to subjects and involves no procedures for which written consent is normally required outside the research context.

Diaries

Will subjects be asked to complete a diary(ies)? For example, a drug diary, pain diary, symptom diary, etc.

- ○ Yes
- ◉ No

Privacy / Confidentiality

Health/Medical Records

Will medical history / clinical information be obtained from the subject's health / medical record for study purposes?

- (●) Yes
- ( ) No

Explain what information will be abstracted from health / medical records:

Age

Race

Ethnicity

Primary language

Primary insurer

Address

Pregnancy information

Primary care provider/address

Number of PCP visits prior to start of pregnancy

Medical history, including diabetes, hypertension, mood/anxiety disorders

Prenatal clinic

Prenatal care provider

Number of prenatal care visits

Pregnancy-related medical conditions, including gestational diabetes and pregnancy-induced hypertensive disorders

Height / weight / BMI

Delivery information, including delivery-related complications

Number of postpartum care visits

Primary care provider encounters before and after delivery

Urgent / unscheduled visits before and after delivery

Will sensitive personal health information resulting from this study become part of the subject's

health/medical record?

◯ Yes
◉ No

Sensitive personal information obtained for research purposes typically does not become part of the subject's health/medical record. Rather, these data are retained in the research records only.

NOTE: Certain categories of medical information are commonly recognized as potentially sensitive, such as information related to mental health; sexual behaviors or sexual orientation; illegal behaviors; and substance abuse. Some genetic test results may be viewed similarly. When applicable, investigators are reminded to inform subjects in the consent form that sensitive personal health information may become part of their health/medical record, e.g., routine testing for HIV infection.

Will you enroll subjects in the study in Epic?

◉ Yes
◯ No

Do you wish to receive notifications when subjects enrolled in the study are admitted to an Emergency Department or admitted for a day procedure or inpatient stay at a hospital that uses the Mass General Brigham version of Epic?

◉ Yes
◯ No

NOTE: If you wish to receive notifications about the death of subjects enrolled in your study, submit a ticket to the Research-DHeC.

Who do you want to receive the notifications?

◯ PI Only
◉ All Study Staff

NOTE: Epic is both the Mass General Brigham electronic medical record and the billing system, including research billing. For more information about Epic/eCare, refer to Research Information Services & Computing's Support & Training page eCare Research FAQs.

Do you consider this to be a sensitive study that requires the title of the study be masked in the electronic medical record (eCare)?

◯ Yes
◉ No

NOTE: The title of studies determined to be sensitive will be masked in the Mass General Brigham eCare system (e.g., Psychiatry Study 1 will appear as MGB 2017P001234). The IRB is responsible for making the final determination whether a study is sensitive. Examples of studies that may be considered sensitive are studies that collect information about:

Mass General Brigham

- **sexual practice**
- **sexual victimization**
- **illegal behaviors**
- **alcohol, drugs, or other addictive products**
- **stigmatizing illnesses**

Certificate of Confidentiality

NIH funded research that collects or uses ***identifiable sensitive information*** is automatically issued a Certificate of Confidentiality as part of the terms and conditions of the award. The NIH considers research that is reviewed by the IRB to involve identifiable sensitive information requiring a Certificate.

If you do **NOT** have NIH funding, do you have or do you plan to obtain a Certificate of Confidentiality to protect the data from disclosure (e.g., by court order or subpoena)?

○ Yes
◉ No

NOTE: For information on Certificates of Confidentiality, refer to the OHRP website.

Sending Health Information to Research Collaborators Outside Mass General Brigham

Will identifiable information be shared with research collaborators **outside** Mass General Brigham?

○ Yes
◉ No

NOTE: Data that includes any of the identifiers listed below are considered identifiable.

- **Names, including initials**
- **Social security numbers**
- **Medical record numbers**
- **Addresses by street location**
- **Addresses by city, county, precinct, zip code**
- **All elements of dates (except year) related directly to individuals including, but not limited to, dates of birth, death, admission, discharge, or any service**
- **All ages over 89 and all elements of dates (including year) indicative of such age**
- **Telephone numbers**
- **FAX numbers**
- **Electronic email addresses**
- **Web URLs**
- **Internet protocol (IP) addresses**
- **Account numbers**
- **Certificate/license numbers**
- **Vehicle identification numbers and serial numbers including license plates**
- **Medical device identifiers and serial numbers**

- **Biometric identifiers, including finger and voice prints**
- **Full face photographs and any other comparable images**
- **Any other unique identifying numbers, characteristics or codes including, but not limited to, globally unique identifiers (GUID) and universally unique identifiers (UUID) or equivalent**

For guidance, refer to the MGB IRB web page on the HIPAA Privacy Rule.

NOTE: Non-institutional email services are not approved to transmit confidential data. Use "Send Secure" to transmit confidential data to non-institutional email domains (e.g., gmail, yahoo, icloud, etc.). For more information on appropriate methods of transmitting research information/data to research collaborators, refer to the Research Information & Computing website.

Business Associates

Will identifiable information be shared with persons or entities outside of Mass General Brigham who are not research collaborators or who are not part of the research team, but who will perform some aspect of the research on behalf of Mass General Brigham, e.g., a private recruitment agency, outside laboratory or data analysis group?

○ Yes
◉ No

NOTE: In this situation, the persons or entities referred to above are considered business associates of Mass General Brigham, therefore a Business Associate Agreement is required.

Instruments / Questionnaires

Will the research involve the development of instruments, questionnaires, surveys, interviews, and/or focus group topics?

○ Yes
◉ No

Will the research involve the use of instruments, questionnaires, surveys, interviews, and/or focus group topics?

◉ Yes
○ No

List of Instruments / Questionnaires / Surveys / Interviews / Focus Group Topics

Enter the name of each of the instruments, questionnaires, surveys, interviews and/or focus group topics. Upload each of the instruments, questionnaires, surveys, interviews and/or focus group topics to the Attachments page. **Do not list any that are under development.**

Mass General Brigham

Baseline Survey

Ancillary Drugs

Will you be using FDA-approved drugs for research-related ancillary/supportive care?

- ◯ Yes
- ◉ No

NOTE: An ancillary drug is a commercially available drug that is being used for a research-related ancillary test or for supportive care (e.g. methacholine challenge tests, lidocaine for research-related biopsies, EMLA cream for venipuncture, contrast agent for scans or x-rays). The Research Consent Form should include the risks of ancillary drugs being administered as part of the research.

Ancillary Devices

Will any non-hospital inventory FDA-approved medical devices be used to obtain measurements, collect data, or monitor subjects?

- ◯ Yes
- ◉ No

NOTE: When the sponsor (or another party) provides a medical device for use to obtain measurements, collect data, or monitor subjects, the investigator must request a zero dollar purchase order to track receipt of the medical device and to document BME inspection for electrical safety, when necessary. For more information, refer to the Zero Dollar Purchase Order Policy.

Non-Intervention / Non-Interaction Group

In addition to the intervention/interaction group that provides informed consent to participate in the research, do you plan to also review the health/medical records or other private/confidential information of any subjects **with whom you will have no contact at any time**?

- ◉ Yes
- ◯ No

Source of Health / Medical Information

Check all that apply:

- ☐ BWH
- ☐ Faulkner
- ☑ MGH
- ☐ NWH
- ☐ NSMC
- ☐ PCHI
- ☐ SRH
- ☐ Shriners
- ☐ McLean
- ☐ MEE
- ☐ Other

Data To Be Collected / Obtained

Check all that apply:

Administrative:

- ☐ Billing Data
- ☑ Coded encounter data (diagnoses, procedures, dates)
- ☑ Demographic data (age, gender, vital status)
- ☑ Personal data (name, address, PCP)

Health / Medical:

- ☑ Allergies
- ☑ Discharge Summary
- ☐ Doctors Orders
- ☐ History / Physical
- ☐ Immunizations
- ☑ Medication List
- ☑ Office / Clinic Notes
- ☐ Operative / Procedure Notes (e.g. endoscopy)
- ☑ Pharmacy
- ☑ Problem List

Health/Medical Reports/Results

- ☑ Blood Bank
- ☑ Laboratory
- ☑ Pathology
- ☑ Radiology

Sensitive/Personal Information:

- ☑ HIV Status
- ☑ Mental Health
- ☑ Reproductive History (e.g., abortions)
- ☑ Sexual Behavior/Sexually Transmitted Diseases
- ☐ Substance Abuse (e.g., drug or alcohol abuse)
- ☐ Other potentially stigmatizing behaviors
- ☐ **Other Information**

Have you defined data variables to be collected/obtained?

- ◉ Yes
- ◯ No

NOTE: You must attach a document that lists all data variables to be collected / obtained in the Attachments section before submission.

Mass General Brigham

Data To Be Requested From The Following Time Period

Indicate the time period over which the health / medical information was / will be created as part of clinical care.

From (mm/yyyy):

07/2022

To (mm/yyyy): (For future data, use anticipated project end date)

12/2023

NOTE: This information is needed for the MGB IRB to determine whether the research use of the health/medical information meets the criteria for an exemption from the requirements for IRB review. For more information about HUMAN SUBJECTS RESEARCH or EXEMPT RESEARCH, see the OHRP website.

Protected (Identifiable) Health Information

PHI refers to health/medical information that is accompanied by any of the listed 18 HIPAA identifiers or by a code (where the key to the code is accessible to investigators) that links to the identifiers. DE-IDENTIFIED DATA (without any identifiers or codes that link back to individuals) are not considered PHI, and are not subject to HIPAA regulations.

Will you be recording any of the identifiers listed below with the data or using a code to link the data to any of the identifiers?

- Name, including initials
- Social Security Number (SSN)
- Medical record number
- Address by street location
- Address by city, county, precinct, zip code
- All elements of dates (except year) related directly to individuals including, but not limited to dates of birth, death, admission, discharge, or any service
- All ages over 89 and all elements of dates (including year) indicative of such age
- Fax number
- Electronic email address
- Web URL
- Internet provider (IP) address
- Health plan beneficiary number
- Account number
- Certificate / license number
- Vehicle identification number and serial number
- Biometric identifiers, including finger and voice print
- Full face photographic images and any other comparable images
- Any other unique identifying number, characteristic or code, including, but not limited to, globally unique identifier (GUID) and universally unique identifier (UUID), or equivalent identifier

NOTE: If you are recording medical record number or other identifiers, even if temporarily for QA purposes or to avoid duplicating records, then answer "Yes".

◉ Yes

◯ No

Check the identifiers that will be recorded with or linked by code to the data.

- ☐ Name
- ☐ Social Security Number
- ☑ Medical record number
- ☐ Address by street location
- ☐ Address by town / city / zip code
- ☑ Dates (except year), e.g., date of birth; admission / discharge date; date of procedure; date of death
- ☐ Telephone number
- ☐ Fax number
- ☐ Electronic email address
- ☐ Web URLs
- ☐ Internet protocol (IP) address
- ☐ Health plan beneficiary number
- ☐ Account number
- ☐ Certificate / license number
- ☐ Vehicle identification number and serial number, including license plate number
- ☐ Medical device identifiers and serial numbers
- ☐ Biometric identifiers (finger and voice prints)
- ☐ Full face photographic image
- ☐ Any other identifier; or combination of identifiers likely to identify the subject (e.g., Pathology Accession #)

Explain why it would be impossible to conduct the research without access to and use of identifiable health / medical information.

For example, the data cannot be obtained from electronic health / medical records or databases without access to identifiers or identifiers are needed for prospective data collection.

It would be impossible to conduct this research without access to identifiable health information as this information will be use to track which patients in the practice are eligible for recruitment, who has been approached, and who has consented/declined.

How will the protected health information (PHI) recorded for the research be stored and protected?

For paper-based information, describe where the identifiable information will be stored, who has access to the storage area, and how that access will be audited. If the information is stored off-site, describe how security at the facility is maintained and whether or not a business associate agreement has been or will be signed. At a minimum, consider storing the PHI in locked drawers, cabinets, or offices with access restricted to the princiapl investigator (PI) and study staff designated by the PI.

For electronic information, describe how electronic security is maintained, including what password protections and antivirus software are enabled. Describe how the system will be audited. At a minimum, consider storing PHI in a password-protected Mass General Brigham computer with anitvirus software.

All data will be stored in REDCap.

All data collection involving HPI with identifiers will occur at MGH on MGB-managed computers, whose security profile is managed by MGB Information Systems (password protections, antivirus, etc). All data identified data will be stored and analyzed on a MGB computer (Clapp - stored in MGH AUS 438). Identifiable data will be limited to MGB study staff by use of password protected files or restricted shared file areas. De-identified data will be analyzed on computers/networks in accordance with MGB policies.

Will identifiers be removed from the data and destroyed after all of the data has been collected, the study has been completed, or all regulatory and sponsor obligations have been met, consistent with regulatory and institutional research record keeping requirements?

For guidance, see the Recordkeeping and Record Retention Requirements document.

- (●) Yes
- ( ) No

NOTE: Federal regulations mandate that, under a Waiver of Consent / Authorization, identifiers be destroyed as early as possible. De-identified datasets may be retained indefinitely.

Waiver of Informed Consent / Authorization

Explain why the risk to subjects, specifically the risk to privacy, is no more than minimal risk.

MRNs will be the primary personal identifier that will be used in the data collection process. Once the data collection is completed, the MRN will be replaced by a unique patient identifier before any analyses are performed and all other identifying information will be removed. All data collection involving HPI with identifiers will occur at MGH on MGB-managed computers, whose security profile is managed by MGB Information Systems (password protections, antivirus, etc). All data identified data will be stored and analyzed on a MGB computer (Clapp - stored in MGH AUS 438). Identifiable data will be limited to study staff by use of password protected files or restricted shared file areas. De-identified data will be analyzed on computers/networks in accordance with MGB policies.

Explain why the research could not practicably be carried out without the waiver of consent / authorization.

We anticipate that we will screen over 1000 patients to determine eligibility; the costs, time, and resources to locate and obtain consent for reviewing a patients medical record to determine eligibility criteria would be prohibitive for this study. Furthermore, the bias introduced by studying only consenting patients will limit the interpretability and generalizability of the findings. To protect the private of subjects, MRN will be the only identifier collected and PHI will be destroyed after the data set is constructed.

NOTE: "Only in a few research studies would it be impossible to obtain informed consent; however in many studies the financial cost would be prohibitive and a potentially poor use of limited research resources." ***Ensuring Voluntary Informed Consent and Protecting Privacy and Confidentiality, National Bioethics Advisory Commission.***

Explain why the rights and welfare of the subjects will not be adversely affected by the waiver of consent / authorization.

The welfare and rights of the subjects will not be affected by the waiver of consent because the study is retrospective and will occur after the pregnancy episode has completed. Information gathered will not directly affect the individual patients in the study. Lastly, study subjects' privacy will be protected by limiting the identifiable data to the study staff and having this data stored securely within the MGB Healthcare IS system. Study subjects will remain anonymous; patients will not be personally identifiable from any published or produced data from this project.

NOTE: If the research uncovers information about the subjects that has important health / medical implications for them, contact the MGB IRB to discuss the appropriate process for providing subjects with additional pertinent information.

Sending Health / Medical Information to Collaborators Outside Mass General Brigham

Will any health / medical information be sent to collaborators outside Mass General Brigham?

- (●) Yes
- ( ) No

Explain what health information will be sent, and to whom.

Deidentified data collected in this study will be sent to Dr. Jessica Cohen (HSPH) to assist in the analysis and made publicly available via the funder, as part of their federal data sharing policy. No data will be shared without oversight/approval/DUA by the IRB and Office of Research Management.

HIPAA And Tracking Disclosures Of Identifiable Health Information (PHI)

1. Disclosures of PHI to persons or entities outside Mass General Brigham without the written authorization of the subject must be tracked in accordance with Mass General Brigham policy "Accounting of Disclosures." (MGB Intranet link)

2. Tracking is NOT required for disclosure of LIMITED DATA SETS under a DATA USE AGREEMENT. For more information about LIMITED DATA SETS and DATA USE AGREEMENTS, refer to Mass General Brigham policy "Limited Data Sets Policy/Data Use Agreements."

NOTE: Mass General Brigham (MGB) is the HIPAA covered entity. MGB includes BWH, Faulkner, MGH, McLean, PCHI, SRH, NSMC, and NWH, among others. MGB does not include other Harvard affiliated hospitals, such as BIDMC, DFCI, HSPH, CHB, or MEEI.

Pregnant Women or Human Fetuses

Federal regulations require the IRB to provide additional protections for pregnant women and human fetuses involved in research [45 CFR 46.204].

Assessing Risks And Benefits

When assessing risks and benefits, consider the variability in health status of the subjects to be enrolled, their medical experiences, and the extent to which the research procedures will be a burden to the subjects in the context of their daily lives and/or routine medical care. Procedures that usually present no more than minimal risk include: urinalysis, obtaining a small amount of blood, EEGs, allergy scratch tests, minor changes in diet or daily routine, and/or the use of standard psychological or educational tests. The

Mass General Brigham

assessment of the probability and magnitude of the risk, however, may vary depending on the diseases or conditions the subjects may have.

Minimal Risk
As defined in the regulations 45 CFR 46.102(i), “minimal risk means the probability and magnitude of harm or discomfort anticipated in the research are not greater in and of themselves than those ordinarily encountered in daily life or during the performance of routine physical or psychological examinations or tests.”

Pregnant women or fetuses may be involved in research if **all** of the following conditions listed below are met [45 CFR 46.204]. **Please provide study-specific information with your explanations.**

(a) Where scientifically appropriate, pre-clinical studies, including studies on pregnant animals, and clinical studies (including studies on non-pregnant women) have been conducted and provide data for assessing potential risks to pregnant women and fetuses.

Provide a brief description of relevant prior pre-clinical and clinical studies, and based on this information, what you think the risks to pregnant women and to the fetus are in your research.

This study is minimal risk for all individuals. There are no additional, specific risks to pregnant women or fetuses as part of this research.

(b) The risk to the fetus is caused solely by the interventions or procedures that hold out the prospect of direct benefit for the woman or the fetus; if there is no such prospect of direct benefit, the risk to the fetus is not greater than minimal and the purpose of the research is the development of important biomedical knowledge which cannot be obtained by other means.

Does the research hold out the prospect of direct benefit for the woman, or to the fetus?

- ○ Yes
- ◉ No

Explain why you believe the risk to the fetus is **not** greater than minimal risk.

This study will enroll pregnant individuals and involve sending them messages about the importance of primary care follow-up.

Explain what new important knowledge will be gained, and why the information could not be obtained by any other means.

This study aims to improve care transitions between pregnancy/postpartum providers and primary care providers. By its nature, there is no other way this information could be gained without studying pregnant individuals.

(c) Any risk is the least possible for achieving the objectives of the research.

Explain how the risks have been minimized to the least possible to achieve the research objectives.

The risks have been minimized such that only the minimum necessary information will be collected. To protect the privacy of subjects, MRN will be the only identifier collected and PHI will be destroyed after the study has completed.

Informed Consent

Check all that apply:

- ☑ Informed consent will be obtained from the **pregnant woman** in accordance with the consent provisions of Federal regulation 45 CFR 46.116.

- ☐ When the research holds out the prospect of **direct benefit solely to the fetus**, informed consent will be obtained from the **father** in accordance with the consent provisions of 45 CFR 46.116, except, if he is unable to consent because of unavailability, incompetence, or temporary incapacity, or the pregnancy resulted from rape or incest.
- ☐ **Each individual providing consent will be fully informed** regarding the reasonably foreseeable impact of the research on the fetus. This information is included in the Mass General Brigham Research Consent Form.

MASSACHUSETTS GENERAL LAW CHAPTER 112, SECTION 12J

Experimentation on human fetuses prohibited; medical procedures authorized; consent; approval; civil and criminal liability and proceedings; severability.

(a)I. No person shall use any live human fetus whether before or after expulsion from its mother's womb for scientific, laboratory, research or other kind of experimentation. This section shall not prohibit procedures incident to the study of a human fetus while it is in its mother's womb, provided that in the best medical judgment of the physician, made at the time of the study, said procedures do not substantially jeopardize the life or health of the fetus, and provided said fetus is not the subject of a planned abortion.
(a)II. No experimentation may knowingly be performed upon a dead fetus unless the consent of the mother has first been obtained, provided, however, that such consent shall not be required in the case of a routine pathological study.
(a)III. No person shall perform or offer to perform an abortion where part or all of the consideration for said performance is that the fetal remains may be used for experimentation or other kind of research or study.

Attachments

Name	Mode
Study Fact Sheet_071522 (Consent Fact/Information Sheet)	Electronic
Detailed Protocol_071522	Electronic
Roybal DSMB Charter Final (DSMB/DMC Report)	Electronic
DSMP_Cohen_Clapp (DSMB/DMC Report)	Electronic
Prescreening Information (Data Collection Form)	Electronic
PCP Study Baseline Survey (Instrument/Questionnaire)	Electronic
Patient Gateway Research Invitation Letter (Recruitment Letter)	Electronic
Schema (Schema)	Electronic
Patient Messages (Document for review)	Electronic

Mass General Brigham

Research Study Fact Sheet

Bridging the Gap from Postpartum to Primary Care (The Bridge Study)

Principal Investigators

Mark Clapp, MD MPH (Massachusetts General Hospital)
Jessica Cohen, PhD (Harvard School of Public Health)

Overview

This is a research study being conducted among pregnant and recently postpartum individuals who receive care at MGH.

Purpose

Your primary care provider (PCP) is responsible for managing and overseeing your current and long-term health, and many people do not regularly see their PCP after a pregnancy. The purpose of this project is to improve the transition of care between your obstetric care and PCP after your delivery.

Sponsor

This research is being sponsored by the MIT Roybal Center for Translational Research to Improve Health Care for the Aging and the NBER Roybal Center for Behavior Change in Health.

Contact

We sent you a Patient Gateway message about this study because you have or may be at risk for health conditions later in your life, such as high blood pressure, diabetes, obesity, or anxiety or depression.

Study Details

If you agree to participate, you we will ask you a few questions about your health and visit history at the time of enrollment, which are all optional.

You will then be randomly assigned (50/50 chance) to be in the “standard care” group or the “facilitated transition” group. You will not be informed which group you have been assigned.

In the “standard care” group, you will continue to receive standard postpartum care from your primary obstetric provider. In addition, you will receive Patient Gateway and optional SMS (text) messages from the study team.

In the “facilitated transition” group, you will continue to receive standard postpartum care from your primary obstetric provider. In addition, you will receive Patient Gateway and optional SMS (text) messages from the study team between your enrollment and up to four months after your delivery. The study team will also assist in connecting you with your primary care provider after your postpartum visit with your obstetric provider. Specifically, we may call your primary care provider’s office on your behalf and request a “health care maintenance” or “annual exam” appointment be scheduled. You always have the option to change or cancel this appointment.

Messages we send you via Patient Gateway will also appear in your medical record for other members of your care team to see.

For both groups, we will review your medical record after your delivery to see if/when you received any care within the Mass General Brigham health system and information on your health status for up to two years after your delivery.

We also may contact you once between four and twelve months after your delivery to ask you questions about your postpartum to primary care transition experience. You can decline to participate or answer any or all questions.

You may opt out of receiving messages or decline to participate in any survey, and/or withdraw from the study at any point without penalty.

A total of 360 people will participate in this study.

Privacy/Confidentiality

We are required by the Health Insurance Portability and Accountability Act (HIPAA) to protect the privacy of health information obtained for research. More details related to the privacy of your health information is included on Pages 3-4 of this fact sheet.

All information collected as a part of this study will be kept confidential and secure. As with all studies, there is a very low risk that others may become aware of your participation or health information. The study procedures and its protections of your information have been approved by the Mass General Brigham Human Subjects Research committee. Once the study has completed, all information that could identify you, such as your name or medical record number, will be deleted.

Study Information

We will collect information on your health and pregnancy history, including about visits you have with providers within the Mass General Brigham health system. Data collected as a part of this study will not affect your clinical care directly. Any messages sent from the study team will be visible to you and your care team within the electronic medical record. Your de-identified information may be used or shared with other researchers without your additional informed consent.

Risks

There are minimal risks to participate.

Although we will make every effort to protect participant privacy and confidentiality, it is possible that your involvement in the study could become known to others. We will be sending messages via Patient Gateway, which is a HIPAA-compliant method for communicating with you. You have the option to also receive SMS text messages that will not have any identifiable or personal health information. There is a rare possibility that study participation or health information could become known to others despite the use of firewalls, password protection, and other security measures.

Costs

You do not have to pay to take part in this research study. By attending a PCP visit, there is a possibility that you will incur a charge. Under the Affordable Care Act, commercial health plans are required to cover an annual Preventive Health Exam at no cost to the patient (no co-payment, co-insurance or deductible). MassHealth also covers these visits without a cost. However, if this visit turns into a “sick” or “disease management” visit, you may be billed for some or all aspects of the services provided, depending on your insurer. We will provide you information on how to check potential costs with your insurer prior to your visit.

Participation

Those who participate will receive a $20 gift card after enrollment.

Participation is voluntary, and you can withdrawal or stop the study at any time. Deciding not to participate won't affect medical care they receive at Mass General Brigham now or in the future, or any benefits they receive now or have a right to receive.

Study Contact

Mark Clapp, MD MPH, is the person at MGH who in charge of this research study. You can call him at 617-724-4531 (M-F 9a-5p). You can also call the study coordinator (Fowsia Warsame) at 617-643-5483 (M-F 9a-5p) with questions about this research study.

If you’d like to speak to someone not involved in this research about your rights as a research subject, or any concerns or complaints you may have about the research, contact the Mass General Brigham IRB at (857) 282-1900.

Option to Receive Text (SMS) Messages

Text messages by mobile/cell phones are a common form of communication. The Bridging the Gap from Postpartum to Primary Care study would like to send you no more than 3 text (SMS) messages as a part of the study, though **this is optional and not required to participate in the Bridge Study**. Texting over mobile/cell phones carries security risks because text messages to mobile/cell phones are not encrypted. This means that information you send or receive by text message could be intercepted or viewed by an unintended recipient, or by your mobile/cell phone provider or carrier.

Below are some important points about texting in as a part of a research study:

- Receiving text messages is optional in this study and not required to participate.
- Text messages are not encrypted, and therefore carry security risks. This research study and Mass General Brigham are not responsible for any interception of messages sent through unencrypted text message communications.
- You will be responsible for all fees charged by your carrier's service plan for text messaging. This research study and Mass General Brigham are not responsible for any increased charges, data usage against plan limits or changes to data fees from the research texts.
- Text messaging should not be used in case of an emergency. If you experience a medical emergency, call 911 or go to the nearest hospital emergency department.
- You may decide to not send or receive text messages with staff associated with this research study at any time. You can do this in person or by sending the research number a text message that says "Stop Research Text."
- Your agreement applies to this research study only. Agreeing to other texts from Mass General Brigham, for example appointment reminders, is a separate process. Opting out of other texts from Mass General Brigham is a separate process as well.
- It is your responsibility to update your mobile/cell phone number with this research study in the event of a change.

Information Regarding the Privacy of Your Health Information

Federal law requires Mass General Brigham and its affiliated hospitals to protect the privacy of health information and related information that identifies you. We refer to this information as "protected health information." Your protected health information will be used and shared with others as explained below. You are agreeing to the collection, use, and sharing of your protected health information as described in this information sheet. If you have questions, you may ask the researcher who is reviewing this information sheet with you or you can contact the researcher listed above.

In this study, we may collect protected health information about you from:

- Past, present, and future medical records
- Research procedures, including but not limited to research visits, tests, interviews, and questionnaires

<u>Why will protected health information about you be used or shared with others?</u>
The main reasons include:

- to conduct and oversee the research described in this information sheet for this study;
- to ensure the research meets legal, institutional, and accreditation requirements; and
- to conduct public health activities (including reporting of adverse events or situations where you or others may be at risk of harm).

<u>Who may see, use, and share your protected health information and why they may need to do so?</u>

- Mass General Brigham researchers and staff involved in this study
- The sponsor(s) of the study, and people or groups it hires to help perform this research or to audit the research

- Other researchers and medical centers that are part of this study
- The Mass General Brigham ethics board
- A group that oversees the data (study information) and safety of this study
- Non-research staff within Mass General Brigham who need identifiable information to do their jobs, such as for treatment, payment (billing), or hospital operations (such as assessing the quality of care or research)
- People or groups that we hire to do certain work for us, such as data storage companies, accreditors, insurers, and lawyers
- Federal agencies (such as the U.S. Department of Health and Human Services (DHHS) and agencies within DHHS like the Food and Drug Administration, the National Institutes of Health, and the Office for Human Research Protections), state agencies, and foreign government bodies that oversee, evaluate, and audit research, which may include inspection of your records
- Public health and safety authorities, if we learn information that could mean harm to you or others (such as to make required reports about communicable diseases or about child or elder abuse)

Some people or groups who get your protected health information might not have to follow the same privacy rules that we follow and might use or share your protected health information without your permission in ways that are not described in this form. We share your protected health information only when we must, and we ask anyone who receives it from us to take measures to protect your privacy. However, once your protected health information is shared outside Mass General Brigham, we cannot control all the ways that others use or share it and cannot promise that it will remain private.

The results of this research study may be published in a medical book or journal or used to teach others. However, your name or other identifiable information **will not** be used for these purposes without your specific permission.

For how long will protected health information about you be used or shared with others?
Because research is an ongoing process, we cannot give you an exact date when we will either destroy or stop using or sharing your protected health information. Your permission to use and share your protected health information does not expire.

Your Privacy Rights
You have the right not to agree to our use and sharing of your protected health information for research; however, if you don't agree, you can't take part in this research study. However, refusing to agree will not affect your present or future care and will not cause any penalty or loss of benefits to which you are otherwise entitled.

You have the right to withdraw your permission for the further use or sharing of your protected health information for this research study. If you want to withdraw your permission, you must notify the person in charge of this research study in writing at the name and address listed above. Once permission is withdrawn, you cannot continue to take part in the study.

If you withdraw your permission, then to the extent that we have retained any protected health information that can be linked to you, we will stop using and sharing the protected health information further for the research. However, we will not be able to take back information that has already been used or shared with others, and such information may continue to be used for certain purposes, such as to comply with the law or maintain the reliability of the study.

You have the right to see and get a copy of your protected health information that is used or shared for treatment or for payment. To ask for this information, please contact the person in charge of this research study identified above. You may only get such information after the research is finished.

Mass General Brigham

Institutional Review Board
Intervention/Interaction Detailed Protocol

Principal Investigator:	Mark Clapp, MD MPH (MGH), Jessica Cohen, PhD (HSPH)
Project Title:	Bridging the Gap from Postpartum to Primary Care: A Behavioral Science Informed Intervention to Improve Chronic Disease Management among Postpartum Women
Version Date:	07/15/2022
Version Name/Number:	v1.2

1. Background and Significance

Burden of Chronic Disease and the Role of Primary Care
Chronic health conditions affect millions of people in the US each year. In 2018, 51.8% of adults had at least 1 chronic condition, and 27.2% had multiple conditions.[1] The prevalence of chronic disease was higher in women compared to men, older adults (87.6% in adults ≥65 years old), and people with public insurance.[1] Many chronic conditions, by their nature, develop over time and have risk factors that can be identified prior to the onset of disease. Strong evidence underpins prevention strategies for many conditions, which are advanced by the US Preventative Services Taskforce.[2] While the long-term health of a patient is the responsibility of an entire health system, primary care providers (PCPs) provide an integral role in preventing, screening for, and managing disease across the lifespan. Studies have shown the health benefits of receiving regular care under a PCP.[3–6]

Despite the known benefits of having an identifiable usual source of care and the value of health care maintenance, the percent of the population with a PCP has been decreasing over time.[7] Consistently, adults who are younger (age 20-40 years) have the lowest rates of primary care use. In 2015, 44% and 36% of 20- and 30-year-olds had no identifiable source of primary care.[7] The proportion without primary care were also higher among racial/ethnic minority populations and among those who had less education, lower incomes, and no known comorbidities.[7] The number of adults and the time elapsed without regular primary care follow-up can be considered missed opportunities to improve a patient's current and long-term health. The disproportionate lack of primary care among certain subgroups of the population, often groups who already have worse health outcomes, only serves to widen the pre-existing disparities.

Pregnancy as a Window to Future Health
In the US, 98.2% of pregnant women receive some form of prenatal care, with the average patient having >10 visits during their pregnancy.[8] During a pregnancy, women are screened for pre-existing and pregnancy-related conditions.[9] In adults ages 18-39, the prevalence of obesity, hypertension, prediabetes/diabetes, and mental illness are estimated at 39%, 7.5%, 28%, 25%, respectively.[10–13] Even for those who have no prior identified comorbidities, the most common

pregnancy-related conditions—pregnancy-related hypertension and gestational diabetes (8% and 10% of pregnancies, respectively)—indicate a predisposition to or confer health risks that persist as women age. For example, over 25% of women with gestational diabetes will develop Type 2 diabetes mellitus, and women with pre-eclampsia have more than a two-fold risk of significant cardiovascular disease later in life.[14,15] For these reasons, pregnancy is often considered a "window" into a woman's future health and presents a unique opportunity to optimize a woman's health status early in her life when she otherwise may not have been engaged in care.[16]

Pregnancy as on Opportunity for Engagement with a Long-term Care Provider
Pregnancy is a period when women are highly engaged and active participants in their health care.[17] It has been described as a "golden opportunity" to motivate women towards positive health behaviors, including prevention and management of chronic disease. However, women often fall off a "postpartum cliff" of health system engagement after the early postpartum period.[18] A range of systemic, financial, and behavioral barriers often prevents patients from effectively transitioning to primary care. Postpartum women are often simply told to follow-up with their PCP without much information regarding the importance of this follow up care, without assistance in scheduling an appointment (or identifying a PCP if they don't have one), and often without a direct transfer of relevant health information or accountability across providers.

Postpartum women are left largely on their own to navigate this transition to primary care and, in particular, to navigate it at a time when they face the high cognitive and physical demands of caring for an infant. At this time of limited cognitive bandwidth, the importance of continuity of care for chronic conditions and active engagement in one's longer-term health and wellbeing is unlikely to be salient and top-of-mind. These critical moments of unsupported health care transition can exacerbate pre-existing disparities in health and health care, with patients who are the least able to navigate the US health care system most likely to fall through the cracks. Momentum is building in US health and social safety net policy to facilitate healthy transitions from pregnancy to parenthood. For example, federal and state initiatives to expand pregnancy-related Medicaid coverage from 60 days to one year postpartum have been proposed, and access to paid family leave is increasing. However, very little evidence exists on effective and cost-effective approaches to facilitating transitions to primary care and management of chronic diseases in the postpartum year.

2. Specific Aims and Objectives

The objective of the proposed study is to increase patient engagement in primary care after the immediate postpartum period for women with pregnancy-associated conditions that convey a long-term health risk. Specifically, we aim to evaluate the efficacy of an intervention bundle (patient-tailored health information, automatic scheduling of PCP appointment after delivery, and appointment reminder nudges) to increase patient attendance at a routine health care maintenance appointment (i.e., "annual exam") within 4 months of delivery for women with obesity, diabetes, hypertension, and/or a mental health condition.

Specific Aims:

1) Test the efficacy of an intervention bundle (patient-tailored health information, automatic scheduling of PCP appointment after delivery, and appointment reminder nudges) to increase patient attendance at a routine health care maintenance appointment within 4

months of delivery for women with obesity, diabetes, hypertension, and/or a mental health condition
2) Test the efficacy of the intervention bundle to improve compliance with the condition-specific, guideline-based health screenings
3) Test the efficacy of the intervention bundle to reduce unscheduled or urgent encounters (e.g., emergency department visits) within the health system

3. General Description of Study Design

We will conduct a randomized controlled trial comparing this intervention bundle to the receipt of generic information on the importance of primary care follow-up after delivery.

Women will be randomized with equal probability into either a treatment or control arm, stratified by whether their pregnancy is covered by Medicaid or a commercial insurer and by patient race (white vs. non-white). The intervention combines several features designed to target reasons for low take-up of primary care among postpartum women (see Logic Model). We leverage the potential value of defaults/opt-out, pre-commitment, salient information, and reminders to encourage use of primary care within 4 months of delivery. Women in both the intervention and control arms will receive information via MGH's patient portal toward the end of the pregnancy regarding the importance and benefits of primary care in the postpartum year. This information will be similar to, but reinforcing, the information they would receive from their obstetrician about following up with their primary care physician. In addition to this initial message, women in the treatment arm will receive the following intervention components, developed based on recent evidence regarding behavioral science approaches to activating health behaviors:[19–21]

- Targeted messages about the importance and benefits of primary care, tailored to her condition(s). For example, for women with hypertensive disorders, the intervention will include information about the risk of developing chronic hypertension and the importance of blood pressure screenings to detect and manage this condition.
- Default scheduling into a primary care appointment at approximately 3-4 months after delivery. The patient will be scheduled for a primary care visit with their assigned primary care provider in the Mass General Brigham system. They will be given the opportunity to cancel the appointment, change the appointment day/time, or change the care provider either through the patient portal or over the phone with a research assistant working with the study (whichever they choose).
- Reminders about the appointment and importance of follow up primary care at 2-4 points during the postpartum period via the patient portal.
- Tailored language in the reminders based on recent evidence from behavioral science about the most effective approaches to increasing take-up. For example, messages will inform the patient that an appointment is being held for them at their doctor.

The Logic Framework that underpins the basis for this study is shown below:

Needs/ Problems	Intervention Logic Framework			Long-term Goals
	Barriers to Take-Up of Primary Care	Input/Intervention	Outcomes	
• Low use of primary care among people who have/are at risk of chronic conditions (diabetes, hypertension, obesity, mental illness)<br>• Pregnancy is ideal opportunity to engage in prevention and management of chronic conditions and interrupt progression of chronic disease, but this window into long-term health is missed	• Insufficient patient information<br>• Under-estimation of risks<br>• Low salience<br>• Cognitive demands in postpartum period<br>• Time constraints and competing priorities<br>• Health system/structural barriers to postpartum care continuity<br>• Poor transitions between obstetric and primary care providers	• Targeted information about importance of primary care for patient's specific condition<br>• Default patients into postpartum primary care appointment<br>• Reminder messages about primary care appointment leveraging salience and pre-commitment<br>• Patient checklist regarding important issues to discuss with PCP	**PRIMARY**<br>• Healthcare maintenance visit with primary care provider within 4 months of childbirth<br>**SECONDARY**<br>• Condition-specific screening:<br>-documentation of blood pressure<br>-diabetes screening test<br>-measurement of weight<br>• Counseling on risk prevention or reduction strategies at visit<br>• Use of emergency room or urgent care for any reason	• Engagement with primary care provider<br>• Earlier, more effective prevention and management of diabetes, hypertension, mental illness and obesity<br>• Interruption of disease progression over life course<br>• Improved lifelong health status

The flow of study activities is shown below:

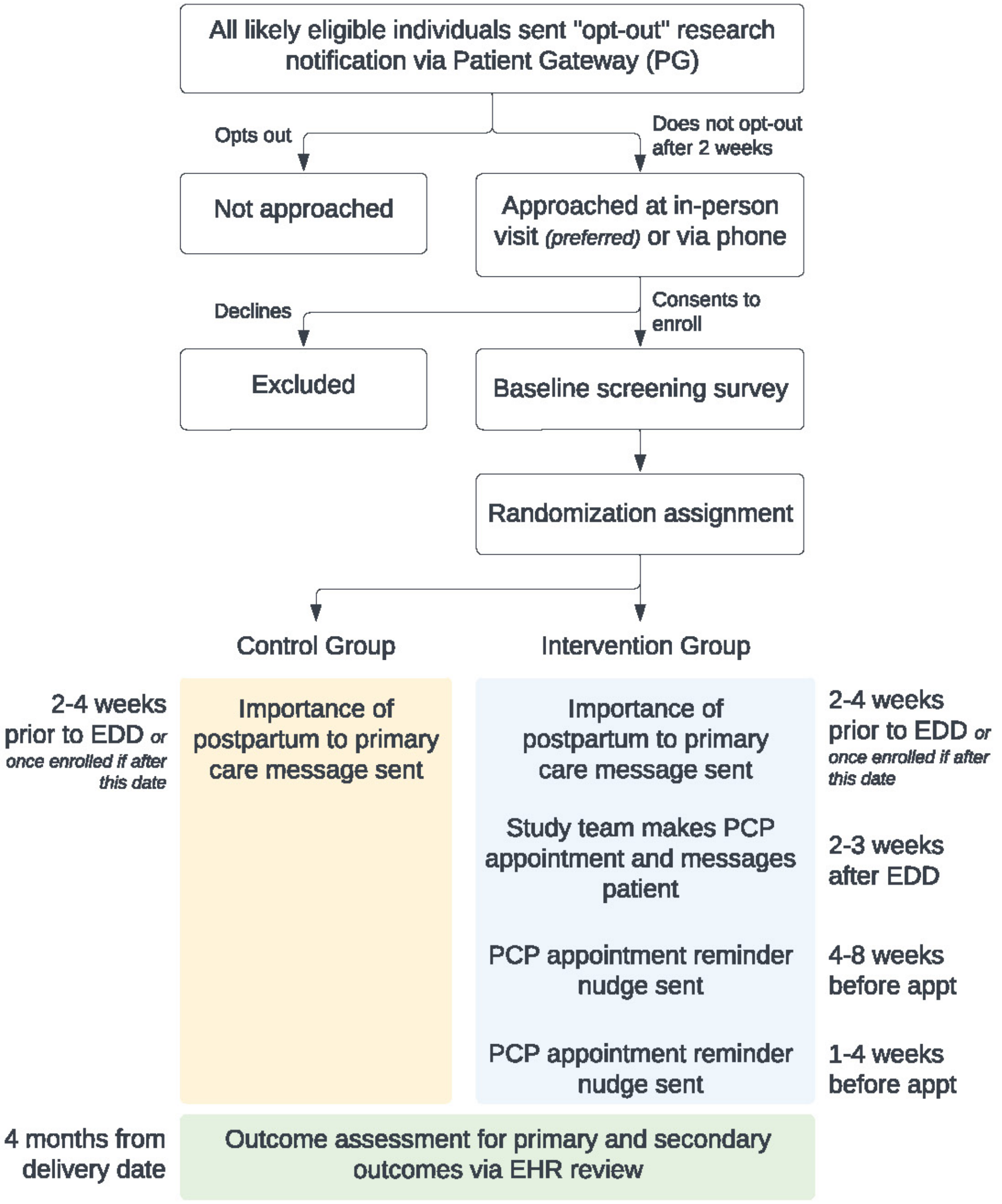

4. Subject Selection

Pregnant patients receiving prenatal care at Massachusetts General Hospital (MGH) will be targeted for recruitment. MGH conducts approximately 3500 deliveries per year, with roughly one-third of patients identifying as non-white and 35% of pregnancies covered by Medicaid.

Approximately 40% of the clinic population may ultimately be eligible to be approached.

The eligibility criteria include:

- Estimated date of delivery and the following 4-month postpartum outcome assessment window completed prior to study end date
- Currently pregnant or within 2 weeks of delivery
- Have one or more of the following conditions:
    - Chronic hypertension
    - Hypertensive disorders of pregnancy or risk factors for hypertensive disorders of pregnancy per the USPTF aspirin prescribing guidelines (e.g., history of pre-eclampsia, kidney disease, multiple gestation, autoimmune disease)[22]
    - Type 1 or 2 diabetes
    - Gestational diabetes
    - Obesity (body mass index ≥30 kg/m2)
    - Depression or anxiety disorder
- Have or be willing to be assigned a primary care provider within the Mass General Brigham network
- Receive obstetric care at an MGH-affiliated outpatient prenatal clinic
- Has access to and be enrolled in the electronic health record patient portal and consents to be contacted via these modalities
- Able to read/speak English or Spanish language
- Age ≥18 years old

All women, regardless of race/ethnicity, who meet the eligibility criteria will be included. In 2019, 3,789 women gave birth at the study institution, of whom 57% were white, 7% were black, 12% were Asian, 18% were Hispanic, and 6% declined to report their race/ethnicity. A similar distribution is expected for this study.

The study will distribute materials in English and Spanish languages. This encompasses >95% of patients who deliver at the study institution.

5. Subject Enrollment

This study will rely on recruiting for research through Patient Gateway and follow the IRB guidance and DHeC Research Checklist and training for this process.

In the month prior to the start of enrollment, all potentially eligible individuals (based on the criteria above) will be identified using RPDR and Epic Reporting search queries. This list (the "potentially eligible" list) will be provided to the DHeCare Research Team to build an RSH Record in Epic. This list will be updated monthly to identify newly eligible individuals (e.g., new diagnosis of gestational diabetes or new patient transferring into the practice) during the recruitment months and fed back to the DHeCare team to update "potentially eligible" list.

Once built and each month during recruitment phase, the study's research coordinator (not study investigators) will send the IRB-approved Research Invitation Letter to patients who are eligible, not already enrolled, and have not declined to be sent research notifications through the portal. The Research Letter will employ an opt-out approach, asking individuals who do not wish to be approached in clinic or remotely to respond via PG messaging, email, or phone within 2 weeks of receipt of the letter. Those who have "read" the letter and not opted out after 2 weeks

will be moved from the “potentially eligible” list to the “waiting to be approached” list in the study workflow.

During recruitment months, the study coordinator will keep a log of patient’s upcoming appointments for those on the “waiting to be approached” list. They will then attempt to approach individuals for enrollment when they present for an in-person encounters (preferred). Patients will ideally be approached between 32-36 weeks of gestation, when feasible; however, priority will be given to patients at the latest gestation. For those Spanish-speaking patients, a hospital-based interpreter will be used when approaching/consenting patients.

The study staff will introduce the study and review the purpose of the study, the nature of the subject’s participation, the possible risks and discomforts associated with participation, the potential benefits of participation, a statement of the voluntary nature of participation, and a description of the mechanisms used to ensure confidentiality.

All patients will receive study-related messages through Patient Gateway. In addition, patients will be asked if they would be willing to receive no more than 3 SMS messages to their personal cell phone during the first 4 months of the postpartum period. The study staff will review the specific concerns and risks about receiving unencrypted text messaging communications, as outlined by the MGB IRB.

A waiver of documentation of informed consent is requested, as the study presents no more than minimal risk of harm to subjects and involves no procedures for which written consent is normally required outside of the research context. Verbal consent will be obtained for 1) overall study participation and 2) optional SMS message participation, separately. Verbal consent will be obtained from both in-person and phone recruitment efforts.

A Study Fact Sheet, which summarizes the study details, risks, and benefits, will be provided to all subjects who are approached, either in-person, by mail, or electronically. This Study Fact Sheet also includes information supplied by the MGB IRB specifically related to the concerns and risks of receiving unencrypted SMS/text messages.

For tracking verbal consent, the study staff will keep a detailed log in REDCap documenting:

1) name of study staff performing consent,
2) date of attempted approach,
3) the method of attempted approach (in-person or via phone),
4) use of Spanish interpreter (yes/no),
5) subject agreement to be approached (agree/disagree),
6) attestation to full review of the study procedures/risks/benefits with the subject, as would be done during the process of reviewing a written consent form,
7) attestation to review of supplemental consent to receive unencrypted SMS messages with the subject,
8) subject overall study participation status (enrolled, declined, deferred – agrees to be recontacted, deferred – wishes not to be recontacted),
9) if enrolled, unencrypted SMS text messaging participation (consents, declines),
10) attestation to Study Fact Sheet provided,
11) method by which Study Fact Sheet was provided (in-person, mail, electronic),
12) date in which Study Fact Sheet was provided (in-person, mail, electronic).

6. STUDY PROCEDURES

The RA will keep a detailed log of all patients in the practice, if they are eligible, if they have been approached, and if they consented.

For those that agree to be enrolled, patients will be asked to complete a baseline survey to obtain voluntarily reported information on their demographics, socioeconomic status, health care visit history, and preferred primary care provider. Patients will also be asked to consent to being contacted by the research team via Patient Gateway messaging. A $20 gift card will be given at the time of enrollment for those that complete the questionnaire and receive the information sheet.

Randomization will occur via a prespecified random allocation sequence within strata. Within each stratum, the PIs will generate a random sequence of treatment-control allocation prior the enrollment of subjects. Then, as patients within strata are enrolled, they will be assigned to the intervention arm associated with that enrollment number.

<u>Control Group</u>
Approximately 2 weeks before a patient's estimated due date (EDD) or as soon as enrolled if this date has passed, the patient will be sent an information via the Patient Gateway on the importance of postpartum care and follow-up with their PCP.

<u>Intervention Group</u>
1. Tailored Information:
   Approximately 2-4 weeks prior to the EDD (or later for those who are enrolled beyond 38 weeks gestation or postnatally), the patient will be sent an information via the Patient Gateway on the importance of postpartum care and follow-up with their PCP, which also includes the name and phone number of their primary care provider and tailored information regarding their specific condition(s) (diabetes, hypertension, obesity, mental health).
2. Scheduled PCP Appointment:
   Between 2-3 weeks after their delivery, the RA will call the patient's PCP office and make an appointment for them between 3-4 months after delivery based on the scheduling preferences obtained in the initial survey.
3. Targeted Appointment Message:
   After the PCP appointment has been made, the patient will be sent a Patient Gateway message saying that a PCP appointment has been reserved for them with the date/time/location information.
   For those consenting to receive SMS messages, an unencrypted text message will also be sent simultaneously.
4. Nudge Reminders:
   Patient Gateway messages will be sent at approximately 4-8 weeks (goal: 4 weeks) and 1-4 week (goal: 1 week) prior to their PCP appointment, reminding a patient of their upcoming appointment.
   For those consenting to receive SMS messages, an unencrypted text message will also be sent simultaneously.

All messages will be made available in English and Spanish.

The text for these Patient Gateway messages is included in the submission. At the end of each patient message, patients will be given the opportunity to stop receiving study-related messages by emailing or calling the study staff. “Opt out” requests will be logged and patients removed from future planned study-related contact.

The text for 3 SMS messages is included in the submission. SMS messages will be sent via Google Voice. We will follow standard recommendations from the MGB Research Information Security for using this system (included under Privacy and Confidentiality section).

The study team will review the patient’s EHR record for the primary and secondary outcome assessments. Patients will be asked to consent to have records reviewed up to 2 years after the date of their delivery to allow for long-term effects of the intervention on primary care use and health status.

During the initial consent process, patients will be asked to agree to be potentially contacted at the end of the first 4-month follow-up period for a survey. Currently, this endline survey is not planned due to funding limitations; however, if funding becomes available, an IRB amendment will be submitted for review of the endline survey prior to being administered to any study subjects.

Deidentified data from this project may be shared outside of MGB with 1) Harvard T. H. Chan School of Public Health (Dr. Jessica Cohen) and 2) the study funders (J-PAL/NBER/NIA) for data sharing and reproducibility requirements and secondary statistical analysis. All data will be stripped of patient identifiers, per IRB guidance. No data will be shared without a formal Data Use Agreement with MGB.

5. Risks and Discomforts

There are minimal risks to participants.

Patients will be reassured that nonparticipation will not affect clinical care. Patients will also be informed that the researchers with whom they will interact (e.g., during consent, face-to-face, during telephone interviews) are not health care providers. Participants will be consistently reminded that responses to any queries deemed sensitive or uncomfortable (e.g., country of origin/immigration status, income, previous history of abortion) should be considered optional, and they may decline to answer any question(s) and can refuse to continue the study at any point. Participants will be reassured that neither their opportunities for continued health care nor their relationships with health care providers will be jeopardized by study participation.

Health information collected as part of this study will be stored in REDCap. No identifiable data will be stored or downloaded on any personal or unauthorized computers. Study staff will access the data on institutionally purchased and managed computers that operate behind the health system’s security and firewall protections. Only the MGH study staff will have access to the identifiable data set. Once the study is completed, data will be deidentified such that it can be analyzed without risk of a breach of privacy or confidentiality. Any data that is shared outside of MGH will require a data use authorization.

Although we will make every effort to protect participant privacy and confidentiality, it is possible that participants' involvement in the study could become known to others. For those in the intervention group, we will be sending personalized messages via the patient portal; for those providing additional consent, we will be sending unencrypted SMS messages that will not contain any personal protected health information. There is the rare possibility that study participation or health information could become known to others despite the use of firewalls, password protection, and other security measures. Standard procedures, as outlined by the MGB Research Information Security Office, will be followed to reduce this risk.

Those in the intervention arm will receive condition-specific information on pregnancy comorbidities and their implications on their long-term health. This information may cause anxiety or worry among patients; however, this same information is routinely reviewed by obstetric providers at visits.

Individuals in the intervention group will be scheduled for an appointment with a primary care doctor. There is the possibility that this visit may result in a charge/cost for the patient. Under the Affordable Care Act, commercial health plans are required to cover an annual Preventive Health Exam at no cost to the patient (no co-payment, co-insurance or deductible). MassHealth also covers these visits without cost-sharing. However, if this visit turns into a “sick” or “disease management” visit, the patient may be billed for some or all aspects of the services provided, depending on their insurer. We will provide a link to the standard MGH information sheet on billing and charges for health care maintenance and directions for patients to contact their insurers prior to a visit to inquire about potential cost-sharing and/or deductibles for their visit. Patients will also be advised that some clinics may penalize individuals for not showing to a scheduled appointment (“no show” fee) and be given opportunities to request the appointment be canceled with each appointment reminder.

6. Benefits

Participants in the control group will receive information on the importance of postpartum care and transitioning to primary care after their delivery.

Participants in the intervention group will receive a bundle of interventions designed to increase attendance at primary care visits and facilitate the transition of care after their delivery.

The goal and potential benefits to the subjects in this trial is to increase patient engagement and connection with their primary care provider, receive recommended health screenings and directed counseling, and reduce unscheduled or urgent visits in the postpartum period.

7. Statistical Analysis

Statistical Methods
Analyses will be performed according to the intention-to-treat principle.

Standard independent, two-sided, two-group comparison testing will be used to compare baseline characteristics between the two groups (chi squared tests, t tests, Wilcoxon rank sum tests, when appropriate).

The primary outcome will be attendance rates at PCP visit within 4 months after delivery, which will be compared between the groups using chi squared tests. Relative risks and 95% confidence intervals will be reported.

Secondary outcomes will include measures of long-term health and health care use after the postpartum period.

Subgroup analyses will be performed by a variety of patient characteristics including gestational age at enrollment, prenatal risk factors, morbidity types, patient race-ethnicity, payer, and enrollment location.

P-value of less than 0.05 will be considered to indicate statistical significance.

Power Calculation
Most study calculations were estimated from the MGH 2020 delivery population. The rates of PCP follow-up were estimated from a randomly selected cohort of 50 patients who met the inclusion criteria. We plan to recruit patients into the study for a period of 4 months and expect 1200 unique patients to be at the targeted gestational age during this period. Among these, we expect 88% to already have an assigned PCP in the network, leaving roughly 1,056 patients. Among these, we estimate that 40% have at least one of the targeted health conditions, leaving a target study population of 422 over the 4-month period. Based on previous studies conducted at MGH, we expect an 85% willingness to participate in the research, leaving an expected recruited population of 360 individuals. Based on our record extraction, we estimate that 33% of the targeted study population has a primary care visit within 4 months of delivery. Assuming an alpha of 0.05 and a baseline mean of 33%, with this expected sample size and power of 80%, our study has a minimum detectable effect size of roughly 15 percentage points (from 33% to 48%). A previous study found that default scheduling into postpartum care appointments (with an OBGYN, not a PCP) increased postpartum care take-up by 24 percentage points; since our intervention incorporates defaults and other activating interventions, an MDE of 15 percentage points is reasonable.

8. Monitoring and Quality Assurance

This is a minimal risk study in which the intervention involves default scheduling of appointments and patient messaging. Adverse events are not expected, and there is no physiologic plausibility for this intervention to cause any NIH-defined serious adverse events (e.g., death, prolonged hospitalization, significant disability).

No interim analyses are planned.

Adverse events will be defined and classified in accordance with NIH guidelines:

Definition of Adverse Events (AE): Any untoward or unfavorable medical occurrence in a human subject, including any abnormal sign, symptom, or disease, temporally associated with the subject's participation in the research, whether considered related to the subject's participation in the research or not.

Definition of Serious Adverse Events (SAE): Any AE that (1) results in death, (2) is life-threatening, (3) results in inpatient hospitalization or prolongation of existing hospitalization, (4) results in persistent or significant disability/incapacity, (5) results in a congenital anomaly/birth defect, and/or (6) may jeopardize the subject's health and may require medical or surgical intervention to prevent one of the other five outcomes listed here.

In the unlikely event an AE or SAE occurs, it will be brought to the PI's attention, and the PI will classify the AE/SAE by severity, expectedness, and relatedness,as listed above. All events that are both serious and unexpected will be reported to Mass General Brigham's IRB, the NIA PO, and to the NIA Roybal DSMB within 48 hours of the research team's knowledge of the SAE. The summary of all other SAEs will be reported to the NIA and to the DSMB quarterly unless otherwise specified by the DSMB. Any unanticipated problem, defined as an issue related to the research suggesting the research places participants or others at greater risk than expected, will be reported to the IRB, the NIA PO, and to the Roybal DSMB within 48 hours of discovery. If the problem involves death then reporting will occur within 24 hours, and this report will include a plan to correct the problem and prevent its occurrence. Any breach of PHI will be reported to the PI, who will report to the IRB and NIA PO within 24 hours of discovery.

The Roybal DSMB oversight is provided by the Standing Roybal DSMB, which includes the members listed: Andrea B. Troxel, ScD (chair); Abby King, PhD; Jerry Gurwitz, MD;Hae-Ra Han, PhD, RN, FAAN; Hang Lee, PhD; Ezra Golberstein, PhD; David Kim, MD PhD; Christopher Celano, MD.

DSMB members will have no direct involvement with the study or conflict of interest with the investigators or institutions conducting the study. Each member has signed a COI statement which includes current affiliations, if any, with pharmaceutical or biotechnology companies (e.g., stockholder, consultant), and any other relationship that could be perceived as a conflict of interest related to the study and/or associatedwith commercial interests pertinent to study objectives.

Data presented to the DSMB will be deidentified as to protect individual participants' privacy and health information. Should the identity of a deidentified subject need to be revealed, the DSMB request will be reviewed and ultimately at the discretion of the Mass General Brigham IRB.

9. Privacy and Confidentiality

☒ Study procedures will be conducted in a private setting
☒ Only data and/or specimens necessary for the conduct of the study will be collected

☒ Data collected (paper and/or electronic) will be maintained in a secure location with appropriate protections such as password protection, encryption, physical security measures (locked files/areas)
☐ Specimens collected will be maintained in a secure location with appropriate protections (e.g. locked storage spaces, laboratory areas)
☒ Data and specimens will only be shared with individuals who are members of the IRB-approved research team or approved for sharing as described in this IRB protocol
☒ Data and/or specimens requiring transportation from one location or electronic space to another will be transported only in a secure manner (e.g. encrypted files, password protection, using chain-of-custody procedures, etc.)
☒ All electronic communication with participants will comply with Mass General Brigham secure communication policies
☒ Identifiers will be coded or removed as soon as feasible and access to files linking identifiers with coded data or specimens will be limited to the minimal necessary members of the research team required to conduct the research
☒ All staff are trained on and will follow the Mass General Brigham policies and procedures for maintaining appropriate confidentiality of research data and specimens
☒ The PI will ensure that all staff implement and follow any Research Information Service Office (RISO) requirements for this research
☒ Additional privacy and/or confidentiality protections

The following procedures (as provided by MGB RISO) will be used for sending SMS messages via Google Voice:

- Google Account:
    - A separate account should be created just for the purpose of the effort/study (in other words, personal Gmail accounts should not be used)
    - For the Google Voice portal, make sure you are not using the same password as your MGB account and ensure the password is strong. (Minimum of 8 characters, alphanumeric, uppercase, lowercase, special character).
        - Two-factor authentication must be enabled
    - Google account will not be shared
    - The Google account used for Google Voice should not be used for emailing or using any other Google Service (i.e., YouTube, Calendar, Contacts, etc.)
    - No credit cards should be added to the Google Account
    - The Google account must be deleted at the end of the study / project
- Participants will be informed not to send personal or health related information via text
- Siri will not be integrated with Google Voice
- Text messages will not address participants by their first name
- Text messages will be sent through the McLean email within the Google account that is created
- Only phone numbers and a unique subject ID will be stored in Google Voice
    - Log records should be deleted manually after 30 days
- No PHI or sensitive information will be communicated via text message
    - Content will not include anything where a healthcare condition or diagnosis can be inferred
- Text message history should be deleted from Google Voice account when no longer needed (within 30 days)

- Study staff will track opt-out requests and delete phone numbers from Google Voice as necessary
- Study staff must not communicate with participants via a group text message
- Access to the portal and overall research must be done from systems that meet MRB RISO compliance requirements; encryption, MobileIron (if Smartphone/Tablet), up to date malware protection, Crowdstrike.
    - https://rc.partners.org/security/secure-your-computer
- Participants:
    - Participants will be texted only if they consent
    - Participants should be informed to delete text messages when no longer needed and hide text push notifications

12. References

1. Boersma P. Prevalence of Multiple Chronic Conditions Among US Adults, 2018. *Prev Chronic Dis*. 2020;17. doi:10.5888/pcd17.200130
2. Home page | United States Preventive Services Taskforce. Accessed December 15, 2021. https://www.uspreventiveservicestaskforce.org/uspstf/
3. Blewett LA, Johnson PJ, Lee B, Scal PB. When a usual source of care and usual provider matter: adult prevention and screening services. *J Gen Intern Med*. 2008;23(9):1354-1360. doi:10.1007/s11606-008-0659-0
4. O'Malley AS, Mandelblatt J, Gold K, Cagney KA, Kerner J. Continuity of care and the use of breast and cervical cancer screening services in a multiethnic community. *Arch Intern Med*. 1997;157(13):1462-1470.
5. DeVoe JE, Saultz JW, Krois L, Tillotson CJ. A medical home versus temporary housing: the importance of a stable usual source of care. *Pediatrics*. 2009;124(5):1363-1371. doi:10.1542/peds.2008-3141
6. Atlas SJ, Grant RW, Ferris TG, Chang Y, Barry MJ. Patient-physician connectedness and quality of primary care. *Ann Intern Med*. 2009;150(5):325-335. doi:10.7326/0003-4819-150-5-200903030-00008
7. Levine DM, Linder JA, Landon BE. Characteristics of Americans With Primary Care and Changes Over Time, 2002-2015. *JAMA Internal Medicine*. 2020;180(3):463-466. doi:10.1001/jamainternmed.2019.6282
8. NVSS - Birth Data. Published June 14, 2021. Accessed September 1, 2021. https://www.cdc.gov/nchs/nvss/births.htm
9. Prenatal care and tests | Office on Women's Health. Accessed December 15, 2021. https://www.womenshealth.gov/pregnancy/youre-pregnant-now-what/prenatal-care-and-tests
10. Centers for Disease Control and Prevention (CDC). Adult Obesity Facts. Published August 29, 2017. Accessed February 14, 2018. https://www.cdc.gov/obesity/data/adult.html
11. Mental Illness. National Institute of Mental Health (NIMH). Accessed December 17, 2021. https://www.nimh.nih.gov/health/statistics/mental-illness
12. Products - Data Briefs - Number 289 - October 2017. Published June 6, 2019. Accessed December 17, 2021. https://www.cdc.gov/nchs/products/databriefs/db289.htm
13. National Diabetes Statistics Report 2020. Estimates of diabetes and its burden in the United States. Published online 2020:32.
14. Sattar N, Greer IA. Pregnancy complications and maternal cardiovascular risk: opportunities for intervention and screening? *BMJ*. 2002;325(7356):157-160. doi:10.1136/bmj.325.7356.157
15. Kim C, Tabaei BP, Burke R, et al. Missed Opportunities for Type 2 Diabetes Mellitus Screening Among Women With a History of Gestational Diabetes Mellitus. *Am J Public Health*. 2006;96(9):1643-1648. doi:10.2105/AJPH.2005.065722
16. Media BKK. Publications & Guidelines | SMFM.org - The Society for Maternal-Fetal Medicine. Accessed October 18, 2021. https://www.smfm.org/publications/225-smfm-statement-implementation-of-the-use-of-antenatal-corticosteroids-in-the-late-preterm-birth-period-in-women-at-risk-for-preterm-delivery
17. Yee LM, Simon MA, Grobman WA, Rajan PV. Pregnancy as a "golden opportunity" for patient activation and engagement. *American Journal of Obstetrics & Gynecology*. 2021;224(1):116-118. doi:10.1016/j.ajog.2020.09.024

18. Cohen JL, Daw JR. Postpartum Cliffs—Missed Opportunities to Promote Maternal Health in the United States. *JAMA Health Forum*. 2021;2(12):e214164. doi:10.1001/jamahealthforum.2021.4164
19. Milkman KL, Patel MS, Gandhi L, et al. A megastudy of text-based nudges encouraging patients to get vaccinated at an upcoming doctor's appointment. *PNAS*. 2021;118(20). doi:10.1073/pnas.2101165118
20. Patel MS, Volpp KG, Asch DA. Nudge Units to Improve the Delivery of Health Care. *N Engl J Med*. 2018;378(3):214-216. doi:10.1056/NEJMp1712984
21. Thaler RH, Sunstein CR. *Nudge: The Final Edition*. Revised edition. Penguin Books; 2021.
22. Henderson JT, Vesco KK, Senger CA, Thomas RG, Redmond N. Aspirin Use to Prevent Preeclampsia and Related Morbidity and Mortality: Updated Evidence Report and Systematic Review for the US Preventive Services Task Force. *JAMA*. 2021;326(12):1192. doi:10.1001/jama.2021.8551

Roybal Centers Program DSMB Charter

The Roybal Data and Safety Monitoring Board (DSMB) will act in an advisory capacity to the National Institute of Aging (NIA) Director to monitor participant safety, data quality and progress of the Roybal Centers. The Roybal DSMB would review and monitor pilot clinical trials that are determined by NIA to require DSMB oversight, and that are supported through the:

- Roybal Centers for Translational Research on Aging (P30 Center Core Grants, under RFA-AG-19-006),
- Roybal Centers for Translational Research on Dementia Care Provider Support (P30 Center Core Grants, RFA-AG-19-007), and
- Edward R. Roybal Coordinating Center (R24 Resource-Related Research Projects, RFA-AG-19-008).

DSMB Responsibilities

- Prior to each DSMB meeting, DSMB members will confirm that no conflict of interest exists with investigators or key personnel of the pilot trials that will be discussed. Interests that may create a potential conflict of interest should be disclosed to the DSMB prior to any discussion. The DSMB will determine how to handle any such potential conflict. The DSMB can require that a member with a potential conflict be present during discussions but abstain from voting or take other means deemed appropriate. NIA or DSMB Chair may request a member of the DSMB to leave the meeting in the event of unmanageable potential conflict or appearance of conflict. At the beginning of every DSMB meeting, DSMB members will reconfirm that no conflict of interests exists.

- Review the entire IRB-approved study protocols and the MOP, with regard to participant safety, recruitment, randomization, intervention, data management, quality control and analysis and the informed consent documents.

- Recommend changes to the protocol and the informed consent form, when applicable.

- Identify the relevant data parameters and the format of the information to be regularly reported.

- Recommend participant recruitment be initiated after receipt of a satisfactory protocol. If the need for modifications to the protocol, the MOP, consent form, DSMP or any other study document is indicated by the DSMB and/or the NIA Program Officer (PO), the DSMB will postpone its recommendation for the initiation of participant recruitment until after the receipt of a satisfactory revised protocol(s) or other study documents.

- Presentations by PIs for pilot trials will be limited to 20 min, including questions and answer from DSMB members. Pilot PI will provide overview of the study; data and safety related issues and presentation will be limited to 15 min. At the discretion of the Chair, discussions may continue.

- Review periodic (safety) reports from Safety Officers, as needed.

- Review masked and unmasked data. These data can be related to safety, recruitment, randomization, retention, protocol adherence, trial operations, data completeness, form completion, intervention effects, gender and minority inclusion.

- Identify needs for additional data relevant to safety issues and request these data from the study investigators.

- Propose additional analyses and periodically review developing data on safety and endpoints.

- At each meeting, consider the rationale for continuation of the study, with respect to progress of randomization, retention, protocol adherence, data management, safety issues, and outcome data (if relevant) and make a recommendation for or against the trial's continuation.

- Review and make recommendations on proposed protocol changes, and/or new protocols proposed during the trial. When the DSMBs are unblinded, the Boards may recommend to NIA to appoint a blinded working group of the DSMB to review the proposed protocol changes and make recommendations to NIA on whether to approve the requests.

- Provide advice on issues regarding data discrepancies found by the data auditing system or other sources.

- Review manuscripts of trial results if requested by the Board or the NIA PO who may seek DSMB review of manuscripts reporting major outcomes prior to their submission for publication.

The DSMB will discharge itself from its duties when the study is complete.

Membership

Membership consists of persons completely independent of the investigators who have no financial, scientific, or other conflict of interest with the trial. Collaborators or associates of the PI are not eligible to serve on the DSMB. Written documentation attesting to absence of conflict of interest is required and will be collected by NIA. This DSMB will consist of [insert number] members having been approved by the Director of NIA.

The DSMB includes experts in or representatives of the fields of:

- Geriatrics
- Dementia
- Psychiatry
- Health policy
- Psychology
- Biostatistics

Dr. Andrea B. Troxel has been selected by NIA to serve as the Chairperson and is responsible for facilitating the meetings, reviewing the first draft of the meeting notes with the NIA Program Official and any decision making in the case of a tie vote. The Chair and NIA Program Officer are the contact people for the DSMB. The Feinstein Institute for Medical Research will provide the logistical management and support for the DSMB.

Meeting Format

Meetings of the DSMB will be held regularly (e.g., every six to nine months) at the call of NIA or the DSMB Chair. The NIA Program Officer or designee (NIA staff) will be present at every meeting. An emergency meeting of the DSMB may be called at any time by the Chair or the NIA, should participant safety questions or other unanticipated problems arise. The DSMB should discuss the definition of a quorum.

DSMB meetings will consist of open, closed and optional executive sessions, all closed to the public because discussions may address confidential participant data. The study PI and key staff members, DSMB members and NIA Program Officer and/or authorized NIA staff attend the **open sessions**. Discussions at these sessions focus on the review of the aggregate data, conduct and progress of the study, including participant accrual, protocol compliance, and problems encountered. Data by treatment group are not presented in the open session.

The **closed session** will be attended by unblinded study staff, the DSMB members and the NIA PO or designee(s). The NIA PO attends the closed and open sessions as an observer, not as a DSMB member, to answer any policy or administrative questions the DSMB members may have. The primary objective of the closed sessions is to review data by study group. To ensure participants safety and well-being, DSMBs for NIA-funded trials are required to review safety data by the actual treatment group. In many instances, safety data could also be the outcome data. Therefore, the unblinded Boards no longer review and provide recommendations to NIA on any, but safety-related protocol changes. All other protocol modifications are subject to review by the blinded working groups of the DSMBs. DSMBs' working groups are appointed by NIA and provide their recommendations to NIA Director who makes decisions about whether to approve or decline proposed modifications.

If necessary, an **executive session** may be requested by the DSMB and will be attended only by voting DSMB members. The NIA Program Officer or designee is not permitted to attend the executive sessions.

The NIA PO, DSMB Chair or the Principal Investigator will prepare the meeting agenda that usually includes the following:

1. Welcome and introduction – study team, DSMB members, NIA and KAI staff
2. Open session (review study protocol and its amendments, consent form, open study report, etc.) - study team, DSMB members, NIA and KAI staff
3. Closed session (review closed session report, including unblinded data, etc.) – unblinded study statistician, DSMB members, NIA and KAI staff
4. Executive session (optional, upon DSMB request) – DSMB members only
5. Debriefing (optional, upon DSMB request, time permitting) - study team, DSMB members, NIA and KAI staff

The DSMB may modify its processes and procedures at any time with the approval of the NIA POs. Pilot studies to be presented to the DSMB for review would be determined by NIA, annually, through an internal process that culminates in a meeting for Roybal studies.

Meeting Materials

DSMB interim report templates developed by the study staff or study-specific versions of NIA report templates for both the open and closed sessions and plans for interim analyses will be reviewed and either approved at the initial DSMB meeting or changes requested. Upon DSMB

request and approval by NIA, reports could be modified at any time during the study. All meeting materials should be sent by email to NIA at least 7 to 10 calendar days prior to the meeting. NIA PO will send the reports to the DSMB.

Part 1 - Open Session Reports (Template for Single Site - Open; Template for Multi Site - Open): Open session reports will include administrative reports that describe participants screened, enrolled, completed, and discontinued, as well as baseline characteristics of the study population. Other general information on study status may also be presented. Listings of adverse events and serious adverse events as well as any other information requested by the DSMB may also be in the open session report, but none of the data will be presented in an unblinded manner.

Part 2 – Closed Session Report (Template for Single Site - Closed; Template for Multi Site - Closed): Closed session reports will present the same information as presented in the open session but by unblinded treatment group.

Reports from the DSMB

A report containing the recommendations for continuation or modification of the study will be prepared by the DSMB, NIA Project Officer or NIA contractors. The draft report will be sent to the DSMB members for review and approval not later than three weeks after the meeting. Once approved by the DSMB members, the Program Official will forward the DSMB recommendations to the Principal Investigator indicating NIA's concurrence with the report or its parts. It is the responsibility of the Principal Investigator to distribute the DSMB recommendation to all co-investigators and to ensure that copies are submitted to the IRB that reviewed and approved the study documents.

As it stated above, each meeting must include a recommendation to continue the study made by a formal DSMB majority or unanimous vote. Should the DSMB decide to issue a termination recommendation, the full vote of the DSMB is required. In the event of a split vote, majority vote will rule and a minority report should be appended. The DSMB Chair provides the tiebreaking vote in the event of a 50-50 split vote.

A recommendation to terminate the study may be made by the DSMB at any time by majority vote. If this recommendation was made during the DSMB's Executive session, the Chair should provide notify the NIA immediately by telephone and email. After the NIA Director makes a decision about whether to accept or decline the DSMB recommendation to terminate the study, NIA PO informs the PI about the decision.

Confidentiality

All materials, discussions, and proceedings of the DSMB are completely confidential. Members and other participants in DSMB meetings are expected to maintain confidentiality.

DATA & SAFETY MONITORING PLAN (DSMP)

Amy Finkelstein

P30 AG064190

MIT Roybal Center for Translational Research to Improve Healthcare for the Aging

Project PIs: Mark Clapp, MD, MPH; Jessica Cohen, PhD

Bridging the Gap to Primary Care: A Behavioral Science Informed Intervention to Improve Chronic Disease Management among Postpartum Women

Brief Description of Intervention: We aim to evaluate the efficacy of a behavioral-science inspired intervention bundle (patient-tailored health information, automatic scheduling of PCP appointment after delivery, and appointment reminder nudges) to increase patient attendance at a routine health care maintenance appointment within 4 months of delivery for women with obesity, diabetes, hypertension, and/or a mental health condition. We will conduct a randomized controlled trial comparing this intervention bundle to the receipt of generic information on the importance of primary care follow-up after delivery.

Brief Description of Project Design

360 pregnant or recently postpartum patients with pre-existing or newly diagnosed health condition (hypertension, diabetes, obesity, mood disorder) will be randomly assigned to one of two interventions:

1. Routine care
2. Routine care + a bundle of behavioral-based interventions intended to improve a patient’s transition from postpartum to primary care

Stage of Behavioral Intervention Development Stage II (traditional efficacy testing)

NIH Phase III Clinical Trial? No

Note: An NIH-defined Phase III clinical trial is a broadly based prospective clinical investigation, usually involving several hundred or more human subjects, for the purpose of evaluating an experimental intervention in comparison with a standard or control intervention or comparing two or more existing treatments.

Multiple Site Trial? *No*

List of Specific Aims

1. Test the efficacy of a behavioral science inspired intervention bundle to increase patient attendance within 4 months of delivery for women with chronic conditions.
2. Test the efficacy of the intervention bundle to improve receipt of tailored health screenings.
3. Test the efficacy of the intervention bundle to reduce unscheduled or urgent encounters within the health system.

TABLE OF CONTENTS

Page

1.0 PARTICIPANT SAFETY 1
1.1 Potential Risks and Benefits for Participants 1
1.2 Adverse Event and Serious Adverse Event Collection and Reporting 1
1.3 Protection Against Study Risks 1
2.0 INTERIM ANALYSIS 4
3.0 DATA AND SAFETY MONITORING 4
3.1 Frequency of Data and Safety Monitoring 4
3.2 Data Analysis and Coordination 4
3.3 Content of Data and Safety Monitoring Report 4
3.4 DSMB Membership and Affiliation 5
3.5 Conflict of Interest for DSMB's 5
3.6 Protection of Confidentiality 5
3.7 DSMB Responsibilities 5

1.0 PARTICIPANT SAFETY

1.1 Potential Risks and Benefits for Participants

Potential Risks:

There are minimal risks to participants. Those in the intervention arm will receive condition-specific information on pregnancy comorbidities and their implications on their long-term health. This information may cause anxiety or worry among patients; however, this same information is routinely reviewed by obstetrics providers at visits. Furthermore, individuals in the intervention group will be scheduled for an appointment with a primary care doctor. There is the possibility that this visit may result in a charge/cost for the patient. Under the Affordable Care Act, commercial health plans are required to cover an annual Preventive Health Exam at no cost to the patient (no co-payment, co-insurance or deductible). MassHealth also covers these visits without cost-sharing. However, if this visit turns into a “sick” or “disease management” visit, the patient may be billed for some or all aspects of the services provided, depending on their insurer.

Potential Benefits:

Participants in the control group will receive information on the importance of postpartum care and transitioning to primary care after their delivery.

Participants in the intervention group will receive a bundle of interventions designed to increase attendance at primary care visits and facilitate the transition of care after their delivery.

The goal and potential benefits to the subjects in this trial is to increase patient engagement and connection with their primary care provider, receive recommended health screenings and directed counseling, and reduce unscheduled or urgent visits in the postpartum period.

1.2 Adverse Event and Serious Adverse Event Collection and Reporting

This is a minimal-risk study in which the intervention involves default scheduling of appointments and patient messaging. Adverse events are not expected, and there is no physiologic plausibility for this intervention to cause any NIH-defined serious adverse events (e.g., death, prolonged hospitalization, significant disability).

Adverse events will be defined and classified in accordance with NIH guidelines:

1.2.1 Adverse Event (AE)/Serious Adverse Event (SAE) Definitions

Definition of Adverse Events: Any untoward or unfavorable medical occurrence in a human subject, including any abnormal sign, symptom, or

disease, temporally associated with the subject's participation in the research, whether considered related to the subject's participation in the research or not.

Definition of Serious Adverse Events: Any AE that (1) results in death, (2) is life-threatening, (3) results in inpatient hospitalization or prolongation of existing hospitalization, (4) results in persistent or significant disability/incapacity, (5) results in a congenital anomaly/birth defect, and/or (6) may jeopardize the subject's health and may require medical or surgical intervention to prevent one of the other five outcomes listed here.

1.2.2 Grading Scale

Severity
Mild: Awareness of signs or symptoms, but easily tolerated and of minor irritation, causing no loss of time from normal activities. Symptoms do not require therapy or a medical evaluation; signs and symptoms are transient.
Moderate: Events introduce a low level of inconvenience or concern to the participant and may interfere with daily activities, but are usually improved by simple therapeutic measures.
Severe: Events interrupt the participant's normal daily activities and generally require systemic drug therapy or other treatment. They are usually incapacitating.

Expectedness
Unexpected: nature or severity of the event is not consistent with information about the condition under study or intervention in the protocol or consent form.
Expected: event is known to be associated with the intervention or condition under study. Because the intervention being delivered is informational in nature, there are no expected AEs or SAEs for this study.

1.2.3. Study Relatedness

Definitely Related: The adverse event is clearly related to the intervention- i.e. an event that follows a reasonable temporal sequence from administration of the intervention, follows a known or expected response pattern to the suspected intervention, that is confirmed by improvement on stopping and reappearance of the event on repeated exposure and that could not be reasonably explained by the known characteristics of the subject's clinical state.
Possibly Related: An adverse event that follows a reasonable temporal sequence from administration of the study intervention, follows a known or expected response pattern to the suspected intervention, but that could readily have been produced by a number of other factors.
Not Related: The adverse event is clearly not related to the intervention- i.e. another cause of the event is most plausible; and/or a plausible temporal sequence is inconsistent with the onset of the event and the study intervention and/or causal relationships is considered implausible.

1.2.4. Timeline for Reporting

In the unlikely event an AE or SAE occurs, it will be brought to the PI's attention and the PI will classify the AE/SAE by severity, expectedness, and relatedness, as listed above. All events that are both serious and unexpected will be reported to Mass General Brigham's IRB, the NIA PO, and to the DSMB within 48 hours of the research team's knowledge of the SAE. The summary of all other SAEs will be reported to the NIA and to the DSMB quarterly unless otherwise specified by the DSMB. Any unanticipated problem, defined as an issue related to the research suggesting the research places participants or others at greater risk than expected, will be reported to the IRB, the NIA PO, and to the DSMB within 48 hours of discovery. If the problem involves death then reporting will occur within 24 hours, and this report will include a plan to correct the problem and prevent its occurrence. Any breach of PHI will be reported to the PI, who will report to the IRB and NIA PO within 24 hours of discovery

1.3 Protection Against Study Risks

Informed Consent Process

Eligible patients who are interested in the study may agree to meet with a study staff member, who will describe the study, answer questions, and obtain written informed consent from eligible patients who elect to participate. The consent form will include information about the purpose of the study, the nature of the subject's participation, the possible risks and discomforts associated with participation, the potential benefits of participation, a statement of the voluntary nature of participation, and a description of the mechanisms used to ensure confidentiality. All prospective subjects will be provided with an informed consent document written at the 8th-grade reading level in English and Spanish. The study staff also will provide a scripted oral form of the informed consent document and, to ensure comprehension, will ask prospective subjects to restate the main purposes and risks of the study, as well as restate that their access to ongoing care will not be influenced by their decision about participation. Individuals who cannot accurately restate these three elements of consent will not be enrolled, regardless of their interest.

Protection Against Risks

Health information collected as part of this study will be stored in REDCap. No identifiable data will be stored or downloaded on any personal or unauthorized computers. Study staff will access the data on institutionally purchased and managed computers that operate behind the health system's security and firewall protections. Only the study MGH study staff will have access to the identifiable data set. Once the study is completed, data will be deidentified such that it can be analyzed without risk of a breach of privacy or confidentiality. Any data that is shared outside of MGH will require a data use authorization.

Although we will make every effort to protect participant privacy and confidentiality, it is possible that participants' involvement in the study could become known to others. For those in the intervention group, we will be sending messages via text or the patient portal (per subject request); there is the possibility that study participation or health information could become known to others despite the use of firewalls, password protection, and other security measures.

For those in the intervention group, we will provide information about the possibility that their scheduled primary care visit may have an associated charge. We will provide the standard MGH information sheet on billing and charges for health care maintenance and directions for patients to contact their insurers prior to a visit to inquire about potential cost-sharing and/or deductibles for their visit.

2.0 INTERIM ANALYSIS

No interim analysis is planned as this study is no more than minimal risk for participants.

3.0 DATA SAFETY AND MONITORING

The Principal Investigator (PI), with the assistance of the research study staff, is responsible for ensuring participants' safety and ensuring the study is implemented in accordance with the approved IRB protocol and procedures. The study protocol and DSMP will be reviewed by the NIA Roybal Center Data Safety Monitoring Board (DSMB), who will make recommendations regarding the modification, continuation, or conclusion of the trial.

3.6 Frequency of Data and Safety Monitoring

Any AEs or SAEs will be dealt with as described in section above. The frequency of additional monitoring, including the submission of safety reports, will be determined by the DSMB and safety officer (if applicable).

3.7 Data Analysis and Coordination

The PIs and research assistant will be responsible for data processing and analysis. All data will be directly available to the DSMB and Program Officer on request, except raw data identifying individual participants. Any data sharing requests of patient information outside the research team and the approved IRB protocol will be facilitated through the Mass General Brigham Office of Research Management.

3.8 Content of Data and Safety Monitoring Report

We anticipate the content of the data and safety monitoring report will include study progress, recruitment and retention status, and safety information.

3.9 DSMB Membership and Affiliation

DSMB oversight is provided by the Standing Roybal DSMB, which includes the members listed: Andrea B. Troxel, ScD (chair); Abby King, PhD; Jerry Gurwitz, MD; Hae-Ra Han, PhD, RN, FAAN; Hang Lee, PhD; Ezra Golberstein, PhD; David Kim, MD PhD; Christopher Celano, MD.

3.10 Conflict of Interest for DSMB/SO

DSMB members will have no direct involvement with the study or conflict of interest with the investigators or institutions conducting the study. Each member has signed a COI statement which includes current affiliations, if any, with pharmaceutical or biotechnology companies (e.g., stockholder, consultant), and any other relationship that could be perceived as a conflict of interest related to the study and/or associated with commercial interests pertinent to study objectives.

3.6 Protection of Confidentiality

Data presented to the DSMB will be deidentified as to protect individual participant's privacy and health information. Should the identify of a deidentified subject need to be revealed, the DSMB request will be reviewed and processed by the Mass General Brigham IRB.

3.7 DSMB/SO Responsibilities

Please see the DSMB charter for a description of DSMB responsibilities.

Bridging the Gap from Postpartum to Primary Care: A Behavioral Science Informed Intervention to Improve Chronic Disease Management among Postpartum Women

Study PIs: Mark Clapp, MD MPH (MGH); Jessica Cohen, PhD (Harvard)
Site PI: Mark Clapp, MD MPH (MGH)

Eligibility Screening Variable List

- Name
- Medical Record Number
- Age
- Estimated due date
- Primary OB provider
- Primary prenatal care clinic location
- Pregnancy history (gravidity/parity)
- Pre-pregnancy comorbidities:
    - Obesity (BMI)
    - Hypertension
    - Diabetes
    - Anxiety
    - Depression
- Pregnancy-related conditions:
    - Hypertension
    - Diabetes
    - Anxiety
    - Depression
- Risk factors for hypertensive disorders of pregnancy
    - Chronic kidney disease
    - Lupus / autoimmune disease
    - Multifetal gestation
    - History of pre-eclampsia
    - Aspirin 81 mg use (as proxy for provider recognition/designation for elevated risk)
- Race/ethnicity
- Primary/preferred language
- Primary insurer/payer
- Patient Gateway enrollment status

Bridging the Gap from Postpartum to Primary Care: A Behavioral Science Informed Intervention to Improve Chronic Disease Management among Postpartum Women

Study PIs: Mark Clapp, MD MPH (MGH); Jessica Cohen, PhD (Harvard)
Site PI: Mark Clapp, MD MPH (MGH)

Baseline Survey

SECTION 1. Interview Information

101. RA ID number
102: Enrollment Facility ID number
103: Facility name
104: Date of interview (MM/DD/YY)
105: Start time of interview
106: End time of interview
107: Time of interview in relation to patient's prenatal/postnatal visit:<br>1=Before visit<br>2=After visit<br>3=Partly before and partly after visit

SECTION 2. Consent

Instructions: please make sure to complete this section for every individual approached.

201. Respondent First Name
202: Respondent Last Name
203: Respondent Medical Record Number
204: Respondent OBGYN/CNM Last Name:
205: Respondent Primary OBGYN Practice Location:
206: Respondent EDD
208: Date respondent was sent Patient Gateway Research Letter (MM/DD/YY)
209: Is interview date at least two weeks after Patient Gateway Research Letter was sent?<br>1= Yes<br>2= No ->STOP, End Survey
210: READ OUT LOUD TO PATIENT: May we have your permission to explain why we are here today, and to ask you some questions?<br>1= Yes<br>2= No ->STOP, if respondent would not like to participate: End Survey.
211: REVIEW INFORMED CONSENTS and indicate whether consent was granted.<br>1 = Consented → Skip to next section<br>2 = Did not consent → STOP, End Survey.

SECTION 3. Verification of Respondent Demographic Information
Pre-fill this section based on Patient Record and ask patient to verify or update this information.

301.	Respondent family name:
302.	Respondent first name:
303.	Respondent mobile number:
304.	If consented to SMS messagint, respondent's ability to receive SMS messages:
305.	Respondent home address:
306.	Respondent email address:
307.	Respondent race:
308.	Respondent ethnicity:
309.	Respondent Preferred Language:
310.	Respondent Date of Birth:
311.	Primary Care Provider Practice:
312.	Primary Care Provider Name:

SECTION 4. Demographic Information and Plans for Postpartum Leave

401. How many children have you had (not including your current pregnancy)?<br>Number: ________<br>Refused -99
402. How many people (not including yourself) currently live in your household?<br>Number: ___________<br>Don't know -88<br>Refused -99
403. What is your marital status?<br>1. Married<br>2. Not married<br>Refused -99
404. Are you currently cohabitating with a spouse or partner?<br>1. Yes<br>2. No<br>Refused -99
405. At any time during your pregnancy did you work for pay in a job located in Massachusetts?<br>1. Yes<br>2. No, I worked for pay outside of MA<br>3. No<br>Refused -99
406. Which of the below best describes the type of work you had? (Tick all that apply. List name of employer)<br>1. Federal or State Government<br>2. Private or For-Profit Company<br>3. Municipality (school department, public works, housing authority, regional school district)<br>4. Church or Religious Organization<br>5. Self-employed<br>6. I don't know which of these categories by employer falls into.<br>Refused -99
407. In the past 12 months, approximately how much did you earn?<br>1. $0<br>2. $1 - $10,000<br>3. $10,001 – $29,999<br>4. $30,000-$49,999<br>5. $50,000 - $74,999<br>6. $75,000-$100,000<br>7. > $100,000<br>8. Don't know<br>99. Refused
408. What type of leave(s) do you plan on taking after the birth of your baby? (Tick all that

apply)<br>1. Paid leave<br>2. Unpaid leave<br>3. I do not plan on taking leave.<br>8. Don't know<br>99. Refused
409. Approximately how many weeks or months of leave in total do you plan to take after the birth of your baby?<br>________Weeks<br><br>________ Months<br><br>8. Don't know<br>99. Refused
410. Have you ever seen, read or heard anything about a state program called the "Paid Family and Medical Leave" (PFML), which provides eligible workers with paid time off for family or medical reasons?<br>1. Yes<br>2. No<br>99. Refused
411. What do you plan to do for childcare after your baby is 3 months old? (Tick all that apply)<br>1. My partner and/or I will care for the child<br>2. Child care by a family member or friend for free<br>3. Child care by a family member or friend you pay<br>4. Nanny or babysitter<br>5. Private daycare center<br>6. Headstart Daycare Center or Other Free Daycare<br>88. Don't Know<br>99. Refused
412. Do you have access to a car?<br>1. Yes, always<br>2. Yes, sometimes<br>3. No<br>88. Don't know<br>99. Refused
413. What mode of transportation do you usually take to get to medical appointments?<br>1. Personal car<br>2. Taxi, Uber or Lyft<br>3. Bus<br>4. Subway ("T")<br>5. Train<br>6. Bicycle<br>7. On foot<br>88. Don't know<br>99. Refused

414. Aside from money you may earn yourself, do you expect any of the following people to also provide financial support for you and your child/children after the baby is born? (Tick all that apply)<br>1. Baby's father<br>2. Partner (if not baby's father)<br>3. Family<br>4. Friends<br>5. Government Support<br>6. Other<br>88. Don't know<br>99. Refused

Mass General Brigham

[Date]

[Patient name
Address
City, State, Zip]

Dear [Ms. Patient],

We are writing to let you know about a research study that might interest you. Mass General Brigham is committed to providing excellent care to you and to our community. An important part of our mission is to learn new ways to care for our patients by doing research. Our patients play an important role in research by joining studies to help discover better treatments or better ways to prevent health problems.

Mass General Brigham is a health system that includes hospitals, community health centers, and groups of doctors. We work together to provide the best care possible to our community. Patients at Mass General Brigham have access to high-quality care across our entire system. As a healthcare system, we also work together to provide access to research studies to all our patients.

The information below tells you about the study that might interest you. You can learn more or speak with the research team to decide if it is a good match for you.

Study Name: Bridging the Gap from Postpartum to Primary Care

What we are studying: Methods to improve primary care among postpartum women

Who might qualify: Pregnant and postpartum people who may particularly benefit from primary care in the postpartum period.

These may or may not apply to you.

What you are asked to do in the study: Agree to receive occasional messages by the study team via Patient Gateway between the time of enrollment through 4 months postpartum

For more information:

To learn more about THIS study: Contact the study's research coordinator, Fowsia Warsame, at 617-643-5483. After you learn more, you can decide to join or not. We may also find out that you do not qualify for the study. Whether you join the study or not, it will not change the medical care you receive here at Mass General Brigham.

Mass General Brigham

If you are definitely NOT interested in THIS study, and don't want to hear more about it, please call study coordinator Fowsia Warsame at 617-643-5483. If we do not hear back from you within two weeks, we may approach or call you to see if you wish to hear more about the study.

To learn more about research in general: Call the Research Navigator Office: **857-282-5370**

Go to the Rally website: rally.partners.org/research

If you do not want to receive notices about *any* studies at Mass General Brigham:

- Log in to your Patient Gateway account.
  On the Menu scroll down to "Resources" and choose "Research Opportunities"
  Read the brief information and chose the appropriate button.

OR

- Contact the Research Navigator Office: **Call 857-282-5370**

Thank you in advance for considering this research study.

Sincerely,

Mark Clapp, MD MPH
Maternal-Fetal Medicine
Massachusetts General Hospital
617-724-4531

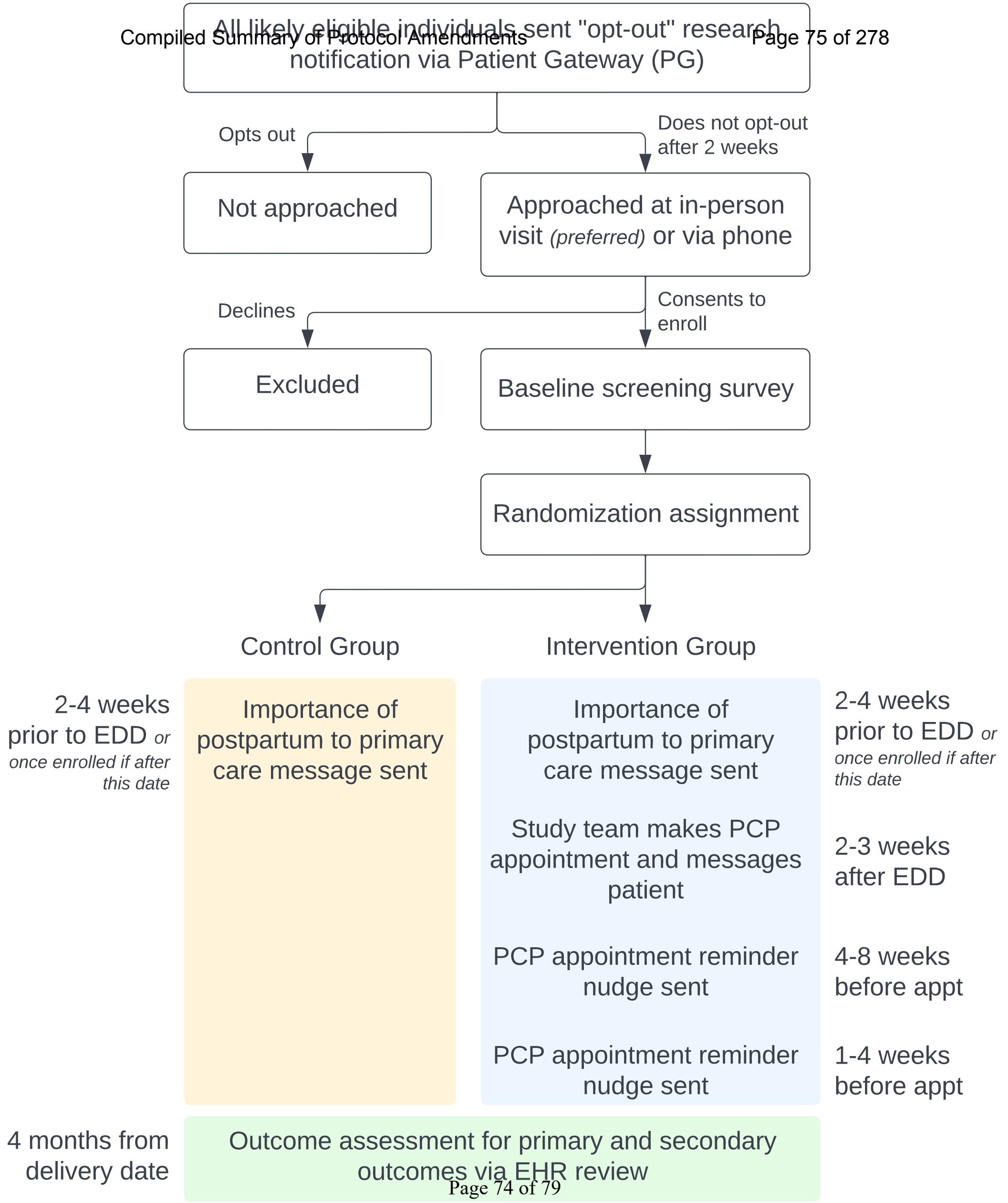
All likely eligible individuals sent "opt-out" research notification via Patient Gateway (PG)
Opts out
Does not opt-out after 2 weeks
Not approached
Approached at in-person visit (preferred) or via phone
Declines
Consents to enroll
Excluded
Baseline screening survey
Randomization assignment
Control Group
Intervention Group
2-4 weeks prior to EDD or once enrolled if after this date
Importance of postpartum to primary care message sent
Importance of postpartum to primary care message sent
2-4 weeks prior to EDD or once enrolled if after this date
Study team makes PCP appointment and messages patient
2-3 weeks after EDD
PCP appointment reminder nudge sent
4-8 weeks before appt
PCP appointment reminder nudge sent
1-4 weeks before appt
4 months from delivery date
Outcome assessment for primary and secondary outcomes via EHR review

Bridging the Gap from Postpartum to Primary Care: A Behavioral Science Informed Intervention to Improve Chronic Disease Management among Postpartum Women

Study PIs: Mark Clapp, MD MPH (MGH); Jessica Cohen, PhD (Harvard)
Site PI: Mark Clapp, MD MPH (MGH)

Patient Message Text

Group	Timing	Type	Message content if PCP appt scheduled	Message content if PCP appt not scheduled
Control	2-4 weeks prior to EDD	PG	N/A	Dear XXX,<br><br>Having a primary care provider (PCP) and seeing them at least once a year is important for your health. These providers can identify health risks and help you improve your health through your entire life.<br><br>These providers have different roles than your obstetrician (pregnancy doctor) or midwife. It is recommended that all patients see their PCP after your pregnancy ends and your relationship with your pregnancy provider has ended (typically 6-12 weeks after your delivery).<br><br>If you have questions about finding or seeing your PCP after your delivery, please ask your pregnancy provider or call your PCP.<br><br>--<br>*This message has been sent from The Bridge Study team. If you wish to no longer receive any messages from this team, please call the study coordinator XX or email the study team at XX. Do not reply directly to this message with questions about your health or to arrange follow-up.*

** EDD, estimated date of delivery; PG, Patient Gateway secure messaging portal.*

Group	Timing	Type	Message content if PCP appt scheduled	Message content if PCP appt not scheduled
Intervention	2-4 weeks prior to EDD	PG	N/A	Dear XXX,<br><br>Having a primary care provider (PCP) and seeing them at least once a year is important for your health. These providers can identify health risks and help you improve your health through your entire life.<br><br>These providers have different roles than your obstetrician (pregnancy doctor) or midwife. It is recommended that all patients see their PCP after your pregnancy ends and your relationship with your pregnancy provider has ended (typically 6-12 weeks after your delivery).<br><br>We will help you schedule an appointment with your PCP!<br><br>If you have preferences for your appointment, please fill out this form [REDCap link]. If not, we will schedule your appointment with the PCP listed in your record within the next few months.<br><br>If you have questions about finding or seeing your PCP after your delivery, please ask your pregnancy provider or call your PCP.<br><br>--<br>*This message has been sent from The Bridge Study team. If you wish to no longer receive any messages from this team, please call the study coordinator XX or email the study team at XX. Do not reply directly to this message with questions about your health or to arrange follow-up.*

Group	Timing	Type	Message content if PCP appt scheduled	Message content if PCP appt not scheduled
Intervention	2 weeks after EDD	PG	An appointment with your primary care provider (PCP) has been specially reserved for you:<br>XXX APPT INFO ##<br><br>This appointment is important to transition your care from your pregnancy provider back to your PCP after your delivery. Your PCP will ensure you have the appropriate screening tests and a plan in place to keep you healthy in the future.<br><br>Specific issues or risks from your pregnancy that you may want to address with your PCP include: XXX<br><br>Often, this “annual exam” is offered at no cost to you, depending on your insurer. For more information on costs, please call your PCP’s office or visit: https://www.massgeneral.org/notices/billing/preventive-health-exams<br><br>If you are unable to make this appointment or wish to reschedule, please call your PCP’s office at XXX or contact XXX with the Bridge Study Team. Please note that some clinics may have penalties for not showing to a scheduled visit.<br><br>--<br>*This message has been sent from The Bridge Study team. If you wish to no longer receive any messages from this team, please call the study coordinator XX or email the study team at XX. Do not reply directly to this message with questions about your health or to arrange follow-up.*	We have been unable to reserve a primary care provider (PCP) appointment for you with XXX at XXX Practice because XXX. Please call your PCP’s office to schedule. We will also call you within the next week to see if we can help.<br><br>The PCP appointment is important to transition your care from your pregnancy provider back to your PCP after your delivery.<br><br>Specific issues or risks from your pregnancy that you may want to address with your provider include: XXX<br><br>Often, this “annual exam” is offered at no cost to you, depending on your insurer. For more information on costs, please visit: https://www.massgeneral.org/notices/billing/preventive-health-exams<br><br>Some conditions that you may be at risk for that may need continued long-term follow-up with your PCP include: XXX. Your PCP will ensure you have the appropriate screening tests and a plan in place to keep you healthy in the future.<br><br>--<br>*This message has been sent from The Bridge Study team. If you wish to no longer receive any messages from this team, please call the study coordinator XX or email the study team at XX. Do not reply directly to this message with questions about your health or to arrange follow-up.*
Intervention	2 weeks after EDD	SMS	An appointment with your PCP has been specially reserved for you! Check Patient Gateway for details. -The Bridge Study Team<br>[do not reply to this message]<br>*character count: 154*	Oh no! We are having trouble arranging your PCP appointment. Check Patient Gateway for details. -The Bridge Study Team<br>[do not reply to this message]<br>*character count: 147*

Group	Timing	Type	Message content if PCP appt scheduled	Message content if PCP appt not scheduled
Intervention	4 weeks before PCP appointment	PG	Dear XXX,<br><br>Your primary care provider (PCP) is looking forward to caring for you going forward after your pregnancy at this upcoming important appointment:<br>XXX APPT INFO ##<br><br>This appointment is important to transition your care from your pregnancy provider back to your PCP after your delivery.<br><br>Often, this "annual exam" is offered at no cost to you, depending on your insurer. For more information on costs, please visit: https://www.massgeneral.org/notices/billing/preventive-health-exams<br><br>If you are unable to make this appointment or wish to reschedule, please call your PCP's office at XXX or contact XXX with the Bridge Study Team. Please note that some clinics may have penalties for not showing to a scheduled visit.<br><br>--<br>*This message has been sent from The Bridge Study team. If you wish to no longer receive any messages from this team, please call the study coordinator XX or email the study team at XX. Do not reply directly to this message with questions about your health or to arrange follow-up.*	Dear XXX,<br><br>We do not see that you have a PCP appointment scheduled after your pregnancy in the upcoming 1-2 months. Please call your PCP's office to schedule. We will also call you within the next week to see if we can help.<br><br>The PCP appointment is important to transition your care from your pregnancy provider back to your PCP after your delivery. Often, this "annual exam" is offered with little to no cost to you, depending on your insurer.<br><br>Specific issues or risks from your pregnancy that you may want to address with your PCP include: XXX<br><br>--<br>*This message has been sent from The Bridge Study team. If you wish to no longer receive any messages from this team, please call the study coordinator XX or email the study team at XX. Do not reply directly to this message with questions about your health or to arrange follow-up.*
Intervention	4 weeks before PCP appt<br>OR<br>8 weeks pp if no PCP appt	SMS	Your PCP appointment is looking forward to your upcoming appointment! Check Patient Gateway for details. -The Bridge Study Team<br>[do not reply to this message]<br>*character count: 158*	Oh no! You do not have a PCP care transition appointment scheduled. Check Patient Gateway for details. -The Bridge Study Team<br>[do not reply to this message]<br>*character count: 159*

Group	Timing	Type	Message content if PCP appt scheduled	Message content if PCP appt not scheduled
Intervention	1 week before PCP appt OR 12 weeks pp if no PCP appt	PG	Dear XXX,<br><br>Your reserved postpartum-to-primary care transition appointment with your PCP is coming up!<br>XXX APPT INFO ##<br><br>This appointment is important to keeping you healthy in the future. Specific issues or risks from your pregnancy that you may want to address with your provider include: XXX<br><br>Often, this “annual exam” is offered at no cost to you, depending on your insurer. For more information on costs, please visit: https://www.massgeneral.org/notices/billing/preventive-health-exams<br><br>If you are unable to make this appointment or wish to reschedule, please call your PCP’s office at XXX or contact XXX with the Bridge Study Team. Please note that some clinics may have penalties for not showing to a scheduled visit.<br><br>--<br>*This message has been sent from The Bridge Study team. If you wish to no longer receive any messages from this team, please call the study coordinator XX or email the study team at XX. Do not reply directly to this message with questions about your health or to arrange follow-up.*	Dear XXX,<br><br>We do not see that you have a PCP appointment scheduled to transition your care after your pregnancy in the upcoming 1-2 months. We will call you within the next week to see if we can help.<br><br>The PCP appointment is important to transition your care from your pregnancy provider back to your PCP after your delivery. Often, this “annual exam” is offered with little to no cost to you, depending on your insurer.<br><br>--<br>*This message has been sent from The Bridge Study team. If you wish to no longer receive any messages from this team, please call the study coordinator XX or email the study team at XX. Do not reply directly to this message with questions about your health or to arrange follow-up.*
		SMS	Reminder: Your PCP appointment is within the next few days! Check Patient Gateway for details. -The Bridge Study Team<br>[do not reply to this message]<br>*character count: 148*	Reminder! You do not have a PCP care transition appointment scheduled. Check Patient Gateway for details. -The Bridge Study Team<br>[do not reply to this message]<br>*character count: 158*

Mass General Brigham

Title: Bridging the Gap from Postpartum to Primary Care: A Behavioral Science Informed Intervention to Improve Chronic Disease Management among Postpartum Women (the Bridge Study)

Sponsor Name:

PI Name: Clapp, Mark A **Protocol #:** 2022P001723 **Type:** Amendment (AME2)

Date Received: September 13, 2022

Signatures

PI Name: Clapp, Mark A, MD, MPH

Authenticated: September 13, 2022

Amendment

COVID-19 Amendment

Is this amendment ONLY related to research impacted by COVID-19?

Refer to MGB Policy on Conduct of Human Research Activities during COVID-19 Operations for description of Amendments which do **require prior IRB review and approval.**

- ◯ Yes
- ◉ No

Central IRB Performance Sites

Is this a protocol where the Mass General Brigham IRB is serving as the single IRB (sIRB) for external sites/institutions?

- ◯ Yes
- ◉ No

Sponsor Amendment

Is there a sponsor amendment number?

- ◯ Yes
- ◉ No

Change in Protocol Status

Is this a cede protocol or project that was determined to be exempt, not human subjects research or not engaged in human subjects research?

- ◯ Yes
- ◉ No

Do you need to change the overall status of the protocol? For example, Re-Open to Enrollment or indicate that Research Interventions/Assessments Continue after telling the IRB these have ceased.

- ◯ Yes
- ◉ No

Briefly describe the proposed changes:

No patients have been enrolled or approached yet.

The following amendments are being made:

1) minor adjustments to the enrollment criteria

2) minor updates to the language in the patient messages

3) added additional component to intervention arm (study team messages PCP that their patient is recently postpartum and an appointment has been scheduled for them)

4) added explicit language on the possibility of PCP visit cost to study messages and study fact sheet at request of project funder

5) minor sample size adjustments after more accurate estimates on potential eligible patients

6) study schema changed to reflect accurate outcome assessment time point

---

Provide rationale for the proposed changes:

Rationale:

1) minor adjustments to the enrollment criteria

- requires patient to have a PCP listed in Epic as there is limited to no available of "New" primary care appointments at MGH

- avoids enrollment of a patient with a new diagnosis or being evaluated for a stillbirth given the extenuating circumstances associated with this encounter

2) minor updates to the language in the patient messages

- updated to be consistent with a 6th grade reading level

- added information on the possibility of PCP visit cost at the recommendation of the project funder

3) added additional component to intervention arm (study team messages PCP that their patient is recently postpartum and an appointment has been scheduled for them)

- after discussion with Dr. Ray (PCP on the study team), this element was felt to be important

4) added explicit language on the possibility of PCP visit cost to study messages and study fact sheet

- added at the recommendation of the project funder

5) minor sample size adjustments

- more accurate estimates on potential eligible patients became available

6) study schema changed to reflect accurate outcome assessment time point

- the components of the intervention arm will be timed around the estimated date of delivery for consistency (not the actual variable date)

Will the proposed change(s) significantly alter the risk to benefit assessment the IRB relied upon to approve the protocol?

Mass General Brigham

◯ Yes
◉ No

Will the proposed change(s) significantly affect the integrity of the protocol?

◯ Yes
◉ No

Informed Consent

Do the changes require a revision to the consent form?

◯ Yes
◉ No

Attachments

Name	Mode
Study Fact Sheet_090122 (Consent Fact/Information Sheet)	Electronic
Detailed Protocol_090122_v1-3_clean (Detailed Protocol)	Electronic
PCP Study Baseline Survey_9-13-22 (Instrument/Questionnaire)	Electronic
Study Schema (Schema)	Electronic

Mass General Brigham

Research Study Fact Sheet

Bridging the Gap from Postpartum to Primary Care (The Bridge Study)

Principal Investigators	Mark Clapp, MD MPH (Massachusetts General Hospital)<br>Jessica Cohen, PhD (Harvard T. H. Chan School of Public Health)
Overview	This is a research study being conducted among pregnant and recently postpartum individuals who receive care at MGH.
Purpose	Your primary care provider (PCP) is responsible for managing and overseeing your current and long-term health. The purpose of this project is to improve primary care in the postpartum period.
Sponsor	This research is being sponsored by the National Institute on Aging, MIT Roybal Center for Translational Research to Improve Health Care for the Aging and the NBER Roybal Center for Behavior Change in Health.
Contact	We sent you a Patient Gateway message about this study because you are a person who may particularly benefit from receiving postpartum primary care.
Study Details	If you agree to participate, we will ask you a few questions about your health and visit history at the time of enrollment, which are all optional.<br><br>You will then be randomly assigned (50/50 chance) to be in the "standard care" group or the "facilitated transition" group. Regardless of which group you are assigned, you will continue to receive standard prenatal and postpartum care from your obstetrical care provider.<br><br>In the "standard care" group, you will receive a limited number of messages (less than 5) from the study team.<br><br>In the "facilitated transition" group, you will receive a limited number of messages (less than 5) from the study team between your enrollment and up to four months after your delivery. The study team will also assist in connecting you with your primary care provider after your delivery. Specifically, we may call your primary care provider's office on your behalf to help schedule an appointment. You always have the option to change or cancel this appointment. We also will message your PCP that you have recently delivered and that a follow-up appointment is being arranged.<br><br>Messages that we send you via Patient Gateway will also appear in your medical record for other members of your care team to see.<br><br>For both groups, we will review your medical record after your delivery to see if/when you received any care within the Mass General Brigham health system and information on your health status for up to two years after your delivery.<br><br>We also may contact you once between four and twelve months after your delivery to ask you questions about your postpartum care experience. You can decline to participate or answer any or all questions.<br><br>You may opt out of receiving messages or decline to participate in any survey, and/or withdraw from the study at any point without penalty.<br><br>Approximately 350 people will participate in this study.
Privacy/Confidentiality	We are required by the Health Insurance Portability and Accountability Act (HIPAA) to protect the privacy of health information obtained for research. More details related to the privacy of your health information is included on Pages 3-4 of this fact sheet.

	All information collected as a part of this study will be kept confidential and secure. As with all studies, there is a very low risk that others may become aware of your participation or health information. The study procedures and its protections of your information have been approved by the Mass General Brigham Human Subjects Research committee. Once the study has completed, all information that could identify you, such as your name or medical record number, will be deleted.
Study Information	We will collect information on your health and pregnancy history, including about visits you have with providers within the Mass General Brigham health system. Data collected as a part of this study will not affect your clinical care directly. Any messages sent from the study team will be visible to you and your care team within the electronic medical record. Your de-identified information may be used or shared with other researchers without your additional informed consent.
Risks	There are minimal risks to participate.<br><br>Although we will make every effort to protect participant privacy and confidentiality, it is possible that your involvement in the study could become known to others. We will be sending messages via Patient Gateway, which is a HIPAA-compliant method for communicating with you. You have the option to also receive SMS text messages that will not have any identifiable or personal health information. There is a rare possibility that study participation or health information could become known to others despite the use of firewalls, password protection, and other security measures.
Costs	You do not have to pay to take part in this research study.<br><br>By attending a PCP visit, there is a possibility that you will incur a charge, such as a co-pay or deductible payment, depending on your individual insurance coverage. If you do not have insurance, you may have to pay for the entire cost of the visit. We will provide you information on how to check potential costs with your insurer prior to your visit.
Participation	Those who participate will receive a $20 gift card after enrollment.<br><br>Participation is voluntary, and you can withdraw or stop the study at any time. Deciding not to participate won't affect medical care you receive at Mass General Brigham now or in the future, or any benefits they receive now or have a right to receive.
Study Contact	Mark Clapp, MD MPH, is the person at MGH who in charge of this research study. You can call him at 617-724-4531 (M-F 9a-5p). You can also call the study coordinator (Fowsia Warsame) at 617-643-5483 (M-F 9a-5p) with questions about this research study. You can also email bridgestudy@mgh.harvard.edu.<br><br>If you'd like to speak to someone not involved in this research about your rights as a research subject, or any concerns or complaints you may have about the research, contact the Mass General Brigham IRB at (857) 282-1900.

Option to Receive Text (SMS) Messages

Text messages by mobile/cell phones are a common form of communication. Our study team would like to send you no more than 3 text (SMS) messages as a part of the study, though **this is optional and not required to participate in the Bridge Study**. Texting over mobile/cell phones carries security risks because text messages to mobile/cell phones are not encrypted. This means that information you send or receive by text message could be intercepted or viewed by an unintended recipient, or by your mobile/cell phone provider or carrier.

Below are some important points about texting as a part of a research study:

- Receiving text messages is optional in this study and not required to participate.
- Text messages are not encrypted, and therefore carry security risks. This research study and Mass General Brigham are not responsible for any interception of messages sent through unencrypted text message communications.
- You will be responsible for all fees charged by your carrier's service plan for text messaging. This research study and Mass General Brigham are not responsible for any increased charges, data usage against plan limits or changes to data fees from the research texts.
- Text messaging should not be used in case of an emergency. If you experience a medical emergency, call 911 or go to the nearest hospital emergency department.
- You may decide to not send or receive text messages with staff associated with this research study at any time. You can do this in person or by sending the research number a text message that says "Stop Research Text."
- Your agreement applies to this research study only. Agreeing to other texts from Mass General Brigham, for example appointment reminders, is a separate process. Opting out of other texts from Mass General Brigham is a separate process as well.
- It is your responsibility to update your mobile/cell phone number with this research study in the event of a change.

Information Regarding the Privacy of Your Health Information

Federal law requires Mass General Brigham and its affiliated hospitals to protect the privacy of health information and related information that identifies you. We refer to this information as "protected health information." Your protected health information will be used and shared with others as explained below. You are agreeing to the collection, use, and sharing of your protected health information as described in this information sheet. If you have questions, you may ask the researcher who is reviewing this information sheet with you or you can contact the researcher listed above.

In this study, we may collect protected health information about you from:

- Past, present, and future medical records
- Research procedures, including but not limited to research visits, tests, interviews, and questionnaires

<u>Why will protected health information about you be used or shared with others?</u>
The main reasons include:

- to conduct and oversee the research described in this information sheet for this study;
- to ensure the research meets legal, institutional, and accreditation requirements; and
- to conduct public health activities (including reporting of adverse events or situations where you or others may be at risk of harm).

<u>Who may see, use, and share your protected health information and why they may need to do so?</u>

- Mass General Brigham researchers and staff involved in this study
- The sponsor(s) of the study, and people or groups it hires to help perform this research or to audit the research

- Other researchers and medical centers that are part of this study
- The Mass General Brigham ethics board
- A group that oversees the data (study information) and safety of this study
- Non-research staff within Mass General Brigham who need identifiable information to do their jobs, such as for treatment, payment (billing), or hospital operations (such as assessing the quality of care or research)
- People or groups that we hire to do certain work for us, such as data storage companies, accreditors, insurers, and lawyers
- Federal agencies (such as the U.S. Department of Health and Human Services (DHHS) and agencies within DHHS like the Food and Drug Administration, the National Institutes of Health, and the Office for Human Research Protections), state agencies, and foreign government bodies that oversee, evaluate, and audit research, which may include inspection of your records
- Public health and safety authorities, if we learn information that could mean harm to you or others (such as to make required reports about communicable diseases or about child or elder abuse)

Some people or groups who get your protected health information might not have to follow the same privacy rules that we follow and might use or share your protected health information without your permission in ways that are not described in this form. We share your protected health information only when we must, and we ask anyone who receives it from us to take measures to protect your privacy. However, once your protected health information is shared outside Mass General Brigham, we cannot control all the ways that others use or share it and cannot promise that it will remain private.

The results of this research study may be published in a medical book or journal or used to teach others. However, your name or other identifiable information **will not** be used for these purposes without your specific permission.

<u>For how long will protected health information about you be used or shared with others?</u>
Because research is an ongoing process, we cannot give you an exact date when we will either destroy or stop using or sharing your protected health information. Your permission to use and share your protected health information does not expire.

<u>Your Privacy Rights</u>
You have the right not to agree to our use and sharing of your protected health information for research; however, if you don't agree, you can't take part in this research study. However, refusing to agree will not affect your present or future care and will not cause any penalty or loss of benefits to which you are otherwise entitled.

You have the right to withdraw your permission for the further use or sharing of your protected health information for this research study. If you want to withdraw your permission, you must notify the person in charge of this research study in writing at the name and address listed above. Once permission is withdrawn, you cannot continue to take part in the study.

If you withdraw your permission, then to the extent that we have retained any protected health information that can be linked to you, we will stop using and sharing the protected health information further for the research. However, we will not be able to take back information that has already been used or shared with others, and such information may continue to be used for certain purposes, such as to comply with the law or maintain the reliability of the study.

You have the right to see and get a copy of your protected health information that is used or shared for treatment or for payment. To ask for this information, please contact the person in charge of this research study identified above. You may only get such information after the research is finished.

Mass General Brigham

Institutional Review Board
Intervention/Interaction Detailed Protocol

Principal Investigator:	Mark Clapp, MD MPH (MGH), Jessica Cohen, PhD (HSPH)
Project Title:	Bridging the Gap from Postpartum to Primary Care: A Behavioral Science Informed Intervention to Improve Chronic Disease Management among Postpartum Women
Version Date:	09/01/2022
Version Name/Number:	v1.3

1. Background and Significance

Burden of Chronic Disease and the Role of Primary Care
Chronic health conditions affect millions of people in the US each year. In 2018, 51.8% of adults had at least 1 chronic condition, and 27.2% had multiple conditions.[1] The prevalence of chronic disease was higher in women compared to men, older adults (87.6% in adults ≥65 years old), and people with public insurance.[1] Many chronic conditions, by their nature, develop over time and have risk factors that can be identified prior to the onset of disease. Strong evidence underpins prevention strategies for many conditions, which are advanced by the US Preventative Services Taskforce.[2] While the long-term health of a patient is the responsibility of an entire health system, primary care providers (PCPs) provide an integral role in preventing, screening for, and managing disease across the lifespan. Studies have shown the health benefits of receiving regular care under a PCP.[3–6]

Despite the known benefits of having an identifiable usual source of care and the value of health care maintenance, the percent of the population with a PCP has been decreasing over time.[7] Consistently, adults who are younger (age 20-40 years) have the lowest rates of primary care use. In 2015, 44% and 36% of 20- and 30-year-olds had no identifiable source of primary care.[7] The proportion without primary care were also higher among racial/ethnic minority populations and among those who had less education, lower incomes, and no known comorbidities.[7] The number of adults and the time elapsed without regular primary care follow-up can be considered missed opportunities to improve a patient's current and long-term health. The disproportionate lack of primary care among certain subgroups of the population, often groups who already have worse health outcomes, only serves to widen the pre-existing disparities.

Pregnancy as a Window to Future Health
In the US, 98.2% of pregnant women receive some form of prenatal care, with the average patient having >10 visits during their pregnancy.[8] During a pregnancy, women are screened for pre-existing and pregnancy-related conditions.[9] In adults ages 18-39, the prevalence of obesity, hypertension, prediabetes/diabetes, and mental illness are estimated at 39%, 7.5%, 28%, 25%, respectively.[10–13] Even for those who have no prior identified comorbidities, the most common

pregnancy-related conditions—pregnancy-related hypertension and gestational diabetes (8% and 10% of pregnancies, respectively)—indicate a predisposition to or confer health risks that persist as women age. For example, over 25% of women with gestational diabetes will develop Type 2 diabetes mellitus, and women with pre-eclampsia have more than a two-fold risk of significant cardiovascular disease later in life.[14,15] For these reasons, pregnancy is often considered a "window" into a woman's future health and presents a unique opportunity to optimize a woman's health status early in her life when she otherwise may not have been engaged in care.[16]

Pregnancy as on Opportunity for Engagement with a Long-term Care Provider
Pregnancy is a period when women are highly engaged and active participants in their health care.[17] It has been described as a "golden opportunity" to motivate women towards positive health behaviors, including prevention and management of chronic disease. However, women often fall off a "postpartum cliff" of health system engagement after the early postpartum period.[18] A range of systemic, financial, and behavioral barriers often prevents patients from effectively transitioning to primary care. Postpartum women are often simply told to follow-up with their PCP without much information regarding the importance of this follow up care, without assistance in scheduling an appointment (or identifying a PCP if they don't have one), and often without a direct transfer of relevant health information or accountability across providers.

Postpartum women are left largely on their own to navigate this transition to primary care and, in particular, to navigate it at a time when they face the high cognitive and physical demands of caring for an infant. At this time of limited cognitive bandwidth, the importance of continuity of care for chronic conditions and active engagement in one's longer-term health and wellbeing is unlikely to be salient and top-of-mind. These critical moments of unsupported health care transition can exacerbate pre-existing disparities in health and health care, with patients who are the least able to navigate the US health care system most likely to fall through the cracks. Momentum is building in US health and social safety net policy to facilitate healthy transitions from pregnancy to parenthood. For example, federal and state initiatives to expand pregnancy-related Medicaid coverage from 60 days to one year postpartum have been proposed, and access to paid family leave is increasing. However, very little evidence exists on effective and cost-effective approaches to facilitating transitions to primary care and management of chronic diseases in the postpartum year.

2. Specific Aims and Objectives

The objective of the proposed study is to increase patient engagement in primary care after the immediate postpartum period for women with pregnancy-associated conditions that convey a long-term health risk. Specifically, we aim to evaluate the efficacy of an intervention bundle (automatic scheduling of PCP appointment after delivery, salient labeling, and appointment reminder nudges) to increase patient attendance at a primary care provider appointment (within 4 months of delivery for women with or at risk for obesity, diabetes, hypertension, and/or a mental health condition.

Specific Aims:

1) Test the efficacy of an intervention bundle (patient-tailored health information, automatic scheduling of PCP appointment after delivery, and appointment reminder nudges) to increase patient attendance at a primary care provider appointment within 4 months of

delivery for women with obesity, diabetes, hypertension, and/or a mental health condition
2) Test the efficacy of the intervention bundle to improve compliance with the condition-specific, guideline-based health screenings
3) Test the efficacy of the intervention bundle to reduce unscheduled or urgent encounters (e.g., emergency department visits) within the health system

3. General Description of Study Design

We will conduct a randomized controlled trial comparing this intervention bundle to the receipt of generic information on the importance of primary care follow-up after delivery.

Women will be randomized with equal probability into either a treatment or control arm. The intervention combines several features designed to target reasons for low take-up of primary care among postpartum women (see Logic Model). We leverage the potential value of defaults/opt-out, salient labels, and reminders to encourage use of primary care within 4 months of delivery. Women in both the intervention and control arms will receive information via MGH's patient portal toward the end of the pregnancy regarding the importance of transitions to primary care in the postpartum year. This information will be similar to, but reinforcing, the information they would receive from their obstetrician about following up with their primary care physician. In addition to this initial message, women in the treatment arm will receive the following intervention components, developed based on recent evidence regarding behavioral science approaches to activating health behaviors:[19–21]

- Targeted messages about the importance and benefits of primary care.
- Default scheduling into a primary care appointment at approximately 3-4 months after delivery. The patient will be scheduled for a primary care visit with their assigned primary care provider in the Mass General Brigham system. They will be informed of the option to cancel the appointment, change the appointment day/time, or change the care provider either through the patient portal).
- Reminders about the appointment and importance of follow up primary care at 2-4 points during the postpartum period via the patient portal.
- Tailored language in the reminders based on recent evidence from behavioral science about the most effective approaches to increasing take-up. For example, messages will inform the patient that an appointment has been reserved for them at their doctor.

The Logic Framework that underpins the basis for this study is shown below:

Needs/ Problems	Barriers to Primary Care Use	Input/Intervention	Outcomes	Long-term Goals
• Low PCP use among people who have/are at risk of chronic conditions (diabetes, hypertension, obesity, mental illness)<br><br>• Pregnancy is ideal opportunity to interrupt progression of chronic disease, but this window into long-term health is often missed	• Insufficient patient information<br>• Underestimate risks<br>• Low salience<br>• Cognitive demands in postpartum period<br>• Time constraints and competing priorities<br>• Health system/structural barriers to postpartum care continuity<br>• Poor transitions between obstetricians and PCPs	• Targeted information about importance of primary care<br>• Default scheduling of postpartum primary care appointment<br>• Reminder messages about primary care appointment leveraging salience and pre-commitment<br>• Salient labeling of the transition appointment<br>• PCP messaging about a patient's recent pregnancy and upcoming follow-up	**PRIMARY**<br>• Visit with primary care provider within 4 months of childbirth<br><br>**SECONDARY**<br>• Condition-specific screening:<br>-documentation of blood pressure<br>-diabetes screening test<br>-measurement of weight<br><br>• Counseling on risk prevention or reduction strategies at visit<br><br>• Use of emergency room or urgent care for any reason	• Engagement with PCP<br>• Earlier, more effective chronic disease prevention and management<br>• Interruption of disease progression over life course<br>• Improved lifelong health status

The flow of study activities is shown below:

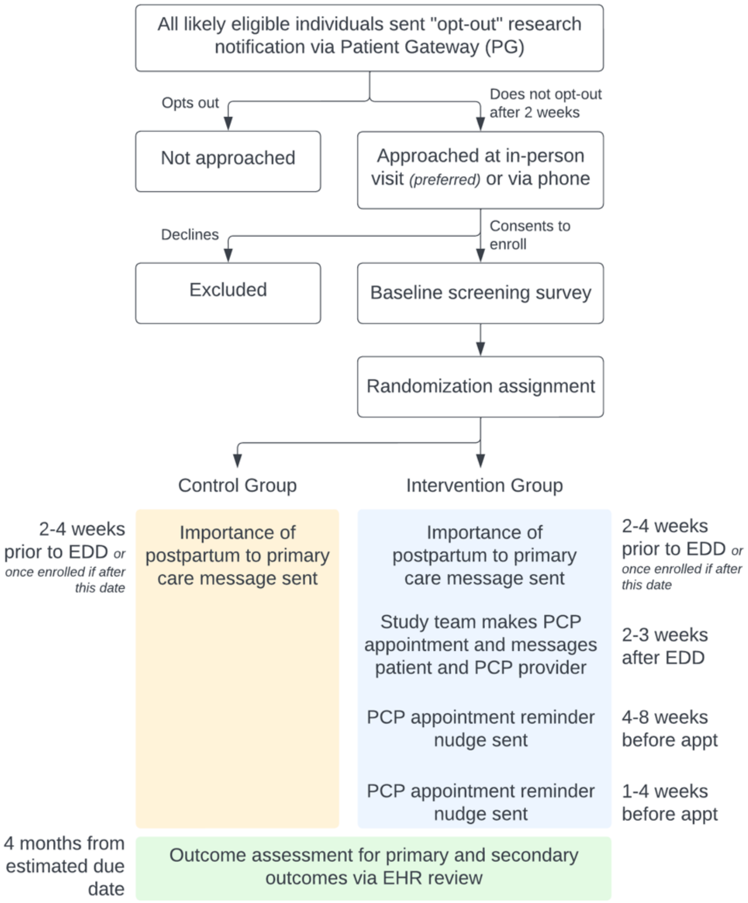

4. Subject Selection

Pregnant patients receiving prenatal care at Massachusetts General Hospital (MGH) will be targeted for recruitment. MGH conducts approximately 3500 deliveries per year, with roughly one-third of patients identifying as non-white and 35% of pregnancies covered by Medicaid.

Approximately 49% of the clinic population may ultimately be eligible to be approached. The eligibility criteria include:

- Estimated date of delivery and the following 4-month postpartum outcome assessment window completed prior to study end date
- Currently pregnant or within 2 weeks of delivery
- Have one or more of the following conditions:
  - Chronic hypertension
  - Hypertensive disorders of pregnancy or risk factors for hypertensive disorders of pregnancy per the USPTF aspirin prescribing guidelines (e.g., history of pre-eclampsia, kidney disease, multiple gestation, autoimmune disease)[22]
  - Type 1 or 2 diabetes

  - Gestational diabetes
  - Obesity (body mass index ≥30 kg/m2)
  - Depression or anxiety disorder
- Have a primary care provider listed in the patient's medical record
- Receive obstetric care at an MGH-affiliated outpatient prenatal clinic
- Has access to and be enrolled in the electronic health record patient portal and consents to be contacted via these modalities
- Able to read/speak English or Spanish language
- Age ≥18 years old
- Not diagnosed with or undergoing evaluation for stillbirth/fetal demise

All women, regardless of race/ethnicity, who meet the eligibility criteria will be included. In 2019, 3,789 women gave birth at the study institution, of whom 57% were white, 7% were black, 12% were Asian, 18% were Hispanic, and 6% declined to report their race/ethnicity. A similar distribution is expected for this study.

The study will distribute materials in English and Spanish languages. This encompasses >95% of patients who deliver at the study institution.

5. Subject Enrollment

This study will rely on recruiting for research through Patient Gateway and follow the IRB guidance and DHeC Research Checklist and training for this process.

In the month prior to the start of enrollment, all potentially eligible individuals (based on the criteria above) will be identified using RPDR and Epic Reporting search queries. This list (the "potentially eligible" list) will be provided to the DHeCare Research Team to build an RSH Record in Epic. This list will be updated monthly to identify newly eligible individuals (e.g., new diagnosis of gestational diabetes or new patient transferring into the practice) during the recruitment months and fed back to the DHeCare team to update "potentially eligible" list.

Once built and each month during recruitment phase, the study's research coordinator (not study investigators) will send the IRB-approved Research Invitation Letter to patients who are eligible, not already enrolled, and have not declined to be sent research notifications through the portal. The Research Letter will employ an opt-out approach, asking individuals who do not wish to be approached in clinic or remotely to respond via PG messaging, email, or phone within 2 weeks of receipt of the letter. Those who have "read" the letter and not opted out after 2 weeks will be moved from the "potentially eligible" list to the "waiting to be approached" list in the study workflow.

During recruitment months, the study coordinator will keep a log of patient's upcoming appointments for those on the "waiting to be approached" list. They will then attempt to approach individuals for enrollment when they present for an in-person encounters (preferred). Patients will ideally be approached between 32-36 weeks of gestation, when feasible; however, priority will be given to patients at the latest gestation. For those Spanish-speaking patients, a hospital-based interpreter will be used when approaching/consenting patients.

The study staff will introduce the study and review the purpose of the study, the nature of the subject's participation, the possible risks and discomforts associated with participation, the potential benefits of participation, a statement of the voluntary nature of participation, and a description of the mechanisms used to ensure confidentiality.

All patients will receive study-related messages through Patient Gateway. In addition, patients will be asked if they would be willing to receive no more than 5 SMS messages to their personal cell phone during the first 4 months of the postpartum period. The study staff will review the specific concerns and risks about receiving unencrypted text messaging communications, as outlined by the MGB IRB.

A waiver of documentation of informed consent is requested, as the study presents no more than minimal risk of harm to subjects and involves no procedures for which written consent is normally required outside of the research context. Verbal consent will be obtained for 1) overall study participation and 2) optional SMS message participation, separately. Verbal consent will be obtained from both in-person and phone recruitment efforts.

A Study Fact Sheet, which summarizes the study details, risks, and benefits, will be provided to all subjects who are approached, either in-person, by mail, or electronically. This Study Fact Sheet also includes information supplied by the MGB IRB specifically related to the concerns and risks of receiving unencrypted SMS/text messages.

For tracking verbal consent, the study staff will keep a detailed log in REDCap documenting:

1) name of study staff performing consent,
2) date of attempted approach,
3) the method of attempted approach (in-person or via phone),
4) use of Spanish interpreter (yes/no),
5) subject agreement to be approached (agree/disagree),
6) attestation to full review of the study procedures/risks/benefits with the subject, as would be done during the process of reviewing a written consent form,
7) attestation to review of supplemental consent to receive unencrypted SMS messages with the subject,
8) subject overall study participation status (enrolled, declined, deferred – agrees to be recontacted, deferred – wishes not to be recontacted),
9) if enrolled, unencrypted SMS text messaging participation (consents, declines),
10) attestation to Study Fact Sheet provided,
11) method by which Study Fact Sheet was provided (in-person, mail, electronic),
12) date in which Study Fact Sheet was provided (in-person, mail, electronic).

6. STUDY PROCEDURES

The RA will keep a detailed log of all patients in the practice, if they are eligible, if they have been approached, and if they consented.

For those that agree to be enrolled, patients will be asked to complete a baseline survey to obtain voluntarily reported information on their demographics, socioeconomic status, health care visit history, and primary care provider. Patients will also be asked to consent to being contacted

by the research team via Patient Gateway messaging. A $20 gift card will be given at the time of enrollment for those that complete the questionnaire and receive the information sheet.

Randomization will occur via a prespecified random allocation sequence within strata. Within each stratum, the PIs will generate a random sequence of treatment-control allocation prior the enrollment of subjects. Then, as patients within strata are enrolled, they will be assigned to the treatment arm associated with that enrollment number.

<u>Control Group</u>
Approximately 2 weeks before a patient's estimated due date (EDD) or as soon as enrolled if this date has passed, the patient will be sent information via the Patient Gateway on the importance of postpartum care and follow-up with their PCP.

<u>Intervention Group</u>

1. Tailored Information:
   Approximately 2-4 weeks prior to the EDD (or later for those who are enrolled beyond 38 weeks gestation or postnatally), the patient will be sent an information via the Patient Gateway on the importance of postpartum care and follow-up with their PCP, which also includes the name and phone number of their primary care provider.
2. Scheduled PCP Appointment:
   Between 2-3 weeks after their delivery, the RA will call the patient's PCP office and make an appointment for them between 3-4 months after delivery based on the scheduling preferences obtained in the initial survey.
3. Targeted Appointment Message with Salient Labeling:
   After the PCP appointment has been made, the patient will be sent a Patient Gateway message saying that a PCP appointment has been reserved for them with the date/time/location information.
   For those consenting to receive SMS messages, an unencrypted text message will also be sent simultaneously.
4. Nudge Reminders:
   Patient Gateway messages will be sent at approximately 4-8 weeks (goal: 4 weeks) and 1-4 week (goal: 1 week) prior to their PCP appointment, reminding a patient of their upcoming appointment.
   For those consenting to receive SMS messages, an unencrypted text message will also be sent simultaneously.
5. Facilitated PCP Communication:
   A study staff member will send the patient's PCP an Epic Inbasket message that the patient 1) is recently postpartum, 2) has or developed health conditions that need long-term management, and 3) has been scheduled (or attempted to be scheduled) for a follow-up visit.

All messages will be made available in English and Spanish.

The text for these Patient Gateway messages is included in the submission. At the end of each patient message, patients will be given the opportunity to stop receiving study-related messages by emailing or calling the study staff. "Opt out" requests will be logged and patients removed from future planned study-related contact.

The text for 3 SMS messages is included in the submission. SMS messages will be sent via Google Voice. We will follow standard recommendations from the MGB Research Information Security for using this system (included under Privacy and Confidentiality section).

The study team will review the patient's EHR record for the primary and secondary outcome assessments. Patients will be asked to consent to have records reviewed up to 2 years after the date of their delivery to allow for long-term effects of the intervention on primary care use and health status.

During the initial consent process, patients will be asked to agree to be potentially contacted at the end of the first 4-month follow-up period for a survey. Currently, this endline survey is not planned due to funding limitations; however, if funding becomes available, an IRB amendment will be submitted for review of the endline survey prior to being administered to any study subjects.

Deidentified data from this project may be shared outside of MGB with the study funders (J-PAL/NBER/NIA) for data sharing and reproducibility requirements and secondary statistical analysis. All data will be stripped of patient identifiers, per IRB guidance. No data will be shared without a formal Data Use Agreement with MGB.

6. Risks and Discomforts

There are minimal risks to participants.

Patients will be reassured that nonparticipation will not affect clinical care. Patients will also be informed that the researchers with whom they will interact (e.g., during consent, face-to-face, during telephone interviews) are not health care providers. Participants will be consistently reminded that responses to any queries deemed sensitive or uncomfortable (e.g., country of origin/immigration status, income, previous history of abortion) should be considered optional, and they may decline to answer any question(s) and can refuse to continue the study at any point. Participants will be reassured that neither their opportunities for continued health care nor their relationships with health care providers will be jeopardized by study participation.

Health information collected as part of this study will be stored in REDCap. No identifiable data will be stored or downloaded on any personal or unauthorized computers. Study staff will access the data on institutionally purchased and managed computers that operate behind the health system's security and firewall protections. Only the MGH study staff will have access to the identifiable data set. Once the study is completed, data will be deidentified such that it can be analyzed without risk of a breach of privacy or confidentiality. Any data that is shared outside of MGH will require a data use authorization.

Although we will make every effort to protect participant privacy and confidentiality, it is possible that participants' involvement in the study could become known to others. For those in the intervention group, we will be sending personalized messages via the patient portal; for those providing additional consent, we will be sending unencrypted SMS messages that will not contain any personal protected health information. There is the rare possibility that study participation or health information could become known to others despite the use of firewalls,

password protection, and other security measures. Standard procedures, as outlined by the MGB Research Information Security Office, will be followed to reduce this risk.

Individuals in the intervention group will be scheduled for an appointment with a primary care doctor. There is the possibility that this visit may result in a charge/cost for the patient. Under the Affordable Care Act, commercial health plans are required to cover an annual Preventive Health Exam at no cost to the patient (no co-payment, co-insurance or deductible). MassHealth also covers these visits without cost-sharing. However, if this visit turns or scheduled as into a “sick” or “disease management” visit, the patient may be billed for some or all aspects of the services provided, depending on their insurer. We will provide directions for patients on how to to contact their insurers or PCP’s office prior to the visit to inquire about potential cost-sharing and/or deductibles. Patients will also be advised that some clinics may penalize individuals for not showing to a scheduled appointment (“no show” fee) and be given opportunities to request the appointment be canceled or rescheduled with each appointment reminder.

7. Benefits

Participants in the control group will receive information on the importance of postpartum care and transitioning to primary care after their delivery.

Participants in the intervention group will receive a bundle of interventions designed to increase attendance at primary care visits and facilitate the transition of care after their delivery.

The goal and potential benefits to the subjects in this trial is to increase patient engagement and connection with their primary care provider, receive recommended health screenings and directed counseling, and reduce unscheduled or urgent visits in the postpartum period.

8. Statistical Analysis

Statistical Methods
Analyses will be performed according to the intention-to-treat principle.

Standard independent, two-sided, two-group comparison testing will be used to compare baseline characteristics between the two groups (chi squared tests, t tests, Wilcoxon rank sum tests, when appropriate).

The primary outcome will be attendance rates at PCP visit within 4 months after estimated date of delivery (captured at time of enrollment), which will be compared between the groups using chi squared tests. Relative risks and 95% confidence intervals will be reported.

Secondary outcomes will include measures of long-term health and health care use after the postpartum period.

Subgroup analyses will be performed by a variety of patient characteristics including gestational age at enrollment, prenatal risk factors, morbidity types, patient race-ethnicity, payer, and enrollment location.

P-value of less than 0.05 will be considered to indicate statistical significance.

Power Calculation
Most study calculations were estimated from the MGH 2020 delivery population. The rates of PCP follow-up were estimated from a randomly selected cohort of 50 patients who met the inclusion criteria. We plan to recruit patients into the study for a period of 4 months and expect 1200 unique patients to be at the targeted gestational age during this period. Among these, we expect 86% to already have an assigned PCP in the network, leaving roughly 1,032 patients. Among these, we estimate that 49% have at least one of the targeted health conditions, leaving a target study population of 506 over the 4-month period. Based on previous studies conducted at MGH, we expect an 70% willingness to participate in the research, leaving an expected recruited population of 354 individuals. Based on our record extraction, we estimate that 15% of the targeted study population has a primary care visit within 4 months of delivery. Assuming an alpha of 0.05 and a baseline mean of 15%, with this expected sample size and power of 80%, our study has a minimum detectable effect size of roughly 13 percentage points (from 15% to 28%). A previous study found that default scheduling into postpartum care appointments (with an OBGYN, not a PCP) increased postpartum care take-up by 24 percentage points; since our intervention incorporates defaults and other activating interventions, an MDE of 13 percentage points is reasonable.

9. Monitoring and Quality Assurance

This is a minimal risk study in which the intervention involves default scheduling of appointments and patient messaging. Adverse events are not expected, and there is no physiologic plausibility for this intervention to cause any NIH-defined serious adverse events (e.g., death, prolonged hospitalization, significant disability).

No interim analyses are planned.

Adverse events will be defined and classified in accordance with NIH guidelines:

> Definition of Adverse Events (AE): Any untoward or unfavorable medical occurrence in a human subject, including any abnormal sign, symptom, or disease, temporally associated with the subject’s participation in the research, whether considered related to the subject’s participation in the research or not.
>
> Definition of Serious Adverse Events (SAE): Any AE that (1) results in death, (2) is life-threatening, (3) results in inpatient hospitalization or prolongation of existing hospitalization, (4) results in persistent or significant disability/incapacity, (5) results in a congenital anomaly/birth defect, and/or (6) may jeopardize the subject’s health and may require medical or surgical intervention to prevent one of the other five outcomes listed here.

In the unlikely event an AE or SAE occurs, it will be brought to the PI's attention, and the PI will classify the AE/SAE by severity, expectedness, and relatedness,as listed above. All events that are both serious and unexpected will be reported to Mass General Brigham's IRB, the NIA

PO, and to the NIA Roybal DSMB within 48 hours of the research team's knowledge of the SAE. The summary of all other SAEs will be reported to the NIA and to the DSMB quarterly unless otherwise specified by the DSMB. Any unanticipated problem, defined as an issue related to the research suggesting the research places participants or others at greater risk than expected, will be reported to the IRB, the NIA PO, and to the Roybal DSMB within 48 hours of discovery. If the problem involves death then reporting will occur within 24 hours, and this report will include a plan to correct the problem and prevent its occurrence. Any breach of PHI will be reported to the PI, who will report to the IRB and NIA PO within 24 hours of discovery.

The Roybal DSMB oversight is provided by the Standing Roybal DSMB, which includes the members listed: Andrea B. Troxel, ScD (chair); Abby King, PhD; Jerry Gurwitz, MD;Hae-Ra Han, PhD, RN, FAAN; Hang Lee, PhD; Ezra Golberstein, PhD; David Kim, MD PhD; Christopher Celano, MD.

DSMB members will have no direct involvement with the study or conflict of interest with the investigators or institutions conducting the study. Each member has signed a COI statement which includes current affiliations, if any, with pharmaceutical or biotechnology companies (e.g., stockholder, consultant), and any other relationship that could be perceived as a conflict of interest related to the study and/or associatedwith commercial interests pertinent to study objectives.

Data presented to the DSMB will be deidentified as to protect individual participants' privacy and health information. Should the identity of a deidentified subject need to be revealed, the DSMB request will be reviewed and ultimately at the discretion of the Mass General Brigham IRB.

10. Privacy and Confidentiality

- ☒ Study procedures will be conducted in a private setting
- ☒ Only data and/or specimens necessary for the conduct of the study will be collected
- ☒ Data collected (paper and/or electronic) will be maintained in a secure location with appropriate protections such as password protection, encryption, physical security measures (locked files/areas)
- ☐ Specimens collected will be maintained in a secure location with appropriate protections (e.g. locked storage spaces, laboratory areas)
- ☒ Data and specimens will only be shared with individuals who are members of the IRB-approved research team or approved for sharing as described in this IRB protocol
- ☒ Data and/or specimens requiring transportation from one location or electronic space to another will be transported only in a secure manner (e.g. encrypted files, password protection, using chain-of-custody procedures, etc.)
- ☒ All electronic communication with participants will comply with Mass General Brigham secure communication policies
- ☒ Identifiers will be coded or removed as soon as feasible and access to files linking identifiers with coded data or specimens will be limited to the minimal necessary members of the research team required to conduct the research
- ☒ All staff are trained on and will follow the Mass General Brigham policies and procedures for maintaining appropriate confidentiality of research data and specimens

☒ The PI will ensure that all staff implement and follow any Research Information Service Office (RISO) requirements for this research
☒ Additional privacy and/or confidentiality protections

The following procedures (as provided by MGB RISO) will be used for sending SMS messages via Google Voice:

- Google Account:
    - A separate account should be created just for the purpose of the effort/study (in other words, personal Gmail accounts should not be used)
    - For the Google Voice portal, make sure you are not using the same password as your MGB account and ensure the password is strong. (Minimum of 8 characters, alphanumeric, uppercase, lowercase, special character).
        - Two-factor authentication must be enabled
    - Google account will not be shared
    - The Google account used for Google Voice should not be used for emailing or using any other Google Service (i.e., YouTube, Calendar, Contacts, etc.)
    - No credit cards should be added to the Google Account
    - The Google account must be deleted at the end of the study / project
- Participants will be informed not to send personal or health related information via text
- Siri will not be integrated with Google Voice
- Text messages will not address participants by their first name
- Text messages will be sent through the McLean email within the Google account that is created
- Only phone numbers and a unique subject ID will be stored in Google Voice
    - Log records should be deleted manually after 30 days
- No PHI or sensitive information will be communicated via text message
    - Content will not include anything where a healthcare condition or diagnosis can be inferred
- Text message history should be deleted from Google Voice account when no longer needed (within 30 days)
- Study staff will track opt-out requests and delete phone numbers from Google Voice as necessary
- Study staff must not communicate with participants via a group text message
- Access to the portal and overall research must be done from systems that meet MRB RISO compliance requirements; encryption, MobileIron (if Smartphone/Tablet), up to date malware protection, Crowdstrike.
    - https://rc.partners.org/security/secure-your-computer
- Participants:
    - Participants will be texted only if they consent
    - Participants should be informed to delete text messages when no longer needed and hide text push notifications

12. References

1. Boersma P. Prevalence of Multiple Chronic Conditions Among US Adults, 2018. *Prev Chronic Dis*. 2020;17. doi:10.5888/pcd17.200130
2. Home page | United States Preventive Services Taskforce. Accessed December 15, 2021. https://www.uspreventiveservicestaskforce.org/uspstf/
3. Blewett LA, Johnson PJ, Lee B, Scal PB. When a usual source of care and usual provider matter: adult prevention and screening services. *J Gen Intern Med*. 2008;23(9):1354-1360. doi:10.1007/s11606-008-0659-0
4. O'Malley AS, Mandelblatt J, Gold K, Cagney KA, Kerner J. Continuity of care and the use of breast and cervical cancer screening services in a multiethnic community. *Arch Intern Med*. 1997;157(13):1462-1470.
5. DeVoe JE, Saultz JW, Krois L, Tillotson CJ. A medical home versus temporary housing: the importance of a stable usual source of care. *Pediatrics*. 2009;124(5):1363-1371. doi:10.1542/peds.2008-3141
6. Atlas SJ, Grant RW, Ferris TG, Chang Y, Barry MJ. Patient-physician connectedness and quality of primary care. *Ann Intern Med*. 2009;150(5):325-335. doi:10.7326/0003-4819-150-5-200903030-00008
7. Levine DM, Linder JA, Landon BE. Characteristics of Americans With Primary Care and Changes Over Time, 2002-2015. *JAMA Internal Medicine*. 2020;180(3):463-466. doi:10.1001/jamainternmed.2019.6282
8. NVSS - Birth Data. Published June 14, 2021. Accessed September 1, 2021. https://www.cdc.gov/nchs/nvss/births.htm
9. Prenatal care and tests | Office on Women's Health. Accessed December 15, 2021. https://www.womenshealth.gov/pregnancy/youre-pregnant-now-what/prenatal-care-and-tests
10. Centers for Disease Control and Prevention (CDC). Adult Obesity Facts. Published August 29, 2017. Accessed February 14, 2018. https://www.cdc.gov/obesity/data/adult.html
11. Mental Illness. National Institute of Mental Health (NIMH). Accessed December 17, 2021. https://www.nimh.nih.gov/health/statistics/mental-illness
12. Products - Data Briefs - Number 289 - October 2017. Published June 6, 2019. Accessed December 17, 2021. https://www.cdc.gov/nchs/products/databriefs/db289.htm
13. National Diabetes Statistics Report 2020. Estimates of diabetes and its burden in the United States. Published online 2020:32.
14. Sattar N, Greer IA. Pregnancy complications and maternal cardiovascular risk: opportunities for intervention and screening? *BMJ*. 2002;325(7356):157-160. doi:10.1136/bmj.325.7356.157
15. Kim C, Tabaei BP, Burke R, et al. Missed Opportunities for Type 2 Diabetes Mellitus Screening Among Women With a History of Gestational Diabetes Mellitus. *Am J Public Health*. 2006;96(9):1643-1648. doi:10.2105/AJPH.2005.065722
16. Media BKK. Publications & Guidelines | SMFM.org - The Society for Maternal-Fetal Medicine. Accessed October 18, 2021. https://www.smfm.org/publications/225-smfm-statement-implementation-of-the-use-of-antenatal-corticosteroids-in-the-late-preterm-birth-period-in-women-at-risk-for-preterm-delivery
17. Yee LM, Simon MA, Grobman WA, Rajan PV. Pregnancy as a "golden opportunity" for patient activation and engagement. *American Journal of Obstetrics & Gynecology*. 2021;224(1):116-118. doi:10.1016/j.ajog.2020.09.024

18. Cohen JL, Daw JR. Postpartum Cliffs—Missed Opportunities to Promote Maternal Health in the United States. *JAMA Health Forum*. 2021;2(12):e214164. doi:10.1001/jamahealthforum.2021.4164
19. Milkman KL, Patel MS, Gandhi L, et al. A megastudy of text-based nudges encouraging patients to get vaccinated at an upcoming doctor's appointment. *PNAS*. 2021;118(20). doi:10.1073/pnas.2101165118
20. Patel MS, Volpp KG, Asch DA. Nudge Units to Improve the Delivery of Health Care. *N Engl J Med*. 2018;378(3):214-216. doi:10.1056/NEJMp1712984
21. Thaler RH, Sunstein CR. *Nudge: The Final Edition*. Revised edition. Penguin Books; 2021.
22. Henderson JT, Vesco KK, Senger CA, Thomas RG, Redmond N. Aspirin Use to Prevent Preeclampsia and Related Morbidity and Mortality: Updated Evidence Report and Systematic Review for the US Preventive Services Task Force. *JAMA*. 2021;326(12):1192. doi:10.1001/jama.2021.8551

Bridging the Gap from Postpartum to Primary Care: A Behavioral Science Informed Intervention to Improve Chronic Disease Management among Postpartum Women

Study PIs: Mark Clapp, MD MPH (MGH); Jessica Cohen, PhD (Harvard)
Site PI: Mark Clapp, MD MPH (MGH)

Baseline Survey

SECTION 1. Interview Information

101. RA ID number
102: Enrollment Facility ID number
103: Enrollment Facility name
104: Date of interview (MM/DD/YY)
105: Start time of interview (HH:MM)<br>AM_____ PM_______ (Tick one)
106: End time of interview (HH:MM)<br>AM_____ PM_______ (Tick one)
107: Time of interview in relation to patient's prenatal/postnatal visit:<br>1. In-person, during prenatal visit<br>2. In-person, during postpartum visit<br>3. Phone, before delivery<br>4. Phone, after delivery

SECTION 2. Eligibility Criteria

- ≥18 years old
- Able to read/speak English or Spanish language
- Received research letter > 2 weeks ago and
- Did not opt out of study
- Has PCP listed in Epic
- Not diagnosed with or being evaluated for fetal demise at time of enrollment
- Have one or more of the following conditions listed in Epic Problem List:
  - □ Chronic hypertension
  - □ Hypertensive disorders of pregnancy or risk factors for hypertensive disorders of pregnancy (e.g., history of preeclampsia, kidney disease, multiple gestation, autoimmune disease)
  - □ Type 1 or 2 diabetes
  - □ Gestational diabetes
  - □ Obesity (body mass index ≥30 kg/m2) prior to pregnancy
  - □ Depression or anxiety disorder

SECTION 3. Consent

301: READ OUT LOUD TO PATIENT: I am a Research Assistant with the MGH Bridge Study. Our research team thinks you might be eligible to participate in a study to improve your health after your delivery.<br>May we have your permission to tell you more about the study and ask you some questions?<br>1. Yes -> **Review Study Information Sheet**<br>2. No -> **STOP, if respondent would not like to participate: End Survey.**

302: After Study Information Sheet reviewed, indicate whether consent was granted.<br>1. Consented for overall study AND text messaging -> **Skip to next section**<br>2. Consented for overall study but NOT text messaging -> **Skip to next section**<br>3. Did not consent
303. RATIONALE FOR DECLINING CONSENT: Would you like to share why you do not want to participate in this study? If so, please tell us in your own words why you did not choose to participate.<br>1. Did not give a reason/no response<br>2. Rationale for declining: ____

SECTION 4. Pre-Fill & Verification of Respondent Information
*Instructions: Pre-fill **bolded** items based on Patient Record before approaching the respondent. Please make sure to ask the patient to verify or update this information.*

401. **Respondent First Name:**<br>a. Confirmed? Yes ___ No ____<br>b. If No, specify correct information:
402. **Respondent Last Name:**<br>a. Confirmed? Yes ___ No ____<br>b. If No, specify correct information:
403. **Respondent Medical Record Number:**
404. **Respondent Home Address:** ☐ None listed<br>a. Confirmed? Yes ___ No ____<br>b. If No, specify correct information:
405. **Respondent Mobile Number:** ☐ None listed<br>a. Confirmed? Yes ___ No ____<br>b. If No, specify correct information:
406. **Respondent Email Address:** ☐ None listed<br>a. Confirmed? Yes ___ No ____<br>b. If No, specify correct information:<br>c. How often does the respondent check this email address? Rarely___ Monthly___ Weekly___ Daily___
407. **Respondent Preferred Language:** ☐ None listed<br>a. Confirmed? Yes ___ No ____<br>b. If No, specify correct information:
408. **Respondent Race (tick all that apply)**<br>i. White ______<br>ii. Black or African American ______<br>iii. American Indian or Alaska Native ______<br>iv. Asian ______<br>v. Native Hawaiian or Other Pacific Islander ______<br>vi. Other, please specify ______<br>vii. Declined ______<br>viii. Unavailable ______<br><br>a. Confirmed? Yes ___ No ____<br>b. If No, specify correct information:<br>c. No race provided

409. **Respondent Ethnicity:**<br>i. Hispanic or Latino ______<br>ii. Not Hispanic or Latino ______<br>iii. Prefer not to say/Decline ______<br>iv. Unavailable ______<br><br>a. Confirmed? Yes ___ No ____<br>b. If No, specify correct information:<br>c. No ethnicity provided
410. **Primary Care Provider Name:**<br>a. Confirmed? Yes ___ No ____ Patient does not know/unsure__________<br>b. If No, specify correct information: _______________<br>Notes:
411. **Primary Care Provider Location/Address:**<br>a. Confirmed? Yes ___ No ____ Patient does not know/unsure__________<br>b. If No, specify correct information: _______________<br>Notes:
412. Patient Gateway Access<br>How easy or difficult is it for you to access the Patient Gateway portal?<br>o Very easy to access<br>o Somewhat easy to access<br>o Neither easy nor difficult<br>o Somewhat difficult to access<br>o Very difficult to access

SECTION 5. Health and Health Care Use

501. Before your pregnancy, would you say that in general your PHYSICAL health was:<br>1. Excellent<br>2. Very good<br>3. Good<br>4. Fair<br>5. Poor<br>88. Don't know/not sure<br>99. Refused
502. Before your pregnancy, would you say that in general your MENTAL health was:<br>1. Excellent<br>2. Very good<br>3. Good<br>4. Fair<br>5. Poor<br>88. Don't know/not sure<br>99. Refused
503. In the year before your pregnancy, if you had a medical issue or concern, how easy or difficult was it for you to see a health care provider in a clinic (not for an emergency)?<br>1. Very easy

2. Somewhat easy<br>3. Neither easy nor difficult<br>4. Somewhat difficult<br>5. Very difficult<br>6. I did not try to receive health care prior to pregnancy<br>88. Don't know/not sure<br>99. Refused
504. There are many reasons people delay or avoid getting medical care. Please select any of the following reasons that have caused you to not get the care you needed before your pregnancy.<br>1. Didn't know who to make an appointment with.<br>2. Couldn't get an appointment<br>3. Didn't have transportation<br>4. The clinic or doctor's office wasn't open when you could get there<br>5. Once you got to the appointment, the wait was too long to see the doctor<br>6. You were worried about the financial cost of care<br>7. You couldn't get time off of work to get care<br>8. You didn't think that the medical care would help<br>9. You didn't have childcare<br>10. Other, please specify ______<br>88. Don't know/not sure<br>99. Refused
505. How do you usually get to medical appointments? **Tick all that apply.**<br>1. Personal car<br>2. Taxi, Uber, or Lyft<br>3. Bus<br>4. Subway ("T")<br>5. Train<br>6. Bicycle<br>7. On foot/walking<br>8. Other, please specify ______<br><br>99. Refused
506. In the year before your pregnancy, did you receive care at any clinics (outpatient appointments) that were NOT a part of Mass General Hospital or the Mass General Brigham health system (including primary care visits, specialty visits, urgent care visits)?<br>1. Yes<br>2. No<br>3. I did not have any appointments in the year before my pregnancy<br>88. Don't know/not sure<br>99. Refused

507-510: Have you ever been told by a doctor, nurse, or other health professional that you have or are:

Condition	Yes	No	Not sure
High blood pressure	◯	◯	◯

or hypertension			
Diabetes or high blood sugar	◯	◯	◯
Anxiety or a depressive disorder (including depression, major depression, dysthymia, or minor depression)	◯	◯	◯
Overweight or obese	◯	◯	◯

SECTION 6. **Postpartum / Leave**

601. At any time during your pregnancy have you worked for pay in a job located in Massachusetts?
1. Yes
2. No **-> Skip to 603**
88. Don't know/not sure **-> Skip to 603**
99. Refused **-> Skip to 603**

602. Which best describes the type(s) of work you have done during your pregnancy? (Check all that apply. List name of employer)
1. Federal or State Government.______ Name:___________________________
2. Private or For-Profit Company: .______ Name:___________________________
3. Municipality (school dept, public works, housing authority, regional school district):.______ Name:___________________________
4. Church or Religious Organization_______ Name: ______________________
5. Self-employed ______________ Name: _______________________
6. I don't know which of these categories my employer falls into
7. Other: _______________
88. Don't know/not sure
99. Refused

603. Do you plan to return to work or start a new job within the first year after your baby is born?
1. Yes, return to previous job
2, Yes, start a new job
3. No **-> Skip to 606**
88. Don't know/not sure **-> Skip to 606**
99. Refused **-> Skip to 606**

604. How long after the baby is born do you intend to return to work/start working?
_________Weeks
_________ Months

88. Don't know/not sure
99. Refused

605. Do you intend to take any fully or partially paid leave from your job after your baby is

born?
1. Yes
2. No
88. Don’t know/not sure
99. Refused

606. Have you ever seen, read, or heard anything about a Massachusetts state program called “Paid Family and Medical Leave“ (PFML)? This program provides eligible workers with paid time off for family or medical reasons.

1.Yes, I have seen, read, or heard about the Massachusetts PFML program.
2.No, I have not seen, read, or heard about the Massachusetts PFML program. **-> Skip to next section**
88. Don’t know/not sure **-> Skip to next section**
99.Refused **-> Skip to next section**

607. Do you intend to apply for the Massachusetts Paid Family Medical Leave program?
1. Yes
2. No. State reason why: ______________________________
88. Don’t know/not sure
99. Refused

SECTION 7. Additional Demographic Information

701. What is your marital status?
1. Married
2. Divorced
3. Widowed
4. Separated
5. Never married
6. Member of an unmarried couple
99. Refused

702. Do you anticipate not having or losing health insurance coverage in the year after your baby is born?
1. Yes
2. No
88. Don’t know/not sure
99. Refused

703. Do you expect your primary insurance provider to change after your pregnancy?
1. Yes
2. No
88. Don’t know/not sure
99. Refused

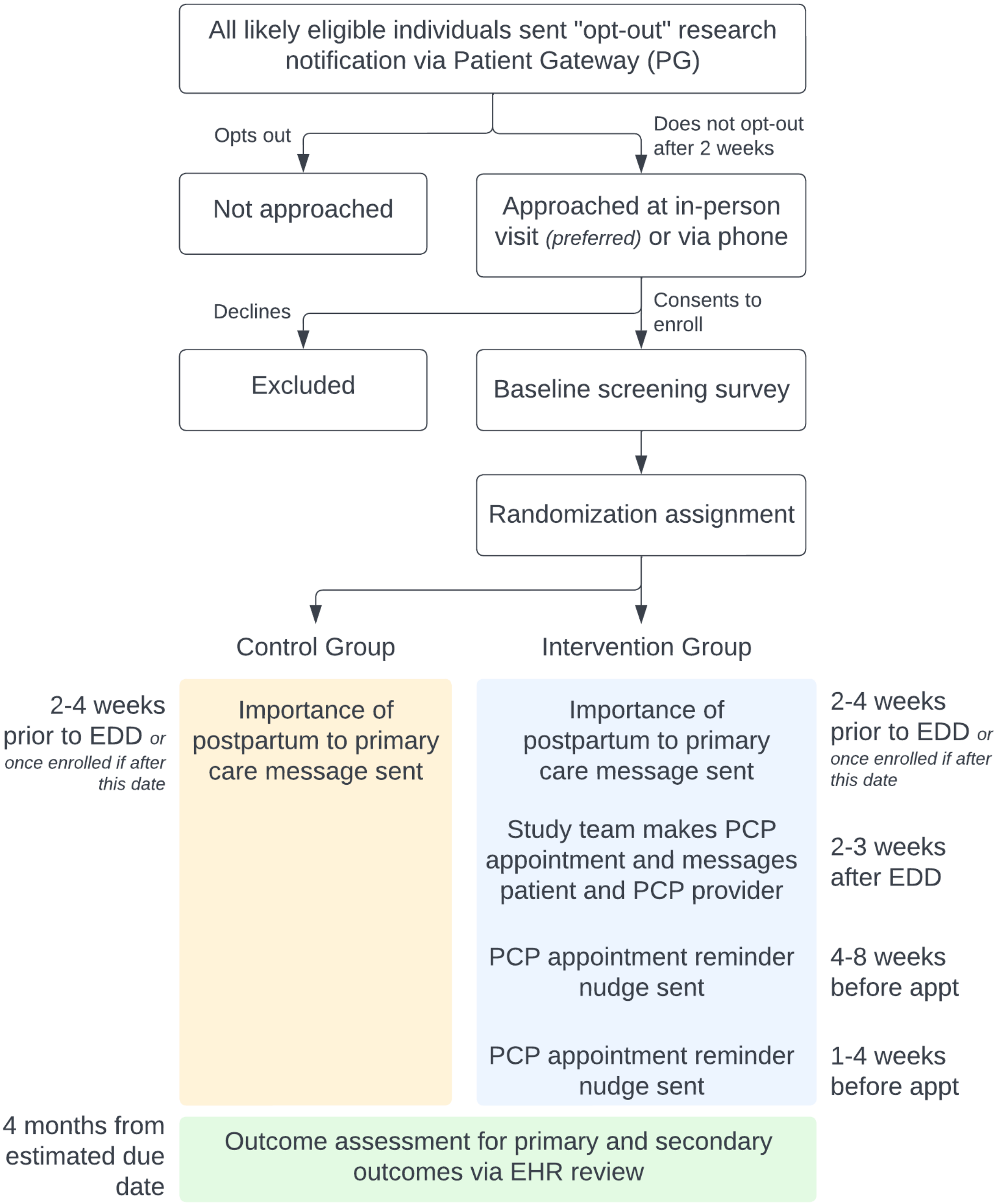
All likely eligible individuals sent "opt-out" research notification via Patient Gateway (PG)
Opts out
Does not opt-out after 2 weeks
Not approached
Approached at in-person visit *(preferred)* or via phone
Declines
Consents to enroll
Excluded
Baseline screening survey
Randomization assignment
Control Group
Intervention Group
2-4 weeks prior to EDD *or once enrolled if after this date*
Importance of postpartum to primary care message sent
Importance of postpartum to primary care message sent
2-4 weeks prior to EDD *or once enrolled if after this date*
Study team makes PCP appointment and messages patient and PCP provider
2-3 weeks after EDD
PCP appointment reminder nudge sent
4-8 weeks before appt
PCP appointment reminder nudge sent
1-4 weeks before appt
4 months from estimated due date
Outcome assessment for primary and secondary outcomes via EHR review

Mass General Brigham

Title: Bridging the Gap from Postpartum to Primary Care: A Behavioral Science Informed Intervention to Improve Chronic Disease Management among Postpartum Women (the Bridge Study)

Sponsor Name:

PI Name: Clapp, Mark A **Protocol #:** 2022P001723 **Type:** Amendment (AME3)

Date Received: October 06, 2022

Signatures

PI Name: Clapp, Mark A, MD, MPH

Authenticated: October 06, 2022

Amendment

COVID-19 Amendment

Is this amendment ONLY related to research impacted by COVID-19?

Refer to MGB Policy on Conduct of Human Research Activities during COVID-19 Operations for description of Amendments which do **require prior IRB review and approval.**

- ◯ Yes
- ◉ No

Central IRB Performance Sites

Is this a protocol where the Mass General Brigham IRB is serving as the single IRB (sIRB) for external sites/institutions?

- ◯ Yes
- ◉ No

Sponsor Amendment

Is there a sponsor amendment number?

- ◯ Yes
- ◉ No

Change in Protocol Status

Is this a cede protocol or project that was determined to be exempt, not human subjects research or not engaged in human subjects research?

- ◯ Yes
- ◉ No

Do you need to change the overall status of the protocol? For example, Re-Open to Enrollment or indicate that Research Interventions/Assessments Continue after telling the IRB these have ceased.

- ◯ Yes
- ◉ No

Briefly describe the proposed changes:

Mass General Brigham

Updated baseline survey after initial piloting

Provide rationale for the proposed changes:

The baseline survey questions ordering and language have been updated after additional piloting with non-study subjects. The content and purpose of the baseline survey did not change. Non-patient-facing survey questions or tasks (e.g., instructions for survey staff) were removed.

Will the proposed change(s) significantly alter the risk to benefit assessment the IRB relied upon to approve the protocol?

- ◯ Yes
- ◉ No

Will the proposed change(s) significantly affect the integrity of the protocol?

- ◯ Yes
- ◉ No

Informed Consent

Do the changes require a revision to the consent form?

- ◯ Yes
- ◉ No

Attachments

Name	Mode
Baseline_10-5-22_final (Instrument/Questionnaire)	Electronic

Bridging the Gap from Postpartum to Primary Care

Study PIs: Mark Clapp, MD MPH (MGH); Jessica Cohen, PhD (Harvard)

Site PI: Mark Clapp, MD MPH (MGH)

SECTION 5. Patient Survey

All information will be kept private and will not affect any services you are now getting.

701. What is your marital status?<br>1. Married<br>2. Divorced<br>3. Widowed<br>4. Separated<br>5. Never married<br>6. Member of an unmarried couple<br>88. Don’t know/not sure<br>99. Prefer not to say
702. What option best describes the highest degree or level of school you have completed?<br>1. 8th grade or less<br>2. 9th - 12th grade, no diploma<br>3. High school graduate or GED completed<br>4. Some college credit but no degree<br>5. Associate degree (e.g., AA, AS)<br>6. Bachelor’s degree (e.g., BA, AB, BS)<br>7. Master’s degree (e.g., MA, MS, MEng, MEd, MSW, MBA)<br>8. Doctorate (e.g., Ph.D., EdD) or Professional degree<br>88. Don’t know/not sure<br>99. Prefer not to say

703. In the past 12 months, approximately how much money have you individually earned?

1. $0

2. $1 – $6,000

3. $6,001 – $10,000

3. $10,001 – $30,000

4. $30,001 – $50,000

5. $50,001 – $75,000

6. $75,001 – $100,000

7. > $100,001

88. Don't know/not sure

99. Prefer not to say

704. Do you anticipate losing or going without health insurance the year after your baby is born?

1. Yes

2. No

88. Don't know/not sure

99. Prefer not to say

705. Do you expect your primary insurance provider to change after your pregnancy?

1. Yes

2. No

88. Don't know/not sure

99. Prefer not to say

501. How easy or difficult is it for you to access the Patient Gateway portal?

1. Very easy

2. Somewhat easy

3. Neither easy nor difficult

4. Somewhat difficult

5. Very difficult

88. Don't know/not sure

99. Prefer not to answer

502. In the year **BEFORE** your pregnancy, how would describe your PHYSICAL health?<br>1. Excellent<br>2. Very good<br>3. Good<br>4. Fair<br>5. Poor<br>88. Don't know/not sure<br>99. Prefer not to answer
503. In the year **BEFORE** your pregnancy, how would describe your MENTAL health?<br>1. Excellent<br>2. Very good<br>3. Good<br>4. Fair<br>5. Poor<br>88. Don't know/not sure<br>99. Prefer not to answer
504. Thinking about the year before your pregnancy, if you needed to see a health care provider for a non-emergency medical issue, how easy or difficult was it for you?<br>1. Very easy<br>2. Somewhat easy<br>3. Neither easy nor difficult<br>4. Somewhat difficult<br>5. Very difficult88. Don't know/not sure<br>99. Prefer not to answer
505. There are many reasons people delay or avoid getting medical care. Did any of the following issues cause you to not get the medical care you needed before your pregnancy? *Please read each option aloud*. *When finished, ask "Anything else?".*<br>1. Unsure who to make an appointment with<br>2. Difficulty getting an appointment<br>3. Difficulty arranging transportation

4. The clinic or doctor’s office wasn’t open when you could get there<br>6. Concern about the financial cost of care<br>7. Difficulty obtaining time off work to get care<br>8. Unsure that the medical care would help<br>9. Difficulty getting childcare<br>10. Other, please specify ______<br>88. Don’t know/not sure<br>99. Prefer not to answer
505. Do you have a personal car?<br>1. Yes<br>2. No<br>88. Don’t know/not sure<br>99. Prefer not to answer
506. What was your mode of transportation to your appointment today? *Select all that apply*.<br>1. Personal car<br>2. Taxi, Uber, or Lyft<br>3. Car of a friend or family member<br>4. Bus<br>5. Subway/Train (“T”)<br>6. Bicycle<br>7. On foot/walking<br>8. Other, please specify ______<br>88. Don’t know/not sure<br>99. Prefer not to answer
505. Do you have a personal car?<br>1. Yes<br>2. No<br>88. Don’t know/not sure<br>99. Prefer not to answer

507. In the year before your pregnancy, other than visits to the emergency room, did you see a doctor or nurse for any reason? This does not include visits to the emergency room but can include visits with your primary care doctor, specialty care doctor, urgent care, etc.<br>1. Yes, please specify where________<br>2. No<br>88. Don’t know/not sure<br>99. Prefer not to answer

508-511: Have you ever been told by a doctor, nurse, or other health professional that you have or are:

Condition	Yes	No	Don’t Know/Not sure	Prefer not to Answer
High blood pressure or hypertension	◯	◯	◯	◯
Diabetes or high blood sugar	◯	◯	◯	◯
Anxiety or depression	◯	◯	◯	◯
Overweight or obese	◯	◯	◯	◯

SECTION 6. **Employment and Postpartum Leave**

601. Are you currently working for pay?<br>1. Yes → **Skip to 603**<br>2. No<br>88. Don’t know/not sure<br>99. Prefer not to say
602. Have you worked for pay at any time in the past 12 months?<br>1. Yes

2. No → **Skip to 604**<br>88. Don't know/not sure<br>99. Prefer not to say
603. Which best describes the type(s) of work you have done the past 12 months? (*Check all that apply. List name of employer. For each employer listed, ask "Is this employer in Massachusetts?"*).<br>1. Federal or State Government:_____ Name:__________________________<br>In Massachusetts? Yes: _____ No: _______<br>2. Private or For-Profit Company: _____ Name:__________________________<br>In Massachusetts? Yes: _____ No: _______<br>3. Municipality (school dept, public works, housing authority, regional school district):_____ Name:__________________________<br>In Massachusetts? Yes: _____ No: _______<br>4. Church or Religious Organization______ Name: _____________________<br>In Massachusetts? Yes: _____ No: _______<br>5. Self-employed _____________ Name: ______________________<br>In Massachusetts? Yes: _____ No: _______<br>6. Independent Contractor _____________ Name: ______________________<br>In Massachusetts? Yes: _____ No: _______<br>7. Other: ______________<br>In Massachusetts? Yes: _____ No: _______<br>88. Don't know/not sure<br>99. Prefer not to say
604. Do you plan to return to work **or** start a new job within the first year after your baby is born?<br>1. Yes, return to previous job<br>2, Yes, start a new job<br>3. No **-> Skip to 606**<br>88. Don't know/not sure<br>99. Prefer not to say
605. How long after the baby is born do you intend to return to work/start working?<br>1. Less than one week<br>2. 1-4 weeks<br>3. 5-8 weeks<br>4. 9-12 weeks

5. 13-16 weeks<br>6. 17-20 weeks<br>7. 21-24 weeks<br>8. 25 weeks or more<br>88. Don’t know/not sure<br>99. Prefer not to say
606. Have you ever seen, read, or heard anything about a Massachusetts state program called “Paid Family and Medical Leave“ (PFML)? This program provides eligible workers with paid time off for family or medical reasons.<br>1.Yes, I have seen, read, or heard about the Massachusetts Paid Family and Medical Leave program.<br>2.No, I have not seen, read, or heard about the Massachusetts Paid Family and Medical Leave program.<br>88. I don't know/am not sure about the Massachusetts Paid Family and Medical Leave program.<br>99. Prefer not to say
607. Do you intend to apply for the Massachusetts Paid Family Medical Leave program?<br>1. Yes<br>2. No. State reason why: ____________________________<br>88. Don’t know/not sure<br>99. Prefer not to say

Mass General Brigham

Title: Bridging the Gap from Postpartum to Primary Care: A Behavioral Science Informed Intervention to Improve Chronic Disease Management among Postpartum Women (the Bridge Study)

Sponsor Name:

PI Name: Clapp, Mark A **Protocol #:** 2022P001723 **Type:** Amendment (AME4)

Date Received: October 12, 2022

Signatures

PI Name: Clapp, Mark A, MD, MPH

Authenticated: October 12, 2022

Amendment

COVID-19 Amendment

Is this amendment ONLY related to research impacted by COVID-19?

Refer to MGB Policy on Conduct of Human Research Activities during COVID-19 Operations for description of Amendments which do **require prior IRB review and approval.**

- ◯ Yes
- ◉ No

Central IRB Performance Sites

Is this a protocol where the Mass General Brigham IRB is serving as the single IRB (sIRB) for external sites/institutions?

- ◯ Yes
- ◉ No

Sponsor Amendment

Is there a sponsor amendment number?

- ◯ Yes
- ◉ No

Change in Protocol Status

Is this a cede protocol or project that was determined to be exempt, not human subjects research or not engaged in human subjects research?

- ◯ Yes
- ◉ No

Do you need to change the overall status of the protocol? For example, Re-Open to Enrollment or indicate that Research Interventions/Assessments Continue after telling the IRB these have ceased.

- ◯ Yes
- ◯ No

Briefly describe the proposed changes:

1) Adding Spanish versions of the IRB-approved documents (study invitation letter, patient messages, study fact sheet)

2) Added clinicaltrials.gov registration number

Provide rationale for the proposed changes:

To be able to send materials in individuals' preferred language

---

Will the proposed change(s) significantly alter the risk to benefit assessment the IRB relied upon to approve the protocol?

○ Yes
◉ No

Will the proposed change(s) significantly affect the integrity of the protocol?

○ Yes
◉ No

Informed Consent

Do the changes require a revision to the consent form?

○ Yes
◉ No

Clinical Trials Registration

Clinical Trials Registration and Results Reporting

Investigator–initiated clinical trials must be registered on ClinicalTrials.gov to comply with federal FDA requirements in FDA 42 CFR 11 (Final Rule) and/or NIH Policy. Studies that falls under both FDA and NIH requirements only need to be registered once. The information posted on ClinicalTrials.gov (CT.gov) must be updated and verified at least every 12 months.

The following information is used to identify studies that require clinical trials registration and results reporting, and to inform Principal Investigators (PI) of their responsibilities for registration and results reporting.

IMPORTANT NOTE: Even if your investigator-initiated clinical trial does not meet the NIH or FDA clinical trials registration requirements, you are strongly advised to read and consider registering your trial to comply with the following additional requirements:

- **A non-federal sponsor may require registration as part of the award's terms and conditions**
- **International Committee of Medical Journal Editors (ICMJE) for publication purposes**
- **Center for Medicare & Medicaid for research billing claims for qualifying clinical trials (Mass General Brigham Clinical Trials Office will notify you if applicable)**
- **Research funders now requiring registration and results reporting: May 18, 2017 Joint Statement**

For additional information, please see the Human Research Affairs Compliance and Education Office website for Clinical Trials Registration.

Is this a Mass General Brigham investigator – initiated research study?

- ○ No
- ◉ Yes

Is this research funded in whole or in part by NIH **AND** does this research meet the NIH's definition of **clinical trial**?

NIH defines a clinical trial as any research study that meets all of the following criteria:

- The study involves human participants;
- The participants are prospectively assigned to an intervention;
- The study is designed to evaluate the effect of the intervention on participants; **AND**
- The effect being evaluated is a health-related, biomedical or behavioral outcome.

- ○ No
- ◉ Yes

The responses to the questions above indicate this study meets the NIH Policy requirements for clinical trials registration and results reporting. Mass General Brigham Institutions have delegated responsibility for clinical trials registration, periodic updates, and results and adverse event reporting to the Principal Investigator ("Responsible Party"). This study must be registered on ClinicalTrials.gov prior to the first subject being enrolled into the study.

Additional information is available from the NIH:

- **NIH's Definition of a Clinical Trial | grants.nih.gov**
- **NIH Definition of Clinical Trial Case Studies | grants.nih.gov**

Responsible Party

Enter Name of MGB investigator who will be the Responsible Party, for example Jane Doe, MD:

Mark Clapp, MD

Enter the full name of the MGB Institution of the MGB investigator, for example, Massachusetts General Hospital:

Massachusetts General Hospital

Mass General Brigham

Documentation of ClinicalTrials.gov Registration

Have you submitted registration information to ClinicalTrials.gov?

- ◯ No
- ◉ Yes

Indicate if a National Clinical Trial (NCT) registration number has been assigned:

- ◉ Assigned
- ◯ Pending

Enter the NCT number; for example NCT12345678:

NCT05543265

Results Reporting

ClinicalTrials.gov results reporting is required within 12 months of the primary endpoint completion date defined as the date that the final subject was examined or received the intervention for the purposes of collection of primary outcome. Early consultation is strongly advised. Please contact the HRA Compliance and Education Office for assistance:

Mass General Brigham Human Research Affairs Compliance and Education Office:
QIProgramCTgovTeam@partners.org

Additional information can be found at:
HRA Compliance and Education Office website for **Clinical Trials Registration**
www.ClinicalTrials.gov

Attachments

Name	Mode
Study Fact Sheet_090122_Spanish (Consent Fact/Information Sheet)	Electronic
Letter of Certification_P-2022-3783 (Other)	Electronic
Patient Gateway Research Invitation Letter_Spanish (Recruitment Letter)	Electronic
Patient Messages_final_Spanish (Document for review)	Electronic

Mass General Brigham

Documento informativo del estudio de investigación

Cerrando la brecha entre el posparto y la atención primaria (Bridge Study)

Investigadores principales	Mark Clapp, MD MPH (Massachusetts General Hospital)<br>Jessica Cohen, PhD (Harvard T. H. Chan School of Public Health)
Descripción general	Este es un estudio de investigación que se realiza entre mujeres embarazadas y que han dado a luz recientemente y que reciben atención en MGH.
Objetivo	Su proveedor de atención primaria (PCP) es responsable de manejar y supervisar su salud actual y a largo plazo. El objetivo de este proyecto es mejorar la atención primaria en el período de posparto.
Patrocinador	Esta investigación está patrocinada por el Instituto Nacional del Envejecimiento (*National Institute of Aging*, NIA), el Centro Roybal del MIT para la Investigación Traslacional para Mejorar la Atención Médica de los Adultos Mayores (*MIT Roybal Center for Translational Research to Improve Health Care for the Aging*) y el Centro Roybal del NBER para el Cambio de Comportamiento en la Salud (*NBER Roybal Center for Behavior Change in Health*).
Contacto	Le hemos enviado un mensaje de Patient Gateway sobre este estudio porque usted es una persona que puede beneficiarse especialmente de recibir atención primaria de posparto.
Detalles del estudio	Si acepta participar, le haremos algunas preguntas sobre su salud y su historial de visitas en el momento de la inscripción, todas ellas opcionales.<br><br>Entonces se la asignará aleatoriamente (50/50 de probabilidades) para participar en el grupo de “atención estándar” o en el grupo de “transición asistida”. Independientemente del grupo al que se la asigne, seguirá recibiendo atención prenatal y posparto estándar por parte de su proveedor de atención obstétrica.<br><br>En el grupo de “atención estándar”, recibirá una cantidad limitada de mensajes (menos de 5) del equipo del estudio.<br><br>En el grupo de “transición asistida”, recibirá una cantidad limitada de mensajes (menos de 5) del equipo del estudio entre el momento de la inscripción y hasta cuatro meses después del parto. El equipo del estudio también la ayudará a comunicarse con su proveedor de atención primaria después del parto. Específicamente, podemos llamar al consultorio de su proveedor de atención primaria de su parte para ayudar a programar una cita. Usted siempre tiene la opción de cambiar o cancelar esta cita. También le enviaremos un mensaje a su PCP para informarle que ha dado a luz recientemente y que se está programando una cita de seguimiento.<br><br>Los mensajes que le enviemos a través de Patient Gateway también figurarán en su historia clínica para que los demás miembros de su equipo de atención los vean.<br><br>Para ambos grupos, revisaremos su historia clínica después de su parto para ver si recibió y cuándo recibió algún tipo de atención dentro del sistema de salud de Mass General Brigham e información sobre su estado de salud hasta dos años después de su parto.<br><br>También es posible que nos comuniquemos con usted una vez entre cuatro y doce meses después de su parto para hacerle preguntas sobre su experiencia de atención posparto. Puede negarse a participar o a responder a alguna o a todas las preguntas.

	Puede optar por no recibir mensajes o negarse a participar en cualquier encuesta, y/o retirarse del estudio en cualquier momento sin recibir ninguna sanción.<br><br>En este estudio, participarán aproximadamente 350 personas.
Privacidad/ Confidencialidad	La Ley de Portabilidad y Responsabilidad de Seguros Médicos (*Health Insurance Portability and Accountability Act*, HIPAA) nos exige que protejamos la privacidad de la información de salud obtenida para la investigación. En las páginas 3 y 4 de este documento informativo se incluyen más detalles relacionados con la privacidad de su información de salud.<br><br>Se mantendrá la confidencialidad y la seguridad de toda la información recopilada como parte de este estudio. Como en todos los estudios, existe un riesgo muy bajo de que otras personas puedan conocer su participación o su información de salud. El Comité de Investigación en Seres Humanos de Mass General Brigham ha aprobado los procedimientos del estudio y la protección de su información. Una vez finalizado el estudio, se eliminará toda la información que pudiera identificarla, como por ejemplo su nombre o número de historia clínica.
Información del estudio	Recopilaremos información sobre sus antecedentes de salud y embarazo, incluso sobre las visitas que realice a los proveedores del sistema de salud de Mass General Brigham. Los datos recopilados como parte de este estudio no afectarán de manera directa su atención clínica. Usted y su equipo de atención podrán ver todos los mensajes enviados por el equipo del estudio en la historia clínica electrónica. La información sobre usted sin identificación se puede usar o compartir con otros investigadores sin su consentimiento informado adicional.
Riesgos	Los riesgos de participar son mínimos.<br><br>Aunque haremos todo lo posible para proteger la privacidad y confidencialidad de los participantes, es posible que otras personas conozcan su participación en el estudio. Para comunicarnos con usted, le enviaremos mensajes a través de Patient Gateway, que es un método que cumple con la HIPAA. Usted tiene la opción de recibir también mensajes de texto SMS que no incluirán ninguna información de salud personal o que permita identificarla. Existe una posibilidad poco frecuente de que otras personas conozcan su participación en el estudio o la información sobre su salud, a pesar del uso de cortafuegos, protección con contraseña y otras medidas de seguridad.
Costos	No está obligada a pagar para participar en este estudio de investigación.<br><br>Puede haber un costo para que usted asista a una visita con un PCP, como por ejemplo el pago de un copago o un deducible. Sus costos se basan en la cobertura de su seguro individual. Si no tiene seguro, es posible que se le facture de manera directa la totalidad del costo de esta visita. Le proporcionaremos información sobre cómo comprobar los posibles costos con su aseguradora o con el consultorio de su PCP antes de su visita.<br><br>Algunas clínicas pueden sancionar a las personas que no se presenten a una cita programada (tarifa por “no presentarse”). Se le dará la oportunidad de optar por no ser citada y se le recordará varias veces que debe cancelar o reprogramar la cita si no puede asistir.

Participación	Aquellas personas que participen recibirán una tarjeta de regalo de $20 después de la inscripción.<br><br>La participación es voluntaria, y puede retirarse o suspender el estudio en cualquier momento.<br>Su decisión de no participar no afectará la atención médica que reciba en Mass General Brigham ahora o en el futuro ni ningún beneficio que reciba ahora o que tenga derecho a recibir.
Contacto del estudio	Mark Clapp, MD MPH, es la persona de MGH a cargo de este estudio de investigación. Puede llamarle al 617-724-4531 (de lunes a viernes de 9 a. m. a 5 p. m.). También puede llamar a la coordinadora del estudio (Fowsia Warsame) al 617-643-5483 (de lunes a viernes, de 9 a. m. a 5 p. m.), si tiene preguntas sobre este estudio de investigación. También puede enviar un correo electrónico a bridgestudy@mgh.harvard.edu.<br><br>Si desea hablar con alguien que no participe en esta investigación acerca de sus derechos como sujeto de una investigación o sobre cualquier preocupación o queja que pueda tener respecto a la investigación, comuníquese con el IRB de Mass General Brigham IRB al (857) 282-1900.

Opción de recibir mensajes de texto (SMS)

Los mensajes de texto por teléfonos móviles/celulares son una forma común de comunicación. A nuestro equipo del estudio le gustaría enviarle no más de 3 mensajes de texto (SMS) como parte del estudio, aunque **esto es opcional y no se requiere para participar en Bridge Study.** El envío de mensajes de texto por teléfonos móviles/celulares implica riesgos de seguridad debido a que tales mensajes de texto no están cifrados. Esto significa que la información que usted envíe o reciba por mensajes de texto podría ser interceptada o vista por un destinatario no deseado, o bien por su proveedor o compañía de servicios de telefonía móvil/celular.

A continuación se presentan algunos puntos importantes sobre el envío de mensajes de texto como parte de un estudio de investigación:

- Recibir mensajes de texto en este estudio es opcional y no es obligatorio para participar.
- Los mensajes de texto no están cifrados y, por lo tanto, presentan riesgos de seguridad. Ni este estudio de investigación ni Mass General Brigham son responsables de la interceptación de los mensajes enviados mediante comunicaciones por mensajes de texto no cifrados.
- Usted será responsable de todas las tarifas que se le cobren según el plan de servicios de mensajes de texto de su compañía de servicios de telefonía. Ni este estudio de investigación ni Mass General Brigham son responsables de los aumentos de los cargos, del uso de los datos en función de los límites del plan ni de los cambios en las tarifas de datos de los mensajes de texto de la investigación.
- No se debe enviar mensajes de texto en caso de emergencia. Si tiene una emergencia médica, llame al 911 o vaya al departamento de emergencias del hospital más cercano.
- En cualquier momento, puede decidir no comunicarse con el personal asociado a este estudio de investigación mediante el envío o la recepción de mensajes de texto. Puede hacerlo personalmente o enviar un mensaje de texto que diga “Stop Research Text” (Detener mensajes de texto de la investigación).
- Su aceptación se aplica a este estudio de investigación solamente. La aceptación de otros mensajes de texto de Mass General Brigham, como por ejemplo los recordatorios de citas, corresponde a un proceso independiente. La opción de no recibir otros mensajes de texto de Mass General Brigham también representa un proceso independiente.
- Es su responsabilidad actualizar su número de teléfono móvil/celular en este estudio de investigación si se produce algún cambio.

Información sobre la privacidad de su información de salud

Las leyes federales exigen que Mass General Brigham y sus hospitales afiliados protejan la privacidad de la información de salud y la información relacionada que la identifica. Nos referimos a esa información como “información de salud protegida”. Se usará y compartirá la información de salud protegida sobre usted con otras personas como se explica a continuación. Usted acepta que se recopile, utilice y comparta la información de salud protegida sobre usted tal y como se describe en esta hoja informativa. Si tiene preguntas, puede preguntarle al investigador que está revisando este documento informativo con usted o puede comunicarse con el investigador indicado arriba.

En este estudio, podemos recopilar información de salud protegida sobre usted proveniente de:

- Historia clínica pasada, presente y futura
- Procedimientos de investigación, que incluyen, entre otros, visitas para la investigación, pruebas, entrevistas y cuestionarios

<u>¿Por qué se utilizará o compartirá con terceros la información de salud protegida sobre usted?</u>
Las principales razones incluyen:

- llevar a cabo y supervisar la investigación descrita en este documento informativo para este estudio;

- garantizar que la investigación cumpla con los requisitos legales, institucionales y de acreditación; y
- llevar a cabo actividades de salud pública (incluida la notificación de acontecimientos adversos o situaciones en las que usted u otras personas puedan correr riesgo de sufrir daños).

¿Quiénes pueden ver, usar y compartir la información de salud protegida sobre usted y por qué es necesario que lo hagan?

- Los investigadores y el personal de Mass General Brigham que participan en este estudio
- El o los patrocinadores del estudio y las personas o los grupos que ellos contraten para ayudar a realizar o auditar la investigación
- Otros investigadores e instituciones médicas que participen en este estudio
- La junta de ética de Mass General Brigham
- Un grupo que supervise los datos (la información del estudio) y la seguridad de este estudio
- El personal de Mass General Brigham que no se dedica a la investigación y que necesita información que permite la identificación para realizar su trabajo, por ejemplo para el tratamiento, el pago (facturación) o las operaciones del hospital (como la evaluación de la calidad de la atención o la investigación)
- Personas o grupos que contratemos para hacer ciertos trabajos para nosotros, tales como empresas de almacenamiento de datos, agentes autorizados, aseguradoras y abogados
- Agencias federales como el Departamento de Salud y Servicios Sociales (*Department of Health and Human Services*, DHHS) y organismos dentro de DHHS como la Administración de Alimentos y Medicamentos (*Food and Drug Administration,* FDA), los Institutos Nacionales de Salud (*National Institutes of Health*, NIH) y la Oficina para la Protección de Seres Humanos que Participan en Investigaciones (*Office for Human Research Protections*), entidades gubernamentales extranjeras que supervisan, evalúan y auditan la investigación, lo que puede incluir la inspección de los registros.
- Autoridades de salud y seguridad públicas, si obtenemos información que podría significar un daño para usted o para otras personas (como realizar los informes obligatorios sobre enfermedades contagiosas o sobre abuso infantil o de adultos mayores).

Es posible que algunas personas o grupos que obtengan la información de salud protegida sobre usted no tengan que cumplir con las mismas normas de privacidad que cumplimos nosotros y podrían usar o compartir la información de salud protegida sobre usted sin su permiso de maneras que no se detallan en este formulario. Compartimos la información de salud protegida sobre usted solamente cuando debemos hacerlo, y solicitamos que quienes la reciban tomen medidas para proteger su privacidad. Sin embargo, una vez que la información de salud protegida sobre usted se comparta fuera de Mass General Brigham, no podremos controlar todas las maneras en las que otras personas o entidades la usen o la compartan, y no podemos prometer que se mantendrá en privado.

Los resultados de este estudio de investigación pueden publicarse en un libro o una revista médica, o usarse para enseñar a otros. Sin embargo, **no** se usará su nombre ni otra información que permita identificarla con este fin sin su permiso específico.

¿Durante cuánto tiempo se utilizará o compartirá con terceros la información de salud protegida sobre usted?
Debido a que la investigación es un proceso continuo, no podemos darle una fecha exacta en la que destruiremos o dejaremos de usar o de compartir la información de salud protegida sobre usted. El permiso que usted nos ha dado para usar y compartir la información de salud protegida sobre usted no vence.

Sus derechos de privacidad
Tiene derecho a no aceptar que utilicemos y compartamos la información de salud protegida sobre usted para investigaciones. Sin embargo, si no está de acuerdo, no podrá participar en este estudio de investigación. Sin embargo, negarse a aceptar no afectará su atención presente o futura y no le ocasionará ninguna sanción o pérdida de beneficios a los que de otro modo tiene derecho.

Tiene derecho a retirar su permiso para que se siga utilizando o compartiendo la información de salud protegida sobre usted para este estudio de investigación. Si desea retirar su permiso, debe comunicárselo por escrito a la persona a cargo de este estudio de investigación según el nombre y la dirección indicados arriba. Una vez retirado el permiso, no podrá seguir participando en el estudio.

Si retira su permiso, en la medida en que hayamos conservado cualquier información de salud protegida que pueda relacionarse con usted, dejaremos de utilizar y compartir la información de salud protegida para la investigación. Sin embargo, no podremos recuperar la información que ya se haya usado o compartido con otras personas o entidades, y tal información se puede seguir utilizando para determinados fines, como por ejemplo cumplir con la ley o mantener la confiabilidad del estudio.

Usted tiene derecho a ver y obtener una copia de la información de salud protegida sobre usted que se usa o se comparte para el tratamiento o para el pago. Para pedir esta información, comuníquese con la persona a cargo de este estudio de investigación identificada arriba. Podrá obtener dicha información únicamente después de que la investigación haya finalizado.

BURG TRANSLATIONS

460 W Irving Park Rd
Suite C
Bensenville, IL 60106
800 959 2874
www.burgtranslations.com

September 27, 2022

I, the undersigned, having been duly sworn, depose and say that the following documents:

- Patient Gateway Research Invitation Letter_Spanish.docx
- Patient Messages_final_Spanish.docx
- Study Fact Sheet_090122_Spanish.docx

have been translated from **English** into **Spanish** by BURG Translations, Inc. and, to the best of my knowledge and belief, they are true and accurate renderings of the original documents.

STATE OF ILLINOIS
COUNTY OF COOK
Chicago, Illinois, September 27, 2022

Signed and attested before me on this 27th day of September, 2022 by **Michelle Delana**

BURG TRANSLATIONS, INC.
460 W Irving Park Rd,
Suite C
Bensenville, Illinois 60106
(800) 959-2874

Michelle Delana
Project Manager

ANGELO PASSALACQUA
Official Seal
Notary Public - State of Illinois
My Commission Expires Nov 29, 2024

Angelo Passalacqua, Notary Public

Cook County, State of Illinois

American Translators Association CORPORATE MEMBER · TÜV MANAGEMENT SERVICE ISO 9001 · Certified to ISO 17100:2015 TÜVRheinland · ALC ASSOCIATION OF LANGUAGE COMPANIES

Mass General Brigham

[Fecha]

[Nombre del paciente
Dirección
Ciudad, Estado, Código postal]

Estimada [Sra. paciente]:

Le estamos escribiendo para informarle sobre un estudio de investigación que podría interesarle. Mass General Brigham se compromete a proporcionar una excelente atención para usted y nuestra comunidad. Una parte importante de nuestra misión es aprender nuevas formas de cuidar a nuestros pacientes realizando investigaciones. Nuestros pacientes tienen un papel importante en la investigación al participar en estudios para ayudar a descubrir mejores tratamientos o mejores formas de prevenir los problemas de salud.

Mass General Brigham es un sistema de salud que incluye hospitales, centros de salud comunitarios y grupos de médicos. Trabajamos juntos para proporcionar la mejor atención posible a nuestra comunidad. Los pacientes de Mass General Brigham tienen acceso a una atención de alta calidad en todo nuestro sistema. Como sistema de atención médica, también trabajamos juntos para proporcionar acceso a los estudios de investigación a todos nuestros pacientes.

La información que se incluye a continuación es sobre el estudio que podría interesarle. Para decidir si es una buena opción para usted, puede obtener más información o hablar con el equipo de investigación.

Nombre del estudio: Cerrando la brecha entre el posparto y la atención primaria

Qué estamos estudiando: Métodos para mejorar la atención primaria entre las mujeres puérperas

Quiénes podrían calificar: Embarazadas y puérperas que podrían beneficiarse especialmente de la atención primaria en el período de posparto.

Tal vez esto se aplica a usted o tal vez no.

Qué se le solicita que haga en el estudio: Aceptar recibir mensajes esporádicos del equipo del estudio a través de Patient Gateway desde el momento de la inscripción hasta 4 meses de posparto

Para obtener más información:

Para obtener más información sobre ESTE estudio: Comuníquese con la coordinadora de investigación del estudio, Fowsia Warsame, al 617-643-5483. Después de

Mass General Brigham

obtener más información, puede decidir participar o no. Es posible que también descubra que no califica para el estudio. Independientemente de si participa o no en el estudio, la atención médica que recibe aquí en Mass General Brigham no cambiará.

Si definitivamente NO tiene interés en ESTE estudio, y no quiere saber nada más al respecto, comuníquese con la coordinadora del estudio, Fowsia Warsame, al 617-643-5483. Si no recibimos su respuesta en dos semanas, es posible que nos dirijamos a usted o le llamemos para ver si desea obtener más información sobre el estudio.

Para obtener más información sobre
la investigación en general: Llame a la Oficina
del Orientador de Investigación: **857-282-5370**

Visite el sitio web de Rally:rally.partners.org/research

Si no desea recibir notificaciones sobre *ninguno* de los estudios de Mass General Brigham:

- Inicie sesión en su cuenta de Patient Gateway.
  Busque "Resources" (Recursos) en el menú y seleccione "Research Opportunities" (Oportunidades de investigaciones)
  Lea la información del resumen y seleccione el botón correcto.

O

- Comuníquese con la Oficina del Orientador de Investigaciones:
  Llame al 857-282-5370

Desde ya le agradecemos por considerar este estudio de investigación.

Atentamente,

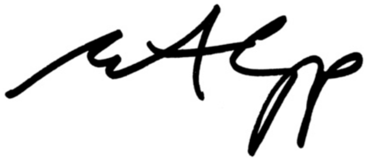

Mark Clapp, MD MPH
Medicina materno-fetal
Massachusetts General Hospital
617-724-4531

Cerrando la brecha entre el posparto y la atención primaria: Una intervención basada en la ciencia del comportamiento para mejorar el manejo de enfermedades crónicas entre las mujeres puérperas

Investigadores principales del estudio: Mark Clapp, MD MPH (MGH); Jessica Cohen, PhD (Harvard)
Investigador principal de la institución: Mark Clapp, MD MPH (MGH)

Mensaje de texto para el paciente

Grupo	Momento	Tipo	Contenido del mensaje si se programó una cita con un PCP	Contenido del mensaje si no se programó una cita con un PCP
Control	Entre 2 y 4 semanas antes de la EDD	PG	N/C	Estimada XXX:<br><br>Disponer de un proveedor de atención primaria (*primary care provider*, PCP) y visitarle al menos una vez al año es importante para su salud y bienestar actuales y futuros.<br><br>Estos proveedores tienen funciones diferentes a las de su obstetra (médico del embarazo) o partera. Se recomienda que vea a su PCP en los meses posteriores al parto, ya que dejará de ver a su proveedor de atención del embarazo.<br><br>Si tiene preguntas sobre cómo encontrar o ver a su PCP después del parto, pregunte a su proveedor de atención del embarazo o llame a su PCP.<br><br>--<br>*El equipo de Bridge Study ha enviado este mensaje. Si desea dejar de recibir mensajes de este equipo, llame al coordinador del estudio al 617-643-5483 o envíe un correo electrónico al equipo del estudio a bridgestudy@mgh.harvard.edu. No responda de manera directa a este mensaje con preguntas sobre su salud o para programar el seguimiento.*

** EDD, fecha estimada de parto; PG, portal de mensajería segura de Patient Gateway.*

Grupo	Moment o	Tipo	Contenido del mensaje si se programó una cita con un PCP	Contenido del mensaje si no se programó una cita con un PCP
Intervención	Entre 2 y 4 seman as antes de la EDD	PG	N/C	Estimada XXX:<br><br>Disponer de un proveedor de atención primaria (*primary care provider*, PCP) y visitarle al menos una vez al año es importante para su salud y bienestar actuales y futuros.<br><br>Estos proveedores tienen funciones diferentes a las de su obstetra (médico del embarazo) o partera. Se recomienda que vea a su PCP en los meses posteriores al parto, ya que dejará de ver a su proveedor de atención del embarazo.<br><br>Para ayudarla en la transición a la atención primaria después del parto, programaremos su cita de "**Transición del embarazo a la atención primaria**" con su PCP en los próximos meses. Le enviaremos un mensaje una vez que se haya programado la cita. *Si tiene preferencias en cuanto a la fecha y el horario para esta cita O no desea que le programemos esta cita, llame al coordinador del estudio (617-643-5483) o envíe un correo electrónico al equipo del estudio a bridgestudy@mgh.harvard.edu en las 2 semanas siguientes.*<br><br>Si tiene preguntas sobre su atención o sobre cómo consultar a su PCP después del parto, pregúntele a su proveedor de atención del embarazo o llame a su PCP.<br><br>--<br>*El equipo de Bridge Study ha enviado este mensaje. Si desea dejar de recibir mensajes de*

Grupo	Moment o	Tipo	Contenido del mensaje si se programó una cita con un PCP	Contenido del mensaje si no se programó una cita con un PCP
				este equipo, llame al coordinador del estudio al 617-643-5483 o envíe un correo electrónico al equipo del estudio a bridgestudy@mgh.harvard.edu. No responda de manera directa a este mensaje con preguntas específicas sobre su salud o plan de seguimiento.
Intervención	Entre 2 y 4 seman as antes de la EDD	SMS	Ha recibido un mensaje importante a través de Patient Gateway. Consulte el portal para obtener más información. -Bridge Study de MGH [no responda]<br>*cantidad de caracteres: 141*	Ha recibido un mensaje importante a través de Patient Gateway. Consulte el portal para obtener más información. -Bridge Study de MGH<br>*cantidad de caracteres: 147*
Intervención	2 seman as después de la EDD	PG	Estimada XXX:<br><br>Se ha reservado especialmente para usted su cita de **“Transición del embarazo a la atención primaria”**:<br>XXX INFORMACIÓN DE LA CITA ##<br><br>Esta cita se programó para ayudarla en la transición de su proveedor de atención del embarazo a su proveedor de atención primaria (PCP) después de su parto. Su PCP se asegurará de que tenga un plan para mantenerse saludable y puede responder a cualquier pregunta o inquietud que tenga sobre su salud.<br><br>Las citas pueden tener un costo dependiendo de su plan de seguro (como por ejemplo, un copago o un deducible). Si tiene preguntas sobre la cobertura de su seguro o los posibles costos asociados con una visita, llame al consultorio de su PCP, al proveedor de seguros o a la Oficina de Facturación de Pacientes de Mass General Brigham al 617-726-3884.<br><br>Si no puede acudir a esta cita o desea cancelarla o reprogramarla, llame al consultorio de su PCP al	Estimada XXX:<br><br>No hemos podido reservar su cita de **"Transición del embarazo a la atención primaria"** con XXX en la consulta de XXX. Llame al consultorio de su PCP para programarla. El número de teléfono del consultorio es: XXX<br><br>Estamos intentando programar esta cita para usted con el fin de ayudarla en la transición de su proveedor de atención del embarazo a su proveedor de atención primaria (PCP) después de su parto. Su PCP se asegurará de que tenga un plan para mantenerse saludable y puede responder a cualquier pregunta o inquietud que tenga sobre su salud.<br><br>Las citas pueden tener un costo dependiendo de su seguro (como por ejemplo, un copago o deducible). Si tiene preguntas sobre la cobertura de su seguro o los posibles costos asociados con una visita, llame al consultorio de su PCP, al proveedor de seguros o a la Oficina de Facturación de Pacientes de Mass General Brigham al 617-726-3884.

Grupo	Moment o	Tipo	Contenido del mensaje si se programó una cita con un PCP	Contenido del mensaje si no se programó una cita con un PCP
			XXX o comuníquese con el equipo de Bridge Study (617-643-5483). Tenga en cuenta que algunas clínicas pueden cobrar una tarifa por no presentarse a una visita programada.<br><br>--<br>*El equipo de Bridge Study ha enviado este mensaje. Si desea dejar de recibir mensajes de este equipo, llame al coordinador del estudio al 617-643-5483 o envíe un correo electrónico al equipo del estudio a bridgestudy@mgh.harvard.edu. No responda de manera directa a este mensaje con preguntas específicas sobre su salud o plan de seguimiento.*	--<br>*El equipo de Bridge Study ha enviado este mensaje. Si desea dejar de recibir mensajes de este equipo, llame al coordinador del estudio al 617-643-5483 o envíe un correo electrónico al equipo del estudio a bridgestudy@mgh.harvard.edu. No responda de manera directa a este mensaje con preguntas específicas sobre su salud o plan de seguimiento.*
Intervención	2 seman as después de la EDD	SMS	¡Se ha reservado para usted una cita con el PCP! Consulte Patient Gateway para obtener más detalles. -Bridge Study de MGH<br>[no responda]<br>*cantidad de caracteres: 115*	¡Oh, no! Estamos teniendo problemas para programar su cita con el PCP. Consulte Patient Gateway para obtener más detalles. -Bridge Study de MGH<br>[no responda]<br>*cantidad de caracteres: 158*
Intervención	4 seman as antes de la cita con el PCP	PG	Estimada XXX:<br><br>Su proveedor de atención primaria (PCP) espera atenderla después de su embarazo.<br>Se ha reservado para usted su cita de "**Transición del embarazo a la atención primaria**":<br>XXX INFORMACIÓN DE LA CITA ##<br><br>Esta cita se programó para ayudarla en la transición de su proveedor de atención en el embarazo a su proveedor de atención primaria (PCP) después de su parto. Su PCP se asegurará de que tenga un plan para mantenerse saludable y puede responder a cualquier pregunta o inquietud que tenga sobre su salud.	Estimada XXX:<br><br>No vemos que haya programado su cita de "**Transición del embarazo a la atención primaria**". Llame al consultorio de su proveedor de atención primaria (PCP) para programarla. El número de teléfono del consultorio es: XXX<br><br>Estamos intentando programar esta cita para usted con el fin de ayudar a la transición de su proveedor de atención del embarazo a su PCP después de su parto. Su PCP se asegurará de que tenga un plan para mantenerse saludable y puede responder a cualquier pregunta o inquietud que tenga sobre su salud.

Grupo	Moment o	Tipo	Contenido del mensaje si se programó una cita con un PCP	Contenido del mensaje si no se programó una cita con un PCP
			Las citas pueden tener un costo dependiendo de su seguro (como por ejemplo, un copago o deducible). Si tiene preguntas sobre la cobertura de su seguro o los posibles costos asociados con una visita, llame al consultorio de su PCP, al proveedor de seguros o a la Oficina de Facturación de Pacientes de Mass General Brigham al 617-726-3884.<br><br>Si no puede acudir a esta cita o desea cancelarla o reprogramarla, llame al consultorio de su PCP al XXX o comuníquese con el equipo de Bridge Study (617-643-5483). Tenga en cuenta que algunas clínicas pueden cobrar una tarifa por no presentarse a una visita programada.<br><br>--<br>*El equipo de Bridge Study ha enviado este mensaje. Si desea dejar de recibir mensajes de este equipo, llame al coordinador del estudio al 617-643-5483 o envíe un correo electrónico al equipo del estudio a bridgestudy@mgh.harvard.edu. No responda de manera directa a este mensaje con preguntas específicas sobre su salud o plan de seguimiento.*	Las citas pueden tener un costo dependiendo de su seguro (como por ejemplo, un copago o deducible). Si tiene preguntas sobre la cobertura de su seguro o los posibles costos asociados con una visita, llame al consultorio de su PCP, al proveedor de seguros o a la Oficina de Facturación de Pacientes de Mass General Brigham al 617-726-3884.<br><br>--<br>*El equipo de Bridge Study ha enviado este mensaje. Si desea dejar de recibir mensajes de este equipo, llame al coordinador del estudio al 617-643-5483 o envíe un correo electrónico al equipo del estudio a bridgestudy@mgh.harvard.edu. No responda de manera directa a este mensaje con preguntas específicas sobre su salud o plan de seguimiento.*
Intervención	4 semanas antes de la cita con el PCP O BIEN 8 semanas después del parto si no	SMS	¡Su PCP está esperando su próxima cita! Consulte Patient Gateway para obtener más detalles. -Bridge Study de MGH<br>[no responda]<br>*cantidad de caracteres: 128*	¡Oh, no! No tiene programada una cita de transición de atención con el PCP. Consulte Patient Gateway para obtener más detalles. -Bridge Study de MGH<br>[no responda]<br>*cantidad de caracteres: 139*

Grupo	Momento	Tipo	Contenido del mensaje si se programó una cita con un PCP	Contenido del mensaje si no se programó una cita con un PCP
	programó una cita con el PCP			
Intervención	1 semana antes de la cita con el PCP O BIEN 12 semanas después del parto si no programó una cita con el PCP	PG	Estimada XXX:<br><br>¡Se aproxima su cita reservada de "**Transición del embarazo a la atención primaria**"!<br>XXX INFORMACIÓN DE LA CITA ##<br><br>Esta cita se programó para ayudarla en la transición de su proveedor de atención en el embarazo a su proveedor de atención primaria (PCP) después de su parto. Su PCP se asegurará de que tenga un plan para mantenerse saludable y puede responder a cualquier pregunta o inquietud que tenga sobre su salud.<br><br>Las citas pueden tener un costo dependiendo de su seguro (como por ejemplo, un copago o deducible). Si tiene preguntas sobre la cobertura de su seguro o los posibles costos asociados con una visita, llame al consultorio de su PCP, al proveedor de seguros o a la Oficina de Facturación de Pacientes de Mass General Brigham al 617-726-3884.<br><br>Si no puede acudir a esta cita o desea cancelarla o reprogramarla, llame al consultorio de su PCP al XXX o comuníquese con el equipo de Bridge Study (617-643-5483). Tenga en cuenta que algunas clínicas pueden cobrar una tarifa por no presentarse a una visita programada.<br><br>--<br>*El equipo de Bridge Study ha enviado este mensaje. Si desea dejar de recibir mensajes de este equipo, llame al coordinador del estudio al*	Estimada XXX:<br><br>No vemos que haya programado su cita de "**Transición del embarazo a la atención primaria**". Llame al consultorio de su proveedor de atención primaria (PCP) para programarla. El número de teléfono del consultorio es: XXX<br><br>Estamos intentando programar esta cita para usted con el fin de ayudar a la transición de su proveedor de atención en el embarazo a su PCP después de su parto. Su PCP se asegurará de que tenga un plan para mantenerse saludable y puede responder a cualquier pregunta o inquietud que tenga sobre su salud.<br><br>Las citas pueden tener un costo dependiendo de su seguro (como por ejemplo, un copago o deducible). Si tiene preguntas sobre la cobertura de su seguro o los posibles costos asociados con una visita, llame al consultorio de su PCP, al proveedor de seguros o a la Oficina de Facturación de Pacientes de Mass General Brigham al 617-726-3884.<br><br>--<br>*El equipo de Bridge Study ha enviado este mensaje. Si desea dejar de recibir mensajes de este equipo, llame al coordinador del estudio al 617-643-5483 o envíe un correo electrónico al equipo del estudio a bridgestudy@mgh.harvard.edu. No responda de manera directa a este mensaje con preguntas específicas sobre su salud o plan de seguimiento.*

Grupo	Moment o	Tipo	Contenido del mensaje si se programó una cita con un PCP	Contenido del mensaje si no se programó una cita con un PCP
			617-643-5483 o envíe un correo electrónico al equipo del estudio a bridgestudy@mgh.harvard.edu. No responda de manera directa a este mensaje con preguntas específicas sobre su salud o plan de seguimiento.	
		SMS	Recordatorio: ¡Su cita con el PCP es dentro de los próximos días! Consulte Patient Gateway para obtener más detalles. -Bridge Study de MGH<br>[no responda]<br>*cantidad de caracteres: 131*	¡Recordatorio! No tiene programada una cita de transición de atención con el PCP. Consulte Patient Gateway para obtener más detalles. -Bridge Study de MGH<br>[no responda]<br>*cantidad de caracteres: 142*

Title: Bridging the Gap from Postpartum to Primary Care: A Behavioral Science Informed Intervention to Improve Chronic Disease Management among Postpartum Women (the Bridge Study)

Sponsor Name:

PI Name: Clapp, Mark A **Protocol #:** 2022P001723 **Type:** Amendment (AME6)

Date Received: October 26, 2022

Signatures

PI Name: Clapp, Mark A, MD, MPH

Authenticated: October 26, 2022

Amendment

COVID-19 Amendment

Is this amendment ONLY related to research impacted by COVID-19?

Refer to MGB Policy on Conduct of Human Research Activities during COVID-19 Operations for description of Amendments which do **require prior IRB review and approval.**

- ◯ Yes
- ◉ No

Central IRB Performance Sites

Is this a protocol where the Mass General Brigham IRB is serving as the single IRB (sIRB) for external sites/institutions?

- ◉ Yes
- ◯ No

Would you like to 'Add' a Site?

- ◯ Yes
- ◉ No

Would you like to 'Remove' a Site?

- ◯ Yes
- ◉ No

Sponsor Amendment

Is there a sponsor amendment number?

- ◯ Yes
- ◉ No

Change in Protocol Status

Is this a cede protocol or project that was determined to be exempt, not human subjects research or not engaged in human subjects research?

- ◯ Yes
- ◉ No

Mass General Brigham

Do you need to change the overall status of the protocol? For example, Re-Open to Enrollment or indicate that Research Interventions/Assessments Continue after telling the IRB these have ceased.

- ◯ Yes
- ◉ No

Briefly describe the proposed changes:

1) Adding Spanish translation of baseline survey

2) Updating patient messages

Provide rationale for the proposed changes:

1) Spanish version of the survey

2) Old version of patient message file was uploaded in the original submission; updated/correct patient message attachment added. The content, frequency, and purpose of the messages have not changed from the originally approved versions.

*no patients have been enrolled yet

Will the proposed change(s) significantly alter the risk to benefit assessment the IRB relied upon to approve the protocol?

- ◯ Yes
- ◉ No

Will the proposed change(s) significantly affect the integrity of the protocol?

- ◯ Yes
- ◉ No

Informed Consent

Do the changes require a revision to the consent form?

- ◯ Yes
- ◉ No

Attachments

Name	Mode
Baseline_10-5-22_final_Spanish (Instrument/Questionnaire)	Electronic
Letter of Certification_P-2022-4226[81] (Other)	Electronic
Patient Messages_final (Document for review)	Electronic

Cerrando la brecha entre el posparto y la atención primaria

Investigadores principales del estudio: Mark Clapp, MD MPH (MGH); Jessica Cohen, PhD (Harvard)

Investigador principal de la institución: Mark Clapp, MD MPH (MGH)

SECCIÓN 5. Encuesta para pacientes

Se mantendrá la privacidad de toda la información y los servicios que está recibiendo actualmente no se verán afectados.

701. ¿Cuál es su estado civil?<br>1. Casada<br>2. Divorciada<br>3. Viuda<br>4. Separada<br>5. Nunca se casó<br>6. Integra una pareja no casada<br>88. No sabe/no está segura<br>99. Prefiere no decirlo
702. ¿Qué opción describe mejor el grado o nivel más alto de escolaridad que ha completado?<br>1. 8.º grado o menos<br>2. 9.º - 12.º grado, sin diploma<br>3. Escuela secundaria completa o GED aprobado<br>4. Algunos créditos universitarios, pero sin título<br>5. Técnico superior (p. ej. AA, AS)<br>6. Licenciatura (p. ej. BA, AB, BS)<br>7. Maestría (p. ej. MA, MS, MEng, MEd, MSW, MBA)<br>8. Doctorado (p. ej. Ph.D., EdD) o título profesional<br>88. No sabe/no está segura<br>99. Prefiere no decirlo

703. ¿Aproximadamente cuánto dinero ha ganado usted personalmente en los últimos 12 meses?<br>1. $0<br>2. $1 – $6,000<br>3. $6,001 – $10,000<br>3. $10,001 – $30,000<br>4. $30,001 – $50,000<br>5. $50,001 – $75,000<br>6. $75,001 – $100,000<br>7. Más de $100,001<br>88. No sabe/no está segura<br>99. Prefiere no decirlo
704. ¿Prevé perder el seguro de salud o estar sin seguro durante el año posterior al nacimiento de su bebé?<br>1. Sí<br>2. No<br>88. No sabe/no está segura<br>99. Prefiere no decirlo
705. ¿Prevé que cambie su proveedor de seguros principal después de su embarazo?<br>1. Sí<br>2. No<br>88. No sabe/no está segura<br>99. Prefiere no decirlo
501. ¿Qué tan fácil o difícil le resulta acceder al portal para pacientes Patient Gateway?<br>1. Muy fácil<br>2. Algo fácil<br>3. Ni fácil ni difícil<br>4. Algo difícil<br>5. Muy difícil<br>88. No sabe/no está segura

99. Prefiere no responder
502. Durante el año **ANTERIOR** a su embarazo, ¿cómo describiría que fue su salud FÍSICA?<br>1. Excelente<br>2. Muy buena<br>3. Buena<br>4. Aceptable<br>5. Mala<br>88. No sabe/no está segura<br>99. Prefiere no responder
503. Durante el año **ANTERIOR** a su embarazo, ¿cómo describiría que fue su salud MENTAL?<br>1. Excelente<br>2. Muy buena<br>3. Buena<br>4. Aceptable<br>5. Mala<br>88. No sabe/no está segura<br>99. Prefiere no responder
504. Piense en el año anterior a su embarazo; <u>si necesitó consultar a un profesional de la salud por un problema que no era de emergencia</u>, ¿qué tan fácil o difícil le resultó?<br>1. Muy fácil<br>2. Algo fácil<br>3. Ni fácil ni difícil<br>4. Algo difícil<br>5. Muy difícil88. No sabe/no está segura<br>99. Prefiere no responder
505. Hay muchos motivos por los que la gente retrasa o evita buscar atención médica. ¿Alguno de los siguientes problemas hizo que no obtuviera la atención médica que necesitaba antes de su embarazo? *Lea cada opción en voz alta. Cuando termine, pregunte: "¿Algo más?".*

1. No estaba segura de con quién debía coordinar una cita<br>2. Le resultó difícil conseguir una cita<br>3. Tuvo dificultades de transporte<br>4. La clínica o el consultorio del médico no estaba abierto cuando usted pudo llegar<br>6. Le preocupaba el costo financiero de la atención<br>7. Dificultad para conseguir tiempo libre en el trabajo para obtener atención<br>8. No estaba segura de que la atención médica le ayudaría<br>9. Dificultad para conseguir cuidado para los niños<br>10. Otro, especifique ______<br>88. No sabe/no está segura<br>99. Prefiere no responder
505. ¿Tiene un auto para su uso personal?<br>1. Sí<br>2. No<br>88. No sabe/no está segura<br>99. Prefiere no responder
506. ¿Cuál fue su medio de transporte a la cita de hoy? *Seleccione todo lo que corresponda.*<br>1. Auto personal<br>2. Taxi, Uber o Lyft<br>3. Auto de un amigo o un familiar<br>4. Autobús<br>5. Metro/tren ("T")<br>6. Bicicleta<br>7. A pie/caminando<br>8. Otro, especifique ______<br>88. No sabe/no está segura<br>99. Prefiere no responder
505. ¿Tiene un auto para su uso personal?<br>1. Sí

2. No<br>88. No sabe/no está segura<br>99. Prefiere no responder
507. En el año anterior a su embarazo, aparte de las visitas a la sala de emergencias, ¿consultó a un médico o a un enfermero por algún motivo? Esto no incluye visitas a la sala de emergencias pero puede incluir consultas con su médico de cabecera, un médico especialista, atención urgente, etc.<br>1. Sí, especifique dónde________<br>2. No<br>88. No sabe/no está segura<br>99. Prefiere no responder

508-511: ¿Alguna vez un médico, un enfermero u otro personal de la salud le dijo que tiene algo de lo siguiente?

Afección	Sí	No	No sabe/no está segura	Prefiere no responder
Presión arterial alta o hipertensión	○	○	○	○
Diabetes o nivel alto de azúcar en sangre	○	○	○	○
Ansiedad o depresión	○	○	○	○
Sobrepeso u obesidad	○	○	○	○

SECCIÓN 6. **Trabajo y licencia posparto**

601. ¿Tiene actualmente un trabajo remunerado?<br>1. Sí → **Pase a la 603**<br>2. No

88. No sabe/no está segura<br><br>99. Prefiere no decirlo
602. ¿Ha tenido un trabajo remunerado en algún momento de los últimos 12 meses?<br><br>1. Sí<br><br>2. No → **Pase a la 604**<br><br>88. No sabe/no está segura<br><br>99. Prefiere no decirlo
603. ¿Qué describe mejor el tipo o tipos de trabajo que tuvo en los últimos 12 meses? (*Marque todo lo que corresponda. Mencione el nombre del empleador. Por cada empleador mencionado, pregunte: "¿Este empleador está en Massachusetts?"*).<br><br>1. Gobierno federal o estatal:_____ Nombre:_________________________<br>¿En Massachusetts? Sí: _____ No: _______<br>2. Empresa privada o con fines de lucro: _____ Nombre:_________________________<br>¿En Massachusetts? Sí: _____ No: _______<br>3. Municipalidad (depto. escolar, obras públicas, autoridad de vivienda, distrito escolar regional):_____ Nombre:_________________________<br>¿En Massachusetts? Sí: _____ No: _______<br>4. Iglesia u organización religiosa______ Nombre: ____________________<br>¿En Massachusetts? Sí: _____ No: _______<br>5. Trabajador independiente_____________ Nombre: _____________________<br>¿En Massachusetts? Sí: _____ No: _______<br>6. Contratista independiente _____________ Nombre: _____________________<br>¿En Massachusetts? Sí: _____ No: _______<br>7. Otro: ______________<br>¿En Massachusetts? Sí: _____ No: _______<br><br>88. No sabe/no está segura<br><br>99. Prefiere no decirlo
604. ¿Tiene planeado reintegrarse a trabajar **o** empezar un trabajo nuevo dentro del primer año después del nacimiento de su bebé?<br><br>1. Sí, me reintegraré a mi trabajo anterior<br><br>2. Sí, empezaré un trabajo nuevo<br><br>3. No **-> Pase a la 606**<br><br>88. No sabe/no está segura<br><br>99. Prefiere no decirlo

605. ¿Cuánto tiempo después del nacimiento del bebé pretende reintegrarse a trabajar o empezar a trabajar?<br><br>1. Menos de una semana<br><br>2. 1 a 4 semanas<br><br>3. 5 a 8 semanas<br><br>4. 9 a 12 semanas<br><br>5. 13 a 16 semanas<br><br>6. 17 a 20 semanas<br><br>7. 21 a 24 semanas<br><br>8. 25 semanas o más<br><br>88. No sabe/no está segura<br><br>99. Prefiere no decirlo
606. ¿Alguna vez vio, leyó o escuchó algo sobre un programa estatal de Massachussetts llamado "Licencia paga por razones médicas y familiares" (*Paid Family and Medical Leave*, PFML)? Este programa ofrece a los trabajadores elegibles tiempo libre pago por razones familiares o médicas.<br><br>1. Sí, he visto, leído o escuchado acerca del programa de Licencia paga por razones médicas y familiares de Massachusetts.<br><br>2. No, no he visto, leído ni escuchado acerca del programa de Licencia paga por razones médicas y familiares de Massachusetts.<br><br>88. No conozco/no estoy segura acerca el programa de Licencia paga por razones médicas y familiares de Massachusetts.<br><br>99. Prefiere no decirlo
607. ¿Tiene intención de aplicar para el programa de Licencia paga por razones médicas y familiares de Massachusetts?<br><br>1. Sí<br><br>2. No. Diga el motivo: ____________________________<br><br>88. No sabe/no está segura<br><br>99. Prefiere no decirlo

BURG TRANSLATIONS
460 W Irving Park Rd
Suite C
Bensenville, IL 60106
800 959 2874
www.burgtranslations.com

October 24, 2022

I, the undersigned, having been duly sworn, depose and say that the following document:

- Baseline_10-5-22_final_Spanish.docx

has been translated from **English** into **Spanish** by BURG Translations, Inc. and, to the best of my knowledge and belief, it is a true and accurate rendering of the original document.

STATE OF ILLINOIS
COUNTY OF COOK
Chicago, Illinois, October 24, 2022

Signed and attested before me on this 24th day of October, 2022 by **Michelle Delana**

BURG TRANSLATIONS, INC.
460 W Irving Park Rd,
Suite C
Bensenville, Illinois 60106
(800) 959-2874

Michelle Delana
Sr. Project Manager

ANGELO PASSALACQUA
Official Seal
Notary Public - State of Illinois
My Commission Expires Nov 29, 2024

Angelo Passalacqua, Notary Public

Cook County, State of Illinois

Certified to ISO 9001:2015 TÜVRheinland

Certified to ISO 17100:2015 TÜVRheinland

ata American Translators Association CORPORATE MEMBER

ALC ASSOCIATION OF LANGUAGE COMPANIES

Bridging the Gap from Postpartum to Primary Care: A Behavioral Science Informed Intervention to Improve Chronic Disease Management among Postpartum Women

Study PIs: Mark Clapp, MD MPH (MGH); Jessica Cohen, PhD (Harvard)
Site PI: Mark Clapp, MD MPH (MGH)

Patient Message Text

Group	Timing	Type	Message content if PCP appt scheduled	Message content if PCP appt not scheduled
Control	2-4 weeks prior to EDD	PG	N/A	Dear XXX,<br><br>Having a primary care provider (PCP) and seeing them at least once a year is important for your current and future health and wellbeing.<br><br>These providers have different roles than your obstetrician (pregnancy doctor) or midwife. It is recommended that you see your PCP in the months after your delivery since you will stop seeing your pregnancy provider.<br><br>If you have questions about finding or seeing your PCP after your delivery, please ask your pregnancy provider or call your PCP.<br><br>--<br>*This message has been sent from The Bridge Study team. If you wish to no longer receive any messages from this team, please call the study coordinator 617-643-5483 or email the study team at bridgestudy@mgh.harvard.edu. Do not reply directly to this message with questions about your health or to arrange follow-up.*

** EDD, estimated date of delivery; PG, Patient Gateway secure messaging portal.*

Group	Timing	Type	Message content if PCP appt scheduled	Message content if PCP appt not scheduled
Intervention	2-4 weeks prior to EDD	PG	N/A	Dear XXX,<br><br>Having a primary care provider (PCP) and seeing them at least once a year is important for your current and future health and wellbeing.<br><br>These providers have different roles than your obstetrician (pregnancy doctor) or midwife. It is recommended that you see your PCP in the months after your delivery since you will stop seeing your pregnancy provider.<br><br>To help support you in transitioning to primary care after your delivery, we will schedule your "**Pregnancy-to-Primary Care Transition**" appointment with your PCP within the next few months. We will message you once the appointment has been scheduled. *If you have date/time preferences for this appointment OR do not want us to schedule this appointment for you, please call the study coordinator (617-643-5483) or email the study team at bridgestudy@mgh.harvard.edu within 2 weeks.*<br><br>If you have questions about your care or seeing your PCP after your delivery, please ask your pregnancy provider or call your PCP.<br><br>--<br>*This message has been sent from The Bridge Study team. If you wish to no longer receive any messages from this team, please call the study coordinator 617-643-5483 or email the study team at bridgestudy@mgh.harvard.edu. Do not reply directly to this message with specific questions about your health or follow-up plan.*

Group	Timing	Type	Message content if PCP appt scheduled	Message content if PCP appt not scheduled
Intervention	2-4 weeks prior to EDD	SMS	You have received an important message through Patient Gateway. Please check the portal for more information. -The MGH Bridge Study<br>[do not reply]<br>*character count: 141*	You have received an important message through Patient Gateway. Please check the portal for more information. -The MGH Bridge Study<br>*character count: 147*
Intervention	2 weeks after EDD	PG	Dear XXX,<br><br>Your “**Pregnancy-to-Primary Care Transition**” Appointment has been specially reserved for you: XXX APPT INFO ##<br><br>This appointment was made to help support your transition from your pregnancy provider to your primary care provider (PCP) after your delivery. Your PCP will make sure you have a plan in place to keep you healthy and can answer any questions or concerns you have about your health.<br><br>Appointments may have a cost depending on your insurance plan (such as a co-pay or deductible). If you have questions about your insurance coverage or possible costs associated with a visit, please call your PCP’s office, insurance provider, or Mass General Brigham Patient Billing office at 617-726-3884.<br><br>If you are unable to make this appointment or wish to cancel or reschedule, please call your PCP’s office at XXX or contact the Bridge Study Team (617-643-5483). Please note that some clinics may charge a fee for not showing to a scheduled visit.<br><br>--<br>*This message has been sent from The Bridge Study team. If you wish to no longer receive any messages from this team, please call the study coordinator 617-643-5483 or email the study team*	Dear XXX,<br><br>We have been unable to reserve your “**Pregnancy-to-Primary Care Transition**” Appointment for you with XXX at XXX practice. Please call your PCP’s office to schedule. The office phone number is: XXX<br><br>We are trying to make this appointment for you to help support your transition from your pregnancy provider to your primary care provider (PCP) after your delivery. Your PCP will make sure you have a plan in place to keep you healthy and can answer any questions or concerns you have about your health.<br><br>Appointments may have a cost depending on your insurance (such as a copay or deductible). If you have questions about your insurance coverage or possible costs associated with a visit, please call your PCP’s office, insurance provider, or Mass General Brigham Patient Billing office at 617-726-3884.<br><br>--<br>*This message has been sent from The Bridge Study team. If you wish to no longer receive any messages from this team, please call the study coordinator 617-643-5483 or email the study team at bridgestudy@mgh.harvard.edu. Do not reply directly to this message with specific questions about your health or follow-up plan.*

Group	Timing	Type	Message content if PCP appt scheduled	Message content if PCP appt not scheduled
			at bridgestudy@mgh.harvard.edu. Do not reply directly to this message with specific questions about your health or follow-up plan.	
Intervention	2 weeks after EDD	SMS	A PCP appointment has been reserved for you! Check Patient Gateway for details. -The MGH Bridge Study<br>[do not reply]<br>*character count: 115*	Oh no! We are having trouble arranging your PCP appointment. Check Patient Gateway for details. -The MGH Bridge Study<br>[do not reply]<br>*character count: 158*
Intervention	4 weeks before PCP appointment	PG	Dear XXX,<br><br>Your primary care provider (PCP) is looking forward to caring for you after your pregnancy. Your "**Pregnancy-to-Primary Care Transition**" appointment has been reserved for you:<br>XXX APPT INFO ##<br><br>This appointment was made to help support your transition from your pregnancy provider to your primary care provider (PCP) after your delivery. Your PCP will make sure you have a plan in place to keep you healthy and can answer any questions or concerns you have about your health.<br><br>Appointments may have a cost depending on your insurance (such as a copay or deductible). If you have questions about your insurance coverage or possible costs associated with a visit, please call your PCP's office, insurance provider, or Mass General Brigham Patient Billing office at 617-726-3884.<br><br>If you are unable to make this appointment or wish to cancel or reschedule, please call your PCP's office at XXX or contact the Bridge Study Team (617-643-5483). Please note that some clinics may charge a fee for not showing to a scheduled visit.	Dear XXX,<br><br>We do not see that you have your "**Pregnancy-to-Primary Care Transition**" appointment scheduled. Please call your primary care provider (PCP) office to schedule. The office phone number is: XXX<br><br>We are attempting to make this appointment for you in order to help support your transition from your pregnancy provider to your PCP after your delivery. Your PCP will make sure you have a plan in place to keep you healthy and can answer any questions or concerns you have about your health.<br><br>Appointments may have a cost depending on your insurance (such as a copay or deductible). If you have questions about your insurance coverage or possible costs associated with a visit, please call your PCP's office, insurance provider, or Mass General Brigham Patient Billing office at 617-726-3884.<br><br>--<br>*This message has been sent from The Bridge Study team. If you wish to no longer receive any messages from this team, please call the study coordinator 617-643-5483 or email the study team at bridgestudy@mgh.harvard.edu. Do not reply directly to this message with specific questions about your health or follow-up plan.*

Group	Timing	Type	Message content if PCP appt scheduled	Message content if PCP appt not scheduled
			--<br>*This message has been sent from The Bridge Study team. If you wish to no longer receive any messages from this team, please call the study coordinator 617-643-5483 or email the study team at bridgestudy@mgh.harvard.edu. Do not reply directly to this message with specific questions about your health or follow-up plan.*	
Intervention	4 weeks before PCP appt<br>OR<br>8 weeks pp if no PCP appt	SMS	Your PCP is looking forward to your upcoming appointment! Check Patient Gateway for details. -The MGH Bridge Study<br>[do not reply]<br>*character count: 128*	Oh no! You do not have a PCP care transition appointment scheduled. Check Patient Gateway for details. -The MGH Bridge Study<br>[do not reply]<br>*character count: 139*
Intervention	1 week before PCP appt<br>OR<br>12 weeks pp if no PCP appt	PG	Dear XXX,<br><br>Your reserved "**Pregnancy-to-Primary Care Transition**" Appointment is coming up!<br>XXX APPT INFO ##<br><br>This appointment was made to help support your transition from your pregnancy provider to your primary care provider (PCP) after your delivery. Your PCP will make sure you have a plan in place to keep you healthy and can answer any questions or concerns you have about your health.<br><br>Appointments may have a cost depending on your insurance (such as a copay or deductible). If you have questions about your insurance coverage or possible costs associated with a visit, please call your PCP's office, insurance provider, or Mass General Brigham Patient Billing office at 617-726-3884.	Dear XXX,<br><br>We do not see that you have your "**Pregnancy-to-Primary Care Transition**" appointment scheduled. Please call your primary care provider (PCP) office to schedule. The office phone number is: XXX<br><br>We are attempting to make this appointment for you in order to help support your transition from your pregnancy provider to your PCP after your delivery. Your PCP will make sure you have a plan in place to keep you healthy and can answer any questions or concerns you have about your health.<br><br>Appointments may have a cost depending on your insurance (such as a copay or deductible). If you have questions about your insurance coverage or possible costs associated with a visit, please call your PCP's office, insurance provider, or Mass General Brigham Patient Billing office at 617-726-3884.

Group	Timing	Type	Message content if PCP appt scheduled	Message content if PCP appt not scheduled
			If you are unable to make this appointment or wish to cancel or reschedule, please call your PCP's office at XXX or contact the Bridge Study Team (617-643-5483). Please note that some clinics may charge a fee for not showing to a scheduled visit.<br><br>--<br>*This message has been sent from The Bridge Study team. If you wish to no longer receive any messages from this team, please call the study coordinator 617-643-5483 or email the study team at bridgestudy@mgh.harvard.edu. Do not reply directly to this message with specific questions about your health or follow-up plan.*	--<br>*This message has been sent from The Bridge Study team. If you wish to no longer receive any messages from this team, please call the study coordinator 617-643-5483 or email the study team at bridgestudy@mgh.harvard.edu. Do not reply directly to this message with specific questions about your health or follow-up plan.*
		SMS	Reminder: Your PCP appointment is within the next few days! Check Patient Gateway for details. -The MGH Bridge Study<br>[do not reply]<br>*character count: 131*	Reminder! You do not have a PCP care transition appointment scheduled. Check Patient Gateway for details. -The MGH Bridge Study<br>[do not reply]<br>*character count: 142*

Mass General Brigham

Title: Bridging the Gap from Postpartum to Primary Care: A Behavioral Science Informed Intervention to Improve Chronic Disease Management among Postpartum Women (the Bridge Study)

Sponsor Name:

PI Name: Clapp, Mark A **Protocol #:** 2022P001723 **Type:** Amendment (AME8)

Date Received: December 01, 2022

Signatures

PI Name: Clapp, Mark A, MD, MPH

Authenticated: December 01, 2022

Amendment

COVID-19 Amendment

Is this amendment ONLY related to research impacted by COVID-19?

Refer to MGB Policy on Conduct of Human Research Activities during COVID-19 Operations for description of Amendments which do **require prior IRB review and approval.**

- ◯ Yes
- ◉ No

Central IRB Performance Sites

Is this a protocol where the Mass General Brigham IRB is serving as the single IRB (sIRB) for external sites/institutions?

- ◉ Yes
- ◯ No

Would you like to 'Add' a Site?

- ◯ Yes
- ◉ No

Would you like to 'Remove' a Site?

- ◯ Yes
- ◉ No

Sponsor Amendment

Is there a sponsor amendment number?

- ◯ Yes
- ◉ No

Change in Protocol Status

Is this a cede protocol or project that was determined to be exempt, not human subjects research or not engaged in human subjects research?

- ◯ Yes
- ◉ No

Do you need to change the overall status of the protocol? For example, Re-Open to Enrollment or indicate that Research Interventions/Assessments Continue after telling the IRB these have ceased.

○ Yes
◉ No

Briefly describe the proposed changes:

Amending the study protocol to add clarity to one component of the eligibility criteria

---

Provide rationale for the proposed changes:

Study approach and intervention timings were designed (and are currently operating) based on the established date of delivery.

However, the current detailed protocol eligibility criteria has the condition "Currently pregnant or within 2 weeks of delivery," which does not exactly match the language in the rest of the protocol. To add clarity and to match the language used throughout the rest of the protocol and the schema, this has been updated to "Currently pregnant or within 2 weeks of estimated date of delivery."

This change does not affect any patients that have been previously enrolled as the study has been operating based on the clarified language since its start.

No other forms or study documents contain language that need to be updated as a result of this change, including the study fact sheet.

Will the proposed change(s) significantly alter the risk to benefit assessment the IRB relied upon to approve the protocol?

○ Yes
◉ No

Will the proposed change(s) significantly affect the integrity of the protocol?

○ Yes
◉ No

Informed Consent

Do the changes require a revision to the consent form?

○ Yes
◉ No

Attachments

Name	Mode
Detailed Protocol_120122_v1-4_clean (Detailed Protocol)	Electronic

Mass General Brigham

Institutional Review Board
Intervention/Interaction Detailed Protocol

Principal Investigator:	Mark Clapp, MD MPH (MGH), Jessica Cohen, PhD (HSPH)
Project Title:	Bridging the Gap from Postpartum to Primary Care: A Behavioral Science Informed Intervention to Improve Chronic Disease Management among Postpartum Women
Version Date:	12/01/2022
Version Name/Number:	v1.4

1. Background and Significance

Burden of Chronic Disease and the Role of Primary Care
Chronic health conditions affect millions of people in the US each year. In 2018, 51.8% of adults had at least 1 chronic condition, and 27.2% had multiple conditions.[1] The prevalence of chronic disease was higher in women compared to men, older adults (87.6% in adults ≥65 years old), and people with public insurance.[1] Many chronic conditions, by their nature, develop over time and have risk factors that can be identified prior to the onset of disease. Strong evidence underpins prevention strategies for many conditions, which are advanced by the US Preventative Services Taskforce.[2] While the long-term health of a patient is the responsibility of an entire health system, primary care providers (PCPs) provide an integral role in preventing, screening for, and managing disease across the lifespan. Studies have shown the health benefits of receiving regular care under a PCP.[3–6]

Despite the known benefits of having an identifiable usual source of care and the value of health care maintenance, the percent of the population with a PCP has been decreasing over time.[7] Consistently, adults who are younger (age 20-40 years) have the lowest rates of primary care use. In 2015, 44% and 36% of 20- and 30-year-olds had no identifiable source of primary care.[7] The proportion without primary care were also higher among racial/ethnic minority populations and among those who had less education, lower incomes, and no known comorbidities.[7] The number of adults and the time elapsed without regular primary care follow-up can be considered missed opportunities to improve a patient’s current and long-term health. The disproportionate lack of primary care among certain subgroups of the population, often groups who already have worse health outcomes, only serves to widen the pre-existing disparities.

Pregnancy as a Window to Future Health
In the US, 98.2% of pregnant women receive some form of prenatal care, with the average patient having >10 visits during their pregnancy.[8] During a pregnancy, women are screened for pre-existing and pregnancy-related conditions.[9] In adults ages 18-39, the prevalence of obesity, hypertension, prediabetes/diabetes, and mental illness are estimated at 39%, 7.5%, 28%, 25%, respectively.[10–13] Even for those who have no prior identified comorbidities, the most common

pregnancy-related conditions—pregnancy-related hypertension and gestational diabetes (8% and 10% of pregnancies, respectively)—indicate a predisposition to or confer health risks that persist as women age. For example, over 25% of women with gestational diabetes will develop Type 2 diabetes mellitus, and women with pre-eclampsia have more than a two-fold risk of significant cardiovascular disease later in life.[14,15] For these reasons, pregnancy is often considered a "window" into a woman's future health and presents a unique opportunity to optimize a woman's health status early in her life when she otherwise may not have been engaged in care.[16]

Pregnancy as on Opportunity for Engagement with a Long-term Care Provider
Pregnancy is a period when women are highly engaged and active participants in their health care.[17] It has been described as a "golden opportunity" to motivate women towards positive health behaviors, including prevention and management of chronic disease. However, women often fall off a "postpartum cliff" of health system engagement after the early postpartum period.[18] A range of systemic, financial, and behavioral barriers often prevents patients from effectively transitioning to primary care. Postpartum women are often simply told to follow-up with their PCP without much information regarding the importance of this follow up care, without assistance in scheduling an appointment (or identifying a PCP if they don't have one), and often without a direct transfer of relevant health information or accountability across providers.

Postpartum women are left largely on their own to navigate this transition to primary care and, in particular, to navigate it at a time when they face the high cognitive and physical demands of caring for an infant. At this time of limited cognitive bandwidth, the importance of continuity of care for chronic conditions and active engagement in one's longer-term health and wellbeing is unlikely to be salient and top-of-mind. These critical moments of unsupported health care transition can exacerbate pre-existing disparities in health and health care, with patients who are the least able to navigate the US health care system most likely to fall through the cracks. Momentum is building in US health and social safety net policy to facilitate healthy transitions from pregnancy to parenthood. For example, federal and state initiatives to expand pregnancy-related Medicaid coverage from 60 days to one year postpartum have been proposed, and access to paid family leave is increasing. However, very little evidence exists on effective and cost-effective approaches to facilitating transitions to primary care and management of chronic diseases in the postpartum year.

2. Specific Aims and Objectives

The objective of the proposed study is to increase patient engagement in primary care after the immediate postpartum period for women with pregnancy-associated conditions that convey a long-term health risk. Specifically, we aim to evaluate the efficacy of an intervention bundle (automatic scheduling of PCP appointment after delivery, salient labeling, and appointment reminder nudges) to increase patient attendance at a primary care provider appointment (within 4 months of delivery for women with or at risk for obesity, diabetes, hypertension, and/or a mental health condition.

Specific Aims:

1) Test the efficacy of an intervention bundle (patient-tailored health information, automatic scheduling of PCP appointment after delivery, and appointment reminder nudges) to increase patient attendance at a primary care provider appointment within 4 months of

delivery for women with obesity, diabetes, hypertension, and/or a mental health condition
2) Test the efficacy of the intervention bundle to improve compliance with the condition-specific, guideline-based health screenings
3) Test the efficacy of the intervention bundle to reduce unscheduled or urgent encounters (e.g., emergency department visits) within the health system

3. General Description of Study Design

We will conduct a randomized controlled trial comparing this intervention bundle to the receipt of generic information on the importance of primary care follow-up after delivery.

Women will be randomized with equal probability into either a treatment or control arm. The intervention combines several features designed to target reasons for low take-up of primary care among postpartum women (see Logic Model). We leverage the potential value of defaults/opt-out, salient labels, and reminders to encourage use of primary care within 4 months of delivery. Women in both the intervention and control arms will receive information via MGH's patient portal toward the end of the pregnancy regarding the importance of transitions to primary care in the postpartum year. This information will be similar to, but reinforcing, the information they would receive from their obstetrician about following up with their primary care physician. In addition to this initial message, women in the treatment arm will receive the following intervention components, developed based on recent evidence regarding behavioral science approaches to activating health behaviors:[19–21]

- Targeted messages about the importance and benefits of primary care.
- Default scheduling into a primary care appointment at approximately 3-4 months after delivery. The patient will be scheduled for a primary care visit with their assigned primary care provider in the Mass General Brigham system. They will be informed of the option to cancel the appointment, change the appointment day/time, or change the care provider either through the patient portal).
- Reminders about the appointment and importance of follow up primary care at 2-4 points during the postpartum period via the patient portal.
- Tailored language in the reminders based on recent evidence from behavioral science about the most effective approaches to increasing take-up. For example, messages will inform the patient that an appointment has been reserved for them at their doctor.

The Logic Framework that underpins the basis for this study is shown below:

Needs/ Problems	Barriers to Primary Care Use	Input/Intervention	Outcomes	Long-term Goals
• Low PCP use among people who have/are at risk of chronic conditions (diabetes, hypertension, obesity, mental illness)<br>• Pregnancy is ideal opportunity to interrupt progression of chronic disease, but this window into long-term health is often missed	• Insufficient patient information<br>• Underestimate risks<br>• Low salience<br>• Cognitive demands in postpartum period<br>• Time constraints and competing priorities<br>• Health system/structural barriers to postpartum care continuity<br>• Poor transitions between obstetricians and PCPs	• Targeted information about importance of primary care<br>• Default scheduling of postpartum primary care appointment<br>• Reminder messages about primary care appointment leveraging salience and pre-commitment<br>• Salient labeling of the transition appointment<br>• PCP messaging about a patient's recent pregnancy and upcoming follow-up	**PRIMARY**<br>• Visit with primary care provider within 4 months of childbirth<br>**SECONDARY**<br>• Condition-specific screening:<br>-documentation of blood pressure<br>-diabetes screening test<br>-measurement of weight<br>• Counseling on risk prevention or reduction strategies at visit<br>• Use of emergency room or urgent care for any reason	• Engagement with PCP<br>• Earlier, more effective chronic disease prevention and management<br>• Interruption of disease progression over life course<br>• Improved lifelong health status

The flow of study activities is shown below:

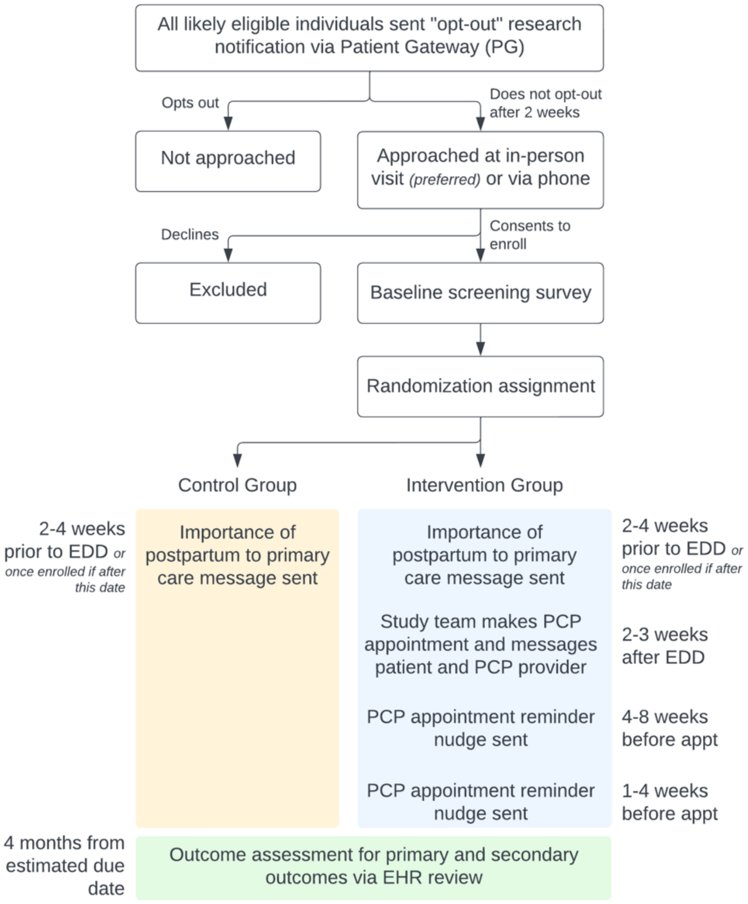

4. Subject Selection

Pregnant patients receiving prenatal care at Massachusetts General Hospital (MGH) will be targeted for recruitment. MGH conducts approximately 3500 deliveries per year, with roughly one-third of patients identifying as non-white and 35% of pregnancies covered by Medicaid.

Approximately 49% of the clinic population may ultimately be eligible to be approached. The eligibility criteria include:

- Estimated date of delivery and the following 4-month postpartum outcome assessment window completed prior to study end date
- Currently pregnant or within 2 weeks of estimated date of delivery
- Have one or more of the following conditions:
  - Chronic hypertension
  - Hypertensive disorders of pregnancy or risk factors for hypertensive disorders of pregnancy per the USPTF aspirin prescribing guidelines (e.g., history of pre-eclampsia, kidney disease, multiple gestation, autoimmune disease)[22]
  - Type 1 or 2 diabetes

    - Gestational diabetes
    - Obesity (body mass index ≥30 kg/m2)
    - Depression or anxiety disorder
- Have a primary care provider listed in the patient's medical record
- Receive obstetric care at an MGH-affiliated outpatient prenatal clinic
- Has access to and be enrolled in the electronic health record patient portal and consents to be contacted via these modalities
- Able to read/speak English or Spanish language
- Age ≥18 years old
- Not diagnosed with or undergoing evaluation for stillbirth/fetal demise

All women, regardless of race/ethnicity, who meet the eligibility criteria will be included. In 2019, 3,789 women gave birth at the study institution, of whom 57% were white, 7% were black, 12% were Asian, 18% were Hispanic, and 6% declined to report their race/ethnicity. A similar distribution is expected for this study.

The study will distribute materials in English and Spanish languages. This encompasses >95% of patients who deliver at the study institution.

5. Subject Enrollment

This study will rely on recruiting for research through Patient Gateway and follow the IRB guidance and DHeC Research Checklist and training for this process.

In the month prior to the start of enrollment, all potentially eligible individuals (based on the criteria above) will be identified using RPDR and Epic Reporting search queries. This list (the "potentially eligible" list) will be provided to the DHeCare Research Team to build an RSH Record in Epic. This list will be updated monthly to identify newly eligible individuals (e.g., new diagnosis of gestational diabetes or new patient transferring into the practice) during the recruitment months and fed back to the DHeCare team to update "potentially eligible" list.

Once built and each month during recruitment phase, the study's research coordinator (not study investigators) will send the IRB-approved Research Invitation Letter to patients who are eligible, not already enrolled, and have not declined to be sent research notifications through the portal. The Research Letter will employ an opt-out approach, asking individuals who do not wish to be approached in clinic or remotely to respond via PG messaging, email, or phone within 2 weeks of receipt of the letter. Those who have "read" the letter and not opted out after 2 weeks will be moved from the "potentially eligible" list to the "waiting to be approached" list in the study workflow.

During recruitment months, the study coordinator will keep a log of patient's upcoming appointments for those on the "waiting to be approached" list. They will then attempt to approach individuals for enrollment when they present for an in-person encounters (preferred). Patients will ideally be approached between 32-36 weeks of gestation, when feasible; however, priority will be given to patients at the latest gestation. For those Spanish-speaking patients, a hospital-based interpreter will be used when approaching/consenting patients.

The study staff will introduce the study and review the purpose of the study, the nature of the subject's participation, the possible risks and discomforts associated with participation, the potential benefits of participation, a statement of the voluntary nature of participation, and a description of the mechanisms used to ensure confidentiality.

All patients will receive study-related messages through Patient Gateway. In addition, patients will be asked if they would be willing to receive no more than 5 SMS messages to their personal cell phone during the first 4 months of the postpartum period. The study staff will review the specific concerns and risks about receiving unencrypted text messaging communications, as outlined by the MGB IRB.

A waiver of documentation of informed consent is requested, as the study presents no more than minimal risk of harm to subjects and involves no procedures for which written consent is normally required outside of the research context. Verbal consent will be obtained for 1) overall study participation and 2) optional SMS message participation, separately. Verbal consent will be obtained from both in-person and phone recruitment efforts.

A Study Fact Sheet, which summarizes the study details, risks, and benefits, will be provided to all subjects who are approached, either in-person, by mail, or electronically. This Study Fact Sheet also includes information supplied by the MGB IRB specifically related to the concerns and risks of receiving unencrypted SMS/text messages.

For tracking verbal consent, the study staff will keep a detailed log in REDCap documenting:

1) name of study staff performing consent,
2) date of attempted approach,
3) the method of attempted approach (in-person or via phone),
4) use of Spanish interpreter (yes/no),
5) subject agreement to be approached (agree/disagree),
6) attestation to full review of the study procedures/risks/benefits with the subject, as would be done during the process of reviewing a written consent form,
7) attestation to review of supplemental consent to receive unencrypted SMS messages with the subject,
8) subject overall study participation status (enrolled, declined, deferred – agrees to be recontacted, deferred – wishes not to be recontacted),
9) if enrolled, unencrypted SMS text messaging participation (consents, declines),
10) attestation to Study Fact Sheet provided,
11) method by which Study Fact Sheet was provided (in-person, mail, electronic),
12) date in which Study Fact Sheet was provided (in-person, mail, electronic).

6. STUDY PROCEDURES

The RA will keep a detailed log of all patients in the practice, if they are eligible, if they have been approached, and if they consented.

For those that agree to be enrolled, patients will be asked to complete a baseline survey to obtain voluntarily reported information on their demographics, socioeconomic status, health care visit history, and primary care provider. Patients will also be asked to consent to being contacted

by the research team via Patient Gateway messaging. A $20 gift card will be given at the time of enrollment for those that complete the questionnaire and receive the information sheet.

Randomization will occur via a prespecified random allocation sequence within strata. Within each stratum, the PIs will generate a random sequence of treatment-control allocation prior the enrollment of subjects. Then, as patients within strata are enrolled, they will be assigned to the treatment arm associated with that enrollment number.

Control Group
Approximately 2 weeks before a patient's estimated due date (EDD) or as soon as enrolled if this date has passed, the patient will be sent information via the Patient Gateway on the importance of postpartum care and follow-up with their PCP.

Intervention Group
1. Tailored Information:
   Approximately 2-4 weeks prior to the EDD (or later for those who are enrolled beyond 38 weeks gestation or postnatally), the patient will be sent an information via the Patient Gateway on the importance of postpartum care and follow-up with their PCP, which also includes the name and phone number of their primary care provider.
2. Scheduled PCP Appointment:
   Between 2-3 weeks after their delivery, the RA will call the patient's PCP office and make an appointment for them between 3-4 months after delivery based on the scheduling preferences obtained in the initial survey.
3. Targeted Appointment Message with Salient Labeling:
   After the PCP appointment has been made, the patient will be sent a Patient Gateway message saying that a PCP appointment has been reserved for them with the date/time/location information.
   For those consenting to receive SMS messages, an unencrypted text message will also be sent simultaneously.
4. Nudge Reminders:
   Patient Gateway messages will be sent at approximately 4-8 weeks (goal: 4 weeks) and 1-4 week (goal: 1 week) prior to their PCP appointment, reminding a patient of their upcoming appointment.
   For those consenting to receive SMS messages, an unencrypted text message will also be sent simultaneously.
5. Facilitated PCP Communication:
   A study staff member will send the patient's PCP an Epic Inbasket message that the patient 1) is recently postpartum, 2) has or developed health conditions that need long-term management, and 3) has been scheduled (or attempted to be scheduled) for a follow-up visit.

All messages will be made available in English and Spanish.

The text for these Patient Gateway messages is included in the submission. At the end of each patient message, patients will be given the opportunity to stop receiving study-related messages by emailing or calling the study staff. "Opt out" requests will be logged and patients removed from future planned study-related contact.

The text for 3 SMS messages is included in the submission. SMS messages will be sent via Google Voice. We will follow standard recommendations from the MGB Research Information Security for using this system (included under Privacy and Confidentiality section).

The study team will review the patient's EHR record for the primary and secondary outcome assessments. Patients will be asked to consent to have records reviewed up to 2 years after the date of their delivery to allow for long-term effects of the intervention on primary care use and health status.

During the initial consent process, patients will be asked to agree to be potentially contacted at the end of the first 4-month follow-up period for a survey. Currently, this endline survey is not planned due to funding limitations; however, if funding becomes available, an IRB amendment will be submitted for review of the endline survey prior to being administered to any study subjects.

Deidentified data from this project may be shared outside of MGB with the study funders (J-PAL/NBER/NIA) for data sharing and reproducibility requirements and secondary statistical analysis. All data will be stripped of patient identifiers, per IRB guidance. No data will be shared without a formal Data Use Agreement with MGB.

6. Risks and Discomforts

There are minimal risks to participants.

Patients will be reassured that nonparticipation will not affect clinical care. Patients will also be informed that the researchers with whom they will interact (e.g., during consent, face-to-face, during telephone interviews) are not health care providers. Participants will be consistently reminded that responses to any queries deemed sensitive or uncomfortable (e.g., country of origin/immigration status, income, previous history of abortion) should be considered optional, and they may decline to answer any question(s) and can refuse to continue the study at any point. Participants will be reassured that neither their opportunities for continued health care nor their relationships with health care providers will be jeopardized by study participation.

Health information collected as part of this study will be stored in REDCap. No identifiable data will be stored or downloaded on any personal or unauthorized computers. Study staff will access the data on institutionally purchased and managed computers that operate behind the health system's security and firewall protections. Only the MGH study staff will have access to the identifiable data set. Once the study is completed, data will be deidentified such that it can be analyzed without risk of a breach of privacy or confidentiality. Any data that is shared outside of MGH will require a data use authorization.

Although we will make every effort to protect participant privacy and confidentiality, it is possible that participants' involvement in the study could become known to others. For those in the intervention group, we will be sending personalized messages via the patient portal; for those providing additional consent, we will be sending unencrypted SMS messages that will not contain any personal protected health information. There is the rare possibility that study participation or health information could become known to others despite the use of firewalls,

password protection, and other security measures. Standard procedures, as outlined by the MGB Research Information Security Office, will be followed to reduce this risk.

Individuals in the intervention group will be scheduled for an appointment with a primary care doctor. There is the possibility that this visit may result in a charge/cost for the patient. Under the Affordable Care Act, commercial health plans are required to cover an annual Preventive Health Exam at no cost to the patient (no co-payment, co-insurance or deductible). MassHealth also covers these visits without cost-sharing. However, if this visit turns or scheduled as into a “sick” or “disease management” visit, the patient may be billed for some or all aspects of the services provided, depending on their insurer. We will provide directions for patients on how to to contact their insurers or PCP’s office prior to the visit to inquire about potential cost-sharing and/or deductibles. Patients will also be advised that some clinics may penalize individuals for not showing to a scheduled appointment (“no show” fee) and be given opportunities to request the appointment be canceled or rescheduled with each appointment reminder.

7. Benefits

Participants in the control group will receive information on the importance of postpartum care and transitioning to primary care after their delivery.

Participants in the intervention group will receive a bundle of interventions designed to increase attendance at primary care visits and facilitate the transition of care after their delivery.

The goal and potential benefits to the subjects in this trial is to increase patient engagement and connection with their primary care provider, receive recommended health screenings and directed counseling, and reduce unscheduled or urgent visits in the postpartum period.

8. Statistical Analysis

Statistical Methods
Analyses will be performed according to the intention-to-treat principle.

Standard independent, two-sided, two-group comparison testing will be used to compare baseline characteristics between the two groups (chi squared tests, t tests, Wilcoxon rank sum tests, when appropriate).

The primary outcome will be attendance rates at PCP visit within 4 months after estimated date of delivery (captured at time of enrollment), which will be compared between the groups using chi squared tests. Relative risks and 95% confidence intervals will be reported.

Secondary outcomes will include measures of long-term health and health care use after the postpartum period.

Subgroup analyses will be performed by a variety of patient characteristics including gestational age at enrollment, prenatal risk factors, morbidity types, patient race-ethnicity, payer, and enrollment location.

P-value of less than 0.05 will be considered to indicate statistical significance.

Power Calculation
Most study calculations were estimated from the MGH 2020 delivery population. The rates of PCP follow-up were estimated from a randomly selected cohort of 50 patients who met the inclusion criteria. We plan to recruit patients into the study for a period of 4 months and expect 1200 unique patients to be at the targeted gestational age during this period. Among these, we expect 86% to already have an assigned PCP in the network, leaving roughly 1,032 patients. Among these, we estimate that 49% have at least one of the targeted health conditions, leaving a target study population of 506 over the 4-month period. Based on previous studies conducted at MGH, we expect an 70% willingness to participate in the research, leaving an expected recruited population of 354 individuals. Based on our record extraction, we estimate that 15% of the targeted study population has a primary care visit within 4 months of delivery. Assuming an alpha of 0.05 and a baseline mean of 15%, with this expected sample size and power of 80%, our study has a minimum detectable effect size of roughly 13 percentage points (from 15% to 28%). A previous study found that default scheduling into postpartum care appointments (with an OBGYN, not a PCP) increased postpartum care take-up by 24 percentage points; since our intervention incorporates defaults and other activating interventions, an MDE of 13 percentage points is reasonable.

9. Monitoring and Quality Assurance

This is a minimal risk study in which the intervention involves default scheduling of appointments and patient messaging. Adverse events are not expected, and there is no physiologic plausibility for this intervention to cause any NIH-defined serious adverse events (e.g., death, prolonged hospitalization, significant disability).

No interim analyses are planned.

Adverse events will be defined and classified in accordance with NIH guidelines:

> Definition of Adverse Events (AE): Any untoward or unfavorable medical occurrence in a human subject, including any abnormal sign, symptom, or disease, temporally associated with the subject's participation in the research, whether considered related to the subject's participation in the research or not.
>
> Definition of Serious Adverse Events (SAE): Any AE that (1) results in death, (2) is life-threatening, (3) results in inpatient hospitalization or prolongation of existing hospitalization, (4) results in persistent or significant disability/incapacity, (5) results in a congenital anomaly/birth defect, and/or (6) may jeopardize the subject's health and may require medical or surgical intervention to prevent one of the other five outcomes listed here.

In the unlikely event an AE or SAE occurs, it will be brought to the PI's attention, and the PI will classify the AE/SAE by severity, expectedness, and relatedness,as listed above. All events that are both serious and unexpected will be reported to Mass General Brigham's IRB, the NIA

PO, and to the NIA Roybal DSMB within 48 hours of the research team's knowledge of the SAE. The summary of all other SAEs will be reported to the NIA and to the DSMB quarterly unless otherwise specified by the DSMB. Any unanticipated problem, defined as an issue related to the research suggesting the research places participants or others at greater risk than expected, will be reported to the IRB, the NIA PO, and to the Roybal DSMB within 48 hours of discovery. If the problem involves death then reporting will occur within 24 hours, and this report will include a plan to correct the problem and prevent its occurrence. Any breach of PHI will be reported to the PI, who will report to the IRB and NIA PO within 24 hours of discovery.

The Roybal DSMB oversight is provided by the Standing Roybal DSMB, which includes the members listed: Andrea B. Troxel, ScD (chair); Abby King, PhD; Jerry Gurwitz, MD;Hae-Ra Han, PhD, RN, FAAN; Hang Lee, PhD; Ezra Golberstein, PhD; David Kim, MD PhD; Christopher Celano, MD.

DSMB members will have no direct involvement with the study or conflict of interest with the investigators or institutions conducting the study. Each member has signed a COI statement which includes current affiliations, if any, with pharmaceutical or biotechnology companies (e.g., stockholder, consultant), and any other relationship that could be perceived as a conflict of interest related to the study and/or associatedwith commercial interests pertinent to study objectives.

Data presented to the DSMB will be deidentified as to protect individual participants' privacy and health information. Should the identity of a deidentified subject need to be revealed, the DSMB request will be reviewed and ultimately at the discretion of the Mass General Brigham IRB.

10. Privacy and Confidentiality

- ☒ Study procedures will be conducted in a private setting
- ☒ Only data and/or specimens necessary for the conduct of the study will be collected
- ☒ Data collected (paper and/or electronic) will be maintained in a secure location with appropriate protections such as password protection, encryption, physical security measures (locked files/areas)
- ☐ Specimens collected will be maintained in a secure location with appropriate protections (e.g. locked storage spaces, laboratory areas)
- ☒ Data and specimens will only be shared with individuals who are members of the IRB-approved research team or approved for sharing as described in this IRB protocol
- ☒ Data and/or specimens requiring transportation from one location or electronic space to another will be transported only in a secure manner (e.g. encrypted files, password protection, using chain-of-custody procedures, etc.)
- ☒ All electronic communication with participants will comply with Mass General Brigham secure communication policies
- ☒ Identifiers will be coded or removed as soon as feasible and access to files linking identifiers with coded data or specimens will be limited to the minimal necessary members of the research team required to conduct the research
- ☒ All staff are trained on and will follow the Mass General Brigham policies and procedures for maintaining appropriate confidentiality of research data and specimens

☒ The PI will ensure that all staff implement and follow any Research Information Service Office (RISO) requirements for this research
☒ Additional privacy and/or confidentiality protections

The following procedures (as provided by MGB RISO) will be used for sending SMS messages via Google Voice:

- Google Account:
    - A separate account should be created just for the purpose of the effort/study (in other words, personal Gmail accounts should not be used)
    - For the Google Voice portal, make sure you are not using the same password as your MGB account and ensure the password is strong. (Minimum of 8 characters, alphanumeric, uppercase, lowercase, special character).
        - Two-factor authentication must be enabled
    - Google account will not be shared
    - The Google account used for Google Voice should not be used for emailing or using any other Google Service (i.e., YouTube, Calendar, Contacts, etc.)
    - No credit cards should be added to the Google Account
    - The Google account must be deleted at the end of the study / project
- Participants will be informed not to send personal or health related information via text
- Siri will not be integrated with Google Voice
- Text messages will not address participants by their first name
- Text messages will be sent through the McLean email within the Google account that is created
- Only phone numbers and a unique subject ID will be stored in Google Voice
    - Log records should be deleted manually after 30 days
- No PHI or sensitive information will be communicated via text message
    - Content will not include anything where a healthcare condition or diagnosis can be inferred
- Text message history should be deleted from Google Voice account when no longer needed (within 30 days)
- Study staff will track opt-out requests and delete phone numbers from Google Voice as necessary
- Study staff must not communicate with participants via a group text message
- Access to the portal and overall research must be done from systems that meet MRB RISO compliance requirements; encryption, MobileIron (if Smartphone/Tablet), up to date malware protection, Crowdstrike.
    - https://rc.partners.org/security/secure-your-computer
- Participants:
    - Participants will be texted only if they consent
    - Participants should be informed to delete text messages when no longer needed and hide text push notifications

12. References

1. Boersma P. Prevalence of Multiple Chronic Conditions Among US Adults, 2018. *Prev Chronic Dis*. 2020;17. doi:10.5888/pcd17.200130
2. Home page | United States Preventive Services Taskforce. Accessed December 15, 2021. https://www.uspreventiveservicestaskforce.org/uspstf/
3. Blewett LA, Johnson PJ, Lee B, Scal PB. When a usual source of care and usual provider matter: adult prevention and screening services. *J Gen Intern Med*. 2008;23(9):1354-1360. doi:10.1007/s11606-008-0659-0
4. O'Malley AS, Mandelblatt J, Gold K, Cagney KA, Kerner J. Continuity of care and the use of breast and cervical cancer screening services in a multiethnic community. *Arch Intern Med*. 1997;157(13):1462-1470.
5. DeVoe JE, Saultz JW, Krois L, Tillotson CJ. A medical home versus temporary housing: the importance of a stable usual source of care. *Pediatrics*. 2009;124(5):1363-1371. doi:10.1542/peds.2008-3141
6. Atlas SJ, Grant RW, Ferris TG, Chang Y, Barry MJ. Patient-physician connectedness and quality of primary care. *Ann Intern Med*. 2009;150(5):325-335. doi:10.7326/0003-4819-150-5-200903030-00008
7. Levine DM, Linder JA, Landon BE. Characteristics of Americans With Primary Care and Changes Over Time, 2002-2015. *JAMA Internal Medicine*. 2020;180(3):463-466. doi:10.1001/jamainternmed.2019.6282
8. NVSS - Birth Data. Published June 14, 2021. Accessed September 1, 2021. https://www.cdc.gov/nchs/nvss/births.htm
9. Prenatal care and tests | Office on Women's Health. Accessed December 15, 2021. https://www.womenshealth.gov/pregnancy/youre-pregnant-now-what/prenatal-care-and-tests
10. Centers for Disease Control and Prevention (CDC). Adult Obesity Facts. Published August 29, 2017. Accessed February 14, 2018. https://www.cdc.gov/obesity/data/adult.html
11. Mental Illness. National Institute of Mental Health (NIMH). Accessed December 17, 2021. https://www.nimh.nih.gov/health/statistics/mental-illness
12. Products - Data Briefs - Number 289 - October 2017. Published June 6, 2019. Accessed December 17, 2021. https://www.cdc.gov/nchs/products/databriefs/db289.htm
13. National Diabetes Statistics Report 2020. Estimates of diabetes and its burden in the United States. Published online 2020:32.
14. Sattar N, Greer IA. Pregnancy complications and maternal cardiovascular risk: opportunities for intervention and screening? *BMJ*. 2002;325(7356):157-160. doi:10.1136/bmj.325.7356.157
15. Kim C, Tabaei BP, Burke R, et al. Missed Opportunities for Type 2 Diabetes Mellitus Screening Among Women With a History of Gestational Diabetes Mellitus. *Am J Public Health*. 2006;96(9):1643-1648. doi:10.2105/AJPH.2005.065722
16. Media BKK. Publications & Guidelines | SMFM.org - The Society for Maternal-Fetal Medicine. Accessed October 18, 2021. https://www.smfm.org/publications/225-smfm-statement-implementation-of-the-use-of-antenatal-corticosteroids-in-the-late-preterm-birth-period-in-women-at-risk-for-preterm-delivery
17. Yee LM, Simon MA, Grobman WA, Rajan PV. Pregnancy as a "golden opportunity" for patient activation and engagement. *American Journal of Obstetrics & Gynecology*. 2021;224(1):116-118. doi:10.1016/j.ajog.2020.09.024

18. Cohen JL, Daw JR. Postpartum Cliffs—Missed Opportunities to Promote Maternal Health in the United States. *JAMA Health Forum*. 2021;2(12):e214164. doi:10.1001/jamahealthforum.2021.4164
19. Milkman KL, Patel MS, Gandhi L, et al. A megastudy of text-based nudges encouraging patients to get vaccinated at an upcoming doctor's appointment. *PNAS*. 2021;118(20). doi:10.1073/pnas.2101165118
20. Patel MS, Volpp KG, Asch DA. Nudge Units to Improve the Delivery of Health Care. *N Engl J Med*. 2018;378(3):214-216. doi:10.1056/NEJMp1712984
21. Thaler RH, Sunstein CR. *Nudge: The Final Edition*. Revised edition. Penguin Books; 2021.
22. Henderson JT, Vesco KK, Senger CA, Thomas RG, Redmond N. Aspirin Use to Prevent Preeclampsia and Related Morbidity and Mortality: Updated Evidence Report and Systematic Review for the US Preventive Services Task Force. *JAMA*. 2021;326(12):1192. doi:10.1001/jama.2021.8551

Mass General Brigham

Title: Bridging the Gap from Postpartum to Primary Care: A Behavioral Science Informed Intervention to Improve Chronic Disease Management among Postpartum Women (the Bridge Study)

Sponsor Name:

PI Name: Clapp, Mark A **Protocol #:** 2022P001723 **Type:** Amendment (AME9)

Date Received: January 26, 2023

Signatures

PI Name: Clapp, Mark A, MD, MPH

Authenticated: January 26, 2023

Amendment

COVID-19 Amendment

Is this amendment ONLY related to research impacted by COVID-19?

Refer to MGB Policy on Conduct of Human Research Activities during COVID-19 Operations for description of Amendments which do **require prior IRB review and approval.**

- ◯ Yes
- ◉ No

Central IRB Performance Sites

Is this a protocol where the Mass General Brigham IRB is serving as the single IRB (sIRB) for external sites/institutions?

- ◉ Yes
- ◯ No

Would you like to 'Add' a Site?

- ◯ Yes
- ◉ No

Would you like to 'Remove' a Site?

- ◯ Yes
- ◉ No

Sponsor Amendment

Is there a sponsor amendment number?

- ◯ Yes
- ◉ No

Change in Protocol Status

Is this a cede protocol or project that was determined to be exempt, not human subjects research or not engaged in human subjects research?

- ◯ Yes
- ◉ No

Mass General Brigham

Do you need to change the overall status of the protocol? For example, Re-Open to Enrollment or indicate that Research Interventions/Assessments Continue after telling the IRB these have ceased.

- ◯ Yes
- ◉ No

Briefly describe the proposed changes:

The protocol is being amended to include a endline survey among participants enrolled in the study. Consent to contact patients for the survey was approved as part of the initial IRB approval. This amendment adds the survey tool, the administration procedure, and the text of the mail and SMS messages that will be used. The study schema was updated to add "and patient survey" in the bottom outcomes assessment box.

There are no changes to the Study Fact Sheet or consent process as this endline survey was already included.

Provide rationale for the proposed changes:

An endline survey was planned during the conception of this project, but the final tool was developed after the initial study protocol was approved. No individuals that have been enrolled have yet to reach the "4 months after due date" follow-up window yet -- the starting time point for the outcomes assessment.

Will the proposed change(s) significantly alter the risk to benefit assessment the IRB relied upon to approve the protocol?

- ◯ Yes
- ◉ No

Will the proposed change(s) significantly affect the integrity of the protocol?

- ◯ Yes
- ◉ No

Informed Consent

Do the changes require a revision to the consent form?

- ◯ Yes
- ◉ No

Protocol Key Areas

Choose up to 3 key areas below that describes this research protocol. Once you select a key area, you may be able to select additional specialty areas.

- ☐ Anesthesiology
- ☐ Colon and Rectal surgery
- ☐ Dermatology
- ☐ Emergency Medicine

Mass General Brigham

☑ Family Medicine

Choose all that apply:

- ☐ Adolescent Medicine
- ☐ Geriatric Medicine
- ☐ Hospice & Palliative Medicine
- ☐ Pain Medicine
- ☐ Sleep Medicine
- ☐ Sports Medicine
- ☐ Not Applicable

☑ Internal Medicine

Choose all that apply:

- ☐ Adolescent Medicine
- ☐ Adult Congenital Heart Disease
- ☐ Advanced Heart Failure & Transplant
- ☐ Cardiology
- ☐ Cardiovascular Disease
- ☐ Clinical Cardiac Electrophysiology
- ☐ Critical Care Medicine
- ☐ Endocrinology, Diabetes & Metabolism
- ☐ Gastroenterology
- ☐ Geriatric Medicine
- ☐ Hematology
- ☐ Hospice & Palliative Medicine
- ☐ Infectious Disease
- ☐ Interventional Cardiology
- ☐ Medical Oncology
- ☐ Nephrology
- ☐ Neurocritical Care
- ☐ Pulmonary Disease
- ☐ Rheumatology
- ☐ Sleep Medicine
- ☐ Sports Medicine
- ☐ Transplant Hepatology
- ☐ Not Applicable

☐ Medical Genetics and Genomics

☐ Neurological Surgery

☐ Nuclear Medicine

☑ Obstetrics and Gynecology

Choose all that apply:

- ☐ Complex Family Planning
- ☐ Critical Care Medicine
- ☐ Female Pelvic Medicine & Reconstructive Surgery
- ☐ Gynecologic Oncology
- ☐ Hospice & Palliative Medicine

- ☑ Maternal-Fetal Medicine
- ☐ Reproductive Endocrinology & Infertility
- ☐ Not Applicable

- ☐ Ophthalmology
- ☐ Orthopedic Surgery
- ☐ Otolaryngology: Head & Neck Surgery
- ☐ Pathology
- ☐ Pediatrics
- ☐ Physical Medicine and Rehabilitation
- ☐ Plastic Surgery
- ☐ Preventive Medicine
- ☐ Psychiatry and Neurology
- ☐ Radiology
- ☐ Surgery
- ☐ Thoracic Surgery
- ☐ Urology

Remuneration

Will subjects be paid or receive any type of remuneration / compensation for their time and expenses?

- ◉ Yes
- ○ No

Payment for Participation in Research

Indicate the type and total amount of compensation for completion of the study.

- ☐ Cash
- ☐ Check
- ☑ Gift Certificate

Amount e.g., $50, enter 50.00

20

- ☑ Other

Amount e.g., $200, enter 200.00

25

Explain:

An additional $25 gift card is being provided to individuals who opt to complete the end line survey.

NOTE: Payments to subjects must be made by check if payment involves a one-time payment of greater than $50 OR multiple payments of any amount. See policies Payments to Human

Mass General Brigham

Subjects for Participation in Research and **Cash Control and Accountability for Payments to Human Subjects for Participation in Research**.

Reimbursement for Expenses Related to Participation in Research

If there is not a set amount for meals, parking, and transportation, then estimate or enter the maximum amount budgeted per subject.

- ☐ Meals
- ☐ Parking
- ☐ Transportation
- ☐ Other

Instruments / Questionnaires

Will the research involve the development of instruments, questionnaires, surveys, interviews, and/or focus group topics?

○ Yes
◉ No

Will the research involve the use of instruments, questionnaires, surveys, interviews, and/or focus group topics?

◉ Yes
○ No

List of Instruments / Questionnaires / Surveys / Interviews / Focus Group Topics

Enter the name of each of the instruments, questionnaires, surveys, interviews and/or focus group topics. Upload each of the instruments, questionnaires, surveys, interviews and/or focus group topics to the Attachments page. **Do not list any that are under development.**

Baseline Survey

Enter the name of each of the instruments, questionnaires, surveys, interviews and/or focus group topics. Upload each of the instruments, questionnaires, surveys, interviews and/or focus group topics to the Attachments page. **Do not list any that are under development.**

Endline Survey

Attachments

Name	Mode
Detailed Protocol_012623_v1-5_clean (Detailed Protocol)	Electronic

Mass General Brigham

Name	Mode
Bridge Survey Endline _ 012623 (Instrument/Questionnaire)	Electronic
Study Schema_012623 (Schema)	Electronic
Survey Email Text (Document for review)	Electronic
Survey Remider SMS Text (Document for review)	Electronic

Mass General Brigham

Institutional Review Board
Intervention/Interaction Detailed Protocol

Principal Investigator:	Mark Clapp, MD MPH (MGH), Jessica Cohen, PhD (HSPH)
Project Title:	Bridging the Gap from Postpartum to Primary Care: A Behavioral Science Informed Intervention to Improve Chronic Disease Management among Postpartum Women
Version Date:	01/26/2023
Version Name/Number:	v1.5

1. Background and Significance

Burden of Chronic Disease and the Role of Primary Care
Chronic health conditions affect millions of people in the US each year. In 2018, 51.8% of adults had at least 1 chronic condition, and 27.2% had multiple conditions.[1] The prevalence of chronic disease was higher in women compared to men, older adults (87.6% in adults ≥65 years old), and people with public insurance.[1] Many chronic conditions, by their nature, develop over time and have risk factors that can be identified prior to the onset of disease. Strong evidence underpins prevention strategies for many conditions, which are advanced by the US Preventative Services Taskforce.[2] While the long-term health of a patient is the responsibility of an entire health system, primary care providers (PCPs) provide an integral role in preventing, screening for, and managing disease across the lifespan. Studies have shown the health benefits of receiving regular care under a PCP.[3–6]

Despite the known benefits of having an identifiable usual source of care and the value of health care maintenance, the percent of the population with a PCP has been decreasing over time.[7] Consistently, adults who are younger (age 20-40 years) have the lowest rates of primary care use. In 2015, 44% and 36% of 20- and 30-year-olds had no identifiable source of primary care.[7] The proportion without primary care were also higher among racial/ethnic minority populations and among those who had less education, lower incomes, and no known comorbidities.[7] The number of adults and the time elapsed without regular primary care follow-up can be considered missed opportunities to improve a patient's current and long-term health. The disproportionate lack of primary care among certain subgroups of the population, often groups who already have worse health outcomes, only serves to widen the pre-existing disparities.

Pregnancy as a Window to Future Health
In the US, 98.2% of pregnant women receive some form of prenatal care, with the average patient having >10 visits during their pregnancy.[8] During a pregnancy, women are screened for pre-existing and pregnancy-related conditions.[9] In adults ages 18-39, the prevalence of obesity, hypertension, prediabetes/diabetes, and mental illness are estimated at 39%, 7.5%, 28%, 25%, respectively.[10–13] Even for those who have no prior identified comorbidities, the most common

pregnancy-related conditions—pregnancy-related hypertension and gestational diabetes (8% and 10% of pregnancies, respectively)—indicate a predisposition to or confer health risks that persist as women age. For example, over 25% of women with gestational diabetes will develop Type 2 diabetes mellitus, and women with pre-eclampsia have more than a two-fold risk of significant cardiovascular disease later in life.[14,15] For these reasons, pregnancy is often considered a "window" into a woman's future health and presents a unique opportunity to optimize a woman's health status early in her life when she otherwise may not have been engaged in care.[16]

Pregnancy as on Opportunity for Engagement with a Long-term Care Provider
Pregnancy is a period when women are highly engaged and active participants in their health care.[17] It has been described as a "golden opportunity" to motivate women towards positive health behaviors, including prevention and management of chronic disease. However, women often fall off a "postpartum cliff" of health system engagement after the early postpartum period.[18] A range of systemic, financial, and behavioral barriers often prevents patients from effectively transitioning to primary care. Postpartum women are often simply told to follow-up with their PCP without much information regarding the importance of this follow up care, without assistance in scheduling an appointment (or identifying a PCP if they don't have one), and often without a direct transfer of relevant health information or accountability across providers.

Postpartum women are left largely on their own to navigate this transition to primary care and, in particular, to navigate it at a time when they face the high cognitive and physical demands of caring for an infant. At this time of limited cognitive bandwidth, the importance of continuity of care for chronic conditions and active engagement in one's longer-term health and wellbeing is unlikely to be salient and top-of-mind. These critical moments of unsupported health care transition can exacerbate pre-existing disparities in health and health care, with patients who are the least able to navigate the US health care system most likely to fall through the cracks. Momentum is building in US health and social safety net policy to facilitate healthy transitions from pregnancy to parenthood. For example, federal and state initiatives to expand pregnancy-related Medicaid coverage from 60 days to one year postpartum have been proposed, and access to paid family leave is increasing. However, very little evidence exists on effective and cost-effective approaches to facilitating transitions to primary care and management of chronic diseases in the postpartum year.

2. Specific Aims and Objectives

The objective of the proposed study is to increase patient engagement in primary care after the immediate postpartum period for women with pregnancy-associated conditions that convey a long-term health risk. Specifically, we aim to evaluate the efficacy of an intervention bundle (automatic scheduling of PCP appointment after delivery, salient labeling, and appointment reminder nudges) to increase patient attendance at a primary care provider appointment (within 4 months of delivery for women with or at risk for obesity, diabetes, hypertension, and/or a mental health condition.

Specific Aims:

1) Test the efficacy of an intervention bundle (patient-tailored health information, automatic scheduling of PCP appointment after delivery, and appointment reminder nudges) to increase patient attendance at a primary care provider appointment within 4 months of

delivery for women with obesity, diabetes, hypertension, and/or a mental health condition
2) Test the efficacy of the intervention bundle to improve compliance with the condition-specific, guideline-based health screenings
3) Test the efficacy of the intervention bundle to reduce unscheduled or urgent encounters (e.g., emergency department visits) within the health system

3. General Description of Study Design

We will conduct a randomized controlled trial comparing this intervention bundle to the receipt of generic information on the importance of primary care follow-up after delivery.

Women will be randomized with equal probability into either a treatment or control arm. The intervention combines several features designed to target reasons for low take-up of primary care among postpartum women (see Logic Model). We leverage the potential value of defaults/opt-out, salient labels, and reminders to encourage use of primary care within 4 months of delivery. Women in both the intervention and control arms will receive information via MGH's patient portal toward the end of the pregnancy regarding the importance of transitions to primary care in the postpartum year. This information will be similar to, but reinforcing, the information they would receive from their obstetrician about following up with their primary care physician. In addition to this initial message, women in the treatment arm will receive the following intervention components, developed based on recent evidence regarding behavioral science approaches to activating health behaviors:[19–21]

- Targeted messages about the importance and benefits of primary care.
- Default scheduling into a primary care appointment at approximately 3-4 months after delivery. The patient will be scheduled for a primary care visit with their assigned primary care provider in the Mass General Brigham system. They will be informed of the option to cancel the appointment, change the appointment day/time, or change the care provider either through the patient portal).
- Reminders about the appointment and importance of follow up primary care at 2-4 points during the postpartum period via the patient portal.
- Tailored language in the reminders based on recent evidence from behavioral science about the most effective approaches to increasing take-up. For example, messages will inform the patient that an appointment has been reserved for them at their doctor.

The Logic Framework that underpins the basis for this study is shown below:

Needs/ Problems	Barriers to Primary Care Use	Input/Intervention	Outcomes	Long-term Goals
• Low PCP use among people who have/are at risk of chronic conditions (diabetes, hypertension, obesity, mental illness)<br>• Pregnancy is ideal opportunity to interrupt progression of chronic disease, but this window into long-term health is often missed	• Insufficient patient information<br>• Underestimate risks<br>• Low salience<br>• Cognitive demands in postpartum period<br>• Time constraints and competing priorities<br>• Health system/structural barriers to postpartum care continuity<br>• Poor transitions between obstetricians and PCPs	• Targeted information about importance of primary care<br>• Default scheduling of postpartum primary care appointment<br>• Reminder messages about primary care appointment leveraging salience and pre-commitment<br>• Salient labeling of the transition appointment<br>• PCP messaging about a patient's recent pregnancy and upcoming follow-up	**PRIMARY**<br>• Visit with primary care provider within 4 months of childbirth<br>**SECONDARY**<br>• Condition-specific screening:<br>-documentation of blood pressure<br>-diabetes screening test<br>-measurement of weight<br>• Counseling on risk prevention or reduction strategies at visit<br>• Use of emergency room or urgent care for any reason	• Engagement with PCP<br>• Earlier, more effective chronic disease prevention and management<br>• Interruption of disease progression over life course<br>• Improved lifelong health status

The flow of study activities is shown below:

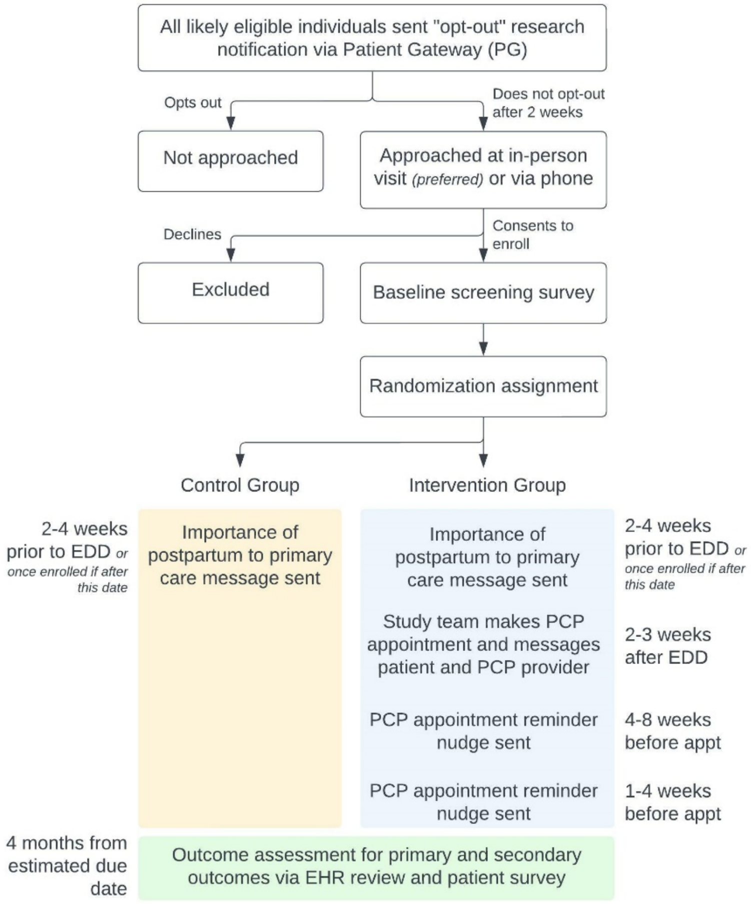

4. Subject Selection

Pregnant patients receiving prenatal care at Massachusetts General Hospital (MGH) will be targeted for recruitment. MGH conducts approximately 3500 deliveries per year, with roughly one-third of patients identifying as non-white and 35% of pregnancies covered by Medicaid.

Approximately 49% of the clinic population may ultimately be eligible to be approached.
The eligibility criteria include:

- Estimated date of delivery and the following 4-month postpartum outcome assessment window completed prior to study end date
- Currently pregnant or within 2 weeks of estimated date of delivery
- Have one or more of the following conditions:
    - Chronic hypertension
    - Hypertensive disorders of pregnancy or risk factors for hypertensive disorders of pregnancy per the USPTF aspirin prescribing guidelines (e.g., history of pre-eclampsia, kidney disease, multiple gestation, autoimmune disease)[22]
    - Type 1 or 2 diabetes

- Gestational diabetes
- Obesity (body mass index ≥30 kg/m2)
- Depression or anxiety disorder

- Have a primary care provider listed in the patient's medical record
- Receive obstetric care at an MGH-affiliated outpatient prenatal clinic
- Has access to and be enrolled in the electronic health record patient portal and consents to be contacted via these modalities
- Able to read/speak English or Spanish language
- Age ≥18 years old
- Not diagnosed with or undergoing evaluation for stillbirth/fetal demise

All women, regardless of race/ethnicity, who meet the eligibility criteria will be included. In 2019, 3,789 women gave birth at the study institution, of whom 57% were white, 7% were black, 12% were Asian, 18% were Hispanic, and 6% declined to report their race/ethnicity. A similar distribution is expected for this study.

The study will distribute materials in English and Spanish languages. This encompasses >95% of patients who deliver at the study institution.

5. Subject Enrollment

This study will rely on recruiting for research through Patient Gateway and follow the IRB guidance and DHeC Research Checklist and training for this process.

In the month prior to the start of enrollment, all potentially eligible individuals (based on the criteria above) will be identified using RPDR and Epic Reporting search queries. This list (the "potentially eligible" list) will be provided to the DHeCare Research Team to build an RSH Record in Epic. This list will be updated monthly to identify newly eligible individuals (e.g., new diagnosis of gestational diabetes or new patient transferring into the practice) during the recruitment months and fed back to the DHeCare team to update "potentially eligible" list.

Once built and each month during recruitment phase, the study's research coordinator (not study investigators) will send the IRB-approved Research Invitation Letter to patients who are eligible, not already enrolled, and have not declined to be sent research notifications through the portal. The Research Letter will employ an opt-out approach, asking individuals who do not wish to be approached in clinic or remotely to respond via PG messaging, email, or phone within 2 weeks of receipt of the letter. Those who have "read" the letter and not opted out after 2 weeks will be moved from the "potentially eligible" list to the "waiting to be approached" list in the study workflow.

During recruitment months, the study coordinator will keep a log of patient's upcoming appointments for those on the "waiting to be approached" list. They will then attempt to approach individuals for enrollment when they present for an in-person encounters (preferred). Patients will ideally be approached between 32-36 weeks of gestation, when feasible; however, priority will be given to patients at the latest gestation. For those Spanish-speaking patients, a hospital-based interpreter will be used when approaching/consenting patients.

The study staff will introduce the study and review the purpose of the study, the nature of the subject's participation, the possible risks and discomforts associated with participation, the potential benefits of participation, a statement of the voluntary nature of participation, and a description of the mechanisms used to ensure confidentiality.

All patients will receive study-related messages through Patient Gateway. In addition, patients will be asked if they would be willing to receive no more than 5 SMS messages to their personal cell phone during the first 4 months of the postpartum period. The study staff will review the specific concerns and risks about receiving unencrypted text messaging communications, as outlined by the MGB IRB.

A waiver of documentation of informed consent is requested, as the study presents no more than minimal risk of harm to subjects and involves no procedures for which written consent is normally required outside of the research context. Verbal consent will be obtained for 1) overall study participation and 2) optional SMS message participation, separately. Verbal consent will be obtained from both in-person and phone recruitment efforts.

A Study Fact Sheet, which summarizes the study details, risks, and benefits, will be provided to all subjects who are approached, either in-person, by mail, or electronically. This Study Fact Sheet also includes information supplied by the MGB IRB specifically related to the concerns and risks of receiving unencrypted SMS/text messages.

For tracking verbal consent, the study staff will keep a detailed log in REDCap documenting:

1) name of study staff performing consent,
2) date of attempted approach,
3) the method of attempted approach (in-person or via phone),
4) use of Spanish interpreter (yes/no),
5) subject agreement to be approached (agree/disagree),
6) attestation to full review of the study procedures/risks/benefits with the subject, as would be done during the process of reviewing a written consent form,
7) attestation to review of supplemental consent to receive unencrypted SMS messages with the subject,
8) subject overall study participation status (enrolled, declined, deferred – agrees to be recontacted, deferred – wishes not to be recontacted),
9) if enrolled, unencrypted SMS text messaging participation (consents, declines),
10) attestation to Study Fact Sheet provided,
11) method by which Study Fact Sheet was provided (in-person, mail, electronic),
12) date in which Study Fact Sheet was provided (in-person, mail, electronic).

6. STUDY PROCEDURES

The RA will keep a detailed log of all patients in the practice, if they are eligible, if they have been approached, and if they consented.

For those that agree to be enrolled, patients will be asked to complete a baseline survey to obtain voluntarily reported information on their demographics, socioeconomic status, health care visit history, and primary care provider. Patients will also be asked to consent to being contacted

by the research team via Patient Gateway messaging. A $20 gift card will be given at the time of enrollment for those that complete the questionnaire and receive the information sheet.

Randomization will occur via a prespecified random allocation sequence within strata. Within each stratum, the PIs will generate a random sequence of treatment-control allocation prior the enrollment of subjects. Then, as patients within strata are enrolled, they will be assigned to the treatment arm associated with that enrollment number.

<u>Control Group</u>
Approximately 2 weeks before a patient's estimated due date (EDD) or as soon as enrolled if this date has passed, the patient will be sent information via the Patient Gateway on the importance of postpartum care and follow-up with their PCP.

<u>Intervention Group</u>
1. Tailored Information:
   Approximately 2-4 weeks prior to the EDD (or later for those who are enrolled beyond 38 weeks gestation or postnatally), the patient will be sent an information via the Patient Gateway on the importance of postpartum care and follow-up with their PCP, which also includes the name and phone number of their primary care provider.
2. Scheduled PCP Appointment:
   Between 2-3 weeks after their delivery, the RA will call the patient's PCP office and make an appointment for them between 3-4 months after delivery based on the scheduling preferences obtained in the initial survey.
3. Targeted Appointment Message with Salient Labeling:
   After the PCP appointment has been made, the patient will be sent a Patient Gateway message saying that a PCP appointment has been reserved for them with the date/time/location information.
   For those consenting to receive SMS messages, an unencrypted text message will also be sent simultaneously.
4. Nudge Reminders:
   Patient Gateway messages will be sent at approximately 4-8 weeks (goal: 4 weeks) and 1-4 week (goal: 1 week) prior to their PCP appointment, reminding a patient of their upcoming appointment.
   For those consenting to receive SMS messages, an unencrypted text message will also be sent simultaneously.
5. Facilitated PCP Communication:
   A study staff member will send the patient's PCP an Epic Inbasket message that the patient 1) is recently postpartum, 2) has or developed health conditions that need long-term management, and 3) has been scheduled (or attempted to be scheduled) for a follow-up visit.

All messages will be made available in English and Spanish.

The text for these Patient Gateway messages is included in the submission. At the end of each patient message, patients will be given the opportunity to stop receiving study-related messages by emailing or calling the study staff. "Opt out" requests will be logged and patients removed from future planned study-related contact.

The text for 3 SMS messages is included in the submission. SMS messages will be sent via Google Voice. We will follow standard recommendations from the MGB Research Information Security for using this system (included under Privacy and Confidentiality section).

Outcome Assessment

1. EHR Review
The study team will review the patient's EHR record for the primary and secondary outcome assessments. Patients will be asked to consent to have records reviewed up to 2 years after the date of their delivery to allow for long-term effects of the intervention on primary care use and health status.

2. Endline Survey
After 4 months from their established due date (EDD), a patient will be eligible to participate the endline survey. The goal timing for the survey completion is 4-6 months after the EDD, but patients will remain eligible for up to one year.

The survey will be administered through REDCap. Study participants will be emailed a unique link to complete the survey. Consent to be contacted for this survey will be obtained at the time of the subject's enrollment in the trial. The patient's email address for contact is verified at the time of study enrollment.

Subjects will be asked to complete a 15-20-minute survey on their care after their delivery. They will be reminded that all questions are optional and that responses will be linked to their study record; however, no responses will be entered into the electronic health record or become part of their permanent medical record. Participants who complete the survey will be offered a $25 gift card for their participation, which they will be informed of prior to starting the survey. There is no penalty for not completing the survey.

If there is no response within approximately 1-2 weeks, the subject will be emailed the link again with a reminder to complete the survey. If there is no response within approximately 2-4 weeks from the initial email OR the patient does not have a working email address, the subject will be called and asked if they would like to complete the survey over the phone. For those that agree, the survey text and questions will be read verbatim by a research assistant not affiliated with the patient's clinical care. Individuals who decline to participate in the survey over the phone or cannot be reached will be marked as non-respondents.

Patients that consented to receive SMS messages as a part of the study will be sent a text message reminder to complete the survey at the same time that emails are sent. No more than 2 SMS message reminders will be sent to each subject.

Deidentified data from this project may be shared outside of MGB with the study funders (J-PAL/NBER/NIA) for data sharing and reproducibility requirements and secondary statistical analysis. All data will be stripped of patient identifiers, per IRB guidance. No data will be shared without a formal Data Use Agreement with MGB.

6. Risks and Discomforts

There are minimal risks to participants.

Patients will be reassured that nonparticipation will not affect clinical care. Patients will also be informed that the researchers with whom they will interact (e.g., during consent, face-to-face, during telephone interviews) are not health care providers. Participants will be consistently reminded that responses to any queries deemed sensitive or uncomfortable (e.g., country of origin/immigration status, income, previous history of abortion) should be considered optional, and they may decline to answer any question(s) and can refuse to continue the study at any point. Participants will be reassured that neither their opportunities for continued health care nor their relationships with health care providers will be jeopardized by study participation.

Health information collected as part of this study will be stored in REDCap. No identifiable data will be stored or downloaded on any personal or unauthorized computers. Study staff will access the data on institutionally purchased and managed computers that operate behind the health system's security and firewall protections. Only the MGH study staff will have access to the identifiable data set. Once the study is completed, data will be deidentified such that it can be analyzed without risk of a breach of privacy or confidentiality. Any data that is shared outside of MGH will require a data use authorization.

Although we will make every effort to protect participant privacy and confidentiality, it is possible that participants' involvement in the study could become known to others. For those in the intervention group, we will be sending personalized messages via the patient portal; for those providing additional consent, we will be sending unencrypted SMS messages that will not contain any personal protected health information. There is the rare possibility that study participation or health information could become known to others despite the use of firewalls, password protection, and other security measures. Standard procedures, as outlined by the MGB Research Information Security Office, will be followed to reduce this risk.

Individuals in the intervention group will be scheduled for an appointment with a primary care doctor. There is the possibility that this visit may result in a charge/cost for the patient. Under the Affordable Care Act, commercial health plans are required to cover an annual Preventive Health Exam at no cost to the patient (no co-payment, co-insurance or deductible). MassHealth also covers these visits without cost-sharing. However, if this visit turns or scheduled as into a "sick" or "disease management" visit, the patient may be billed for some or all aspects of the services provided, depending on their insurer. We will provide directions for patients on how to to contact their insurers or PCP's office prior to the visit to inquire about potential cost-sharing and/or deductibles. Patients will also be advised that some clinics may penalize individuals for not showing to a scheduled appointment ("no show" fee) and be given opportunities to request the appointment be canceled or rescheduled with each appointment reminder.

7. Benefits

Participants in the control group will receive information on the importance of postpartum care and transitioning to primary care after their delivery.

Participants in the intervention group will receive a bundle of interventions designed to increase attendance at primary care visits and facilitate the transition of care after their delivery.

The goal and potential benefits to the subjects in this trial is to increase patient engagement and connection with their primary care provider, receive recommended health screenings and directed counseling, and reduce unscheduled or urgent visits in the postpartum period.

8. Statistical Analysis

Statistical Methods
Analyses will be performed according to the intention-to-treat principle.

Standard independent, two-sided, two-group comparison testing will be used to compare baseline characteristics between the two groups (chi squared tests, t tests, Wilcoxon rank sum tests, when appropriate).

The primary outcome will be attendance rates at PCP visit within 4 months after estimated date of delivery (captured at time of enrollment), which will be compared between the groups using chi squared tests. Relative risks and 95% confidence intervals will be reported.

Secondary outcomes will include measures of long-term health and health care use after the postpartum period.

Subgroup analyses will be performed by a variety of patient characteristics including gestational age at enrollment, prenatal risk factors, morbidity types, patient race-ethnicity, payer, and enrollment location.

P-value of less than 0.05 will be considered to indicate statistical significance.

Power Calculation
Most study calculations were estimated from the MGH 2020 delivery population. The rates of PCP follow-up were estimated from a randomly selected cohort of 50 patients who met the inclusion criteria. We plan to recruit patients into the study for a period of 4 months and expect 1200 unique patients to be at the targeted gestational age during this period. Among these, we expect 86% to already have an assigned PCP in the network, leaving roughly 1,032 patients. Among these, we estimate that 49% have at least one of the targeted health conditions, leaving a target study population of 506 over the 4-month period. Based on previous studies conducted at MGH, we expect an 70% willingness to participate in the research, leaving an expected recruited population of 354 individuals. Based on our record extraction, we estimate that 15% of the targeted study population has a primary care visit within 4 months of delivery. Assuming an alpha of 0.05 and a baseline mean of 15%, with this expected sample size and power of 80%, our study has a minimum detectable effect size of roughly 13 percentage points (from 15% to 28%). A previous study found that default scheduling into postpartum care appointments (with an OBGYN, not a PCP) increased postpartum care take-up by 24 percentage points; since our intervention incorporates defaults and other activating interventions, an MDE of 13 percentage points is reasonable.

9. Monitoring and Quality Assurance

This is a minimal risk study in which the intervention involves default scheduling of appointments and patient messaging. Adverse events are not expected, and there is no physiologic plausibility for this intervention to cause any NIH-defined serious adverse events (e.g., death, prolonged hospitalization, significant disability).

No interim analyses are planned.

Adverse events will be defined and classified in accordance with NIH guidelines:

> Definition of Adverse Events (AE)**:** Any untoward or unfavorable medical occurrence in a human subject, including any abnormal sign, symptom, or disease, temporally associated with the subject's participation in the research, whether considered related to the subject's participation in the research or not.
>
> Definition of Serious Adverse Events (SAE): Any AE that (1) results in death, (2) is life-threatening, (3) results in inpatient hospitalization or prolongation of existing hospitalization, (4) results in persistent or significant disability/incapacity, (5) results in a congenital anomaly/birth defect, and/or (6) may jeopardize the subject's health and may require medical or surgical intervention to prevent one of the other five outcomes listed here.

In the unlikely event an AE or SAE occurs, it will be brought to the PI's attention, and the PI will classify the AE/SAE by severity, expectedness, and relatedness,as listed above. All events that are both serious and unexpected will be reported to Mass General Brigham's IRB, the NIA PO, and to the NIA Roybal DSMB within 48 hours of the research team's knowledge of the SAE. The summary of all other SAEs will be reported to the NIA and to the DSMB quarterly unless otherwise specified by the DSMB. Any unanticipated problem, defined as an issue related to the research suggesting the research places participants or others at greater risk than expected, will be reported to the IRB, the NIA PO, and to the Roybal DSMB within 48 hours of discovery. If the problem involves death then reporting will occur within 24 hours, and this report will include a plan to correct the problem and prevent its occurrence. Any breach of PHI will be reported to the PI, who will report to the IRB and NIA PO within 24 hours of discovery.

The Roybal DSMB oversight is provided by the Standing Roybal DSMB, which includes the members listed: Andrea B. Troxel, ScD (chair); Abby King, PhD; Jerry Gurwitz, MD;Hae-Ra Han, PhD, RN, FAAN; Hang Lee, PhD; Ezra Golberstein, PhD; David Kim, MD PhD; Christopher Celano, MD.

DSMB members will have no direct involvement with the study or conflict of interest with the investigators or institutions conducting the study. Each member has signed a COI statement which includes current affiliations, if any, with pharmaceutical or biotechnology companies (e.g., stockholder, consultant), and any other relationship that could be perceived as a conflict of interest related to the study and/or associatedwith commercial interests pertinent to study objectives.

Data presented to the DSMB will be deidentified as to protect individual participants' privacy and health information. Should the identity of a deidentified subject need to be revealed, the DSMB request will be reviewed and ultimately at the discretion of the Mass General Brigham IRB.

10. Privacy and Confidentiality

☒ Study procedures will be conducted in a private setting
☒ Only data and/or specimens necessary for the conduct of the study will be collected
☒ Data collected (paper and/or electronic) will be maintained in a secure location with appropriate protections such as password protection, encryption, physical security measures (locked files/areas)
☐ Specimens collected will be maintained in a secure location with appropriate protections (e.g. locked storage spaces, laboratory areas)
☒ Data and specimens will only be shared with individuals who are members of the IRB-approved research team or approved for sharing as described in this IRB protocol
☒ Data and/or specimens requiring transportation from one location or electronic space to another will be transported only in a secure manner (e.g. encrypted files, password protection, using chain-of-custody procedures, etc.)
☒ All electronic communication with participants will comply with Mass General Brigham secure communication policies
☒ Identifiers will be coded or removed as soon as feasible and access to files linking identifiers with coded data or specimens will be limited to the minimal necessary members of the research team required to conduct the research
☒ All staff are trained on and will follow the Mass General Brigham policies and procedures for maintaining appropriate confidentiality of research data and specimens
☒ The PI will ensure that all staff implement and follow any Research Information Service Office (RISO) requirements for this research
☒ Additional privacy and/or confidentiality protections

The following procedures (as provided by MGB RISO) will be used for sending SMS messages via Google Voice:

- Google Account:
    - A separate account should be created just for the purpose of the effort/study (in other words, personal Gmail accounts should not be used)
    - For the Google Voice portal, make sure you are not using the same password as your MGB account and ensure the password is strong. (Minimum of 8 characters, alphanumeric, uppercase, lowercase, special character).
        - Two-factor authentication must be enabled
    - Google account will not be shared
    - The Google account used for Google Voice should not be used for emailing or using any other Google Service (i.e., YouTube, Calendar, Contacts, etc.)
    - No credit cards should be added to the Google Account
    - The Google account must be deleted at the end of the study / project
- Participants will be informed not to send personal or health related information via text
- Siri will not be integrated with Google Voice

- Text messages will not address participants by their first name
- Text messages will be sent through the McLean email within the Google account that is created
- Only phone numbers and a unique subject ID will be stored in Google Voice
    - Log records should be deleted manually after 30 days
- No PHI or sensitive information will be communicated via text message
    - Content will not include anything where a healthcare condition or diagnosis can be inferred
- Text message history should be deleted from Google Voice account when no longer needed (within 30 days)
- Study staff will track opt-out requests and delete phone numbers from Google Voice as necessary
- Study staff must not communicate with participants via a group text message
- Access to the portal and overall research must be done from systems that meet MRB RISO compliance requirements; encryption, MobileIron (if Smartphone/Tablet), up to date malware protection, Crowdstrike.
    - https://rc.partners.org/security/secure-your-computer
- Participants:
    - Participants will be texted only if they consent
    - Participants should be informed to delete text messages when no longer needed and hide text push notifications

12. References

1. Boersma P. Prevalence of Multiple Chronic Conditions Among US Adults, 2018. *Prev Chronic Dis*. 2020;17. doi:10.5888/pcd17.200130
2. Home page | United States Preventive Services Taskforce. Accessed December 15, 2021. https://www.uspreventiveservicestaskforce.org/uspstf/
3. Blewett LA, Johnson PJ, Lee B, Scal PB. When a usual source of care and usual provider matter: adult prevention and screening services. *J Gen Intern Med*. 2008;23(9):1354-1360. doi:10.1007/s11606-008-0659-0
4. O'Malley AS, Mandelblatt J, Gold K, Cagney KA, Kerner J. Continuity of care and the use of breast and cervical cancer screening services in a multiethnic community. *Arch Intern Med*. 1997;157(13):1462-1470.
5. DeVoe JE, Saultz JW, Krois L, Tillotson CJ. A medical home versus temporary housing: the importance of a stable usual source of care. *Pediatrics*. 2009;124(5):1363-1371. doi:10.1542/peds.2008-3141
6. Atlas SJ, Grant RW, Ferris TG, Chang Y, Barry MJ. Patient-physician connectedness and quality of primary care. *Ann Intern Med*. 2009;150(5):325-335. doi:10.7326/0003-4819-150-5-200903030-00008
7. Levine DM, Linder JA, Landon BE. Characteristics of Americans With Primary Care and Changes Over Time, 2002-2015. *JAMA Internal Medicine*. 2020;180(3):463-466. doi:10.1001/jamainternmed.2019.6282
8. NVSS - Birth Data. Published June 14, 2021. Accessed September 1, 2021. https://www.cdc.gov/nchs/nvss/births.htm
9. Prenatal care and tests | Office on Women's Health. Accessed December 15, 2021. https://www.womenshealth.gov/pregnancy/youre-pregnant-now-what/prenatal-care-and-tests
10. Centers for Disease Control and Prevention (CDC). Adult Obesity Facts. Published August 29, 2017. Accessed February 14, 2018. https://www.cdc.gov/obesity/data/adult.html
11. Mental Illness. National Institute of Mental Health (NIMH). Accessed December 17, 2021. https://www.nimh.nih.gov/health/statistics/mental-illness
12. Products - Data Briefs - Number 289 - October 2017. Published June 6, 2019. Accessed December 17, 2021. https://www.cdc.gov/nchs/products/databriefs/db289.htm
13. National Diabetes Statistics Report 2020. Estimates of diabetes and its burden in the United States. Published online 2020:32.
14. Sattar N, Greer IA. Pregnancy complications and maternal cardiovascular risk: opportunities for intervention and screening? *BMJ*. 2002;325(7356):157-160. doi:10.1136/bmj.325.7356.157
15. Kim C, Tabaei BP, Burke R, et al. Missed Opportunities for Type 2 Diabetes Mellitus Screening Among Women With a History of Gestational Diabetes Mellitus. *Am J Public Health*. 2006;96(9):1643-1648. doi:10.2105/AJPH.2005.065722
16. Media BKK. Publications & Guidelines | SMFM.org - The Society for Maternal-Fetal Medicine. Accessed October 18, 2021. https://www.smfm.org/publications/225-smfm-statement-implementation-of-the-use-of-antenatal-corticosteroids-in-the-late-preterm-birth-period-in-women-at-risk-for-preterm-delivery
17. Yee LM, Simon MA, Grobman WA, Rajan PV. Pregnancy as a "golden opportunity" for patient activation and engagement. *American Journal of Obstetrics & Gynecology*. 2021;224(1):116-118. doi:10.1016/j.ajog.2020.09.024

18. Cohen JL, Daw JR. Postpartum Cliffs—Missed Opportunities to Promote Maternal Health in the United States. *JAMA Health Forum*. 2021;2(12):e214164. doi:10.1001/jamahealthforum.2021.4164
19. Milkman KL, Patel MS, Gandhi L, et al. A megastudy of text-based nudges encouraging patients to get vaccinated at an upcoming doctor's appointment. *PNAS*. 2021;118(20). doi:10.1073/pnas.2101165118
20. Patel MS, Volpp KG, Asch DA. Nudge Units to Improve the Delivery of Health Care. *N Engl J Med*. 2018;378(3):214-216. doi:10.1056/NEJMp1712984
21. Thaler RH, Sunstein CR. *Nudge: The Final Edition*. Revised edition. Penguin Books; 2021.
22. Henderson JT, Vesco KK, Senger CA, Thomas RG, Redmond N. Aspirin Use to Prevent Preeclampsia and Related Morbidity and Mortality: Updated Evidence Report and Systematic Review for the US Preventive Services Task Force. *JAMA*. 2021;326(12):1192. doi:10.1001/jama.2021.8551

Bridging the Gap from Postpartum to Primary Care

Endline Survey

Study PIs: Mark Clapp, MD MPH (MGH); Jessica Cohen, PhD (Harvard)

Site PI: Mark Clapp, MD MPH (MGH)

Section 0: Survey Text Introduction

This survey is being administered as part of the MGH Bridge Study, which you agreed to participate in during or shortly after your pregnancy.

The survey will take approximately 10-20 minutes to complete. Participants who finish the survey will receive a $25 Amazon gift card.

Your responses will not be shared with anyone outside of the research team, including your doctors or other care team members. They will not be part of your medical record or affect your ability to receive care or services at MGH.

Thank you for your help and contribution to this study. Please feel free to contact me with questions or concerns about this study.

Mark Clapp, MD MPH
Maternal-Fetal Medicine
Massachusetts General Hospital
bridgestudy@mgh.harvard.edu
617-724-4531

By clicking PROCEED, I AGREE TO PARTICIPATE IN THIS SURVEY.

Section 1: Health Insurance

101. Do you currently have health insurance? [Select ONE.]

1. Yes

2. No

88. Don't know/not sure

99. Prefer not to say

102. What kind of health insurance do you have now? [Check ALL that apply.]

1. Health insurance from my job, school, or the job of my spouse or partner
2. Health insurance from my parents
3. Health insurance from the state Marketplace, Massachusetts Health Connector, or HealthCare.gov
4. Medicaid or MassHealth
5. Other government plan or program such as SCHIP/CHIP
6. Other government plan or program not listed above such as MCH program, indigent program or family planning program
7. TRICARE or other military/veteran health care
8. Other health insurance, please specify: ________________________

88. Don't know/not sure
99. Prefer not to say

Section 2: Health

201. In general, how would you describe your current PHYSICAL health? [Select ONE.]

1. Excellent

2. Very good

3. Good

4. Fair

5. Poor

88. Don't know/not sure

99. Prefer not to answer

202. In general, how would you describe your current PHYSICAL health? [Select ONE.]

1. Excellent

2. Very good

3. Good

4. Fair

5. Poor

88. Don't know/not sure

99. Prefer not to answer

203. Have you ever been told by a doctor, nurse, or other health professional that you have any of the following conditions? [Select an answer for EACH option.]

	YES	NO	Don't know/ Not sure	Prefer not to say
1. High blood pressure or hypertension				
2. Diabetes or high blood sugar				
3. Anxiety or depression				
4. Overweight or obesity				

204. Since giving birth, have you received any of the following treatments or support for your **emotional** or **mental health**? [Select an answer for EACH option.]

	YES	NO	Don't know/ Not sure	Prefer not to say
1. Counseling or therapy				
2. Medication (e.g., antidepressants or antianxiety medications)				
3. Treatment at a hospital or emergency department				
4. Support group or care from an in-home visiting health professional (e.g., nurse, midwife, doula)				
5. Support from a social worker				

205. Are you currently using any form of birth control to prevent pregnancy? [Select ONE.]

1. Yes

2. No → Go to Next Section

88. Don't know/not sure

99. Prefer not to answer

206. What kind of birth control are you or your spouse/partner currently using? [Select an answer for EACH option.]

	YES	NO	Don't know/ Not sure	Prefer not to say
1. Have had a hysterectomy or tubes tied/blocked				
2. Partner has vasectomy				
3. IUD (Mirena, ParaGuard, Liletta, Kyleena, etc.)				
4. Contraceptive implant in the arm (Norplant, Implanon, Nexplanon, etc.)				
5. Birth control pills, shots/injections, patch or vaginal ring				
6. Condoms				
7. Withdrawal (pulling out) or calendar rhythm/cycle-based method				
8. Not having sex (abstinence)				
9. I cannot get pregnant due to the sex of my partner				
10. Other method, please specify:				

Section 3: Primary Health Care

301. Do you currently have a primary care provider? This is a person who you would see for an "annual visit" or "physical" or for care when you are feeling ill. *This does NOT refer to an urgent care or emergency department*. [Select ONE.]

1. Yes →(Go Question to 303)
2. No

3. 88. Don't know/not sure

4. 99. Prefer not to say

302. Please indicate ALL of the reasons why you <u>do not currently have a primary care provider</u>.
[Select an answer for EACH option.]

	YES	NO	Don't know/ Not sure	Prefer not to say
1. I don't feel I need one				
2. I don't know how to find one				
3. I want to find one but never have time				
4. I am worried about the cost				
5. I can't find one that is accepting new patients or has appointments available				
6. I can't find one that accepts my insurance				
7. I can't find one that speaks my preferred language				
8. I can't find one I can trust				
9. Other reason, please specify:				

303. <u>Currently</u>, if you needed to see your <u>primary care provider</u> to help you <u>if you are sick</u> or <u>for an annual physical exam</u>, how difficult would it be for you to do the following?
[Select an answer for EACH option.]

	**Very Difficu lt**	**Somewhat Difficult**	**Not Difficult at All**	Don't know/ Not sure	Prefer not to say
**1.** Get an appointment soon enough					
**2**. Find <u>transportation</u> to the appointment					
**3.** Afford the cost of the visit					
**4.** Get time off work or school to get to the appointment					
**5**. Get childcare in order to go to the appointment					

304. Since giving birth, how many times have you had an appointment with a primary care provider for a routine checkup, “annual” or “physical exam”, or sick care (either an in-person visit or a telemedicine visit by phone/video)? *This does not include visits to your **pregnancy care provider (OB/GYN or midwife)**, an emergency department, or urgent care center.* [Select ONE]

1. No appointments with a primary care provider
2. 1 appointment → (Go to Question 307)
3. 2 or more appointments → (Go to Question 307)

88. Don’t know/not sure

99. Prefer not to answer

305. Please indicate ALL of the reasons you have not had an appointment with a primary care provider since delivery. [Select an answer for EACH option.]

	YES	NO	Don’t know/ Not sure	Prefer not to say
1. I have already seen a primary care provider within the year before my delivery				
2. I have an appointment scheduled in the future				
3. I had no need or reason to see them				
4. I was concerned about the cost				
5. I don’t know who to make an appointment with				
6. I couldn’t get an appointment quickly enough				
7. I couldn’t get an appointment at a convenient time				
8. I was unable to miss work				
9. I couldn’t get childcare so I could go to the appointment				
10. I didn’t have transportation to the appointment				
11. I didn’t have the energy or feel well enough.				
12. I don’t have a primary care doctor				
13. Other reason, please specify:				

306. When was the last time you saw a primary care provider for any reason? You can use

either months or years in your answer. Your best estimate is fine.

______ months ago

______ years ago

88. Don't know/not sure

99. Prefer not to answer

**************************************SKIP TO NEXT SECTION***

307. How long after giving birth did you see a primary care provider for any reason (either in-person or virtual)? [Select ONE.]

1. Within the first month after delivery
2. 1 – 2 months after delivery
3. 3 – 4 months after delivery
4. 5 – 6 months after delivery

88. Don't know/not sure

99. Prefer not to answer

308. Since giving birth, what were the reasons for your visit(s) with the primary care provider? *This does not include visits to your* ***pregnancy-care provider (OB/GYN or midwife)*** *OR to an emergency department or urgent care center* [Select an answer for EACH option.]

	YES	NO	Don't know/ Not sure	Prefer not to say
1. Regular checkup (e.g., annual visit)				
2. Sick care (e.g., for an illness, injury, infection, etc.)				
3. To talk about a specific medical problem (examples: high blood pressure or diabetes)				
4. To discuss a mental health concern				
5. To discuss and/or get birth control				
6. To get a prescription				
7. To get a vaccine				
8. Other reason, please specify:				

309. Since giving birth, in any of your primary care visits or phone calls, did the doctor or nurse ever do any of the following? [Select an answer for EACH option.]

	YES	NO	Don't know/ Not sure	Prefer not to say
1. Asked me questions about my mental health and wellbeing				
2. Prescribed me medication or referred me to a therapist/psychiatrist for my mental health				
3. Checked my weight and height				
4. Talked to me about ways to manage my weight				
5. Asked me questions or talked to me about my blood sugar or diabetes				
6. Perform a blood test for diabetes				
7. Checked my blood pressure				
8. Talked to me about ways to manage my blood pressure				

Section 4: Urgent or Emergency Care

401. Since giving birth, have you been to an emergency department or urgent care center to get care for yourself? [Select ONE]

1. Yes
2. No → (Go to Question 403)

88. Don't know/not sure → (Go to Question 403)

99. Prefer not to answer → (Go to Question 403)

402. Since giving birth, which emergency department(s) or urgent care center(s) did you go to get care for yourself?
List the names of all hospital emergency rooms or urgent care centers you visited.

Name 1: ______________________________

Name 2: ______________________________

Name 3: ______________________________

Name 4: ______________________________

88. Don't know/not sure

99. Prefer not to answer

403. The LAST TIME you went to the emergency department or urgent care center for yourself, what was the reason you went there? [Select an answer for EACH option.]

	YES	NO	Don't know/ Not sure	Prefer not to say
1. I needed emergency care				
2. My doctor instructed me to go to an urgent care or emergency department				
3. My doctors' offices were closed				
4. I couldn't get an appointment to my doctor soon enough				
5. I didn't have a doctor or primary care provider				
6. I couldn't afford the cost to see a doctor				
7. I needed a prescription drug				
8. I didn't know where else to go				
9. Some other reason, please specify:				

Section 5: Postpartum Obstetric Care

501. Since giving birth, has your OBGYN or midwife discussed any of the following with you in your appointments (either in person or by phone or video)? [Select an answer for EACH option.]

	YES	NO	Don't know/ Not sure	Prefer not to say
1. My mental health and wellbeing				
2. How to manage my blood pressure or hypertension				
3. How to manage my weight				
4. How to manage my blood sugar or diabetes				
5. My plan for birth control or future pregnancies				
6. A recommendation to see a primary care doctor				
7. A recommendation to see or talk with a social worker				
8. Any follow up with a social worker				

Section 6: Postpartum Leave, Work and Childcare

601. How many children (under age 18) live in your household? Only include children who live with you at least 50% of the time.

_____ children

88. Don't know/not sure

99. Prefer not to answer

602. How many adults (over age 18), including yourself, live in your household? Only include adults who live with you at least 50% of the time.

_____ adults

88. Don't know/not sure

99. Prefer not to answer

603. Since giving birth, did anyone in your household receive assistance from any of the following sources? [Select an answer for EACH option.]

	YES	NO	Don't know/ Not sure	Prefer not to say
1. Unemployment compensation or worker's compensation				
2. Cash assistance from a state or county welfare program				
3. The Supplemental Nutrition Assistance Program (SNAP)				
4. Woman, Infants and Children Nutrition Program (WIC)				
5. Help paying for childcare from a government agency				
6. A federal, state or local government housing program that lowers your rent or provides a housing voucher				
7. Income from SSI (Supplemental Security Income), a federal program to help older adults, blind, and disabled people				
8. Other government program(s) not listed here, please specify:				

604. What is your yearly total household income now, before taxes? Include income from all family members living in your household (your income, your spouse's or partner's income, and any other income you may have received). **All information will be kept private and will not affect any services you are now getting.** [Select ONE]

1. $ 0 - $ 9,999
2. $10,000-$19,999
3. $ 20,000 – $ 29,999
4. $30,000 – $ 39,999
5. $40,000 - $59,999
6. $60,000 - $79,999
7. $80,000 - $99,999
8. $100,000 - $119,999
9. $120,000 - $149,999
10. $150,000 - $174,999
11. $175,000 - $199,999
12. $200,000 or above

88. Don't know/not sure

99. Prefer not to answer

605. Since delivery, how many weeks of parental leave/time-off did your spouse, partner, or the baby's father take from work?

__________ weeks [0-100 NUMBER]

87. Not Applicable

88. Don't know/not sure

99. Prefer not to answer

606. Are you currently working for pay? [Select ONE.]

1. Yes, I am working part time

2. Yes, I am working full time

3. No. -→ Skip to Question 612

88. Don't know/not sure

99. Prefer not to answer

607. How many weeks after your delivery did you begin working?

__________ weeks [0-100 NUMBER]

88. Don't know/not sure

99. Prefer not to answer

608. Since giving birth, did you receive any paid or unpaid parental leave/time-off from your job? [Select ALL THAT APPLY]

1. Yes, I took paid leave
2. Yes, I took unpaid leave
3. No, I did not take any paid or unpaid leave, skip to NEXT SECTION

88. Don't know/not sure

99. Prefer not to answer

609. Since giving birth, which types of parental leave have you used? [Select ALL THAT APPLY]

1. Paid parental leave provided by my employer
2. Unpaid parental leave provided by my employer
3. Massachusetts Paid Family and Medical Leave (MA PFML)
4. Temporary Disability Insurance or Short-Term Disability Insurance

5. Paid Time Off / Paid Vacation Days (Sometimes called PTO)
6. Sick Leave
7. Unpaid Time Off
8. Other, please specify_________________

88. Don't know/not sure

99. Prefer not to answer

610. Since giving birth, how many weeks of parental leave have you taken in total? *Please answer separately for paid leave and unpaid leave. Your best estimate is fine.*

1. Number of weeks of paid leave _________ [0-100, NUMBER]
2. Number of weeks of unpaid leave _________ [0-100, NUMBER]

88. Don't know/not sure

99. Prefer not to answer

611**.** How well did you understand the maternity leave benefits that were available to you? [Select ONE.]

1. Extremely well
2. Very well
3. Moderately well
4. Somewhat well
5. Not well at all

88. Don't know/not sure

99. Prefer not to answer

**************************SKIP TO NEXT SECTION**********************************

612. Do you plan on either <u>returning to work or starting a new job</u> in the next year? [Select ONE.]

1. I don't plan to return to work or start a new job in the next year
2. Yes, I plan to start working within the next 1 month
3. Yes, I plan to start working within the next 1 – 3 months
4. Yes, I plan to start working More than 3 months from now

88. Don't know/not sure

99. Prefer not to answer

613. Since giving birth, did you receive any paid or unpaid parental leave/time-off from your job? [Select ALL THAT APPLY]

1. Yes, I took paid leave/time-off
2. Yes, I took unpaid leave/time-off
3. No, I did not take any paid or unpaid leave, skip to NEXT SECTION

88. Don’t know/not sure

99. Prefer not to answer

614. Since giving birth, which types of parental leave have you used? [Select ALL THAT APPLY]

1. Paid parental leave provided by my employer

2. Unpaid parental leave provided by my employer

3. Massachusetts Paid Family and Medical Leave (MA PFML)

4. Temporary Disability Insurance or Short-Term Disability Insurance

5. Paid Time Off / Paid Vacation Days (Sometimes called PTO)

6. Sick Leave

7. Unpaid Time Off

8. Other, please specify__________________

88. Don’t know/not sure

99. Prefer not to answer

615. Since giving birth, how many weeks of parental leave have you taken in total? *Please answer separately for paid leave and unpaid leave. Your best estimate is fine.*

1. Number of weeks of paid leave __________ [0-100, NUMBER]
2. Number of weeks of unpaid leave __________ [0-100, NUMBER]

88. Don’t know/not sure

99. Prefer not to answer
616. How well did you understand the maternity leave benefits that were available to you? [Select ONE.]<br><br>1. Extremely well<br>2. Very well<br>3. Moderately well<br>4. Somewhat well<br>5. Not well at all<br><br>88. Don't know/not sure<br><br>99. Prefer not to answer

Section 7: Experiences with Intervention (Treatment Group Only)

701. The study you have participated in aimed to help you to transition to primary care after childbirth. **How important was seeing your primary care provider after birth for you?** [Select ONE.]

1. Extremely important

2. Somewhat important

3. Not very important

4. Not important at all

88. Don't know/not sure

99. Prefer not to say

702. How helpful did you find each aspect of the study? [Select an answer for EACH option.]

	Extremely helpful	Somewhat helpful	Not very helpful	Not helpful at all	Don't know / Not sure	Prefer not to say
1. <u>Information</u> about the role of primary care after your delivery						
2. <u>Scheduling</u> of your primary care appointment for you						
3. <u>Text messaging</u> to remind you about your						

appointment						
4. Reconnecting you to your primary care doctor						

88. Don't know/not sure
99. Prefer not to say

703. In your own words, what assistance would you find or have found helpful in reconnecting with your primary care provider after delivery?

88. Don't know/not sure
99. Prefer not to say

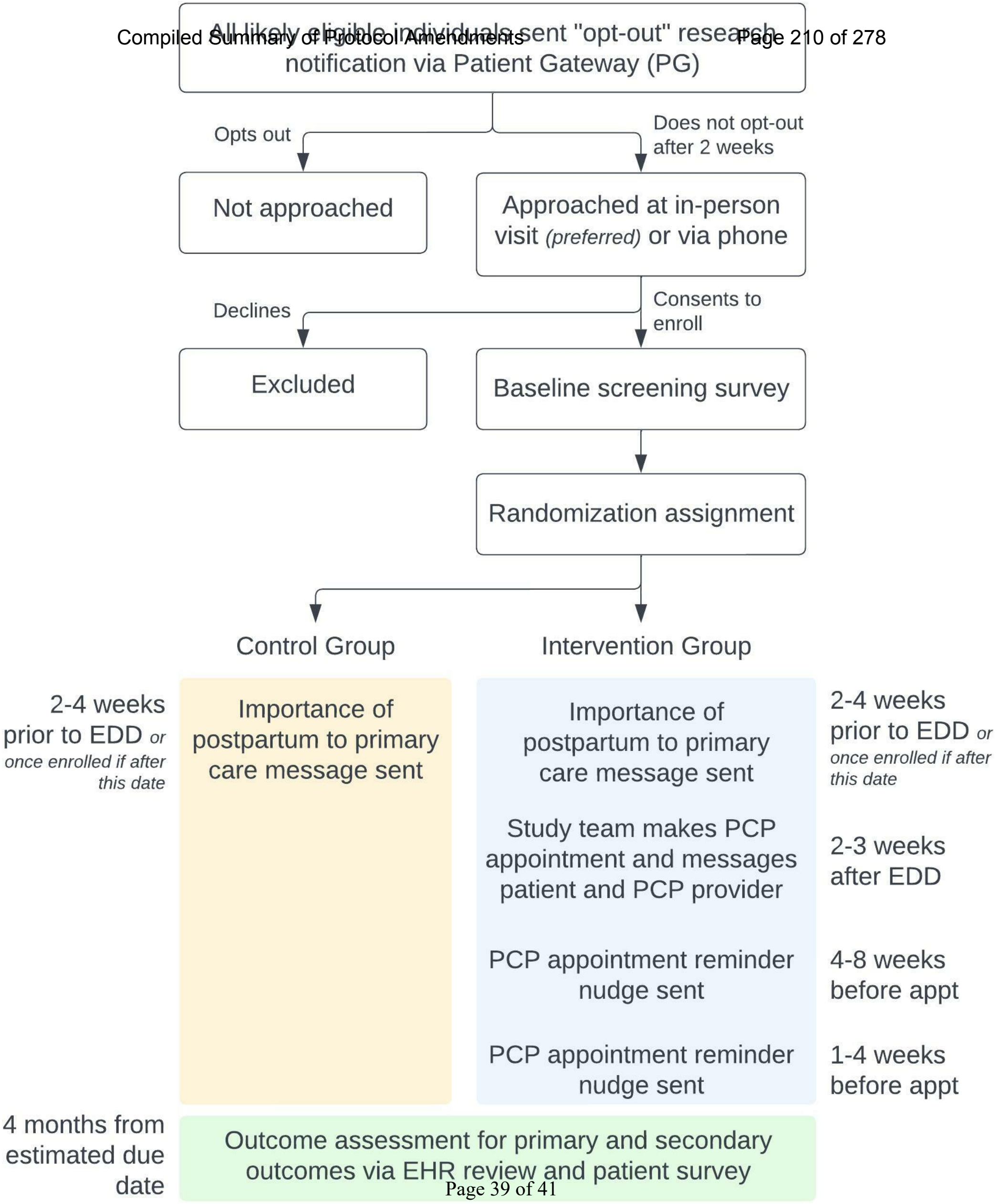
All likely eligible individuals sent "opt-out" research notification via Patient Gateway (PG)
Opts out
Does not opt-out after 2 weeks
Not approached
Approached at in-person visit (preferred) or via phone
Declines
Consents to enroll
Excluded
Baseline screening survey
Randomization assignment
Control Group
Intervention Group
2-4 weeks prior to EDD or once enrolled if after this date
Importance of postpartum to primary care message sent
Importance of postpartum to primary care message sent
2-4 weeks prior to EDD or once enrolled if after this date
Study team makes PCP appointment and messages patient and PCP provider
2-3 weeks after EDD
PCP appointment reminder nudge sent
4-8 weeks before appt
PCP appointment reminder nudge sent
1-4 weeks before appt
4 months from estimated due date
Outcome assessment for primary and secondary outcomes via EHR review and patient survey

Email Text for REDCap Survey

Subject Line: "MGH Obstetrics Bridge Study: Follow-up Survey Link"

Body Text:

"Dear Bridge Study Participant,

Thank you for participating in the Bridge Study in the MGH Department of Obstetrics and Gynecology!

Please complete the following survey about the care you received after your delivery: <<link>>

Participants who complete the survey will receive a $25 Amazon gift card.

Your responses will not be shared with anyone outside of the research team, including your doctors or other care team members.

Thank you for your help and contribution to this important research. Please feel free to contact us with questions or concerns.

MGH Bridge Study
bridgestudy@mgh.harvard.edu
617-643-5483"

SMS Text Message
For individuals who consented to receive SMS messages

"MGH Bridge Study Message: Please check your email to complete a short survey. Respondents will receive a $25 gift card. Thank you!"

Mass General Brigham

Title: Bridging the Gap from Postpartum to Primary Care: A Behavioral Science Informed Intervention to Improve Chronic Disease Management among Postpartum Women (the Bridge Study)

Sponsor Name:

PI Name: Clapp, Mark A **Protocol #:** 2022P001723 **Type:** Amendment (AME12)

Date Received: March 17, 2023

Signatures

PI Name: Clapp, Mark A, MD, MPH

Authenticated: March 17, 2023

Amendment

COVID-19 Amendment

Is this amendment ONLY related to research impacted by COVID-19?

Refer to MGB Policy on Conduct of Human Research Activities during COVID-19 Operations for description of Amendments which do **require prior IRB review and approval.**

- ○ Yes
- ◉ No

Central IRB Performance Sites

Is this a protocol where the Mass General Brigham IRB is serving as the single IRB (sIRB) for external sites/institutions?

- ◉ Yes
- ○ No

Would you like to 'Add' a Site?

- ○ Yes
- ◉ No

Would you like to 'Remove' a Site?

- ○ Yes
- ◉ No

Sponsor Amendment

Is there a sponsor amendment number?

- ○ Yes
- ◉ No

Change in Protocol Status

Is this a cede protocol or project that was determined to be exempt, not human subjects research or not engaged in human subjects research?

- ○ Yes
- ◉ No

Mass General Brigham

Do you need to change the overall status of the protocol? For example, Re-Open to Enrollment or indicate that Research Interventions/Assessments Continue after telling the IRB these have ceased.

- ◯ Yes
- ◉ No

Briefly describe the proposed changes:

After piloting, the endline survey questions were modified slightly. The renumeration was decreased from $25 to $20 after finding the survey could be completed in 5-10 minutes rather than 10-20 minutes, which was originally expected.

Changes to the renumeration amount required an update to patient messages/communication, which are uploaded as attachments.

Provide rationale for the proposed changes:

Improved survey question language

More appropriate renumeration based on the study's ask of the participant

---

Will the proposed change(s) significantly alter the risk to benefit assessment the IRB relied upon to approve the protocol?

- ◯ Yes
- ◉ No

Will the proposed change(s) significantly affect the integrity of the protocol?

- ◯ Yes
- ◉ No

Informed Consent

Do the changes require a revision to the consent form?

- ◯ Yes
- ◉ No

Remuneration

Will subjects be paid or receive any type of remuneration / compensation for their time and expenses?

- ◉ Yes
- ◯ No

Payment for Participation in Research

Mass General Brigham

Indicate the type and total amount of compensation for completion of the study.

- ☐ Cash
- ☐ Check
- ☑ Gift Certificate

Amount e.g., $50, enter 50.00

20

☑ Other

Amount e.g., $200, enter 200.00

20

Explain:

An additional $20 gift card is being provided to individuals who opt to complete the end line survey.

NOTE: Payments to subjects must be made by check if payment involves a one-time payment of greater than $50 OR multiple payments of any amount. See policies Payments to Human Subjects for Participation in Research and Cash Control and Accountability for Payments to Human Subjects for Participation in Research.

Reimbursement for Expenses Related to Participation in Research

If there is not a set amount for meals, parking, and transportation, then estimate or enter the maximum amount budgeted per subject.

- ☐ Meals
- ☐ Parking
- ☐ Transportation
- ☐ Other

Attachments

Name	Mode
Detailed Protocol_031723_v1-6_clean (Detailed Protocol)	Electronic
Bridge Survey Endline _ final (Instrument/Questionnaire)	Electronic
Survey Email Text_031223 (Document for review)	Electronic
Survey Remider SMS Text_031723 (Document for review)	Electronic

Mass General Brigham

Institutional Review Board
Intervention/Interaction Detailed Protocol

Principal Investigator:	Mark Clapp, MD MPH (MGH), Jessica Cohen, PhD (HSPH)
Project Title:	Bridging the Gap from Postpartum to Primary Care: A Behavioral Science Informed Intervention to Improve Chronic Disease Management among Postpartum Women
Version Date:	03/17/2023
Version Name/Number:	v1.6

1. Background and Significance

Burden of Chronic Disease and the Role of Primary Care

Chronic health conditions affect millions of people in the US each year. In 2018, 51.8% of adults had at least 1 chronic condition, and 27.2% had multiple conditions.[1] The prevalence of chronic disease was higher in women compared to men, older adults (87.6% in adults ≥65 years old), and people with public insurance.[1] Many chronic conditions, by their nature, develop over time and have risk factors that can be identified prior to the onset of disease. Strong evidence underpins prevention strategies for many conditions, which are advanced by the US Preventative Services Taskforce.[2] While the long-term health of a patient is the responsibility of an entire health system, primary care providers (PCPs) provide an integral role in preventing, screening for, and managing disease across the lifespan. Studies have shown the health benefits of receiving regular care under a PCP.[3–6]

Despite the known benefits of having an identifiable usual source of care and the value of health care maintenance, the percent of the population with a PCP has been decreasing over time.[7] Consistently, adults who are younger (age 20-40 years) have the lowest rates of primary care use. In 2015, 44% and 36% of 20- and 30-year-olds had no identifiable source of primary care.[7] The proportion without primary care were also higher among racial/ethnic minority populations and among those who had less education, lower incomes, and no known comorbidities.[7] The number of adults and the time elapsed without regular primary care follow-up can be considered missed opportunities to improve a patient's current and long-term health. The disproportionate lack of primary care among certain subgroups of the population, often groups who already have worse health outcomes, only serves to widen the pre-existing disparities.

Pregnancy as a Window to Future Health

In the US, 98.2% of pregnant women receive some form of prenatal care, with the average patient having >10 visits during their pregnancy.[8] During a pregnancy, women are screened for pre-existing and pregnancy-related conditions.[9] In adults ages 18-39, the prevalence of obesity, hypertension, prediabetes/diabetes, and mental illness are estimated at 39%, 7.5%, 28%, 25%, respectively.[10–13] Even for those who have no prior identified comorbidities, the most common

pregnancy-related conditions—pregnancy-related hypertension and gestational diabetes (8% and 10% of pregnancies, respectively)—indicate a predisposition to or confer health risks that persist as women age. For example, over 25% of women with gestational diabetes will develop Type 2 diabetes mellitus, and women with pre-eclampsia have more than a two-fold risk of significant cardiovascular disease later in life.[14,15] For these reasons, pregnancy is often considered a “window” into a woman’s future health and presents a unique opportunity to optimize a woman’s health status early in her life when she otherwise may not have been engaged in care.[16]

Pregnancy as on Opportunity for Engagement with a Long-term Care Provider
Pregnancy is a period when women are highly engaged and active participants in their health care.[17] It has been described as a “golden opportunity” to motivate women towards positive health behaviors, including prevention and management of chronic disease. However, women often fall off a “postpartum cliff” of health system engagement after the early postpartum period.[18] A range of systemic, financial, and behavioral barriers often prevents patients from effectively transitioning to primary care. Postpartum women are often simply told to follow-up with their PCP without much information regarding the importance of this follow up care, without assistance in scheduling an appointment (or identifying a PCP if they don’t have one), and often without a direct transfer of relevant health information or accountability across providers.

Postpartum women are left largely on their own to navigate this transition to primary care and, in particular, to navigate it at a time when they face the high cognitive and physical demands of caring for an infant. At this time of limited cognitive bandwidth, the importance of continuity of care for chronic conditions and active engagement in one’s longer-term health and wellbeing is unlikely to be salient and top-of-mind. These critical moments of unsupported health care transition can exacerbate pre-existing disparities in health and health care, with patients who are the least able to navigate the US health care system most likely to fall through the cracks. Momentum is building in US health and social safety net policy to facilitate healthy transitions from pregnancy to parenthood. For example, federal and state initiatives to expand pregnancy-related Medicaid coverage from 60 days to one year postpartum have been proposed, and access to paid family leave is increasing. However, very little evidence exists on effective and cost-effective approaches to facilitating transitions to primary care and management of chronic diseases in the postpartum year.

2. Specific Aims and Objectives

The objective of the proposed study is to increase patient engagement in primary care after the immediate postpartum period for women with pregnancy-associated conditions that convey a long-term health risk. Specifically, we aim to evaluate the efficacy of an intervention bundle (automatic scheduling of PCP appointment after delivery, salient labeling, and appointment reminder nudges) to increase patient attendance at a primary care provider appointment (within 4 months of delivery for women with or at risk for obesity, diabetes, hypertension, and/or a mental health condition.

Specific Aims:

1) Test the efficacy of an intervention bundle (patient-tailored health information, automatic scheduling of PCP appointment after delivery, and appointment reminder nudges) to increase patient attendance at a primary care provider appointment within 4 months of

delivery for women with obesity, diabetes, hypertension, and/or a mental health condition
2) Test the efficacy of the intervention bundle to improve compliance with the condition-specific, guideline-based health screenings
3) Test the efficacy of the intervention bundle to reduce unscheduled or urgent encounters (e.g., emergency department visits) within the health system

3. General Description of Study Design

We will conduct a randomized controlled trial comparing this intervention bundle to the receipt of generic information on the importance of primary care follow-up after delivery.

Women will be randomized with equal probability into either a treatment or control arm. The intervention combines several features designed to target reasons for low take-up of primary care among postpartum women (see Logic Model). We leverage the potential value of defaults/opt-out, salient labels, and reminders to encourage use of primary care within 4 months of delivery. Women in both the intervention and control arms will receive information via MGH's patient portal toward the end of the pregnancy regarding the importance of transitions to primary care in the postpartum year. This information will be similar to, but reinforcing, the information they would receive from their obstetrician about following up with their primary care physician. In addition to this initial message, women in the treatment arm will receive the following intervention components, developed based on recent evidence regarding behavioral science approaches to activating health behaviors:[19–21]

- Targeted messages about the importance and benefits of primary care.
- Default scheduling into a primary care appointment at approximately 3-4 months after delivery. The patient will be scheduled for a primary care visit with their assigned primary care provider in the Mass General Brigham system. They will be informed of the option to cancel the appointment, change the appointment day/time, or change the care provider either through the patient portal).
- Reminders about the appointment and importance of follow up primary care at 2-4 points during the postpartum period via the patient portal.
- Tailored language in the reminders based on recent evidence from behavioral science about the most effective approaches to increasing take-up. For example, messages will inform the patient that an appointment has been reserved for them at their doctor.

The Logic Framework that underpins the basis for this study is shown below:

Needs/ Problems	Barriers to Primary Care Use	Input/Intervention	Outcomes	Long-term Goals
• Low PCP use among people who have/are at risk of chronic conditions (diabetes, hypertension, obesity, mental illness)<br>• Pregnancy is ideal opportunity to interrupt progression of chronic disease, but this window into long-term health is often missed	• Insufficient patient information<br>• Underestimate risks<br>• Low salience<br>• Cognitive demands in postpartum period<br>• Time constraints and competing priorities<br>• Health system/structural barriers to postpartum care continuity<br>• Poor transitions between obstetricians and PCPs	• Targeted information about importance of primary care<br>• Default scheduling of postpartum primary care appointment<br>• Reminder messages about primary care appointment leveraging salience and pre-commitment<br>• Salient labeling of the transition appointment<br>• PCP messaging about a patient's recent pregnancy and upcoming follow-up	**PRIMARY**<br>• Visit with primary care provider within 4 months of childbirth<br>**SECONDARY**<br>• Condition-specific screening:<br>-documentation of blood pressure<br>-diabetes screening test<br>-measurement of weight<br>• Counseling on risk prevention or reduction strategies at visit<br>• Use of emergency room or urgent care for any reason	• Engagement with PCP<br>• Earlier, more effective chronic disease prevention and management<br>• Interruption of disease progression over life course<br>• Improved lifelong health status

The flow of study activities is shown below:

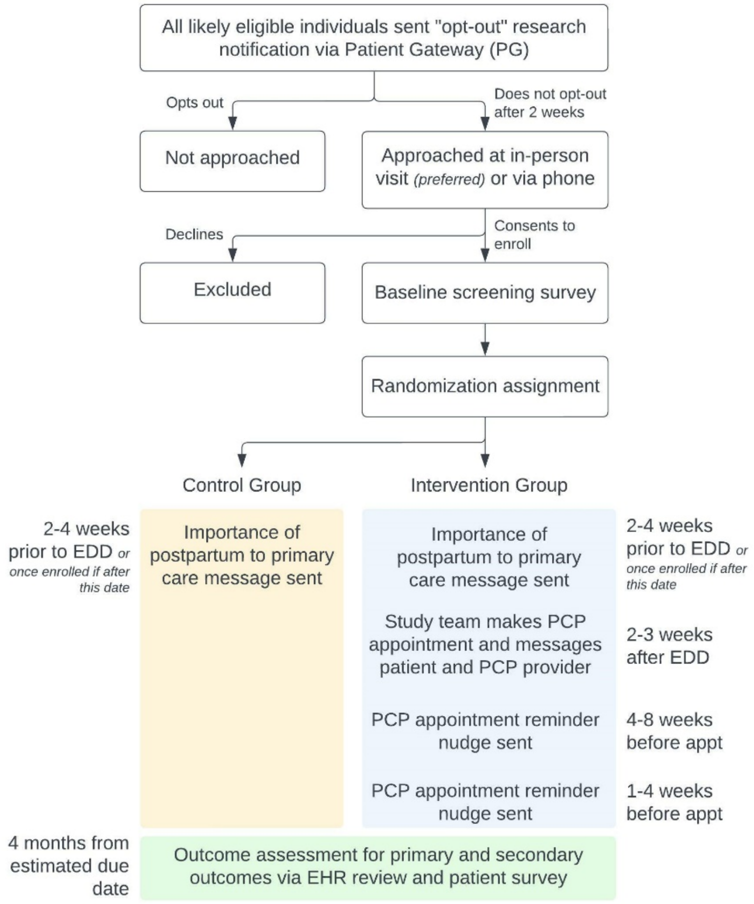

4. Subject Selection

Pregnant patients receiving prenatal care at Massachusetts General Hospital (MGH) will be targeted for recruitment. MGH conducts approximately 3500 deliveries per year, with roughly one-third of patients identifying as non-white and 35% of pregnancies covered by Medicaid.

Approximately 49% of the clinic population may ultimately be eligible to be approached.
The eligibility criteria include:

- Estimated date of delivery and the following 4-month postpartum outcome assessment window completed prior to study end date
- Currently pregnant or within 2 weeks of estimated date of delivery
- Have one or more of the following conditions:
  - Chronic hypertension
  - Hypertensive disorders of pregnancy or risk factors for hypertensive disorders of pregnancy per the USPTF aspirin prescribing guidelines (e.g., history of pre-eclampsia, kidney disease, multiple gestation, autoimmune disease)[22]
  - Type 1 or 2 diabetes

  - Gestational diabetes
  - Obesity (body mass index ≥30 kg/m2)
  - Depression or anxiety disorder
- Have a primary care provider listed in the patient's medical record
- Receive obstetric care at an MGH-affiliated outpatient prenatal clinic
- Has access to and be enrolled in the electronic health record patient portal and consents to be contacted via these modalities
- Able to read/speak English or Spanish language
- Age ≥18 years old
- Not diagnosed with or undergoing evaluation for stillbirth/fetal demise

All women, regardless of race/ethnicity, who meet the eligibility criteria will be included. In 2019, 3,789 women gave birth at the study institution, of whom 57% were white, 7% were black, 12% were Asian, 18% were Hispanic, and 6% declined to report their race/ethnicity. A similar distribution is expected for this study.

The study will distribute materials in English and Spanish languages. This encompasses >95% of patients who deliver at the study institution.

5. Subject Enrollment

This study will rely on recruiting for research through Patient Gateway and follow the IRB guidance and DHeC Research Checklist and training for this process.

In the month prior to the start of enrollment, all potentially eligible individuals (based on the criteria above) will be identified using RPDR and Epic Reporting search queries. This list (the "potentially eligible" list) will be provided to the DHeCare Research Team to build an RSH Record in Epic. This list will be updated monthly to identify newly eligible individuals (e.g., new diagnosis of gestational diabetes or new patient transferring into the practice) during the recruitment months and fed back to the DHeCare team to update "potentially eligible" list.

Once built and each month during recruitment phase, the study's research coordinator (not study investigators) will send the IRB-approved Research Invitation Letter to patients who are eligible, not already enrolled, and have not declined to be sent research notifications through the portal. The Research Letter will employ an opt-out approach, asking individuals who do not wish to be approached in clinic or remotely to respond via PG messaging, email, or phone within 2 weeks of receipt of the letter. Those who have "read" the letter and not opted out after 2 weeks will be moved from the "potentially eligible" list to the "waiting to be approached" list in the study workflow.

During recruitment months, the study coordinator will keep a log of patient's upcoming appointments for those on the "waiting to be approached" list. They will then attempt to approach individuals for enrollment when they present for an in-person encounters (preferred). Patients will ideally be approached between 32-36 weeks of gestation, when feasible; however, priority will be given to patients at the latest gestation. For those Spanish-speaking patients, a hospital-based interpreter will be used when approaching/consenting patients.

The study staff will introduce the study and review the purpose of the study, the nature of the subject's participation, the possible risks and discomforts associated with participation, the potential benefits of participation, a statement of the voluntary nature of participation, and a description of the mechanisms used to ensure confidentiality.

All patients will receive study-related messages through Patient Gateway. In addition, patients will be asked if they would be willing to receive no more than 5 SMS messages to their personal cell phone during the first 4 months of the postpartum period. The study staff will review the specific concerns and risks about receiving unencrypted text messaging communications, as outlined by the MGB IRB.

A waiver of documentation of informed consent is requested, as the study presents no more than minimal risk of harm to subjects and involves no procedures for which written consent is normally required outside of the research context. Verbal consent will be obtained for 1) overall study participation and 2) optional SMS message participation, separately. Verbal consent will be obtained from both in-person and phone recruitment efforts.

A Study Fact Sheet, which summarizes the study details, risks, and benefits, will be provided to all subjects who are approached, either in-person, by mail, or electronically. This Study Fact Sheet also includes information supplied by the MGB IRB specifically related to the concerns and risks of receiving unencrypted SMS/text messages.

For tracking verbal consent, the study staff will keep a detailed log in REDCap documenting:

1) name of study staff performing consent,
2) date of attempted approach,
3) the method of attempted approach (in-person or via phone),
4) use of Spanish interpreter (yes/no),
5) subject agreement to be approached (agree/disagree),
6) attestation to full review of the study procedures/risks/benefits with the subject, as would be done during the process of reviewing a written consent form,
7) attestation to review of supplemental consent to receive unencrypted SMS messages with the subject,
8) subject overall study participation status (enrolled, declined, deferred – agrees to be recontacted, deferred – wishes not to be recontacted),
9) if enrolled, unencrypted SMS text messaging participation (consents, declines),
10) attestation to Study Fact Sheet provided,
11) method by which Study Fact Sheet was provided (in-person, mail, electronic),
12) date in which Study Fact Sheet was provided (in-person, mail, electronic).

6. STUDY PROCEDURES

The RA will keep a detailed log of all patients in the practice, if they are eligible, if they have been approached, and if they consented.

For those that agree to be enrolled, patients will be asked to complete a baseline survey to obtain voluntarily reported information on their demographics, socioeconomic status, health care visit history, and primary care provider. Patients will also be asked to consent to being contacted

by the research team via Patient Gateway messaging. A $20 gift card will be given at the time of enrollment for those that complete the questionnaire and receive the information sheet.

Randomization will occur via a prespecified random allocation sequence within strata. Within each stratum, the PIs will generate a random sequence of treatment-control allocation prior the enrollment of subjects. Then, as patients within strata are enrolled, they will be assigned to the treatment arm associated with that enrollment number.

<u>Control Group</u>
Approximately 2 weeks before a patient's estimated due date (EDD) or as soon as enrolled if this date has passed, the patient will be sent information via the Patient Gateway on the importance of postpartum care and follow-up with their PCP.

<u>Intervention Group</u>

1. Tailored Information:
   Approximately 2-4 weeks prior to the EDD (or later for those who are enrolled beyond 38 weeks gestation or postnatally), the patient will be sent an information via the Patient Gateway on the importance of postpartum care and follow-up with their PCP, which also includes the name and phone number of their primary care provider.
2. Scheduled PCP Appointment:
   Between 2-3 weeks after their delivery, the RA will call the patient's PCP office and make an appointment for them between 3-4 months after delivery based on the scheduling preferences obtained in the initial survey.
3. Targeted Appointment Message with Salient Labeling:
   After the PCP appointment has been made, the patient will be sent a Patient Gateway message saying that a PCP appointment has been reserved for them with the date/time/location information.
   For those consenting to receive SMS messages, an unencrypted text message will also be sent simultaneously.
4. Nudge Reminders:
   Patient Gateway messages will be sent at approximately 4-8 weeks (goal: 4 weeks) and 1-4 week (goal: 1 week) prior to their PCP appointment, reminding a patient of their upcoming appointment.
   For those consenting to receive SMS messages, an unencrypted text message will also be sent simultaneously.
5. Facilitated PCP Communication:
   A study staff member will send the patient's PCP an Epic Inbasket message that the patient 1) is recently postpartum, 2) has or developed health conditions that need long-term management, and 3) has been scheduled (or attempted to be scheduled) for a follow-up visit.

All messages will be made available in English and Spanish.

The text for these Patient Gateway messages is included in the submission. At the end of each patient message, patients will be given the opportunity to stop receiving study-related messages by emailing or calling the study staff. "Opt out" requests will be logged and patients removed from future planned study-related contact.

The text for 3 SMS messages is included in the submission. SMS messages will be sent via Google Voice. We will follow standard recommendations from the MGB Research Information Security for using this system (included under Privacy and Confidentiality section).

<u>Outcome Assessment</u>

1. EHR Review
The study team will review the patient's EHR record for the primary and secondary outcome assessments. Patients will be asked to consent to have records reviewed up to 2 years after the date of their delivery to allow for long-term effects of the intervention on primary care use and health status.

2. Endline Survey
After 4 months from their established due date (EDD), a patient will be eligible to participate the endline survey. The goal timing for the survey completion is 4-6 months after the EDD, but patients will remain eligible for up to one year.

The survey will be administered through REDCap. Study participants will be emailed a unique link to complete the survey. Consent to be contacted for this survey will be obtained at the time of the subject's enrollment in the trial. The patient's email address for contact is verified at the time of study enrollment.

Subjects will be asked to complete a 15-20-minute survey on their care after their delivery. They will be reminded that all questions are optional and that responses will be linked to their study record; however, no responses will be entered into the electronic health record or become part of their permanent medical record. Participants who complete the survey will be offered a $20 gift card for their participation, which they will be informed of prior to starting the survey. There is no penalty for not completing the survey.

If there is no response within approximately 1-2 weeks, the subject will be emailed the link again with a reminder to complete the survey. If there is no response within approximately 2-4 weeks from the initial email OR the patient does not have a working email address, the subject will be called and asked if they would like to complete the survey over the phone. For those that agree, the survey text and questions will be read verbatim by a research assistant not affiliated with the patient's clinical care. Individuals who decline to participate in the survey over the phone or cannot be reached will be marked as non-respondents.

Patients that consented to receive SMS messages as a part of the study will be sent a text message reminder to complete the survey at the same time that emails are sent. No more than 2 SMS message reminders will be sent to each subject.

Deidentified data from this project may be shared outside of MGB with the study funders (J-PAL/NBER/NIA) for data sharing and reproducibility requirements and secondary statistical analysis. All data will be stripped of patient identifiers, per IRB guidance. No data will be shared without a formal Data Use Agreement with MGB.

6. Risks and Discomforts

There are minimal risks to participants.

Patients will be reassured that nonparticipation will not affect clinical care. Patients will also be informed that the researchers with whom they will interact (e.g., during consent, face-to-face, during telephone interviews) are not health care providers. Participants will be consistently reminded that responses to any queries deemed sensitive or uncomfortable (e.g., country of origin/immigration status, income, previous history of abortion) should be considered optional, and they may decline to answer any question(s) and can refuse to continue the study at any point. Participants will be reassured that neither their opportunities for continued health care nor their relationships with health care providers will be jeopardized by study participation.

Health information collected as part of this study will be stored in REDCap. No identifiable data will be stored or downloaded on any personal or unauthorized computers. Study staff will access the data on institutionally purchased and managed computers that operate behind the health system's security and firewall protections. Only the MGH study staff will have access to the identifiable data set. Once the study is completed, data will be deidentified such that it can be analyzed without risk of a breach of privacy or confidentiality. Any data that is shared outside of MGH will require a data use authorization.

Although we will make every effort to protect participant privacy and confidentiality, it is possible that participants' involvement in the study could become known to others. For those in the intervention group, we will be sending personalized messages via the patient portal; for those providing additional consent, we will be sending unencrypted SMS messages that will not contain any personal protected health information. There is the rare possibility that study participation or health information could become known to others despite the use of firewalls, password protection, and other security measures. Standard procedures, as outlined by the MGB Research Information Security Office, will be followed to reduce this risk.

Individuals in the intervention group will be scheduled for an appointment with a primary care doctor. There is the possibility that this visit may result in a charge/cost for the patient. Under the Affordable Care Act, commercial health plans are required to cover an annual Preventive Health Exam at no cost to the patient (no co-payment, co-insurance or deductible). MassHealth also covers these visits without cost-sharing. However, if this visit turns or scheduled as into a "sick" or "disease management" visit, the patient may be billed for some or all aspects of the services provided, depending on their insurer. We will provide directions for patients on how to to contact their insurers or PCP's office prior to the visit to inquire about potential cost-sharing and/or deductibles. Patients will also be advised that some clinics may penalize individuals for not showing to a scheduled appointment ("no show" fee) and be given opportunities to request the appointment be canceled or rescheduled with each appointment reminder.

7. Benefits

Participants in the control group will receive information on the importance of postpartum care and transitioning to primary care after their delivery.

Participants in the intervention group will receive a bundle of interventions designed to increase attendance at primary care visits and facilitate the transition of care after their delivery.

The goal and potential benefits to the subjects in this trial is to increase patient engagement and connection with their primary care provider, receive recommended health screenings and directed counseling, and reduce unscheduled or urgent visits in the postpartum period.

8. Statistical Analysis

Statistical Methods
Analyses will be performed according to the intention-to-treat principle.

Standard independent, two-sided, two-group comparison testing will be used to compare baseline characteristics between the two groups (chi squared tests, t tests, Wilcoxon rank sum tests, when appropriate).

The primary outcome will be attendance rates at PCP visit within 4 months after estimated date of delivery (captured at time of enrollment), which will be compared between the groups using chi squared tests. Relative risks and 95% confidence intervals will be reported.

Secondary outcomes will include measures of long-term health and health care use after the postpartum period.

Subgroup analyses will be performed by a variety of patient characteristics including gestational age at enrollment, prenatal risk factors, morbidity types, patient race-ethnicity, payer, and enrollment location.

P-value of less than 0.05 will be considered to indicate statistical significance.

Power Calculation
Most study calculations were estimated from the MGH 2020 delivery population. The rates of PCP follow-up were estimated from a randomly selected cohort of 50 patients who met the inclusion criteria. We plan to recruit patients into the study for a period of 4 months and expect 1200 unique patients to be at the targeted gestational age during this period. Among these, we expect 86% to already have an assigned PCP in the network, leaving roughly 1,032 patients. Among these, we estimate that 49% have at least one of the targeted health conditions, leaving a target study population of 506 over the 4-month period. Based on previous studies conducted at MGH, we expect an 70% willingness to participate in the research, leaving an expected recruited population of 354 individuals. Based on our record extraction, we estimate that 15% of the targeted study population has a primary care visit within 4 months of delivery. Assuming an alpha of 0.05 and a baseline mean of 15%, with this expected sample size and power of 80%, our study has a minimum detectable effect size of roughly 13 percentage points (from 15% to 28%). A previous study found that default scheduling into postpartum care appointments (with an OBGYN, not a PCP) increased postpartum care take-up by 24 percentage points; since our intervention incorporates defaults and other activating interventions, an MDE of 13 percentage points is reasonable.

9. Monitoring and Quality Assurance

This is a minimal risk study in which the intervention involves default scheduling of appointments and patient messaging. Adverse events are not expected, and there is no physiologic plausibility for this intervention to cause any NIH-defined serious adverse events (e.g., death, prolonged hospitalization, significant disability).

No interim analyses are planned.

Adverse events will be defined and classified in accordance with NIH guidelines:

> Definition of Adverse Events (AE)**:** Any untoward or unfavorable medical occurrence in a human subject, including any abnormal sign, symptom, or disease, temporally associated with the subject's participation in the research, whether considered related to the subject's participation in the research or not.
>
> Definition of Serious Adverse Events (SAE): Any AE that (1) results in death, (2) is life-threatening, (3) results in inpatient hospitalization or prolongation of existing hospitalization, (4) results in persistent or significant disability/incapacity, (5) results in a congenital anomaly/birth defect, and/or (6) may jeopardize the subject's health and may require medical or surgical intervention to prevent one of the other five outcomes listed here.

In the unlikely event an AE or SAE occurs, it will be brought to the PI's attention, and the PI will classify the AE/SAE by severity, expectedness, and relatedness,as listed above. All events that are both serious and unexpected will be reported to Mass General Brigham's IRB, the NIA PO, and to the NIA Roybal DSMB within 48 hours of the research team's knowledge of the SAE. The summary of all other SAEs will be reported to the NIA and to the DSMB quarterly unless otherwise specified by the DSMB. Any unanticipated problem, defined as an issue related to the research suggesting the research places participants or others at greater risk than expected, will be reported to the IRB, the NIA PO, and to the Roybal DSMB within 48 hours of discovery. If the problem involves death then reporting will occur within 24 hours, and this report will include a plan to correct the problem and prevent its occurrence. Any breach of PHI will be reported to the PI, who will report to the IRB and NIA PO within 24 hours of discovery.

The Roybal DSMB oversight is provided by the Standing Roybal DSMB, which includes the members listed: Andrea B. Troxel, ScD (chair); Abby King, PhD; Jerry Gurwitz, MD;Hae-Ra Han, PhD, RN, FAAN; Hang Lee, PhD; Ezra Golberstein, PhD; David Kim, MD PhD; Christopher Celano, MD.

DSMB members will have no direct involvement with the study or conflict of interest with the investigators or institutions conducting the study. Each member has signed a COI statement which includes current affiliations, if any, with pharmaceutical or biotechnology companies (e.g., stockholder, consultant), and any other relationship that could be perceived as a conflict of interest related to the study and/or associatedwith commercial interests pertinent to study objectives.

Data presented to the DSMB will be deidentified as to protect individual participants' privacy and health information. Should the identity of a deidentified subject need to be revealed, the DSMB request will be reviewed and ultimately at the discretion of the Mass General Brigham IRB.

10. Privacy and Confidentiality

☒ Study procedures will be conducted in a private setting
☒ Only data and/or specimens necessary for the conduct of the study will be collected
☒ Data collected (paper and/or electronic) will be maintained in a secure location with appropriate protections such as password protection, encryption, physical security measures (locked files/areas)
☐ Specimens collected will be maintained in a secure location with appropriate protections (e.g. locked storage spaces, laboratory areas)
☒ Data and specimens will only be shared with individuals who are members of the IRB-approved research team or approved for sharing as described in this IRB protocol
☒ Data and/or specimens requiring transportation from one location or electronic space to another will be transported only in a secure manner (e.g. encrypted files, password protection, using chain-of-custody procedures, etc.)
☒ All electronic communication with participants will comply with Mass General Brigham secure communication policies
☒ Identifiers will be coded or removed as soon as feasible and access to files linking identifiers with coded data or specimens will be limited to the minimal necessary members of the research team required to conduct the research
☒ All staff are trained on and will follow the Mass General Brigham policies and procedures for maintaining appropriate confidentiality of research data and specimens
☒ The PI will ensure that all staff implement and follow any Research Information Service Office (RISO) requirements for this research
☒ Additional privacy and/or confidentiality protections

The following procedures (as provided by MGB RISO) will be used for sending SMS messages via Google Voice:

- Google Account:
    - A separate account should be created just for the purpose of the effort/study (in other words, personal Gmail accounts should not be used)
    - For the Google Voice portal, make sure you are not using the same password as your MGB account and ensure the password is strong. (Minimum of 8 characters, alphanumeric, uppercase, lowercase, special character).
        - Two-factor authentication must be enabled
    - Google account will not be shared
    - The Google account used for Google Voice should not be used for emailing or using any other Google Service (i.e., YouTube, Calendar, Contacts, etc.)
    - No credit cards should be added to the Google Account
    - The Google account must be deleted at the end of the study / project
- Participants will be informed not to send personal or health related information via text
- Siri will not be integrated with Google Voice

- Text messages will not address participants by their first name
- Text messages will be sent through the McLean email within the Google account that is created
- Only phone numbers and a unique subject ID will be stored in Google Voice
  - Log records should be deleted manually after 30 days
- No PHI or sensitive information will be communicated via text message
  - Content will not include anything where a healthcare condition or diagnosis can be inferred
- Text message history should be deleted from Google Voice account when no longer needed (within 30 days)
- Study staff will track opt-out requests and delete phone numbers from Google Voice as necessary
- Study staff must not communicate with participants via a group text message
- Access to the portal and overall research must be done from systems that meet MRB RISO compliance requirements; encryption, MobileIron (if Smartphone/Tablet), up to date malware protection, Crowdstrike.
  - https://rc.partners.org/security/secure-your-computer
- Participants:
  - Participants will be texted only if they consent
  - Participants should be informed to delete text messages when no longer needed and hide text push notifications

12. References

1. Boersma P. Prevalence of Multiple Chronic Conditions Among US Adults, 2018. *Prev Chronic Dis*. 2020;17. doi:10.5888/pcd17.200130
2. Home page | United States Preventive Services Taskforce. Accessed December 15, 2021. https://www.uspreventiveservicestaskforce.org/uspstf/
3. Blewett LA, Johnson PJ, Lee B, Scal PB. When a usual source of care and usual provider matter: adult prevention and screening services. *J Gen Intern Med*. 2008;23(9):1354-1360. doi:10.1007/s11606-008-0659-0
4. O'Malley AS, Mandelblatt J, Gold K, Cagney KA, Kerner J. Continuity of care and the use of breast and cervical cancer screening services in a multiethnic community. *Arch Intern Med*. 1997;157(13):1462-1470.
5. DeVoe JE, Saultz JW, Krois L, Tillotson CJ. A medical home versus temporary housing: the importance of a stable usual source of care. *Pediatrics*. 2009;124(5):1363-1371. doi:10.1542/peds.2008-3141
6. Atlas SJ, Grant RW, Ferris TG, Chang Y, Barry MJ. Patient-physician connectedness and quality of primary care. *Ann Intern Med*. 2009;150(5):325-335. doi:10.7326/0003-4819-150-5-200903030-00008
7. Levine DM, Linder JA, Landon BE. Characteristics of Americans With Primary Care and Changes Over Time, 2002-2015. *JAMA Internal Medicine*. 2020;180(3):463-466. doi:10.1001/jamainternmed.2019.6282
8. NVSS - Birth Data. Published June 14, 2021. Accessed September 1, 2021. https://www.cdc.gov/nchs/nvss/births.htm
9. Prenatal care and tests | Office on Women's Health. Accessed December 15, 2021. https://www.womenshealth.gov/pregnancy/youre-pregnant-now-what/prenatal-care-and-tests
10. Centers for Disease Control and Prevention (CDC). Adult Obesity Facts. Published August 29, 2017. Accessed February 14, 2018. https://www.cdc.gov/obesity/data/adult.html
11. Mental Illness. National Institute of Mental Health (NIMH). Accessed December 17, 2021. https://www.nimh.nih.gov/health/statistics/mental-illness
12. Products - Data Briefs - Number 289 - October 2017. Published June 6, 2019. Accessed December 17, 2021. https://www.cdc.gov/nchs/products/databriefs/db289.htm
13. National Diabetes Statistics Report 2020. Estimates of diabetes and its burden in the United States. Published online 2020:32.
14. Sattar N, Greer IA. Pregnancy complications and maternal cardiovascular risk: opportunities for intervention and screening? *BMJ*. 2002;325(7356):157-160. doi:10.1136/bmj.325.7356.157
15. Kim C, Tabaei BP, Burke R, et al. Missed Opportunities for Type 2 Diabetes Mellitus Screening Among Women With a History of Gestational Diabetes Mellitus. *Am J Public Health*. 2006;96(9):1643-1648. doi:10.2105/AJPH.2005.065722
16. Media BKK. Publications & Guidelines | SMFM.org - The Society for Maternal-Fetal Medicine. Accessed October 18, 2021. https://www.smfm.org/publications/225-smfm-statement-implementation-of-the-use-of-antenatal-corticosteroids-in-the-late-preterm-birth-period-in-women-at-risk-for-preterm-delivery
17. Yee LM, Simon MA, Grobman WA, Rajan PV. Pregnancy as a "golden opportunity" for patient activation and engagement. *American Journal of Obstetrics & Gynecology*. 2021;224(1):116-118. doi:10.1016/j.ajog.2020.09.024

18. Cohen JL, Daw JR. Postpartum Cliffs—Missed Opportunities to Promote Maternal Health in the United States. *JAMA Health Forum*. 2021;2(12):e214164. doi:10.1001/jamahealthforum.2021.4164
19. Milkman KL, Patel MS, Gandhi L, et al. A megastudy of text-based nudges encouraging patients to get vaccinated at an upcoming doctor's appointment. *PNAS*. 2021;118(20). doi:10.1073/pnas.2101165118
20. Patel MS, Volpp KG, Asch DA. Nudge Units to Improve the Delivery of Health Care. *N Engl J Med*. 2018;378(3):214-216. doi:10.1056/NEJMp1712984
21. Thaler RH, Sunstein CR. *Nudge: The Final Edition*. Revised edition. Penguin Books; 2021.
22. Henderson JT, Vesco KK, Senger CA, Thomas RG, Redmond N. Aspirin Use to Prevent Preeclampsia and Related Morbidity and Mortality: Updated Evidence Report and Systematic Review for the US Preventive Services Task Force. *JAMA*. 2021;326(12):1192. doi:10.1001/jama.2021.8551

Survey Form Logic

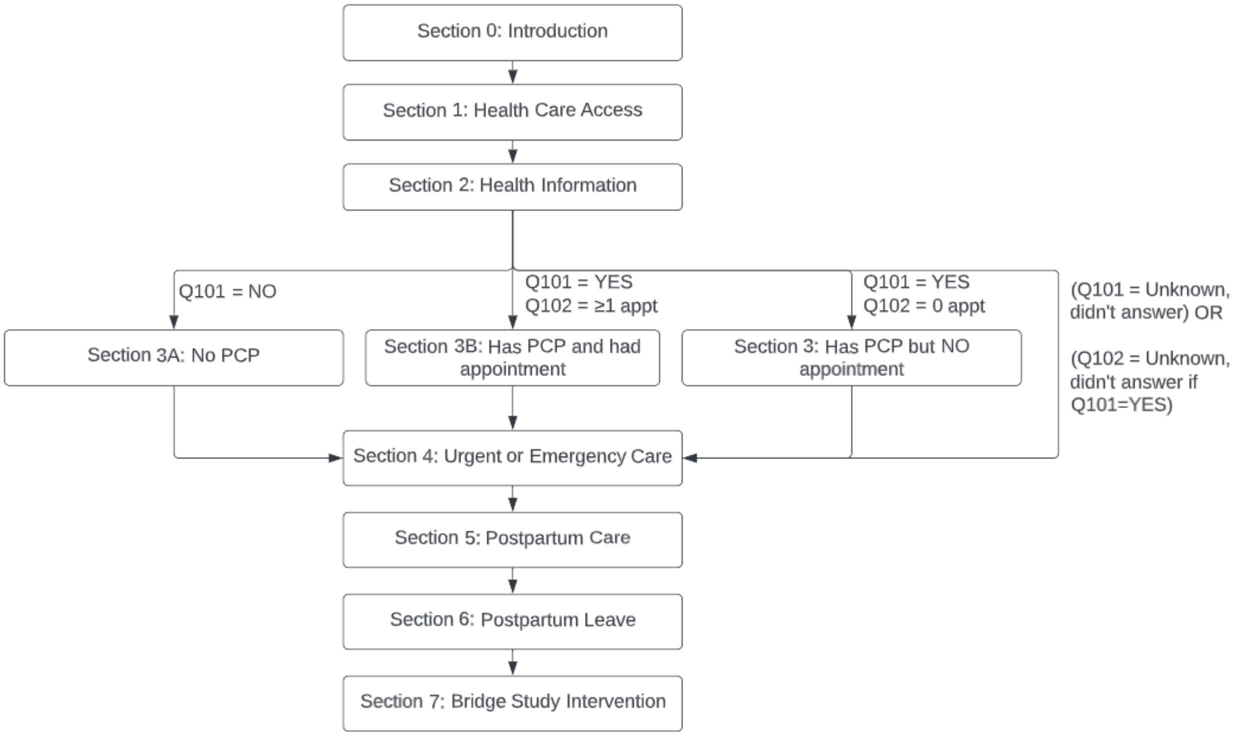

Bridging the Gap from Postpartum to Primary Care

Endline Survey

Study PIs: Mark Clapp, MD MPH (MGH); Jessica Cohen, PhD (Harvard)
Site PI: Mark Clapp, MD MPH (MGH)

Section 0: Survey Text Introduction
This survey is being administered as part of the MGH Bridge Study, which you agreed to participate in during or shortly after your pregnancy.

The survey will take approximately 5-10 minutes to complete. Participants who finish the survey will receive a $20 Amazon gift card.

Your responses will not be shared with anyone outside of the research team, including your doctors or other care team members. They will not be part of your medical record or affect your ability to receive care or services at MGH.

Thank you for your help and contribution to this study. Please feel free to contact me with questions or concerns about this study.

Mark Clapp, MD MPH
Maternal-Fetal Medicine
Massachusetts General Hospital
bridgestudy@mgh.harvard.edu
617-724-4531

By clicking PROCEED, I AGREE TO PARTICIPATE IN THIS SURVEY.

Section 1: Health Care Access

100. Did your pregnancy care provider (OB/GYN or midwife) encourage you to see your primary care provider after giving birth?

1. Yes
2. No
88. Don't know/not sure
99. Prefer not to say

101. Do you currently have a primary care provider? This is a person who you would see for an "annual visit" or "physical" or for care when you are feeling ill. *This does NOT refer to an urgent care or emergency department*. [Select ONE.]

1. Yes
2. No
88. Don't know/not sure
99. Prefer not to say

102. When was the last time you saw a primary care provider for any reason? *This does not include visits to your **pregnancy care provider (OB/GYN or midwife)**, an emergency department, or urgent care center*. You can use either months or years in your answer. Your best estimate is fine. *REQUIRED

1, More than 5 months after giving birth
2, 3-4 months after giving birth
3, 0-2 months after giving birth
4, During pregnancy
5, 0-1 years before my last pregnancy
6, 2-3 years before my last pregnancy
7, More than 3 years before my last pregnancy
88, Don't know/not sure
99, Prefer not to say

103. Currently, if you needed to see a primary care provider to help you if you are sick or for an annual physical exam, how hard would it be for you to do the following?
[Select an answer for EACH option.]

	**Very Hard**	**Somewhat Hard**	**Not Hard at All**	Don't know/ Not sure	Prefer not to say
**1.** Get an appointment soon enough					
**2**. Find transportation to the appointment					
**3.** Afford the cost of the visit					
**4.** Get time off work or school to get to the appointment					
**5**. Get childcare in order to go to the appointment					
**6.** Find a provider with availability					

104. Since giving birth, did you have an appointment with your pregnancy doctor or midwife? This often is called a postpartum visit.<br><br>1, Yes, in-person visits only<br>2, Yes, telemedicine / phone visits only<br>3, Yes, both in-person and telemedicine/phone visits<br>4, No<br>5, Don't know/not sure<br>6, Prefer not to say
105. Since giving birth, have you been to an emergency department or urgent care center to get care for yourself?<br><br>1, Yes<br>2, No<br>88, Don't know/not sure<br>99, Prefer not to say
106. Do you <u>currently</u> have health insurance? [Select ONE.]<br><br>1. Yes<br>2. No<br>88. Don’t know/not sure<br>99. Prefer not to say
107. What kind of health insurance do you have now? [Check ALL that apply.] (If 106=1)<br><br>1. Health insurance from my job, school, or the job of my spouse or partner<br>2. Health insurance from my parents<br>3. Health insurance from the state Marketplace, Massachusetts Health Connector, or HealthCare.gov<br>4. Medicaid or MassHealth<br>5. Other government plan or program such as SCHIP/CHIP<br>6. Other government plan or program not listed above such as MCH program, indigent program or family planning program<br>7. TRICARE or other military/veteran health care<br>8. Other health insurance, please specify: ________________________<br>88. Don’t know/not sure<br>99. Prefer not to say
108. Since giving birth, how much money have you spent out of pocket on all other health care for yourself? Do not include costs of childbirth. Your best estimate is fine. [Select ONE]<br><br>1, I did not spend any money out of pocket<br>2, $0-$500<br>3, $501 - $1,000<br>4, $1,001 - $3,000<br>5, $3,001 - $5,000<br>6, $5,001 - $7,000<br>7, $7,001 - $9,000<br>8, Greater than $9,001<br>88, Don’t know/not sure<br>99, Prefer not to say

109. In general, how worried are you about paying your health care bills? [Select ONE.]

1. Not at all worried
2. Somewhat worried
3. Very worried

88. Don't know/not sure
99. Prefer not to say

Section 2: Health

201. In general, how would you describe your current **PHYSICAL** health? [Select ONE.]
1. Excellent
2. Very good
3. Good
4. Fair
5. Poor
88. Don't know/not sure
99. Prefer not to say

202. In general, how would you describe your current **MENTAL** health? [Select ONE.]
1. Excellent
2. Very good
3. Good
4. Fair
5. Poor
88. Don't know/not sure
99. Prefer not to say

203. Have you ever been told by a doctor, nurse, or other health professional that you have any of the following conditions? [Select an answer for EACH option.]

	YES	NO	Don't know/ Not sure	Prefer not to say
1. High blood pressure or hypertension				
2. Diabetes or high blood sugar				
3. Anxiety or depression				
4. Overweight or obesity				

204. Since giving birth, have you received any of the following treatments or support for your **emotional** or **mental health**? [Select an answer for EACH option.]

	YES	NO	Don't know/ Not sure	Prefer not to say
1. Counseling or therapy				
2. Medication for mood, anxiety, or depression				
3. Treatment at a hospital or emergency department				
4. Support group or care from an in-home visiting health professional (examples: nurse, midwife, doula)				
5. Support from a social worker				

205. What kind of birth control are you or your spouse/partner currently using? [Select all that apply.]<br><br>1, Not using any birth control<br>2, Birth control pills, shots/injections, patch or vaginal ring<br>3, Intrauterine device or IUD (Mirena, ParaGuard, Liletta, Kyleena, etc.)<br>4, Contraceptive implant in the arm (Norplant, Implanon, Nexplanon, etc.)<br>5, Condoms<br>6, Withdrawal (pulling out) or calendar rhythm/cycle-based method<br>7, Not having sex (abstinence)<br>8, Have had a hysterectomy or tubes tied/blocked<br>9, Partner has vasectomy<br>10, I cannot get pregnant due to the sex of my partner<br>88, Don't know/not sure<br>99, Prefer not to say
206. Since giving birth, have you gotten health care from any of the following types of specialty health care providers? SELECT ALL THAT APPLY<br><br>1, Cardiology (heart and blood specialist)<br>2, Endocrinology (diabetes, thyroid, and hormones specialist)<br>3, Allergy or Immunology (immune system, allergies specialist)<br>4, Gynecology<br>5, Nephrology (kidney specialist)<br>6, Oncology (cancer specialist)<br>7, Psychiatry (mental health specialist)<br>8, Psychology / Therapy<br>9, Pulmonology (lung specialist)<br>10, Surgery<br>11, Urology or urogynecology (urinary tract specialist)<br>12, Other specialist not mentioned above (please list) ____<br>88, Don't know/ not sure<br>99, Prefer not to say

Section 3A: No Primary Care

3A01. Please indicate ALL of the reasons why you <u>do not currently have a primary care provider</u>.
[Select an answer for EACH option.]

	YES	NO	Don't know/ Not sure	Prefer not to say
1. I don't feel I need one				
2. I don't know how to find one				
3. I want to find one but never have time				
4. I am worried about the cost				
5. I can't find one that is accepting new patients or has appointments available				
6. I can't find one that accepts my insurance				
7. I can't find one that speaks my preferred language				
8. I can't find one I can trust				
9. Other reason, please specify:				

Section 3B. For individuals with Primary Care Provider and Appointment

3B01. Since giving birth, how important do you feel your primary care visits (either in-person or telemedicine) were for you in helping to manage your overall health?

1, Extremely important
2, Somewhat important
3, Neither important nor unimportant
4, Somewhat unimportant
5, Extremely unimportant
88, Don't know/not sure
99, Prefer not to say

3B02. Since giving birth, what were the reasons for your visit(s) with the primary care provider? *This does not include visits to your* ***pregnancy-care provider (OB/GYN or midwife)*** *OR to an emergency department or urgent care center* [Select an answer for EACH option.]

	YES	NO	Don't know/ Not sure	Prefer not to say
1. Regular checkup (annual visit)				
2. Sick care (examples: an illness, injury, infection)				
3. To talk about a specific medical problem (examples: high blood pressure or diabetes)				
4. To discuss a mental health or mood concern				
5. To discuss and/or get birth control				
6. To get a prescription				
7. To get a vaccine				
8. Other reason, please specify:				

3B03. Since giving birth, in any of your primary care visits or phone calls, did the doctor or nurse ever do any of the following? [Select an answer for EACH option.]

	YES	NO	Don't know/ Not sure	Prefer not to say
1. Asked me questions about my mental health and wellbeing				
2. Prescribed me medication or referred me to a therapist/psychiatrist for my mental health				
3. Checked my weight and height				

4. Talked to me about ways to manage my weight				
5. Asked me questions or talked to me about my blood sugar or diabetes				
6. Perform a blood test for diabetes				
7. Checked my blood pressure				
8. Talked to me about ways to manage my blood pressure				

3B04. The last time you saw your primary care provider, did you have to pay for the visit?

1. I had to pay a fee at the time of the visit only.
2. I received a bill after the visit only.
3. I had to pay a fee at the time of the visit AND also received a bill after the visit.
4. I did not have to pay at the time of the visit and did not receive a bill.
88. Don’t know/ not sure
99. Prefer not to say

3B05. The last time you saw your primary care provider, did you receive a reminder about your appointment through Patient Gateway?

1, Yes, from my provider's office
2, Yes, from the Bridge Study team
3, Yes, from my provider's office and the Bridge Study team
4, No
88, Don't know / not sure
99, Prefer not to say

3B06. The last time you saw your primary care provider, did you receive a text message reminder to your phone about your appointment?

1, Yes, from my provider's office
2, Yes, from the Bridge Study team
3, Yes, from my provider's office and the Bridge Study team
4, No
88, Don't know / not sure
99, Prefer not to say

Section 3C: Primary Care (No visit)

3C01. Please indicate ALL of the reasons you have NOT had an appointment with a primary care provider since giving birth.

	YES	NO	Don't know/ Not sure	Prefer not to say
1. I have an appointment scheduled in the future				
2. I had no need or reason to see them				
3. I was concerned about the cost				
4. I don't know who to make an appointment with				
5. I couldn't get an appointment quickly enough				
6. I was unable to miss work				
7. I couldn't get childcare so I could go to the appointment				
8. I didn't have transportation to the appointment				
9. I didn't have the energy or feel well enough.				
10. I don't have a primary care doctor				
11. Other reason, please specify:				

Section 4: Urgent or Emergency Care

401. Since giving birth, which emergency department(s) or urgent care center(s) did you go to get care for yourself? (Show if 105=1)
List the names of all hospital emergency rooms or urgent care centers you visited.

Name 1: ________________________________
Name 2: ________________________________
Name 3: ________________________________
Name 4: ________________________________

88. Don't know/not sure
99. Prefer not to say

402. The LAST TIME you went to the emergency department or urgent care center for yourself, what was the reason you went there? [Select an answer for EACH option.]

	YES	NO	Don't know/ Not sure	Prefer not to say
1. I needed emergency care				
2. My doctor instructed me to go to an urgent care or emergency department				
3. My doctors' offices were closed				
4. I couldn't get an appointment to my doctor soon enough				
5. I didn't have a doctor or primary care provider				
6. I couldn't afford the cost to see a doctor				
7. I needed a prescription drug				
8. I didn't know where else to go				
9. Some other reason, please specify:				

Section 5: Postpartum Obstetric Care (Only shown if did not say No PP Visit)

501. Since giving birth, has your OBGYN or midwife discussed any of the following with you in your appointments (either in person or by phone or video)? [Select an answer for EACH option.]

	YES	NO	Don't know/ Not sure	Prefer not to say
1. My mental health and wellbeing				
2. How to manage my blood pressure or hypertension				
3. How to manage my weight				
4. How to manage my blood sugar or diabetes				
5. My plan for birth control or future pregnancies				
6. Recommendation to see a primary care doctor				
7. Recommendation to see or talk with a social worker				

Section 6: Postpartum Leave, Work and Childcare

601. How many children (under age 18) live in your household? Only include children who live with you at least 50% of the time.

1, 1
2, 2
3, 3
4, 4
5, 5
6, 6
7, 7
8, 8 or more
88, Don't know/not sure
99, Prefer not to say

602. How many adults (age 18 or older), including yourself, live in your household? Only include adults who live with you at least 50% of the time.

1, 1
2, 2
3, 3
4, 4
5, 5
6, 6
7, 7
8, 8 or more
88, Don't know/not sure
99, Prefer not to say

603. Since giving birth, did anyone in your household receive assistance from any of the following sources? [Select an answer for EACH option.]

	YES	NO	Don't know/ Not sure	Prefer not to say
1. Unemployment compensation or worker's compensation				
2. Cash assistance from a state or county welfare program				
3. Supplemental Nutrition Assistance Program (SNAP)				
4. Woman, Infants and Children Nutrition Program (WIC)				
5. Help paying for childcare from a government agency				
6. Federal, state or local government housing program that lowers your rent or provides a housing voucher				
7. Income from SSI (Supplemental Security Income), a federal program to help older adults, blind, and disabled people				

8. Other government program(s) not listed here, please specify:

604. What is your yearly total household income now, before taxes? Include income from all family members living in your household (your income, your spouse's or partner's income, and any other income you may have received). **All information will be kept private and will not affect any services you are now getting.** [Select ONE]

1. $ 0 - $ 9,999
2. $10,000-$19,999
3. $ 20,000 – $ 29,999
4. $30,000 – $ 39,999
5. $40,000 - $59,999
6. $60,000 - $79,999
7. $80,000 - $99,999
8. $100,000 - $119,999
9. $120,000 - $149,999
10. $150,000 - $174,999
11. $175,000 - $199,999
12. $200,000 or above

88. Don't know/not sure
99. Prefer not to say

606. Are you currently working for pay? [Select ONE.]

1. Yes, I am working part time
2. Yes, I am working full time
3. No. -→ Skip to Question 612

88. Don't know/not sure
99. Prefer not to say

607. How many weeks after your delivery did you begin working?

_________ weeks [0-100 NUMBER]

608. Do you plan on either returning to work or starting a new job in the next year? [Select ONE.]

1. I don't plan to return to work or start a new job in the next year
2. Yes, I plan to start working within the next 1 month
3. Yes, I plan to start working within the next 2 – 3 months
4. Yes, I plan to start working ore than 3 months from now

88. Don't know/not sure
99. Prefer not to say

605. How well did you understand the maternity leave benefits that were available to you?

1. Extremely well
2. Very well
3. Moderately well
4. Somewhat well
5. Not well at all
6. I was not working for pay prior to giving birth and therefore did not have any leave benefits

88. Don't know/not sure
99. Prefer not to say

609. Since giving birth, which types of parental leave have you used? [Select ALL THAT APPLY] (SHOW ONLY IF 605==1-5)

1. Paid parental leave provided by my employer
2. Unpaid parental leave provided by my employer
3. Massachusetts Paid Family and Medical Leave (MA PFML)
4. Temporary Disability Insurance or Short-Term Disability Insurance
5. Paid Time Off / Paid Vacation Days (Sometimes called PTO)
6. Sick Leave
7. Unpaid Time Off
8. Other, please specify_________________

88. Don't know/not sure
99. Prefer not to say

610. Since giving birth, how many weeks of PAID parental leave have you taken in total? *Your best estimate is fine.* [SHOW ONLY IF 608= (1 OR 2 OR 3)]

_________ [0-100, NUMBER]

611. Since giving birth, how many weeks of UNPAID parental leave have you taken in total? *Your best estimate is fine.* [SHOW ONLY IF 608= (1 OR 2 OR 3)]

_________ [0-100, NUMBER]

612. Since delivery, how many weeks of parental leave/time-off did your spouse, partner, or the baby's father take from work?

0, None
1, 1-2 weeks
2, 3-4 weeks
3, 5-6 weeks
4, 7-8 weeks
5, 9-10 weeks
6, 11-12 weeks
7, 13-16 weeks
8, 17+ weeks
77, Not applicable
88, Don't know/not sure
99, Prefer not to say

613. In the last 30 days, who was your usual childcare provider? [Select ONE.]

1. Me
2. Spouse or partner
3. Baby's grandparent
4. Other close family members or relatives
5. Friend or neighbor
6. Babysitter, nanny, or other childcare provider
7. Staff at home-based or day care center
8. Other, please specify:______________________
88. Don't know/not sure
99. Prefer not to say

Section 7: Experiences with Intervention

701. The study you have participated in aimed to help you to transition to primary care after childbirth. **How important was seeing your primary care provider after birth for you?** [Select ONE.]
1. Extremely important
2. Somewhat important
3. Not very important
4. Not important at all
88. Don't know/not sure
99. Prefer not to say

703. How helpful would or did you find each of the following actions in reconnecting you to your primary care provider? [Select an answer for EACH option.]

	Extreme ly helpful	So me wha t help ful	Not very helpf ul	Not helpf ul at all	Don't know / Not sure	Prefe r not to say
1. Information about the role of primary care after your delivery						
2. Scheduling of your primary care appointment for you						
3. Text messaging to remind you about your appointment						

88. Don't know/not sure
99. Prefer not to say

704. In your own words, what assistance would you find or have found helpful in reconnecting with your primary care provider after delivery?
[Free text]

88. Don't know/not sure
99. Prefer not to say

705. If given the opportunity, would you participate in this study again?

1, Yes
2, No
88, Don't know / not sure
99, Prefer not to say

Email Text for REDCap Survey

Subject Line: “MGH OB Bridge Study Follow-up Survey”

Body Text:

Dear Bridge Study Participant,

THANK YOU for participating in the Bridge Study in the MGH Department of Obstetrics and Gynecology!

Please complete this short survey about the care you received after your delivery: [survey-link]

Participants who complete the survey will receive a **$20 Amazon gift card**. Your responses will not be shared with anyone outside of the research team, including your doctors or other care team members.

Thank you for your help and contribution to this research study. Please feel free to contact us with questions or concerns.

MGH Bridge Study
bridgestudy@mgh.harvard.edu
617-643-5483

--

If the link above does not work, try copying the link below into your web browser:
[survey-url]

This link is unique to you and should not be forwarded to others.

SMS Text Message
For individuals who consented to receive SMS messages

"MGH Bridge Study Message: Please check your email to complete a short survey. Respondents will receive a $20 gift card. Thank you!"

Title: Bridging the Gap from Postpartum to Primary Care: A Behavioral Science Informed Intervention to Improve Chronic Disease Management among Postpartum Women (the Bridge Study)

Sponsor Name:

PI Name: Clapp, Mark A **Protocol #:** 2022P001723 **Type:** Amendment (AME14)

Date Received: April 02, 2023

Signatures

PI Name: Clapp, Mark A, MD, MPH

Authenticated: April 02, 2023

Amendment

COVID-19 Amendment

Is this amendment ONLY related to research impacted by COVID-19?

Refer to MGB Policy on Conduct of Human Research Activities during COVID-19 Operations for description of Amendments which do **require prior IRB review and approval.**

- ◯ Yes
- ◉ No

Central IRB Performance Sites

Is this a protocol where the Mass General Brigham IRB is serving as the single IRB (sIRB) for external sites/institutions?

- ◉ Yes
- ◯ No

Would you like to 'Add' a Site?

- ◯ Yes
- ◉ No

Would you like to 'Remove' a Site?

- ◯ Yes
- ◉ No

Sponsor Amendment

Is there a sponsor amendment number?

- ◯ Yes
- ◉ No

Change in Protocol Status

Is this a cede protocol or project that was determined to be exempt, not human subjects research or not engaged in human subjects research?

- ◯ Yes
- ◉ No

Do you need to change the overall status of the protocol? For example, Re-Open to Enrollment or indicate that Research Interventions/Assessments Continue after telling the IRB these have ceased.

- ◯ Yes
- ◉ No

Briefly describe the proposed changes:

Addition of the Endline Survey Materials translated into Spanish with certification of translation

---

Provide rationale for the proposed changes:

To allow the endline survey to be sent to Spanish Speakers

Will the proposed change(s) significantly alter the risk to benefit assessment the IRB relied upon to approve the protocol?

- ◯ Yes
- ◉ No

Will the proposed change(s) significantly affect the integrity of the protocol?

- ◯ Yes
- ◉ No

Informed Consent

Do the changes require a revision to the consent form?

- ◯ Yes
- ◉ No

Attachments

Name	Mode
Endline_TranslationCertification (Other)	Electronic
Survey-Email-Text_031223-Es (Document for review)	Electronic
Survey-Remider-SMS-Text_031723-Es (Document for review)	Electronic
Bridge-Survey-Endline-_-final-Es (Document for review)	Electronic

The Spanish Group LLC
1 Park Plaza, Suite 600
Irvine, CA 92614
Estados Unidos de América
https://www.thespanishgroup.org

Traducción Certificada

Proporcionado el **27** de **marzo** de **2023**

Yo, Alexander Largaespada ( ), por medio de la presente certifico que traduje los presentes documentos de español a inglés o de inglés a español y que es una traducción correcta y fiel. Además, certifico que soy competente en la traducción tanto de español como de inglés, y que soy capaz de producir y certificar la validez de dicha traducción. Estos documentos no se han traducido para un familiar, amigo o socio comercial.

Yo, Salvador G. Ordorica, como Agente de Control de Calidad de The Spanish Group LLC, doy fe de que la persona mencionada es un traductor o traductora competente de español a inglés y de inglés a español. Por lo tanto, como representante autorizado de The Spanish Group, verifico que estos documentos han sido corregidos y que los siguientes documentos son traducciones correctas y fieles de sus originales.

Respetuosamente,

CERTIFIED The Spanish Group TRANSLATION

Salvador G. Ordorica
The Spanish Group LLC
(ATA #267262)

Texto de Email para Encuesta REDCap

Asunto: “Encuesta de Seguimiento del Estudio MGH OB Bridge”

Cuerpo del Texto:

Estimado Participante del Estudio Bridge,

!GRACIAS por participar en el Estudio Bridge del Departamento de Obstetricia y Ginecología de MGH!

Le rogamos complete esta breve encuesta sobre la atención recibida tras su parto: [link de la encuesta]

Quienes completen la encuesta recibirán una **tarjeta regalo de Amazon por valor de 20USD.** Sus respuestas no serán compartidas con nadie ajeno al equipo de investigación, incluyendo a sus médicos y otros miembros del equipo de atención sanitaria.

Gracias por su ayuda y contribución a este estudio. Si tiene alguna duda o inquietud no dude en contactarnos.

MGH Bridge Study
bridgestudy@mgh.harvard.edu
617-643-5483

--

Si el link de arriba no funciona pruebe copiando el siguiente link en su navegador:
[link de la encuesta]

Este link es personal y no debe ser reenviado a otros.

Mensaje de Texto SMS
Para personas que aceptaron recibir mensajes SMS

“Mensaje del Estudio MGH Bridge: Vea su email para completar una breve encuesta. Los participantes recibirán una tarjeta regalo por valor de 20 USD. ¡Gracias!”

Esquema Lógico de la Encuesta

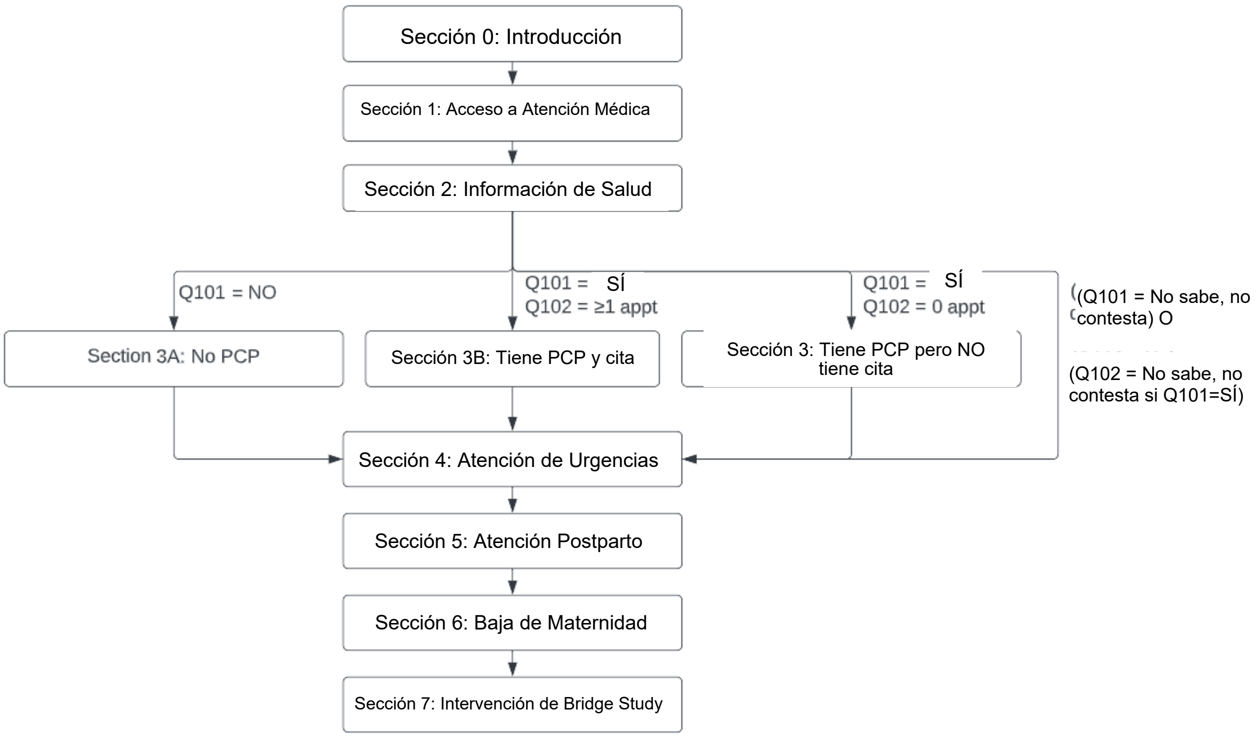

Acortando Distancias entre Atención Postparto y Atención Primaria

Encuesta Final

Autores del Estudio: Mark Clapp, MD MPH (MGH); Jessica Cohen, PhD (Harvard)
PI: Mark Clapp, MD MPH (MGH)

Sección 0: Texto Introductorio
Esta encuesta se administra como parte del Estudio MGH Bridge, en el que usted aceptó participar durante o poco después de su embarazo.

Completar la encuesta le tomará aproximadamente unos 5-10 minutos. Los participantes que completen la encuesta recibirán una tarjeta regalo de Amazon por valor de 20 USD.

Sus respuestas no serán compartidas con nadie ajeno al equipo de investigación, incluyendo sus médicos u otros proveedores de salud. Tampoco serán parte de su historial médico ni afectarán a su derecho a recibir atención o servicios en MGH.

Gracias por su contribución a este estudio. Si tiene alguna pregunta sobre el estudio no dude en contactarme.

Mark Clapp, MD MPH
Maternal-Fetal Medicine
Massachusetts General Hospital
bridgestudy@mgh.harvard.edu
617-724-4531

Al marcar SEGUIR, ACEPTO PARTICIPAR EN ESTA ENCUESTA.

Sección 1: Acceso a la Atención Sanitaria

100. ¿Su proveedor de asistencia durante el embarazo (obstetra, ginecólogo o partera) le alentó a visitar a su médico de cabecera después de dar a luz?

1. Sí
2. No
88. No lo sé/no estoy segura
99. Prefiero no contestar

101. ¿Tiene médico de cabecera actualmente? Entendido como alguien a quien uno ve para una revisión anual o un examen físico, o a quien consulta cuando se encuentra mal. *ESTO NO ALUDE A UN DEPARTAMENTO DE URGENCIAS* [Marcar UNO.]

1. Sí
2. No
88. No lo sé/no estoy segura
99. Prefiero no contestar

102. ¿Cuándo fue la última vez que vio a su médico de cabecera por cualquier motivo? *Esto no incluye las visitas a su* ***proveedor de asistencia durante el embarazo (obstetra, ginecólogo o partera)*** *ni las visitas a urgencias.* Puede usar meses o años en su respuesta. Conteste en la medida de sus conocimientos. *OBLIGATORIO

1, Más de 5 meses después de dar a luz
2, 3-4 meses después de dar a luz
3, 0-2 meses después de dar a luz
4, Durante el embarazo
5, 0-1 año antes de mi último embarazo
6, 2-3 años antes de mi último embarazo
7, Más de 3 años antes de mi último embarazo
88, No lo sé/no estoy segura
99, Prefiero no contestar

103. Al día de hoy, si necesita ver a su médico de cabecera para recibir asistencia si se encuentra enferma o para un examen físico anual, ¿cuánto le costaría hacer lo siguiente? [Seleccionar una respuesta por CADA opción]

	**Muy Difícil**	**Algo Difícil**	**Nada Difícil**	No lo sé	Prefiero no contestar
**1.** Conseguir una cita a corto plazo					
**2**. Conseguir transporte para la cita					
**3.** Pagar el coste de la visita					
**4.** Tomarme tiempo libre en el trabajo o la escuela para acudir a la cita					
**5**. Conseguir niñera para acudir a la cita					
**6.** Encontrar un médico disponible					

104. Desde que dio a luz, ¿tuvo cita con su ginecólogo u obstetra, o partera?
Esto es lo que suele llamarse una visita postparto.

1, Sí, sólo de forma presencial
2, Sí, sólo por vía telemática o telefónica
3, Sí, tanto visitas presenciales como por vía telemática/telefónica
4, No
5, No lo sé/no estoy segura
6, Prefiero no contestar

105. Desde que dio a luz, ¿visitó algún centro de emergencias para recibir atención para usted?

1, Sí
2, No
88, No lo sé/no estoy segura
99, Prefiero no responder

106. ¿Cuenta con seguro médico <u>actualmente</u>? [Marcar UNO.]

1. Sí
2. No
88. No lo sé/no estoy segura
99. Prefiero no responder

107. ¿Qué tipo de seguro médico tiene ahora? [Marcar TODAS las que apliquen] (Si 106=1)

1. Seguro médico de mi trabajo, escuela o del trabajo de mi cónyuge o pareja
2. Seguro médico de mis padres
3. Seguro médico estatal del Marketplace, Massachusetts Health Connector, o HealthCare.gov
4. Medicaid o MassHealth
5. Otro plan o programa gubernamental como SCHIP/CHIP
6. Otro plan o programa gubernamental no listado arriba como el programa MCH, el programa de indigencia o el programa de planificación familiar
7. TRICARE u otro plan de asistencia para militares/veteranos
8. Otro seguro médico, especificar: ________________________
88. No lo sé/no estoy segura
99. Prefiero no responder

108. Desde que dio a luz, ¿cuánto dinero ha gastado en atención sanitaria para usted? No incluir los gastos del parto. Responda en la medida de sus conocimientos. [Marcar UNO]

1, No gasté nada de dinero
2, $0-$500
3, $501 - $1,000
4, $1,001 - $3,000
5, $3,001 - $5,000
6, $5,001 - $7,000
7, $7,001 - $9,000
8, Más de $9,001
88, No lo sé/no estoy segura
99, Prefiero no responder

109. En general, ¿cuánto le preocupa tener que pagar sus facturas de atención sanitaria? [Marcar UNO]

1. No me preocupa nada
2. Me preocupa un poco
3. Me preocupa mucho

88. No lo sé/no estoy segura
99. Prefiero no responder

Sección 2: Salud

201. En general, ¿cómo describiría su salud **FÍSICA** actualmente? [Marcar UNO]
1. Excelente
2. Muy buena
3. Buena
4. Normal
5. Mala
88. No lo sé/no estoy segura
99. Prefiero no responder

202. En general, ¿cómo describiría su salud **MENTAL** actualmente? [Marcar UNO]
1. Excelente
2. Muy buena
3. Buena
4. Normal
5. Mala
88. No lo sé/no estoy segura
99. Prefiero no responder

203. ¿Alguna vez un médico, enfermera u otro profesional de la salud le dijo que tiene alguna de las siguientes condiciones? [Marcar una respuesta por CADA opción]

	SÍ	NO	No lo sé/no estoy segura	Prefiero no respond er
1. Presión alta o hipertensión				
2. Diabetes o azúcar alto en sangre				
3. Ansiedad o depresión				
4. Sobrepeso u obesidad				

204. Desde que dio a luz, ¿recibió alguno de los siguientes tratamientos para su **salud mental** o **emocional**? [Marcar una respuesta por CADA opción]

	´SÍ	NO	No lo sé/no estoy segura	Prefiero no respond er
1. Asesoría o terapia				
2. Medicación para el humor, ansiedad o depresión				
3. Tratamiento en hospital o departamento de urgencias				
4. Grupo de apoyo o asistencia de un profesional sanitario a domicilio (ejemplos: enfermera, partera, doula)				
5. Asistencia de un trabajador social				

205. ¿Qué tipo de anticonceptivo usa con su cónyuge <u>actualmente</u>? [Marcar todos los que apliquen]<br><br>1, No usamos anticonceptivos<br>2, Píldora anticonceptiva, inyecciones, parche o anillo vaginal<br>3, Dispositivo intrauterino o DIU (Mirena, ParaGuard, Liletta, Kyleena, etc.)<br>4, Implante anticonceptivo en el brazo (Norplant, Implanon, Nexplanon, etc.)<br>5, Preservativos<br>6, Retirada (antes de la eyaculación) o método de calendario/ciclos<br>7, No tener sexo (abstinencia)<br>8, Tengo hecha la histerectomía o ligadura de trompas<br>9, Mi cónyuge tiene la vasectomía<br>10, No puedo quedar embarazada debido al sexo de mi pareja<br>88. No lo sé/no estoy segura<br>99. Prefiero no responder
206. Desde que dio a luz, ¿ha recibido asistencia de alguno de los siguientes especialistas sanitarios? MARCAR TODOS LOS QUE APLIQUEN<br><br>1, Cardiólogo (especialista en corazón y flujo sanguíneo)<br>2, Endocrinólogo (especialista en diabetes, tiroides y hormonas)<br>3, Alergólogo o Inmunólogo (especialista en sistema inmunitario y alergias)<br>4, Ginecólogo<br>5, Nefrólogo (especialista en riñones)<br>6, Oncólogo (especialista en cáncer)<br>7, Psiquiatra (especialista en salud mental)<br>8, Psicólogo<br>9, Neumólogo (especialista en pulmones)<br>10, Cirujano<br>11, Urólogo o uroginecólogo (especialista en el tracto urinario)<br>12, Otro especialista (especificar) ____<br>88. No lo sé/no estoy segura<br>99. Prefiero no responder

Sección 3A: Sin Atención Primaria

3A01. Marque TODAS las razones por las que actualmente no cuenta con proveedor de atención primaria/médico de cabecera. [Marcar una respuesta para CADA opción]

	SÍ	NO	No lo sé/no estoy segura	Prefiero no responder
1. No siento que lo necesite				
2. No sé cómo encontrar uno				
3. Quiero encontrar uno pero nunca tengo tiempo				
4. Me preocupa el gasto				
5. No encuentro uno que acepte nuevos pacientes o tenga turnos disponibles				
6. No encuentro uno que acepte mi seguro				
7. No encuentro uno que hable mi idioma				
8. No encuentro uno en quien confíe				
9. Otro motivo, especificar:				

Sección 3B. Para personas con Proveedor de Atención Primaria y Turnos

3B01. Desde que dio a luz, ¿qué importancia le da a sus visitas al médico de cabecera (ya sea en persona o por vía telemática) en la gestión de su salud general?

1, Mucha importancia
2, Algo de importancia
3, No demasiada importancia
4, Poca importancia
5, Nada de importancia
88. No lo sé/no estoy segura
99. Prefiero no responder

3B02. Desde que dio a luz, ¿qué motivo su(s) visita(s) a su médico de cabecera? *Esto no incluye las visitas a su* ***obstetra, ginecólogo o partera*** *NI al departamento o centro de urgencias.* [Marcara una respuesta por CADA opción]

	SÍ	NO	No lo sé/no estoy segura	Prefiero no responder
1. Examen de rutina (visita anual)				
2. Visita por enfermedad, lesión, infección, etc.				
3. Para hablar sobre un problema médico específico (ejemplos: presión alta o diabetes)				
4. Para hacer una consulta relacionada con su salud mental				
5. Para hablar sobre/pedir anticonceptivos				
6. Para pedir una receta				
7. Para recibir una vacuna				
8. Otro motivo, especificar				

3B03. Desde que dio a luz, en alguna de sus visitas o llamadas a atención primaria, ¿el médico o enfermera hizo lo siguiente? [Marcar una respuesta por CADA opción]

	SÍ	NO	No lo sé/no estoy segura	Prefiero no responder
1. Preguntó por mi salud mental y bienestar				
2. Me recetó algo o me derivó a un terapeuta/psiquiatra por mi salud mental				
3. Comprobó mi peso y altura				

4. Me habló sobre formas de controlar mi peso				
5. Me hizo preguntas o habló sobre mi azúcar en sangre o diabetes				
6. Me hizo un análisis de sangre para controlar la diabetes				
7. Me tomó la presión				
8. Me habló sobre formas de controlar la presión sanguínea				

3B04. La última vez que visitó a su médico de cabecera, ¿tuvo que pagar por la visita?

1. Tuve que pagar una cuota sólo al momento de la visita.
2. Recibí una factura después de la visita.
3. Tuve que pagar una cuota al momento de la visita Y también recibí una factura tras la visita.
4. No tuve que pagar al momento de la visita ni recibí una factura.
88. No lo sé/no estoy segura
99. Prefiero no responder

3B05. La última vez que visitó a su médico de cabecera, ¿le llegó un recordatorio de su cita a través de Patient Gateway?

1, Sí, de la oficina de mi médico
2, Sí, del equipo de Bridge Study
3, Sí, de la oficina de mi médico y del equipo de Bridge Study
4, No
88, No lo sé/no estoy segura
99, Prefiero no responder

3B06. La última vez que visitó a su médico de cabecera, ¿recibió un mensaje de texto en su teléfono recordándole su cita?

1, Sí, de la oficina de mi médico
2, Sí, del equipo de Bridge Study
3, Sí, de la oficina de mi médico y del equipo de Bridge Study
4, No
88, No lo sé/no estoy segura
99, Prefiero no responder

Sección 3C: Atención Primaria (Sin visitas)

3C01. Por favor, indique TODOS los motivos por los que NO reservó una cita con su médico de cabecera desde que dio a luz.

	SÍ	NO	No lo sé/no estoy segura	Prefiero no responder
1. Tengo una cita programado en el futuro				
2. No tenía necesidad ni motivo para verlo				
3. Me preocupaba el coste				
4. No sé cómo reservar una cita				
5. No pude reservar una cita a tiempo				
6. No podía faltar al trabajo				
7. No tenía quien cuidase a mis hijos mientras iba al médico				
8. No tenía transporte para ir a la cita				
9. No tenía energía ni me sentía con fuerzas				
10. No tengo médico de cabecera				
11. Otro motivo, especificar				

Sección 4: Atención de Urgencias

401. Desde que dio a luz, ¿qué departamento(s) o centro(s) de urgencias visitó para recibir asistencia? (Mostrar si 105=1)
Indicar los nombres de todas las salas o centros de urgencias que visitó.

Nombre 1: ______________________________
Nombre 2: ______________________________
Nombre 3: ______________________________
Nombre 4: ______________________________

88. No lo sé/no estoy segura
99. Prefiero no responder

402. La ÚLTIMA VEZ que acudió a un departamento o centro de urgencias para recibir asistencia para usted, ¿cuál fue el motivo de su visita? [Marcar una respuesta por CADA opción]

	SÍ	NO	No lo sé/no estoy segura	Prefiero no responder
1. Necesitaba atención urgente				
2. Mi médico me indicó que fuera a un departamento o centro de urgencias				
3. El consultorio de mi médico estaba cerrado				
4. No puede reservar una cita a tiempo con mi médico				
5. No tenía médico de cabecera				
6. No podía pagar la visita al médico				
7. Necesitaba una receta				
8. No sabía dónde más ir				
9. Otro motivo, especificar:				

Sección 5: Atención Obstétrica Postparto (Sólo aparece si no se marcó como No PP Visit)

501. Desde que dio a luz, ¿Su gineco-obstetra o partera habló con usted de alguno de los siguientes temas en sus citas (ya sea en persona o por teléfono/videollamada)? [Seleccionar una respuesta por CADA opción]

	SÍ	NO	No lo sé/no estoy segura	Prefiero no responder
1. Mi salud mental y bienestar				
2. Control de mi presión sanguínea o hipertensión				
3. Cómo controlar mi peso				
4. Cómo controlar mi azúcar en sangre o diabetes				
5. Mi plan anticonceptivo o de embarazos futuros				
6. Derivación a un médico de atención primaria				
7. Recomendación de ver a hablar con un trabajador social				

Sección 6: Licencia de Maternidad, Trabajo y Cuidado de Niños

601. ¿Cuántos niños (menores de 18 años) viven en su casa? Incluir sólo a los niños que vivan con usted al menos el 50% del tiempo.

1, 1
2, 2
3, 3
4, 4
5, 5
6, 6
7, 7
8, 8 o más
88, No lo sé/no estoy segura
99, Prefiero no responder

602. ¿Cuántos adultos (mayores de 18 años), incluyéndola a usted, viven en su casa? Incluir sólo a los adultos que vivan con usted al menos el 50% del tiempo.

1, 1
2, 2
3, 3
4, 4
5, 5
6, 6
7, 7
8, 8 o más
88, No lo sé/no estoy segura
99, Prefiero no responder

603. Desde que dio a luz, ¿alguien en su casa recibió asistencia de alguno de los siguientes? [Marcar una respuesta por CADA opción]

	SÍ	NO	No lo sé/no estoy segura	Prefiero no responder
1. Prestación por desempleo o compensación por accidente				
2. Ayuda en efectivo de un programa benéfico estatal o del condado				
3. Programa de Asistencia de Nutrición Complementaria (SNAP)				
4. Programa de Nutrición de Mujeres, Infantes y Niños (WIC)				
5. Ayuda de una agencia gubernamental para pagar el cuidado de niños				
6. Programa de vivienda federal, estatal o local que disminuya su alquiler o le dé un vale de vivienda				
7. Ingresos del SSI (Supplemental Security Income), un programa federal de asistencia a adultos mayores, personas ciegas y discapacitadas				

8. Otro/s programa/s gubernamental/es no indicados aquí, especificar:

604. ¿Cuáles son sus ingresos familiares anuales actuales antes de impuestos? Incluir ingresos de todos los familiares que vivan en su residencia (sus ingresos, los ingresos de su cónyuge o pareja y cualquier otro ingreso recibido). **Esta información es privada y no afectará a los servicios que recibe actualmente.** [Marcar UNO]

1. $ 0 - $ 9,999
2. $10,000-$19,999
3. $ 20,000 – $ 29,999
4. $30,000 – $ 39,999
5. $40,000 - $59,999
6. $60,000 - $79,999
7. $80,000 - $99,999
8. $100,000 - $119,999
9. $120,000 - $149,999
10. $150,000 - $174,999
11. $175,000 - $199,999
12. $200,000 o más

88. No lo sé/no estoy segura
99. Prefiero no responder

606. ¿Trabaja a cambio de un sueldo actualmente? [Marcar UNO]

1. Sí, trabajo a tiempo parcial
2. Sí, trabajo a tiempo completo
3. No. -→ Pasar a la Pregunta 612

88. No lo sé/no estoy segura
99. Prefiero no responder

607. ¿Cuántas semanas después del parto empezó a trabajar?

_________ semanas [NÚMERO DEL 0-100]

608. ¿Planea volver a trabajar o comenzar en un nuevo trabajo durante el próximo año? [Marcar UNO]

1. No planeo volver a trabajar ni comenzar en un nuevo trabajo durante el próximo año
2. Sí, planeo empezar a trabajar durante el próximo mes
3. Sí, planeo volver a trabajar durante los próximos 2-3 meses
4. Sí, planeo volver a trabajar dentro de más de 3 meses

88. No lo sé/no estoy segura
99. Prefiero no responder

605. ¿Qué tal comprendió las prestaciones de baja por maternidad que tiene a su disposición?

1. Perfectamente
2. Muy bien
3. Moderadamente bien
4. Más o menos bien
5. Nada bien
6. No trabajaba antes de dar a luz y por lo tanto no tuve prestaciones de maternidad

88. No lo sé/no estoy segura
99. Prefiero no responder

609. Desde que dio a luz, ¿qué tipos de licencia por maternidad ha usado? [Marcar TODOS LOS QUE APLIQUEN] (MOSTRAR SÓLO SI 605==1-5)

1. Licencia remunerada proporcionada por mi empleador
2. Licencia no remunerada proporcionada por mi empleador
3. Licencia Familiar Pagada de Massachusetts y Licencia Médica (MA PFML)
4. Seguro Temporal por Discapacidad o Seguro por Discapacidad a Corto Plazo
5. Licencia Remunerada / Días de Vacaciones Remuneradas
6. Licencia por Enfermedad
7. Licencia No Remunerada
8. Otro, especificar_________________

88. No lo sé/no estoy segura
99. Prefiero no responder

610. Desde que dio a luz, ¿cuántas semanas de licencia por maternidad REMUNERADA se ha tomado en total? *Responda en la medida de sus conocimientos.* [MOSTRAR SÓLO SI 608= (1 O 2 O 3)]

_________ [NÚMERO DE 0-100]

611. Desde que dio a luz, ¿cuántas semanas de licencia por maternidad NO REMUNERADA se ha tomado en total? *Responda en la medida de sus conocimientos.* [MOSTRAR SÓLO SI 608= (1 O 2 O 3)]

_________ [NÚMERO DE 0-100]

612. Desde que dio a luz, cuántas semanas de licencia por paternidad/días libres se tomó su cónyuge/pareja/padre del bebé?

0, Ninguna
1, 1-2 semanas
2, 3-4 semanas
3, 5-6 semanas
4, 7-8 semanas
5, 9-10 semanas
6, 11-12 semanas
7, 13-16 semanas
8, 17+ semanas
77, No aplica
88, No lo sé/no estoy segura

99, Prefiero no responder
613. Durante los últimos 30 días, ¿quién fue su proveedor de cuidado infantil? [Marcar UNO]<br><br>1. Yo<br>2. Cónyuge o pareja<br>3. Abuelo/a del bebé<br>4. Otros parientes cercanos<br>5. Amigo/a o vecino/a<br>6. Niñera u otro proveedor de cuidados<br>7. Personal de guardería o centro de día<br>8. Otro, especificar: ______________________<br>88. No lo sé/no estoy segura<br>99. Prefiero no responder

Sección 7: Experiencias con Intervenciones

701. El estudio en el que ha participado tiene como meta asistirle en su transición a atención primaria después de dar a luz. **¿Qué importancia tuvo para usted ver a su médico de cabecera después del parto?** [Marcar UNO]
1. Muy importante
2. Algo importante
3. Poco importante
4. Nada importante
88. No lo sé/no estoy segura
99. Prefiero no responder

703. ¿Qué importancia le da/daría a cada una de las siguientes acciones a la hora de reconectarla con su proveedor de atención primaria [Seleccionar una respuesta por CADA opción]

	Muy importante	Algo importante	Poco importante	Nada importante	No lo sé/no estoy segura	Prefiero no responder
1. Información sobre el rol de la atención primaria después del parto						
2. Programación de sus turnos de atención primaria						
3. Mensajes de texto para recordar sus turnos						

88. No lo sé/no estoy segura
99. Prefiero no responder

704. En sus propias palabras, ¿qué asistencia le ayudó o le ayudaría a reconectar con su médico de cabecera después del parto?
[Texto libre]

88. No lo sé/no estoy segura
99. Prefiero no responder

705. Si tuviera la ocasión, ¿volvería a participar en este estudio?

1, Sí
2, No
88, No lo sé/no estoy segura
99, Prefiero no responder

Mass General Brigham

Title: Bridging the Gap from Postpartum to Primary Care: A Behavioral Science Informed Intervention to Improve Chronic Disease Management among Postpartum Women (the Bridge Study)

Sponsor Name:

PI Name: Clapp, Mark A **Protocol #:** 2022P001723 **Type:** Amendment (AME15)

Date Received: April 25, 2023

Signatures

PI Name: Clapp, Mark A, MD, MPH

Authenticated: April 25, 2023

Amendment

COVID-19 Amendment

Is this amendment ONLY related to research impacted by COVID-19?

Refer to MGB Policy on Conduct of Human Research Activities during COVID-19 Operations for description of Amendments which do **require prior IRB review and approval.**

- ◯ Yes
- ◉ No

Central IRB Performance Sites

Is this a protocol where the Mass General Brigham IRB is serving as the single IRB (sIRB) for external sites/institutions?

- ◉ Yes
- ◯ No

Would you like to 'Add' a Site?

- ◯ Yes
- ◉ No

Would you like to 'Remove' a Site?

- ◯ Yes
- ◉ No

Sponsor Amendment

Is there a sponsor amendment number?

- ◯ Yes
- ◉ No

Change in Protocol Status

Is this a cede protocol or project that was determined to be exempt, not human subjects research or not engaged in human subjects research?

- ◯ Yes
- ◉ No

Mass General Brigham

Do you need to change the overall status of the protocol? For example, Re-Open to Enrollment or indicate that Research Interventions/Assessments Continue after telling the IRB these have ceased.

- (●) Yes
- ( ) No

Indicate new status:

- ( ) Active, Open to Enrollment/Collection
- (●) Active, Closed to Enrollment/Collection
- ( ) Active, Long Term Follow-Up
- ( ) Active, Data Analysis Only

Briefly describe the proposed changes:

1) The study has achieved it's target sample size and enrollment is now closed. Follow-up is ongoing.

2) The SMS endline survey messages have been edited to include the link to the REDCap survey link, which is also being sent via email.

Provide rationale for the proposed changes:

We have poor response rates from emailing the survey. We expect response rates to increase when sent via SMS message.

Will the proposed change(s) significantly alter the risk to benefit assessment the IRB relied upon to approve the protocol?

- ( ) Yes
- (●) No

Will the proposed change(s) significantly affect the integrity of the protocol?

- ( ) Yes
- (●) No

Informed Consent

Do the changes require a revision to the consent form?

- ( ) Yes
- (●) No

Attachments

Name	Mode
Survey Remider SMS Text_042523 (Document for review)	Electronic
Survey-Remider-SMS-Text_042523-Es (Document for review)	Electronic

SMS Text Message
For individuals who consented to receive SMS messages

"MGH Bridge Study Message: Please check your email to complete a short survey. Respondents will receive a $20 gift card. Thank you! [redcap-survey-link]"

Mensaje de Texto SMS
Para personas que aceptaron recibir mensajes SMS

“Mensaje del Estudio MGH Bridge: Vea su email para completar una breve encuesta. Los participantes recibirán una tarjeta regalo por valor de 20 USD. ¡Gracias! [redcap-survey-link]"
