# Supplementary material for: Postpartum Primary Care Engagement Using Default Scheduling and Tailored Messaging: A Randomized Clinical Trial
Source: JAMA Netw Open. 2024 Jul 16;7(7):e2422500. doi: 10.1001/jamanetworkopen.2024.22500 (PMC11252898; doi:10.1001/jamanetworkopen.2024.22500)
Supplement: Supplement 3. — Data Sharing Statement [file jamanetwopen-e2422500-s003.pdf]

Data Sharing Statement

Clapp. Postpartum Primary Care Engagement Using Default Scheduling and Tailored Messaging. *JAMA Netw Open*. Published July 16, 2024.
doi:10.1001/jamanetworkopen.2024.22500

Data
Data available: Yes
Data types: Deidentified participant data, Data dictionary
How to access data: Primary data and source code will be made publicly available at the Abdul Latif Jameel Poverty Action Lab Dataverse (https://dataverse.harvard.edu/dataverse/jpal) once planned secondary analyses have been completed (expected by August 2026).
When available: beginning date: 08-01-2026

Supporting Documents
Document types: Statistical/analytic code
How to access documents: Primary data and source code will be made publicly available at the Abdul Latif Jameel Poverty Action Lab Dataverse (https://dataverse.harvard.edu/dataverse/jpal) once planned secondary analyses have been completed (expected by August 2026).
When available: beginning date: 08-01-2026

Additional Information
Who can access the data: Anyone requesting the data
Types of analyses: For any purpose
Mechanisms of data availability: Without investigator support
Any additional restrictions: The Dataverse Project asks that all users who download datasets from a Dataverse repository follow the following Community Norms (https://dataverse.org/best-practices/dataverse-community-norms).
